# Supplementary material for: Discovery of positive and purifying selection in metagenomic time series of hypermutator microbial populations
Source: PLoS Genet. 2022 Aug 18;18(8):e1010324. doi: 10.1371/journal.pgen.1010324 (PMC9426924; doi:10.1371/journal.pgen.1010324)
Supplement: S1 Text — (DOCX) [file pgen.1010324.s001.docx]

**S1 TEXT**

*Comparison of STIMS to a test for selection based on the Poisson distribution*

One class of statistical tests for selection in evolution experiments uses the Poisson distribution to model the expected distribution of mutations per genomic site per unit time under neutral evolution [1, 2]. Using this framework, significant deviations from the Poisson null expectation can indicate positive or purifying selection. In principle, Poisson methods should be simpler to implement and computationally cheaper than STIMS. However, methods based on the Poisson distribution may be sensitive to model misspecification, due to both local mutation biases that can cause deviation from the Poisson expectation in the absence of selection as well as mutation rate variability depending on mutation class (e.g., point mutations versus indels) [3]. Variations of the bootstrapping approach used by STIMS, on the other hand, can account for such mutational biases through randomization (Materials and Methods).

To better understand the relative strengths and weaknesses of STIMS in comparison to Poisson methods, we implemented the Poisson method used by Kinnersley *et al.* [2], and re-ran all of our positive control experiments on gold-standard sets of genes (Materials and Methods). All classes of mutations (missense, nonsense, synonymous, indels, and structural variants) were included for both STIMS and the Poisson method. Overall, both STIMS and the Poisson method give similar results. The Poisson method successfully recovers signals of purifying selection (*p* < 10^−14^) and positive selection (*p* < 10^−50^) on the respective sets of gold-standard genes. However, the Poisson method finds a weak signal of positive selection on the gold-standard set of genes under relaxed selection (*p* = 0.0397). The Poisson method finds a signal of purifying selection on most proteome sectors (FDR-corrected *p* < 10^−11^ for U-sector; FDR-corrected *p* < 0.00001 for A-sector; FDR-corrected *p* = 0.000162 for R-sector; FDR-corrected *p =* 0.00182 for S-sector; FDR-corrected *p* = 0.00217 for O-sector; FDR-corrected *p* = 0.391 for C-sector). Eigengenes show little to no evidence of selection under the Poisson method (FDR-corrected *p* = 0.0327 for Eigengene 2; the rest are nonsignificant). I-modulon regulators show evidence of very strong positive selection (FDR-corrected *p* < 10^−13^), while 16 I-modulons show evidence of either positive or purifying selection at a FDR-corrected *p*-value threshold of 0.01 (Supplementary Table 4). 14 out of these 16 I-modulons are significant by STIMS, while the other two, “flu-yeeRS” and “Pyruvate”, trend toward significance (Supplementary File S1). Finally, we used the Poisson method as a genome-wide screen for positive and purifying selection, both over all LTEE populations, and on each population individually. We report these results at an FDR-corrected *p*-value threshold of 0.01 in Supplementary File S3. Many of the genes showing evidence of positive selection have been reported before [1, 4, 6].

Altogether, this detailed comparison indicates the Poisson method is faster and is more practical for genome-wide scans for positive and purifying selection on single genes, but that its sensitivity comes at the price of more false positives. STIMS is more conservative than the Poisson method, and gives results that are more consistent with ground truth, as defined by the sets of gold-standard genes that we used for empirical validation. STIMS, unlike the Poisson method, also provides a picture of the tempo of evolution in a gene set over time, as well as changes in statistical significance over time. These visualizations are especially useful for observing *how* the tempo of evolutionary change in a gene set of interest changes over time, and for generating further hypotheses and predictions for empirical validation (Supplementary Files 1 and 2).

**REFERENCES**

1. Good BH, McDonald MJ, Barrick JE, Lenski RE, Desai MM. The dynamics of molecular evolution over 60,000 generations. Nature. 2017;551(7678):45-50.

2. Kinnersley M, Schwartz K, Yang D-D, Sherlock G, Rosenzweig F. Evolutionary dynamics and structural consequences of de novo beneficial mutations and mutant lineages arising in a constant environment. BMC biology. 2021;19(1):1-21.

3. Maddamsetti R, Grant NA. Divergent evolution of mutation rates and biases in the long-term evolution experiment with Escherichia coli. Genome Biology and Evolution. 2020. doi: 10.1093/gbe/evaa178.

4. Tenaillon O, Barrick JE, Ribeck N, Deatherage DE, Blanchard JL, Dasgupta A, et al. Tempo and mode of genome evolution in a 50,000-generation experiment. Nature. 2016;536(7615):165-70.

5. Maddamsetti R, Hatcher PJ, Green AG, Williams BL, Marks DS, Lenski RE. Core genes evolve rapidly in the long-term evolution experiment with Escherichia coli. Genome biology and evolution. 2017;9(4):1072-83.
