# Supplementary material for: Discovery of positive and purifying selection in metagenomic time series of hypermutator microbial populations
Source: PLoS Genet. 2022 Aug 18;18(8):e1010324. doi: 10.1371/journal.pgen.1010324 (PMC9426924; doi:10.1371/journal.pgen.1010324)
Supplement: S1 File — (PDF) [file pgen.1010324.s002.pdf]

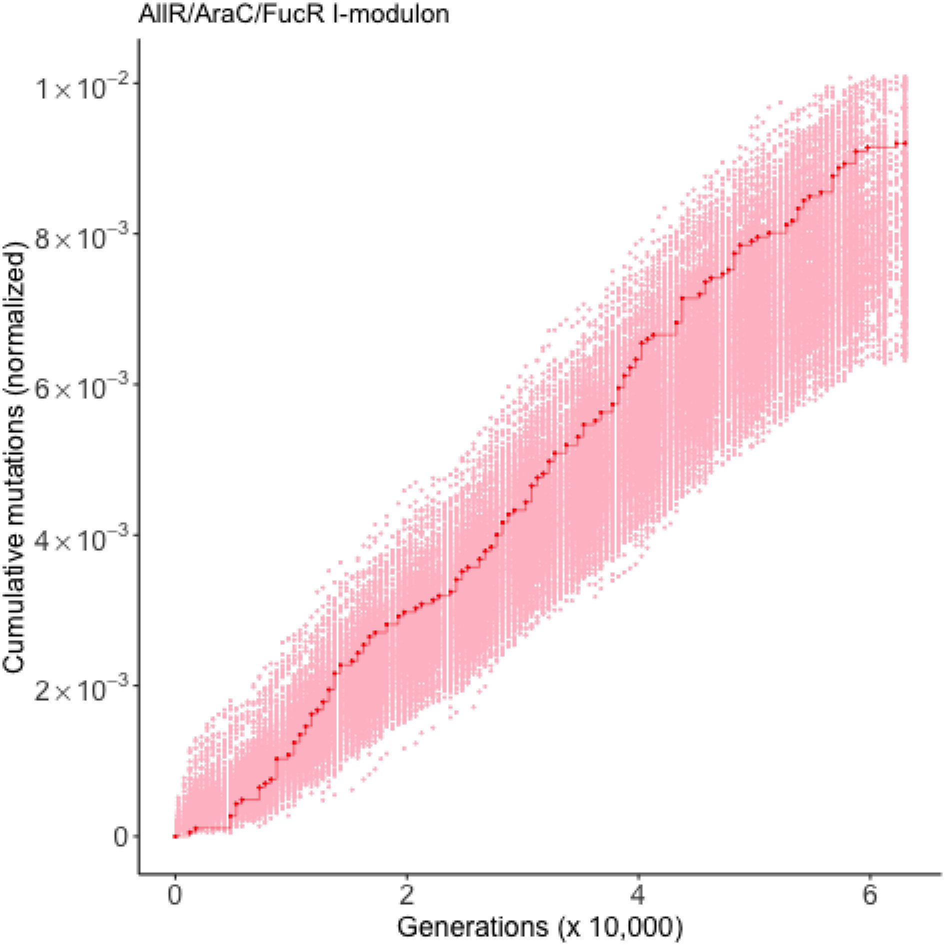

AllR/AraC/FucR I-modulon
Cumulative mutations (normalized)
1×10^{-2}
8×10^{-3}
6×10^{-3}
4×10^{-3}
2×10^{-3}
0
0
2
4
6
Generations (x 10,000)

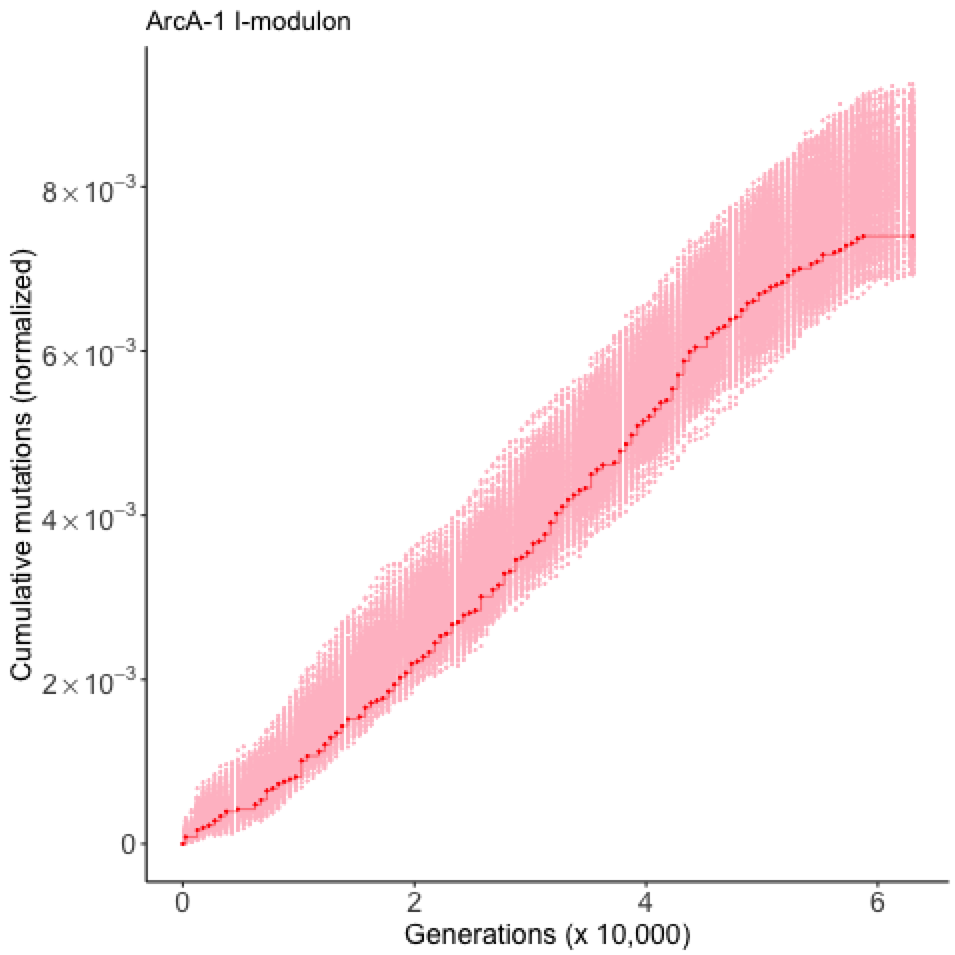

ArcA-1 I-modulon
Cumulative mutations (normalized)
8×10^{-3}
6×10^{-3}
4×10^{-3}
2×10^{-3}
0
0
2
4
6
Generations (x 10,000)

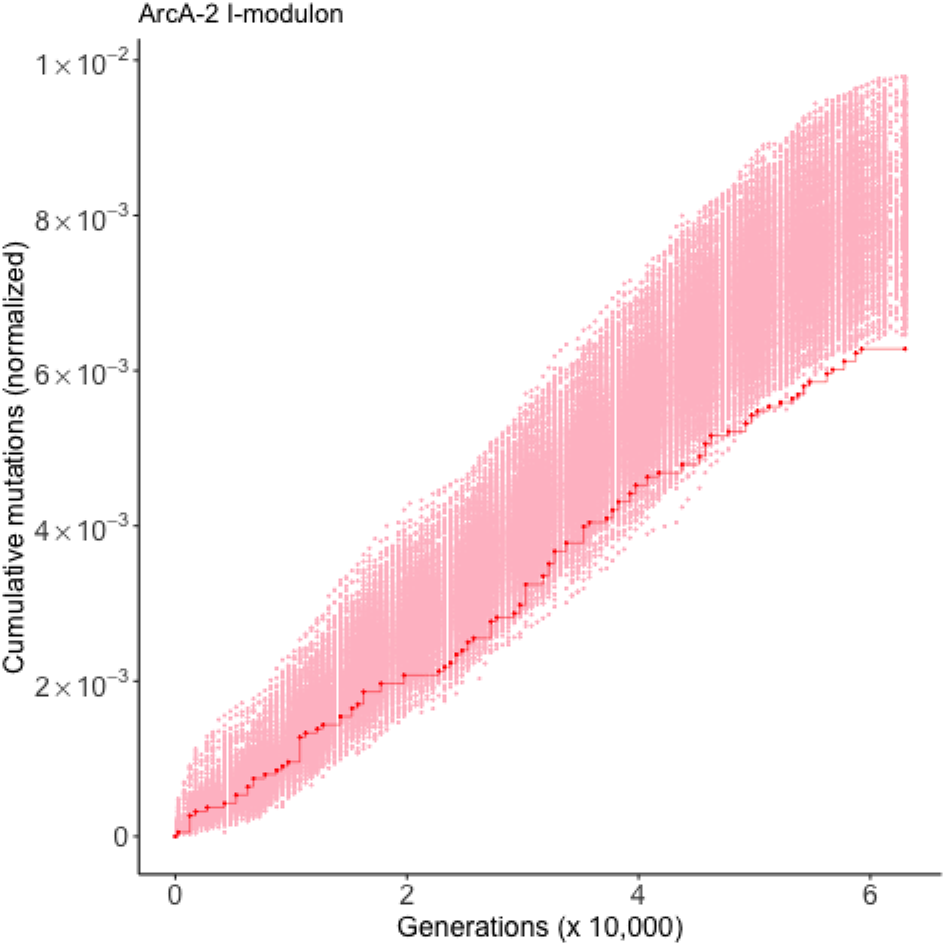

ArcA-2 I-modulon
Cumulative mutations (normalized)
1×10⁻²
8×10⁻³
6×10⁻³
4×10⁻³
2×10⁻³
0
0
2
4
6
Generations (x 10,000)

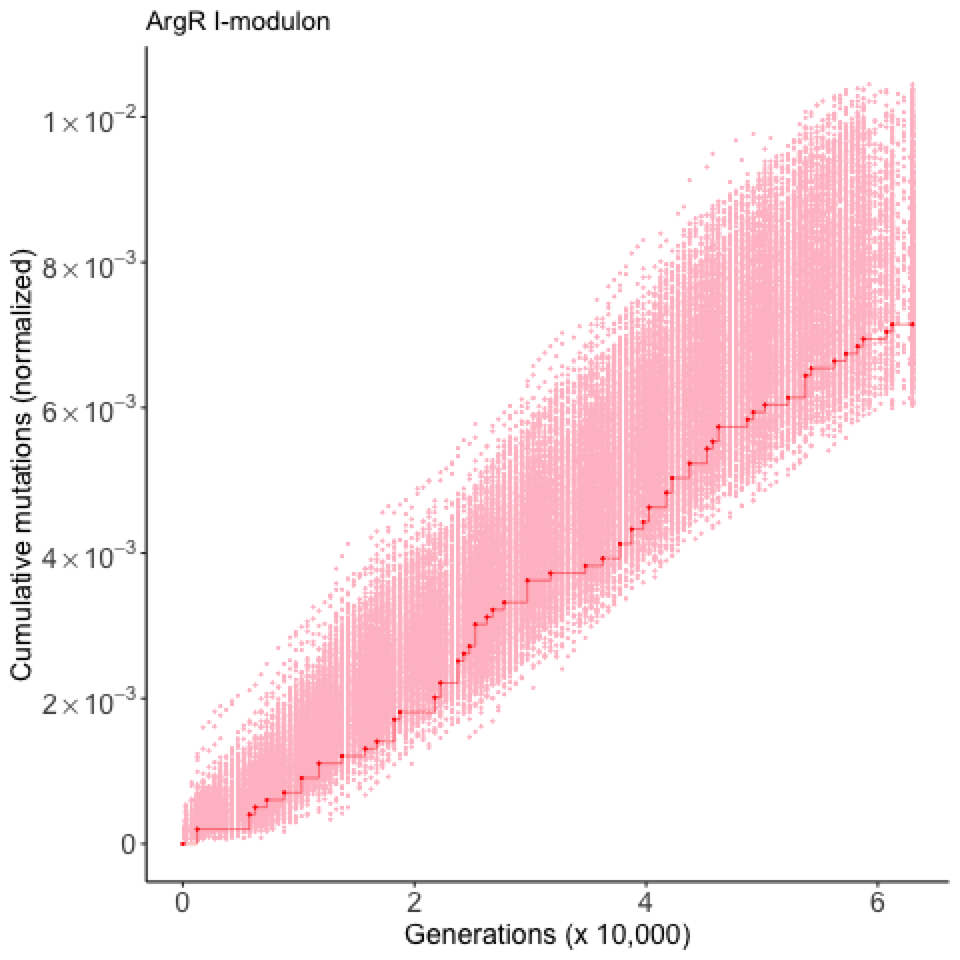

ArgR I-modulon
Cumulative mutations (normalized)
1×10^{-2}
8×10^{-3}
6×10^{-3}
4×10^{-3}
2×10^{-3}
0
0
2
4
6
Generations (x 10,000)

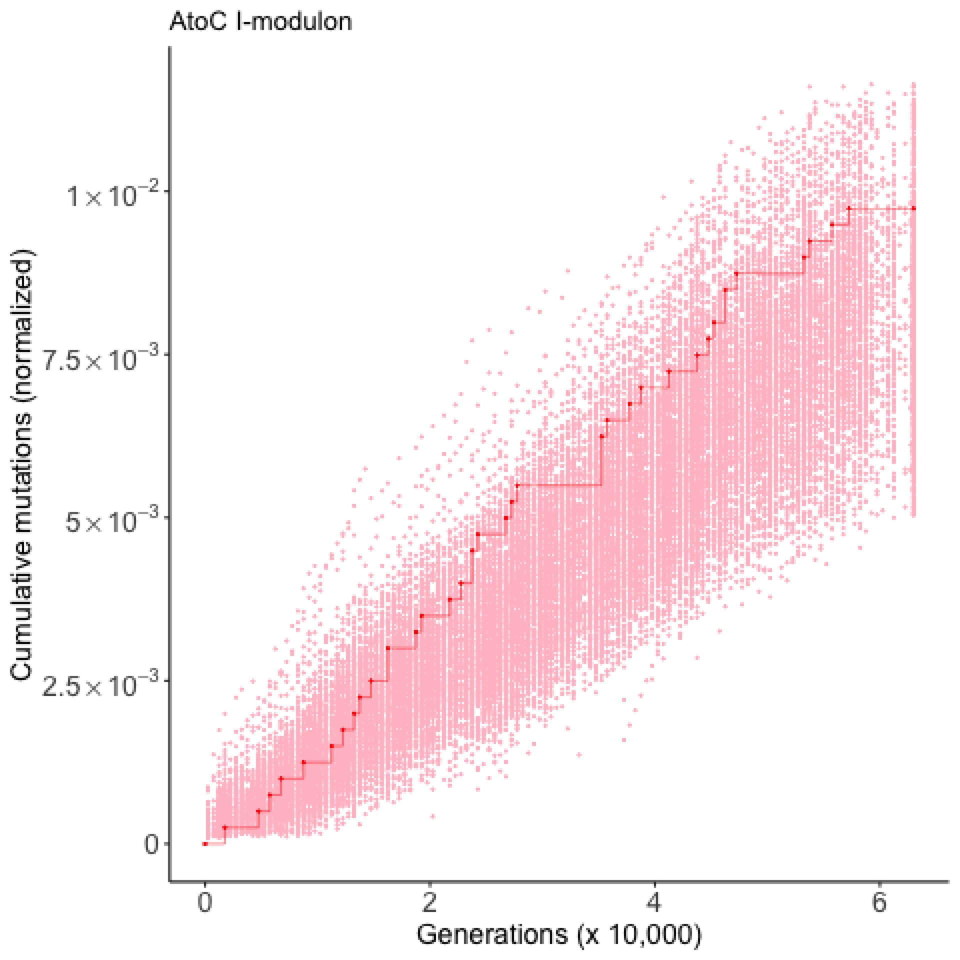

AtoC I-modulon
Cumulative mutations (normalized)
1×10^{-2}
7.5×10^{-3}
5×10^{-3}
2.5×10^{-3}
0
0
2
4
6
Generations (x 10,000)

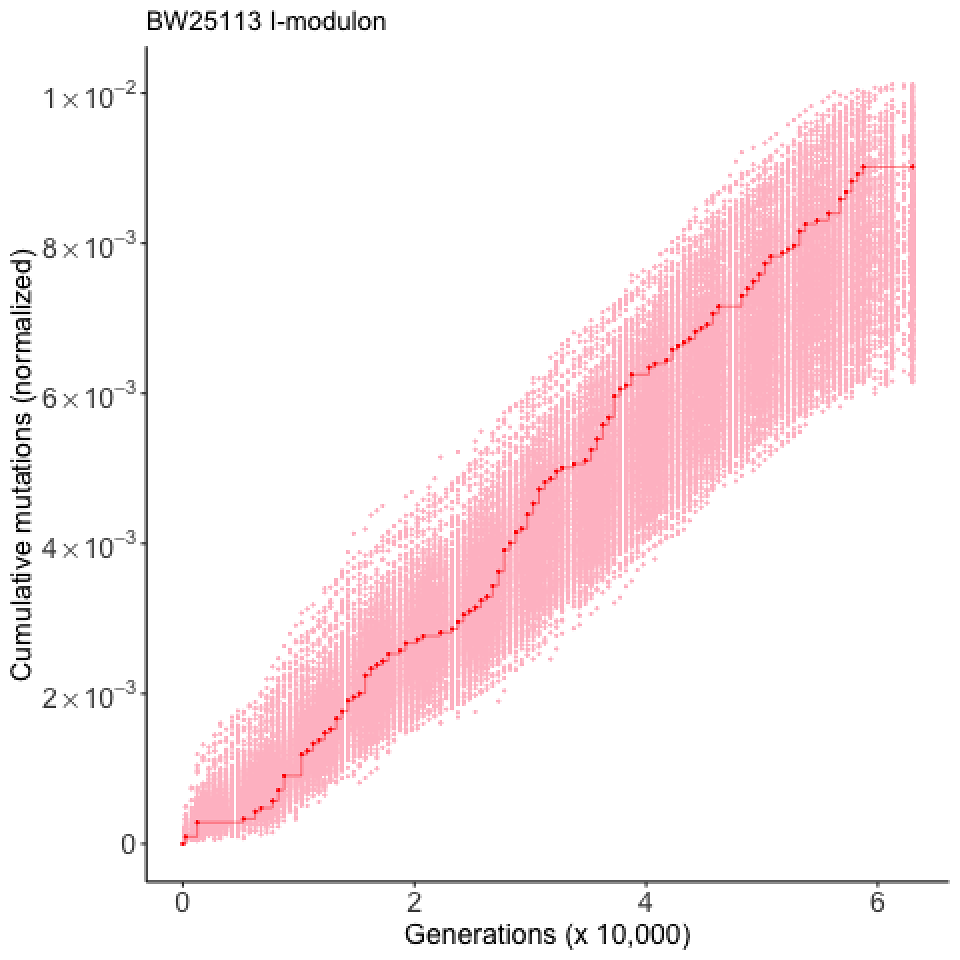

BW25113 I-modulon
Cumulative mutations (normalized)
1×10^{-2}
8×10^{-3}
6×10^{-3}
4×10^{-3}
2×10^{-3}
0
0
2
4
6
Generations (x 10,000)

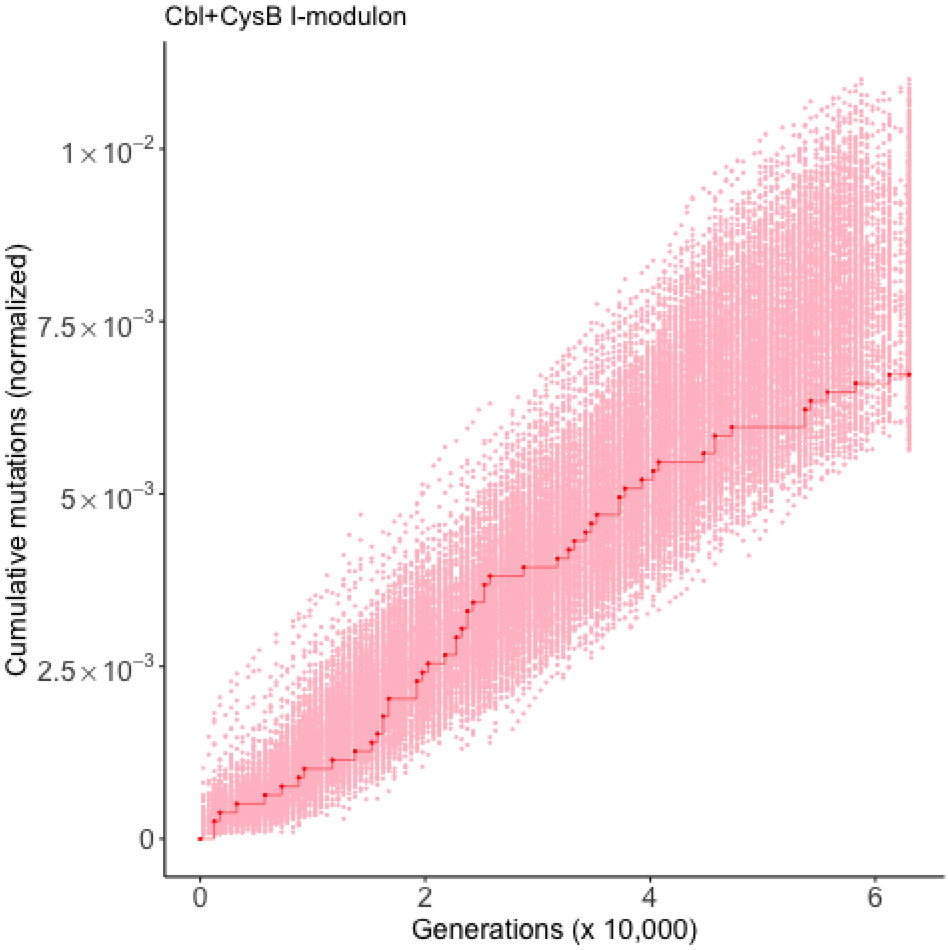
Cbl+CysB I-modulon
Cumulative mutations (normalized)
1×10^{-2}
7.5×10^{-3}
5×10^{-3}
2.5×10^{-3}
0
0
2
4
6
Generations (x 10,000)

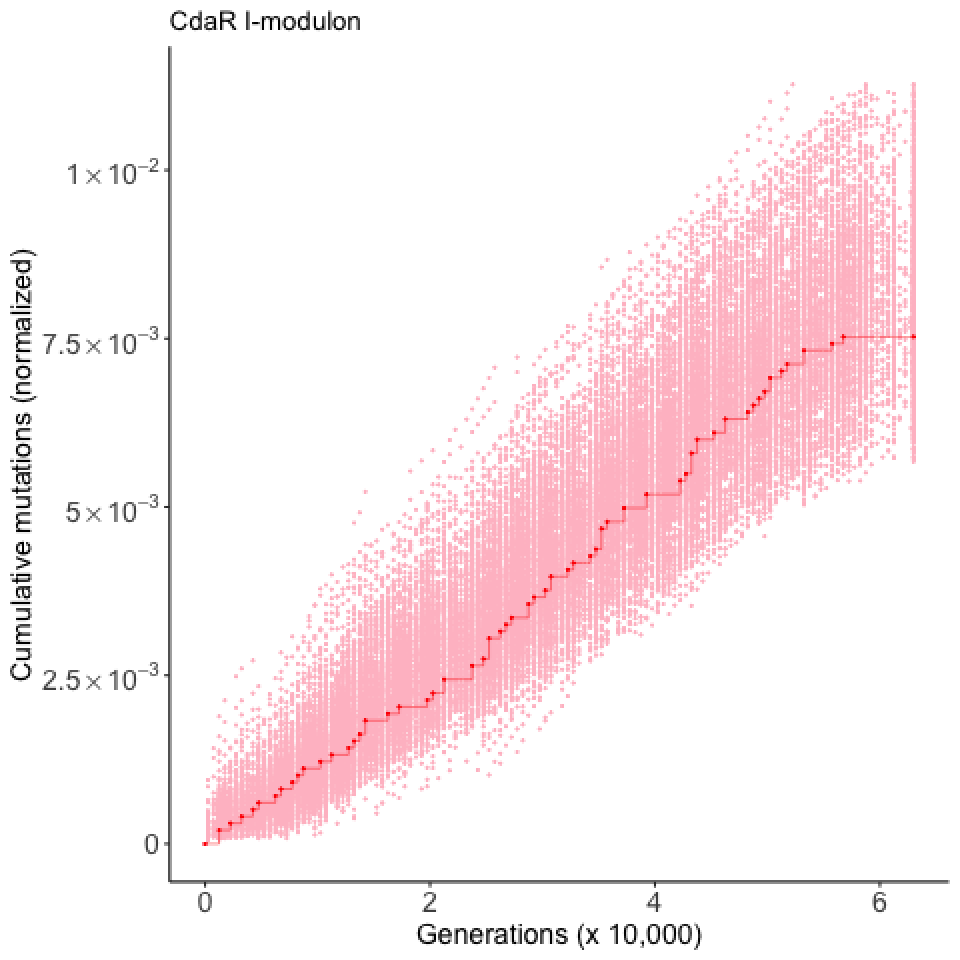
CdaR I-modulon
Cumulative mutations (normalized)
1×10^{-2}
7.5×10^{-3}
5×10^{-3}
2.5×10^{-3}
0
0
2
4
6
Generations (x 10,000)

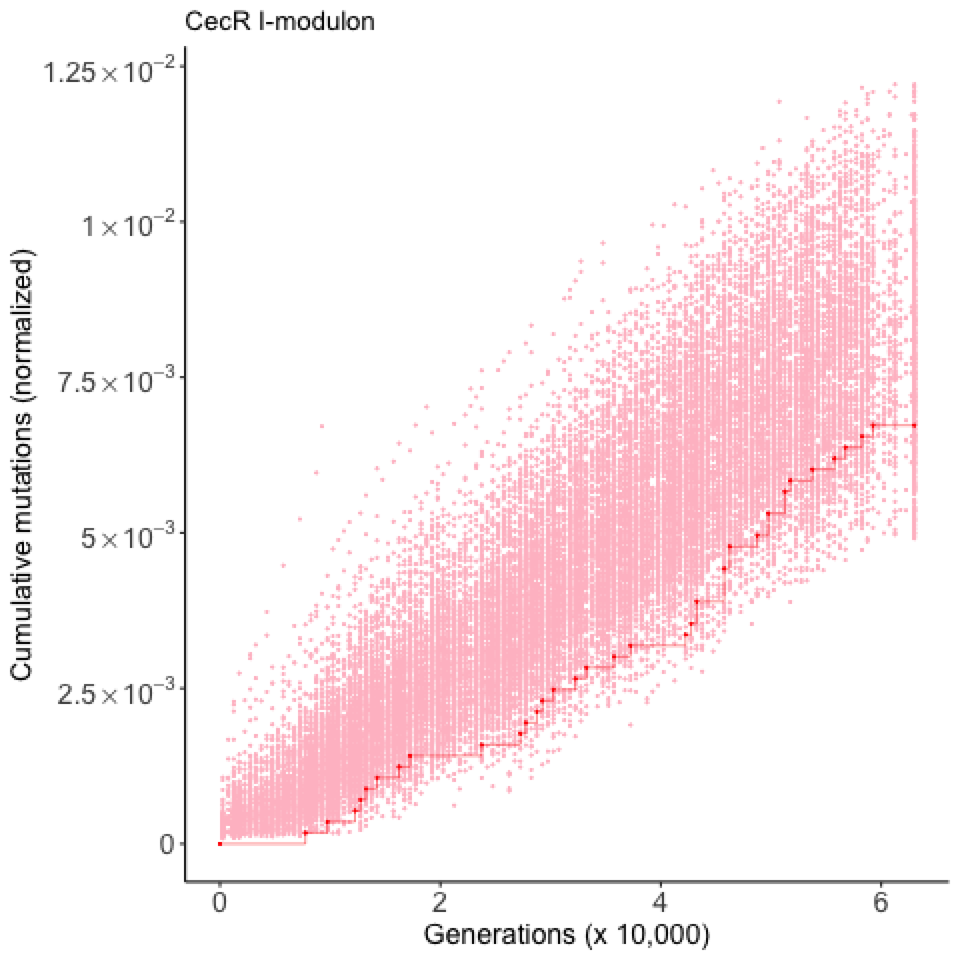

CecR I-modulon
Cumulative mutations (normalized)
1.25×10^-2
1×10^-2
7.5×10^-3
5×10^-3
2.5×10^-3
0
0
2
4
6
Generations (x 10,000)

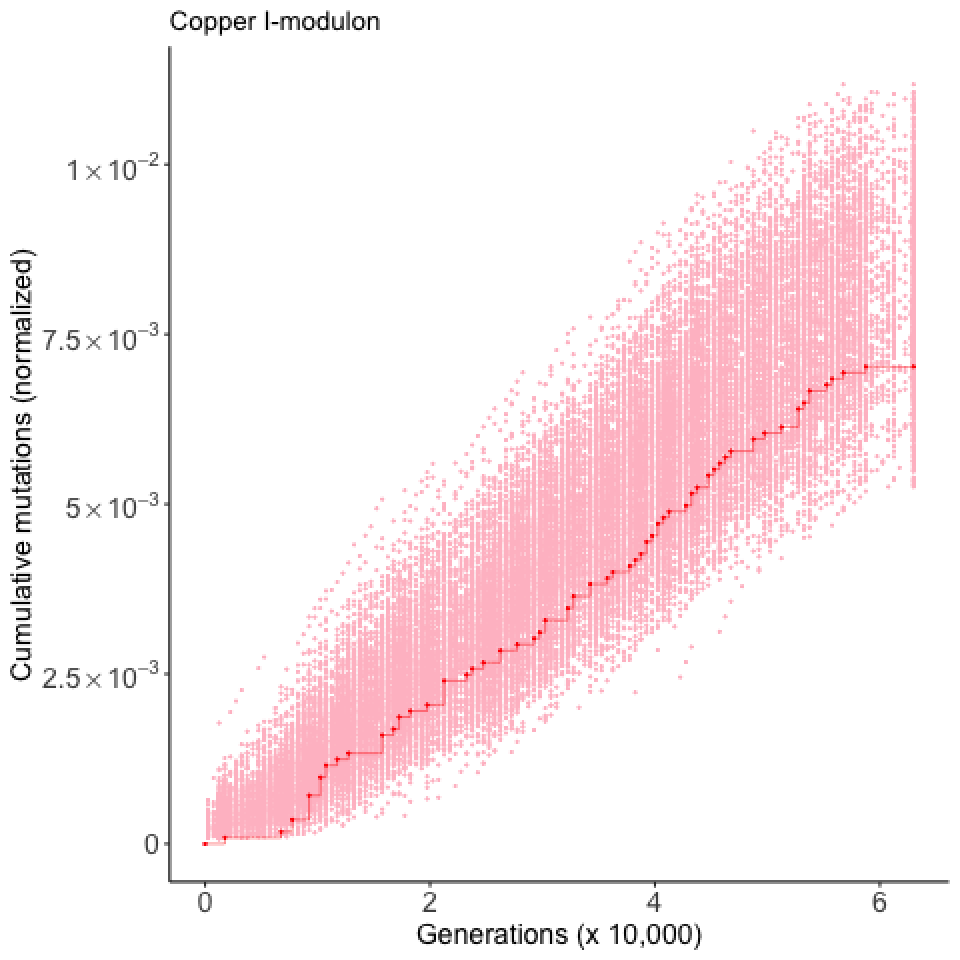

Copper I-modulon
Cumulative mutations (normalized)
1×10^{-2}
7.5×10^{-3}
5×10^{-3}
2.5×10^{-3}
0
0
2
4
6
Generations (x 10,000)

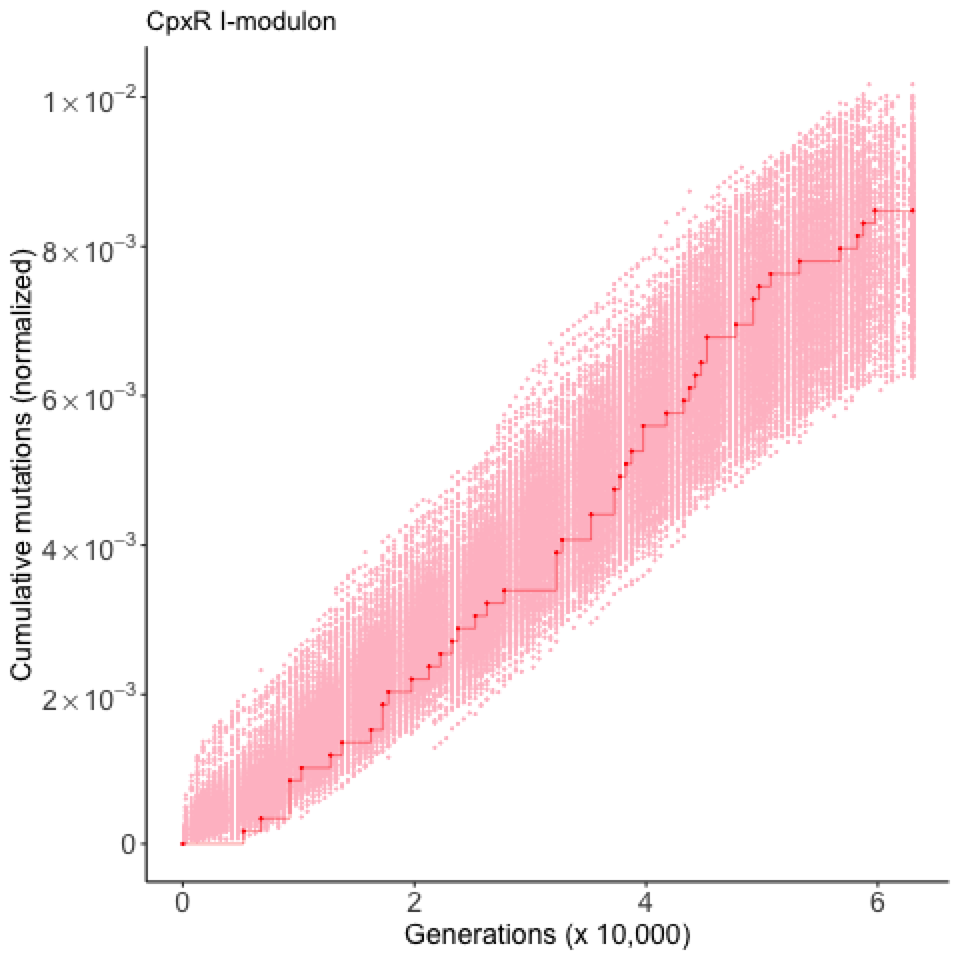
CpxR I-modulon
Cumulative mutations (normalized)
1×10^{-2}
8×10^{-3}
6×10^{-3}
4×10^{-3}
2×10^{-3}
0
0
2
4
6
Generations (x 10,000)

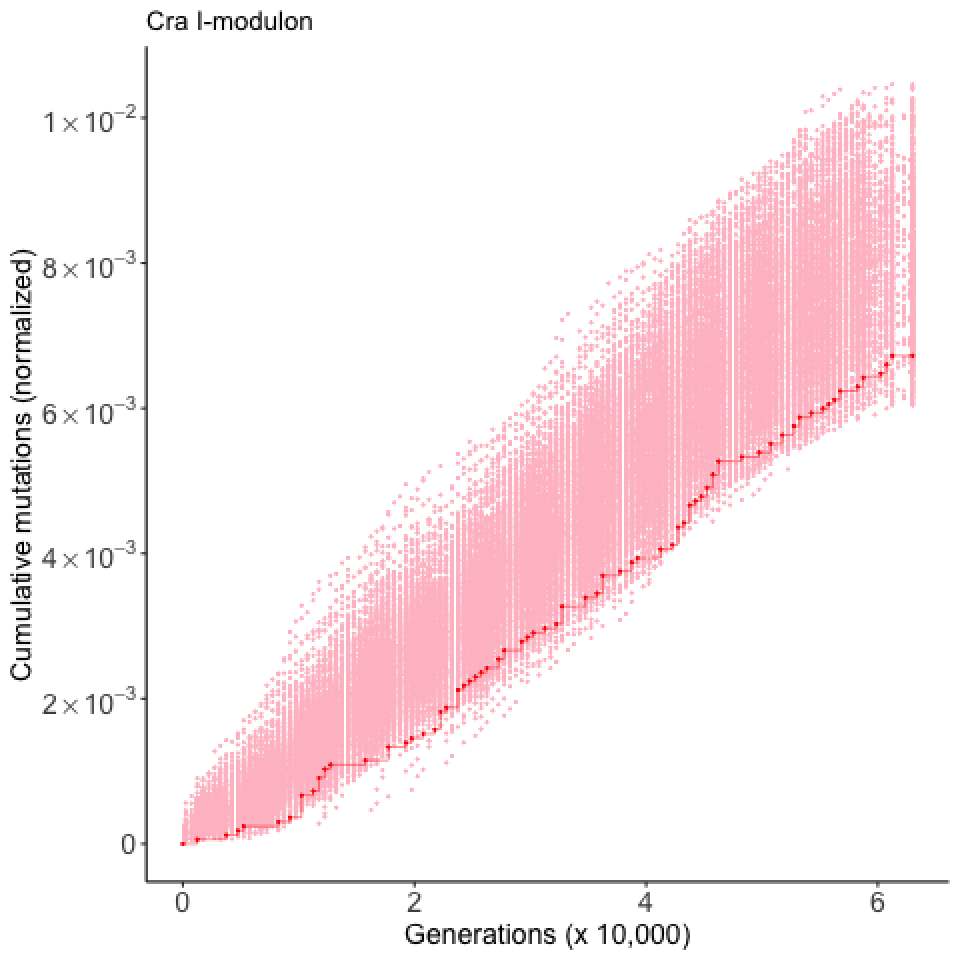

Cra I-modulon
Cumulative mutations (normalized)
1×10^-2
8×10^-3
6×10^-3
4×10^-3
2×10^-3
0
0
2
4
6
Generations (x 10,000)

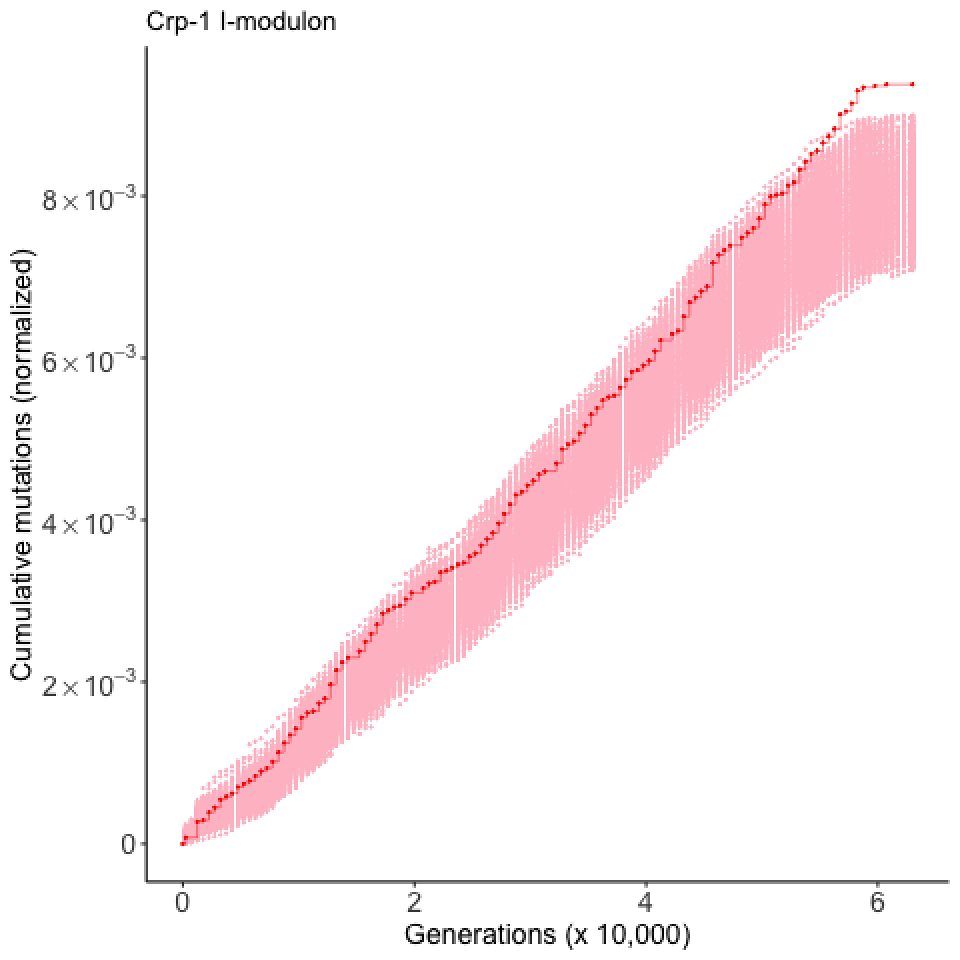

Crp-1 I-modulon
Cumulative mutations (normalized)
8×10^{-3}
6×10^{-3}
4×10^{-3}
2×10^{-3}
0
0
2
4
6
Generations (x 10,000)

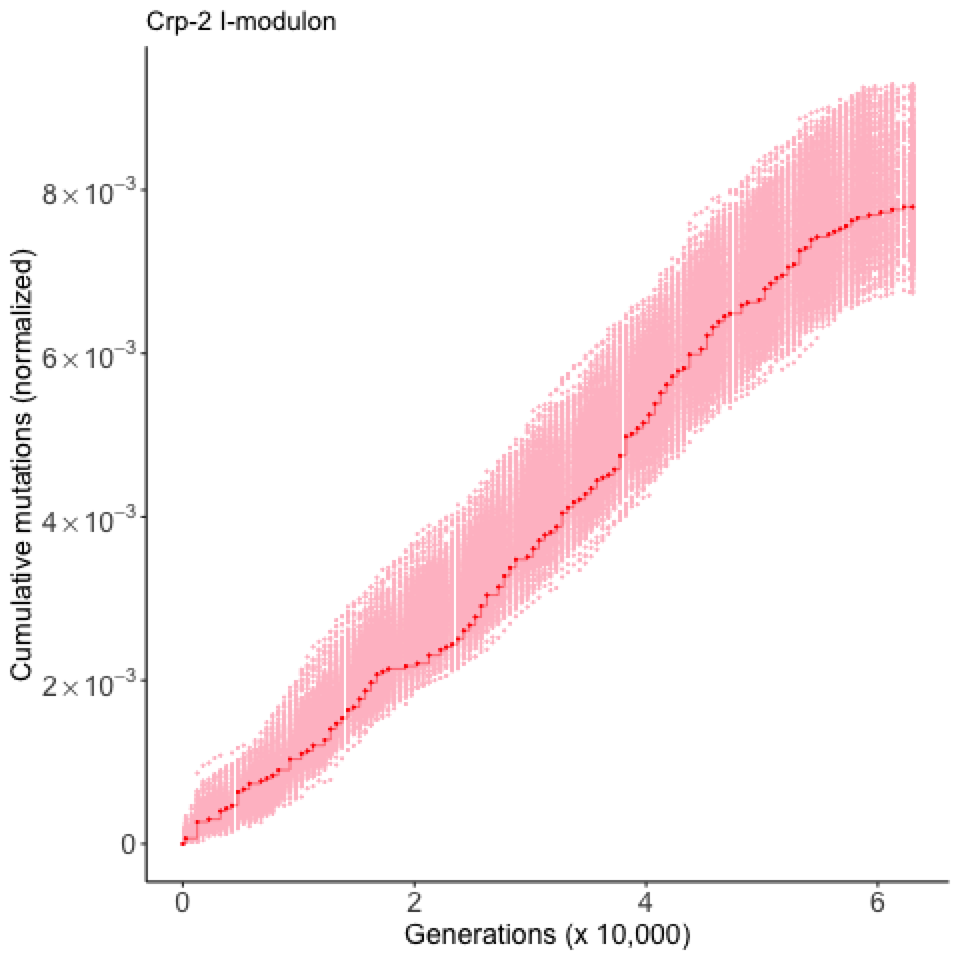

Crp-2 I-modulon
Cumulative mutations (normalized)
8×10^{-3}
6×10^{-3}
4×10^{-3}
2×10^{-3}
0
0
2
4
6
Generations (x 10,000)

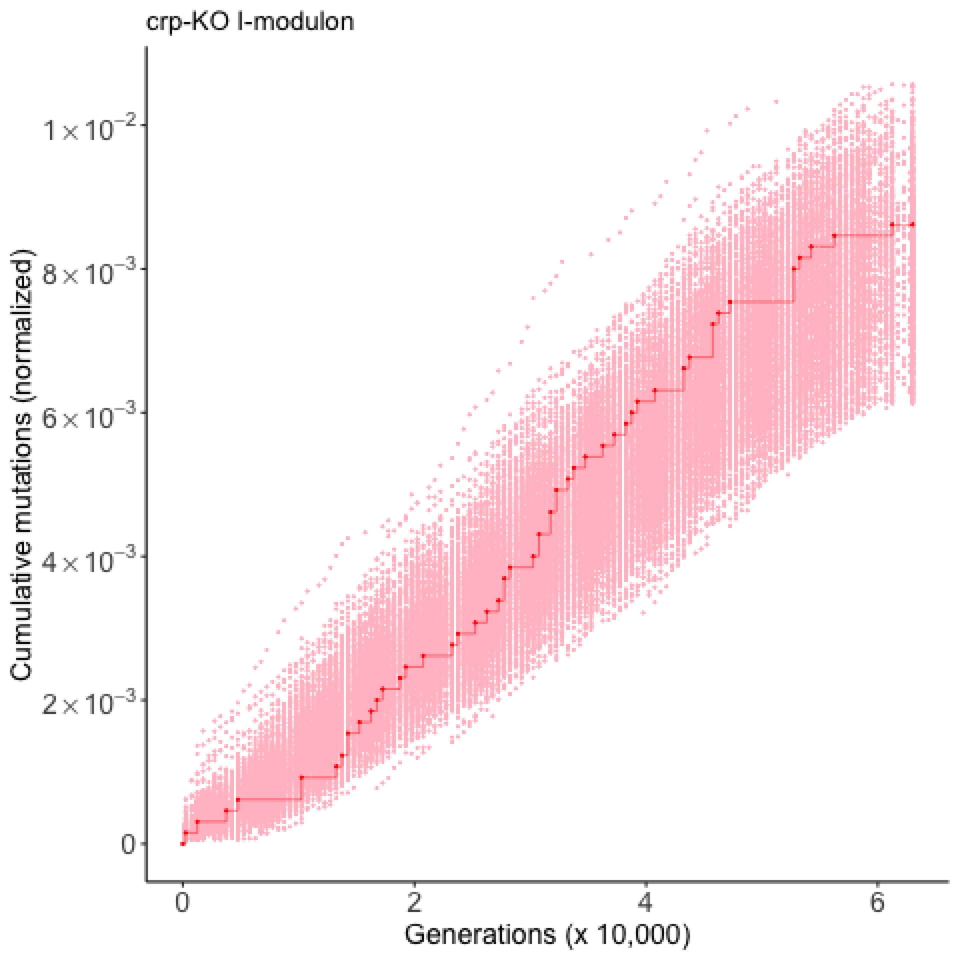
crp-KO I-modulon
Cumulative mutations (normalized)
1×10^{-2}
8×10^{-3}
6×10^{-3}
4×10^{-3}
2×10^{-3}
0
0
2
4
6
Generations (x 10,000)

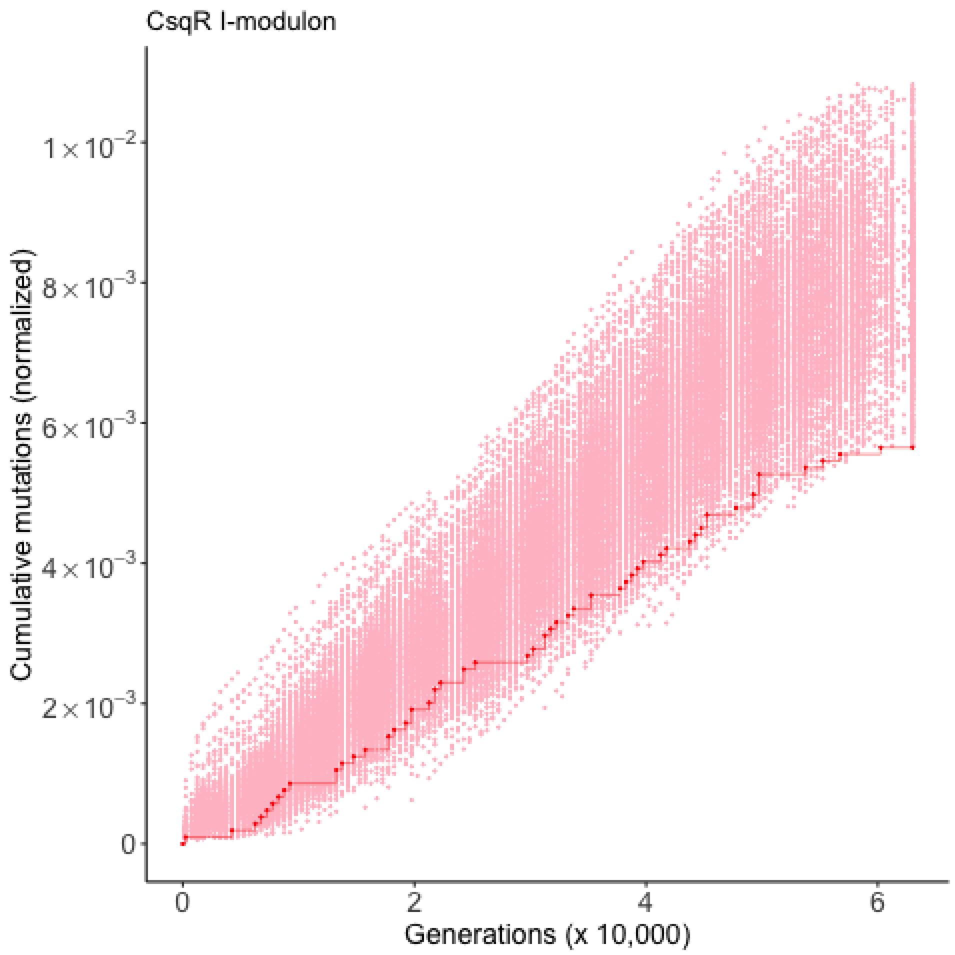

CsqR I-modulon
Cumulative mutations (normalized)
1×10^-2
8×10^-3
6×10^-3
4×10^-3
2×10^-3
0
0
2
4
6
Generations (x 10,000)

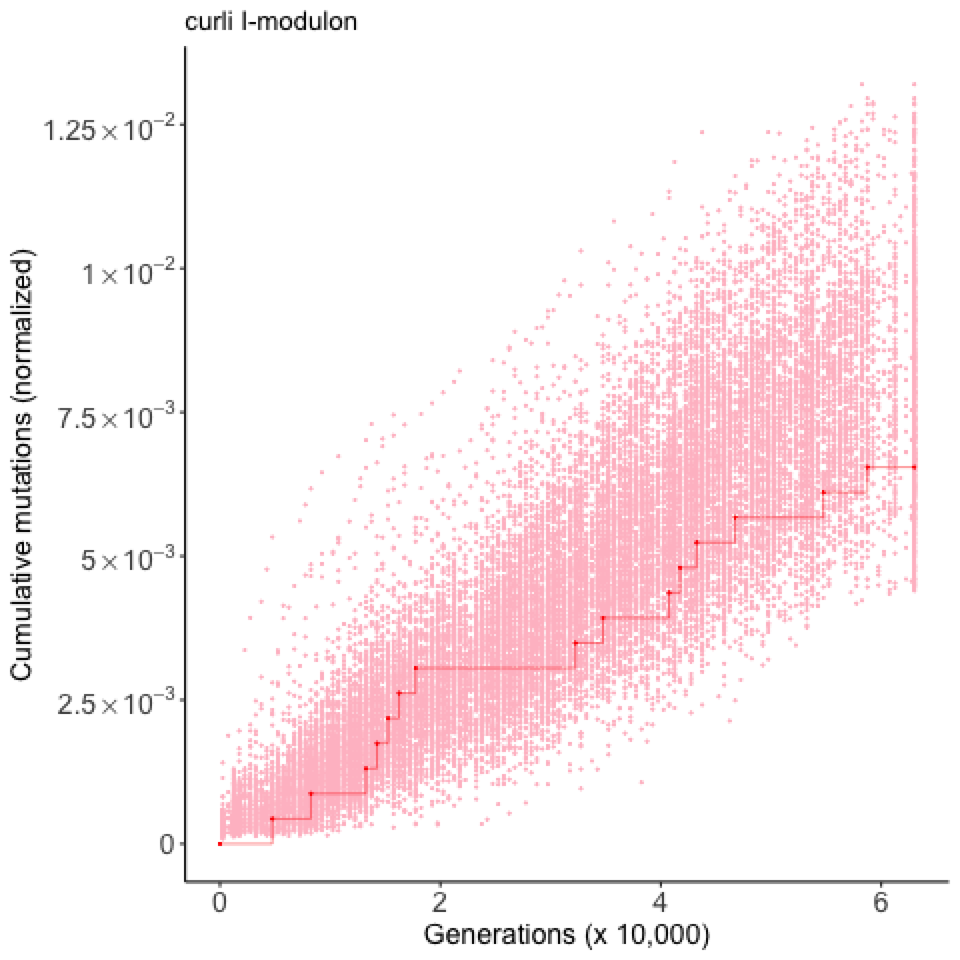

curli I-modulon
Cumulative mutations (normalized)
1.25×10⁻²
1×10⁻²
7.5×10⁻³
5×10⁻³
2.5×10⁻³
0
0
2
4
6
Generations (x 10,000)

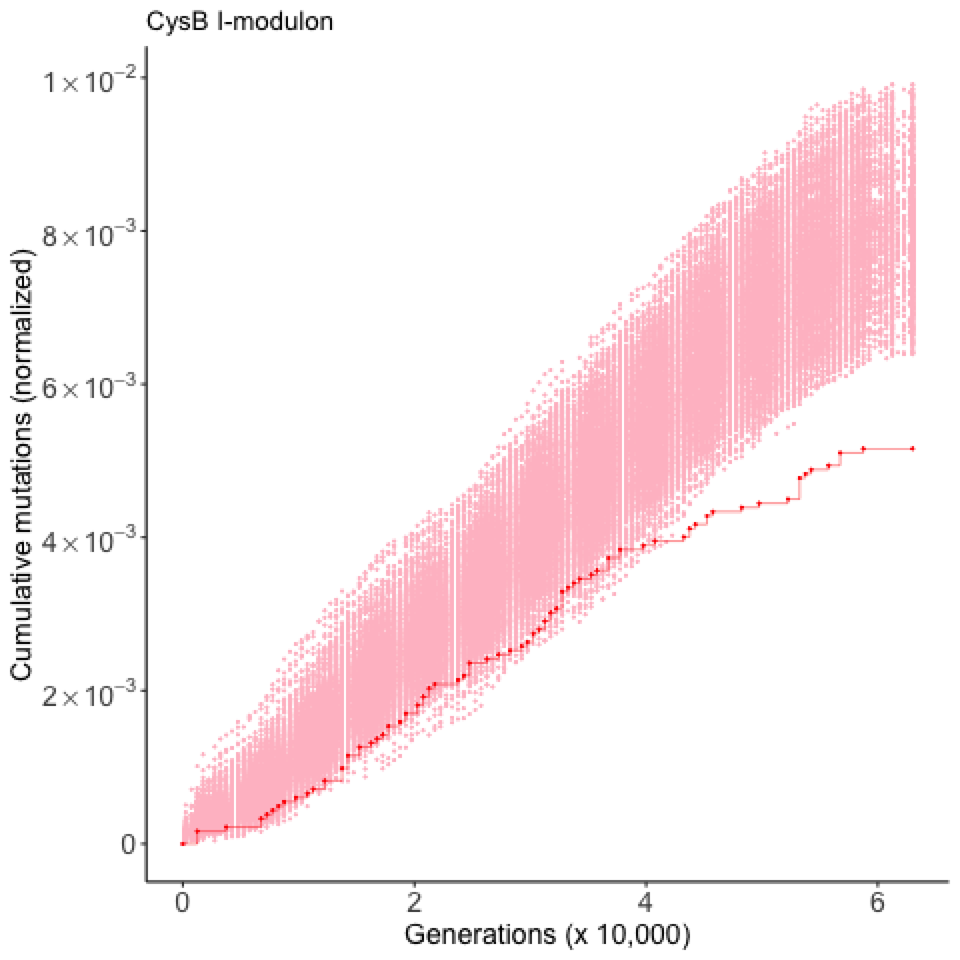

CysB I-modulon
Cumulative mutations (normalized)
1×10⁻²
8×10⁻³
6×10⁻³
4×10⁻³
2×10⁻³
0
0
2
4
6
Generations (x 10,000)

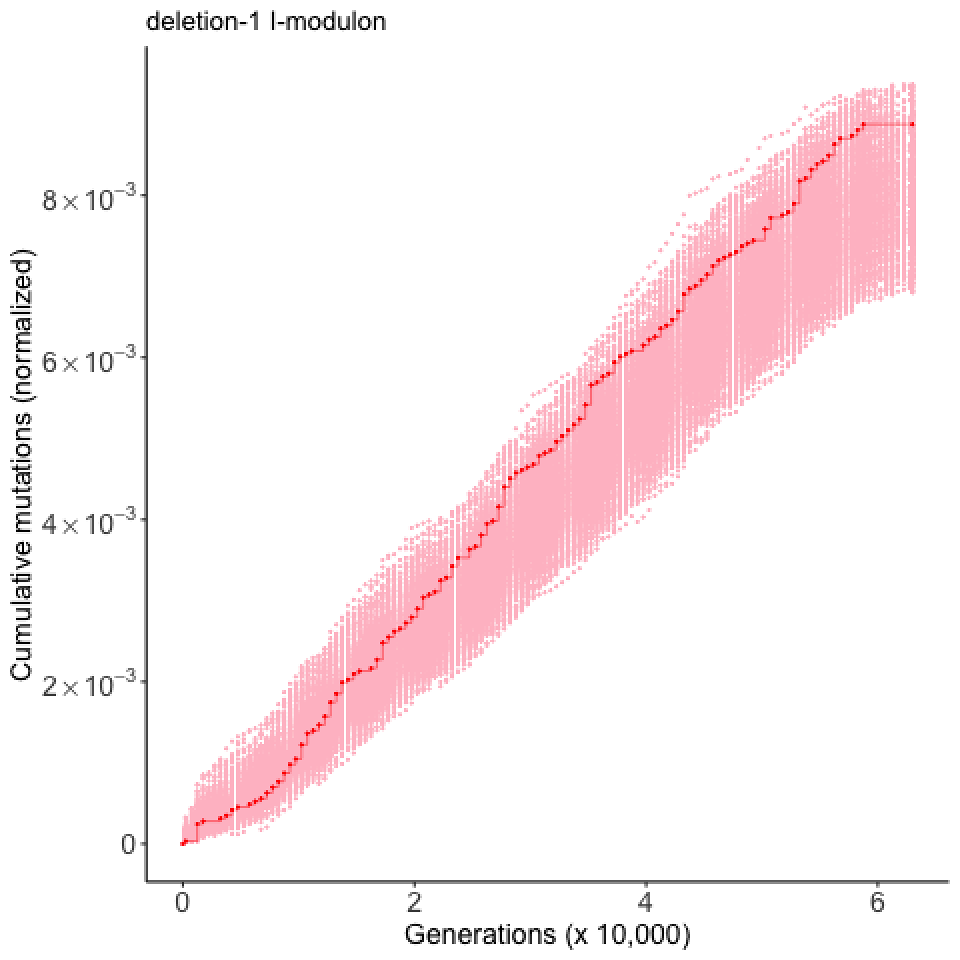

deletion-1 I-modulon
Cumulative mutations (normalized)
8×10^{-3}
6×10^{-3}
4×10^{-3}
2×10^{-3}
0
0
2
4
6
Generations (x 10,000)

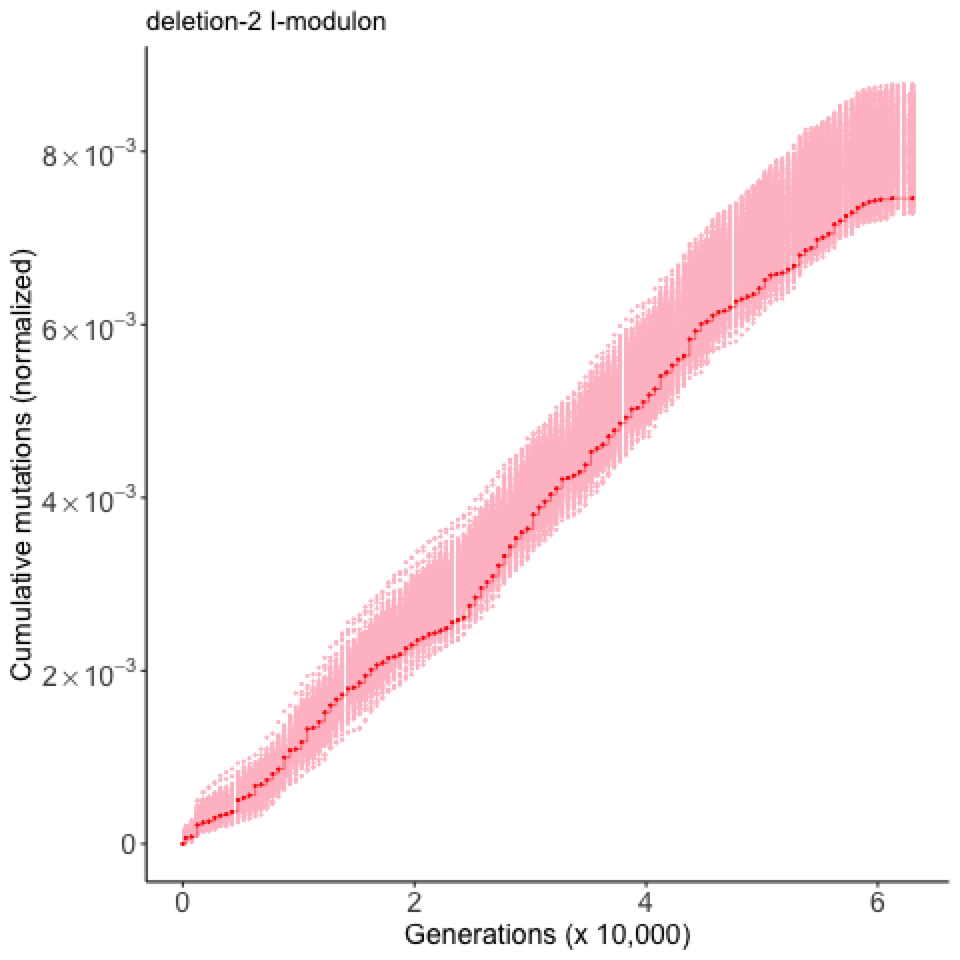

deletion-2 I-modulon
Cumulative mutations (normalized)
8×10⁻³
6×10⁻³
4×10⁻³
2×10⁻³
0
0
2
4
6
Generations (x 10,000)

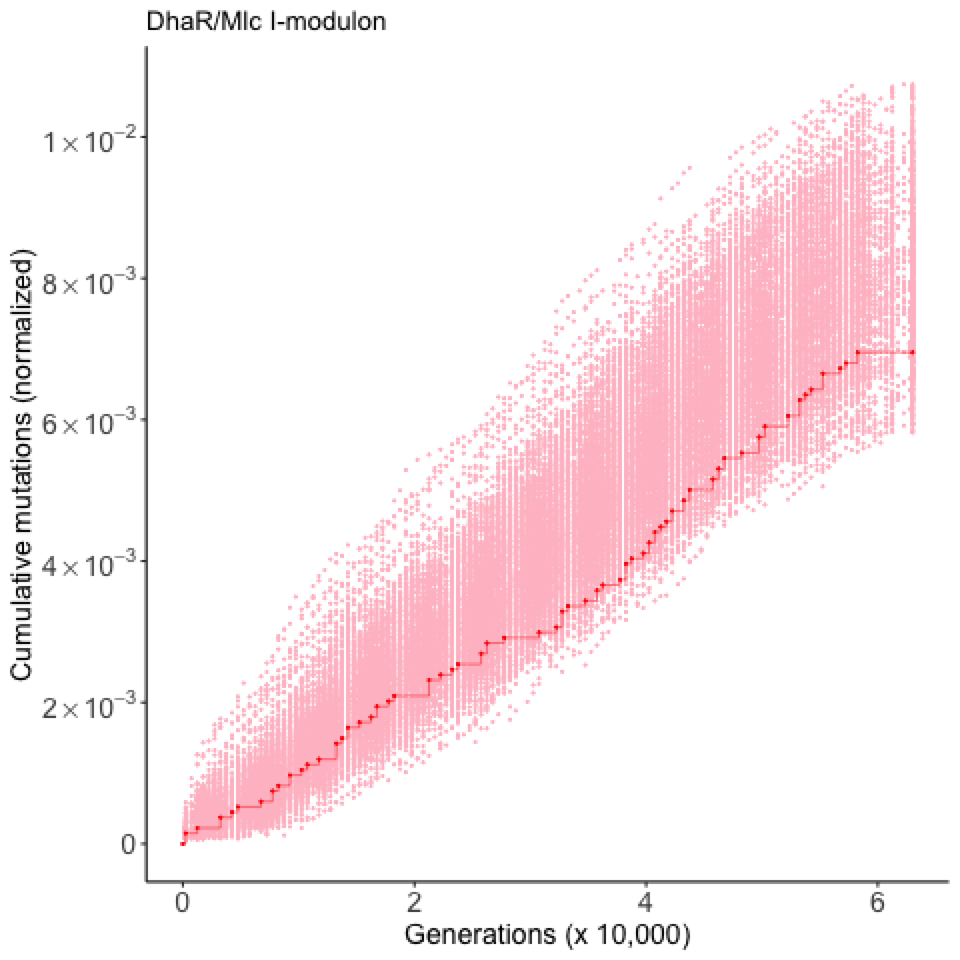

DhaR/Mlc I-modulon
Cumulative mutations (normalized)
1×10^{-2}
8×10^{-3}
6×10^{-3}
4×10^{-3}
2×10^{-3}
0
0
2
4
6
Generations (x 10,000)

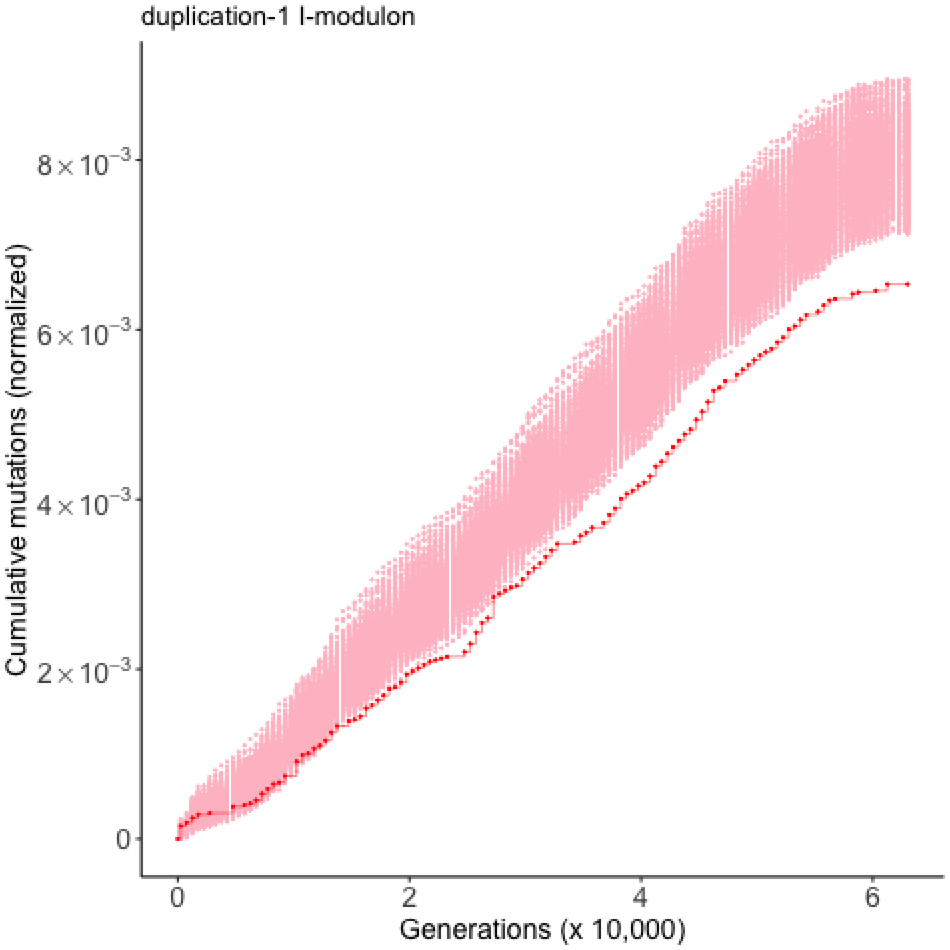

duplication-1 l-modulon
Cumulative mutations (normalized)
8×10^{-3}
6×10^{-3}
4×10^{-3}
2×10^{-3}
0
0
2
4
6
Generations (x 10,000)

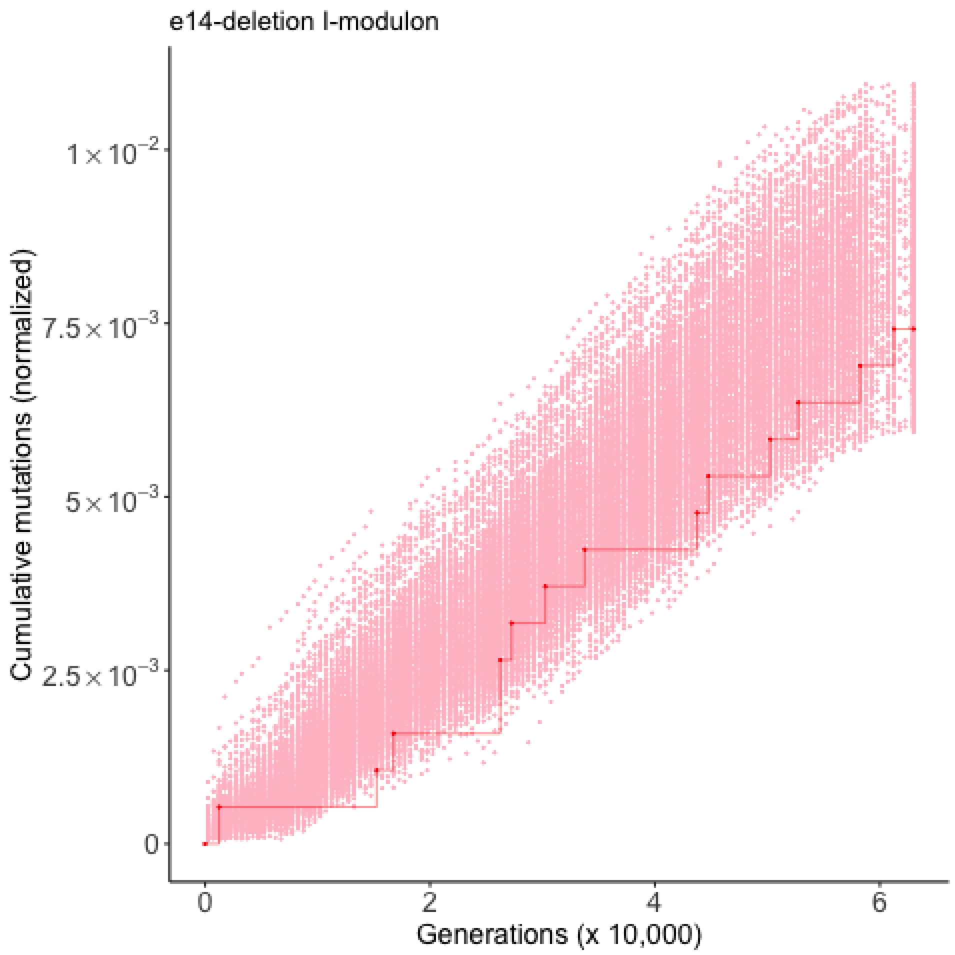

e14-deletion I-modulon
Cumulative mutations (normalized)
1×10^{-2}
7.5×10^{-3}
5×10^{-3}
2.5×10^{-3}
0
0
2
4
6
Generations (x 10,000)

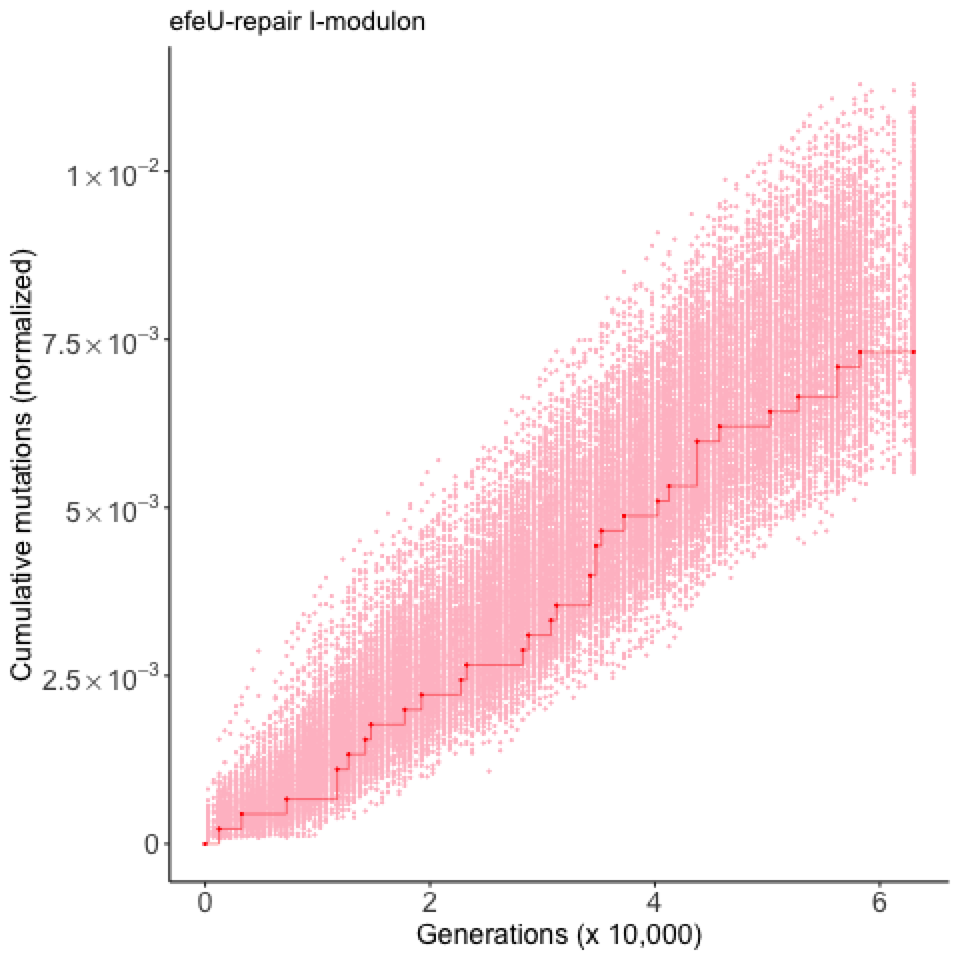

efeU-repair I-modulon
Cumulative mutations (normalized)
1×10^{-2}
7.5×10^{-3}
5×10^{-3}
2.5×10^{-3}
0
0
2
4
6
Generations (x 10,000)

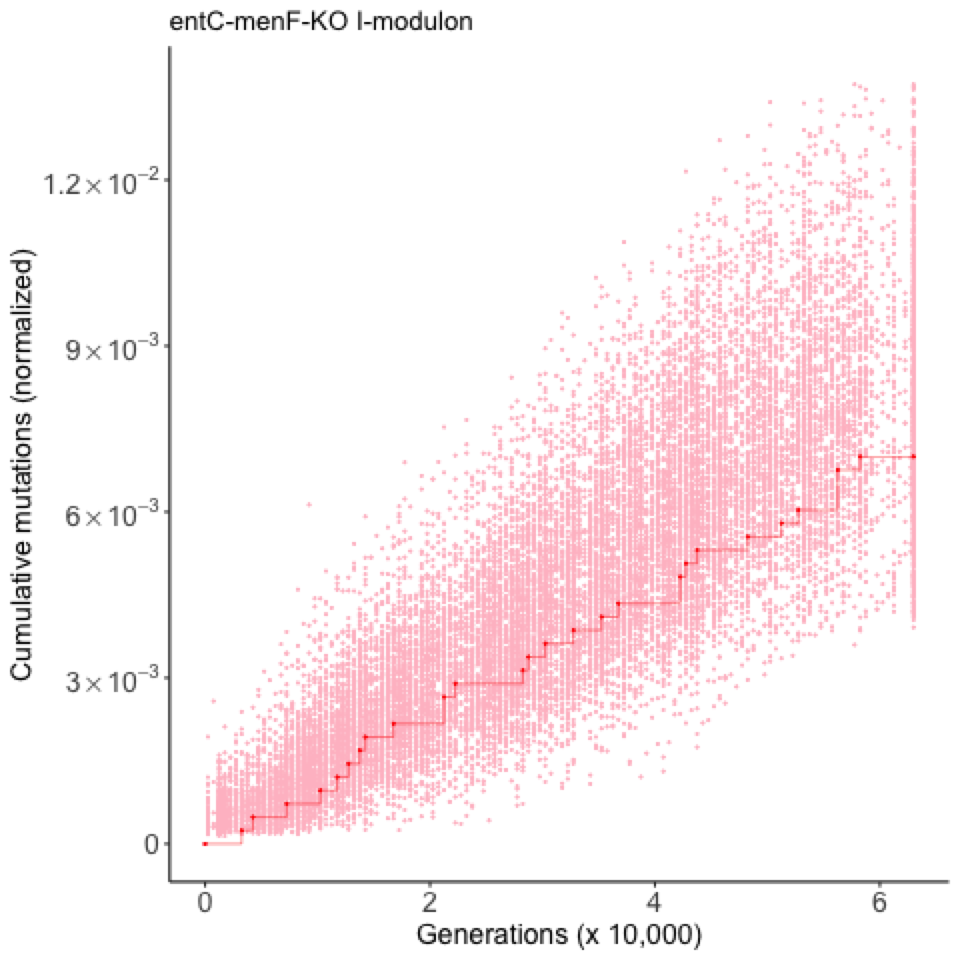

entC-menF-KO I-modulon
Cumulative mutations (normalized)
1.2×10^{-2}
9×10^{-3}
6×10^{-3}
3×10^{-3}
0
0
2
4
6
Generations (x 10,000)

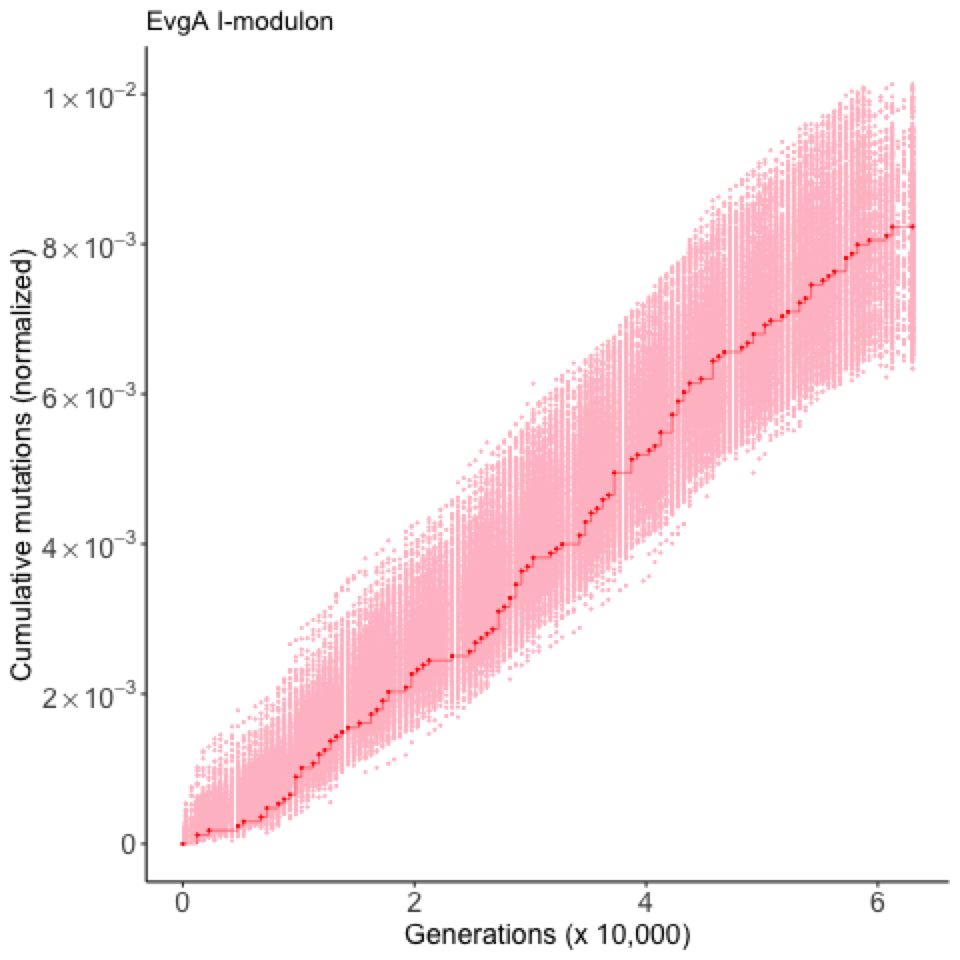
EvgA I-modulon
Cumulative mutations (normalized)
1×10^{-2}
8×10^{-3}
6×10^{-3}
4×10^{-3}
2×10^{-3}
0
0
2
4
6
Generations (x 10,000)

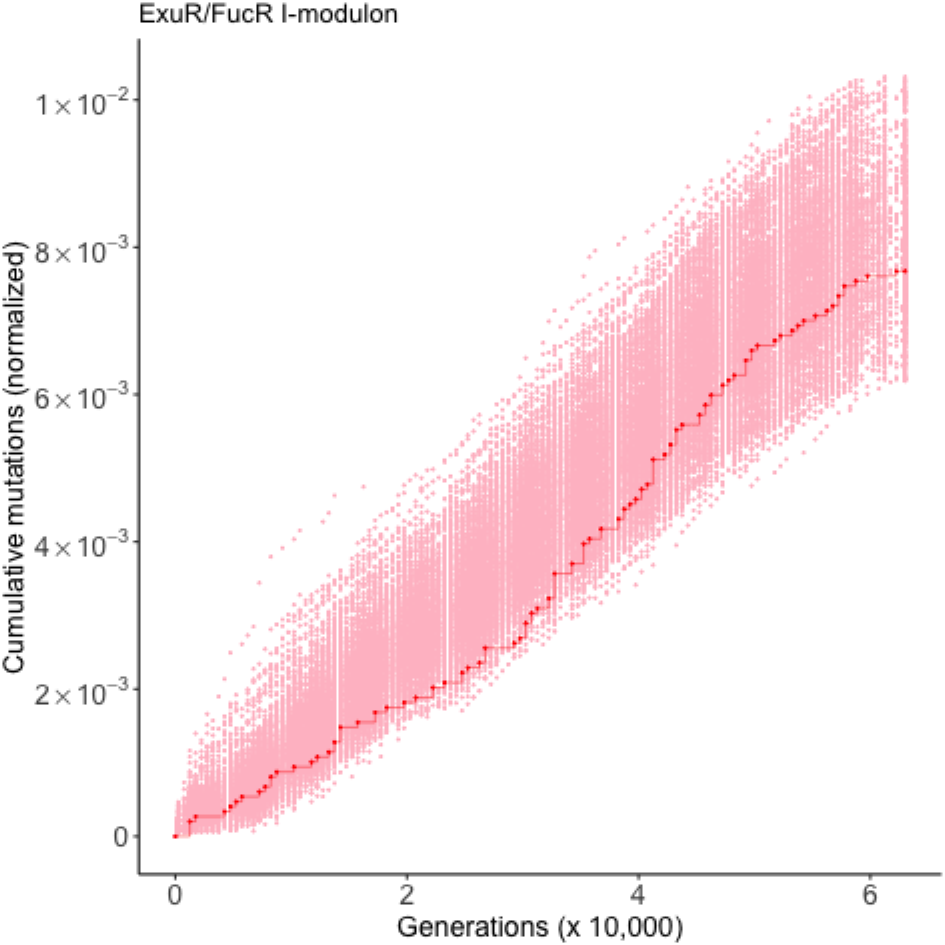
ExuR/FucR I-modulon
Cumulative mutations (normalized)
1×10⁻²
8×10⁻³
6×10⁻³
4×10⁻³
2×10⁻³
0
0
2
4
6
Generations (x 10,000)

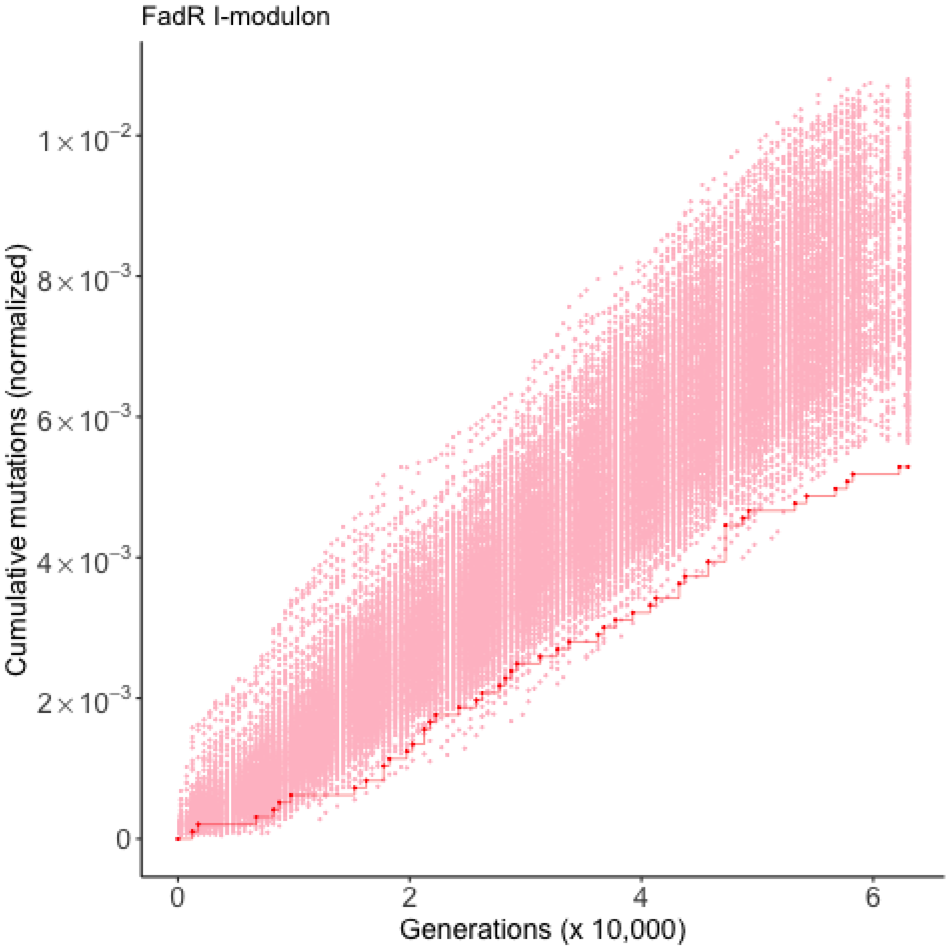

FadR I-modulon
Cumulative mutations (normalized)
1×10^{-2}
8×10^{-3}
6×10^{-3}
4×10^{-3}
2×10^{-3}
0
0
2
4
6
Generations (x 10,000)

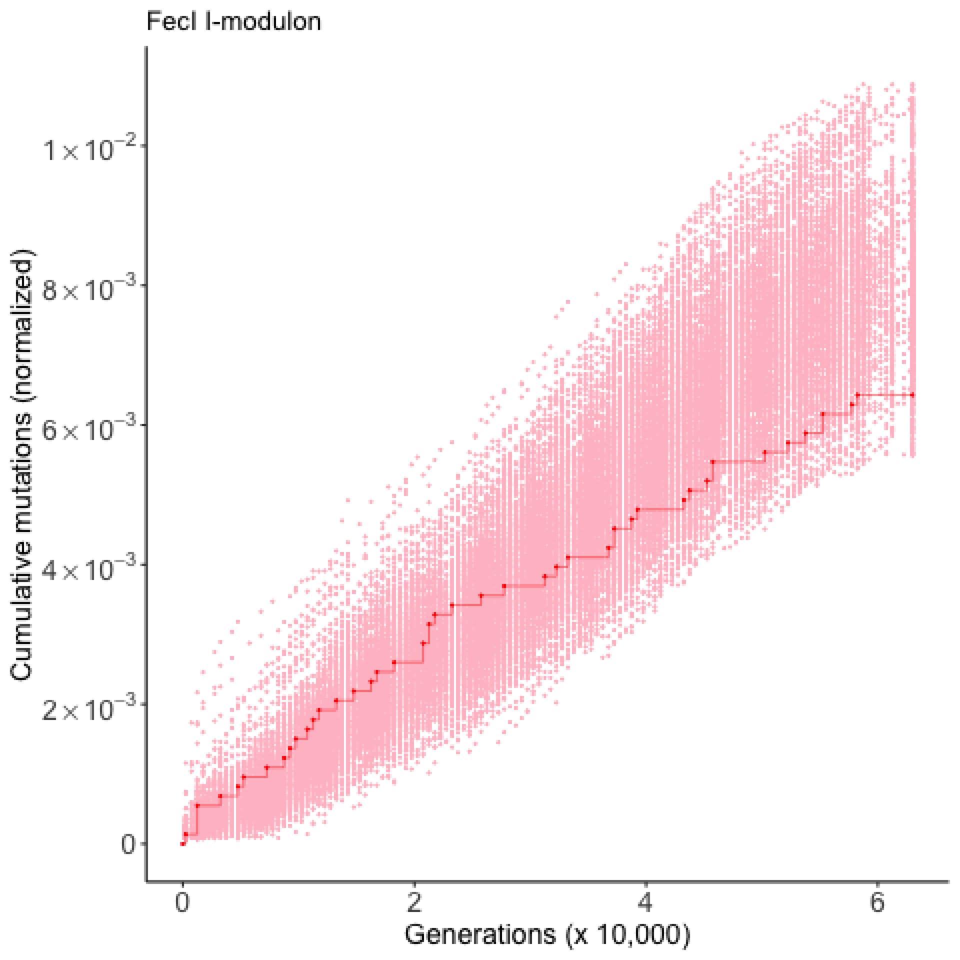
FecI I-modulon
Cumulative mutations (normalized)
1×10^−2
8×10^−3
6×10^−3
4×10^−3
2×10^−3
0
0
2
4
6
Generations (x 10,000)

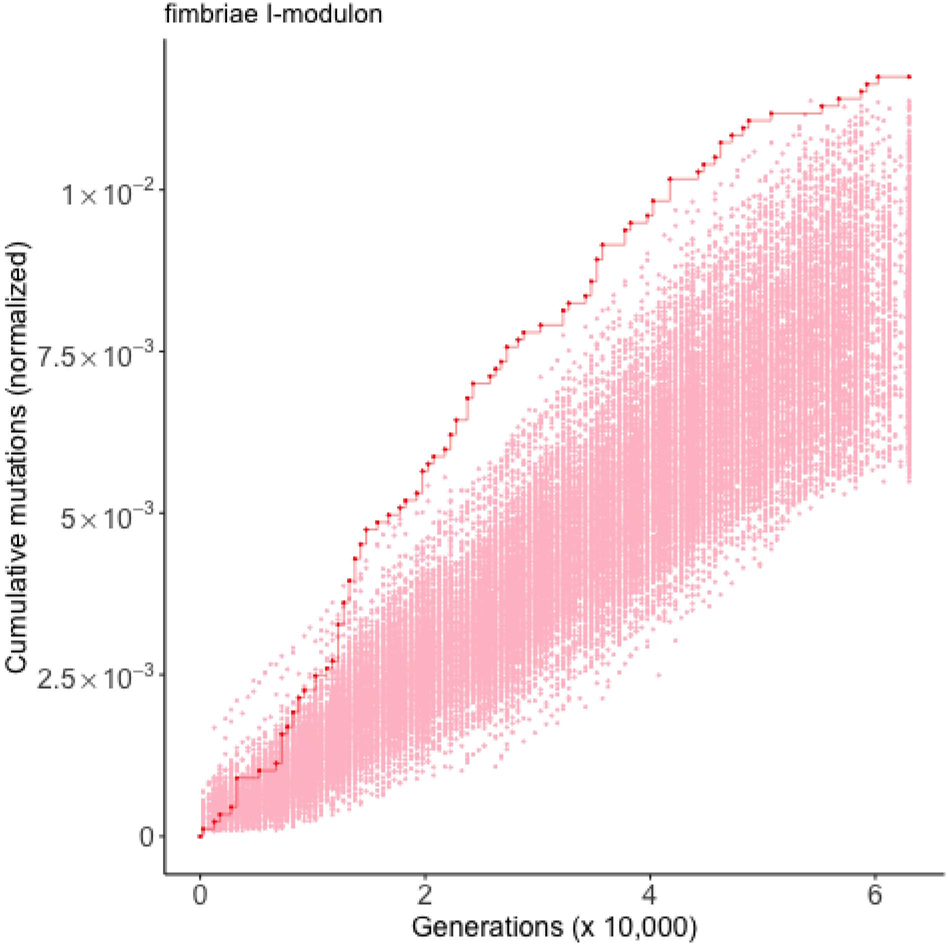

fimbriae I-modulon
Cumulative mutations (normalized)
1×10^{-2}
7.5×10^{-3}
5×10^{-3}
2.5×10^{-3}
0
0
2
4
6
Generations (x 10,000)

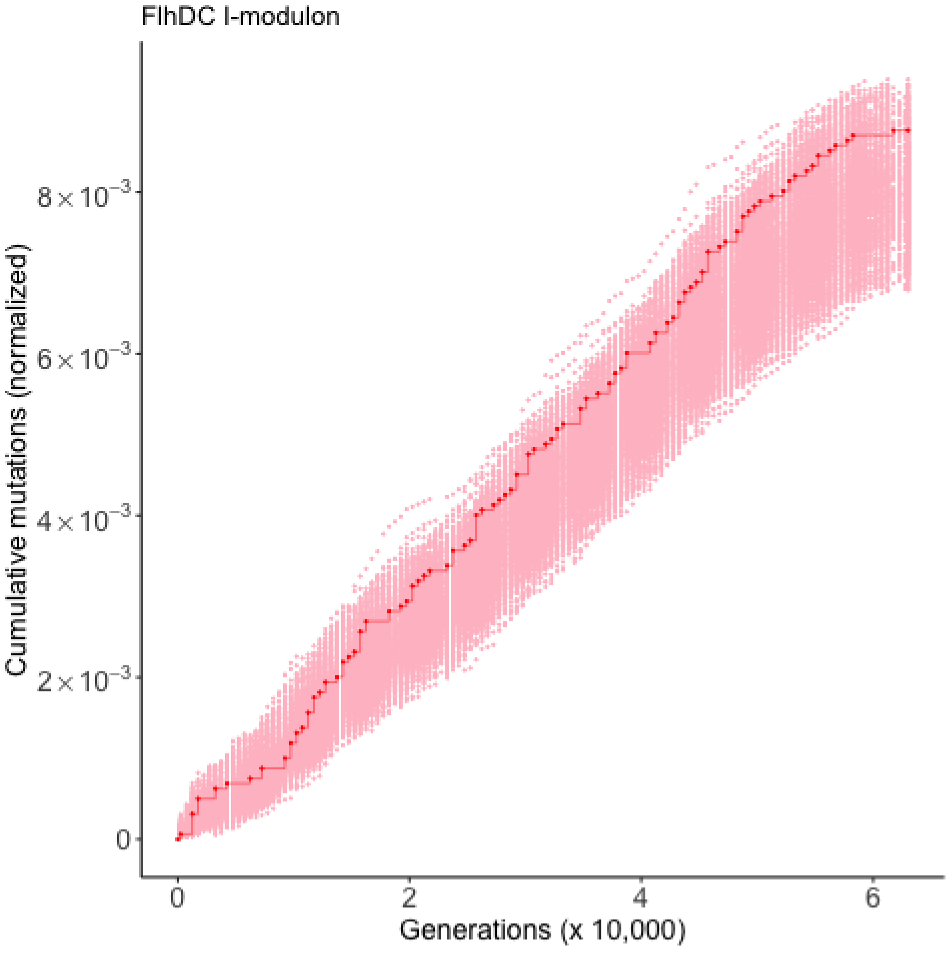

FlhDC I-modulon
Cumulative mutations (normalized)
8×10^{-3}
6×10^{-3}
4×10^{-3}
2×10^{-3}
0
0
2
4
6
Generations (x 10,000)

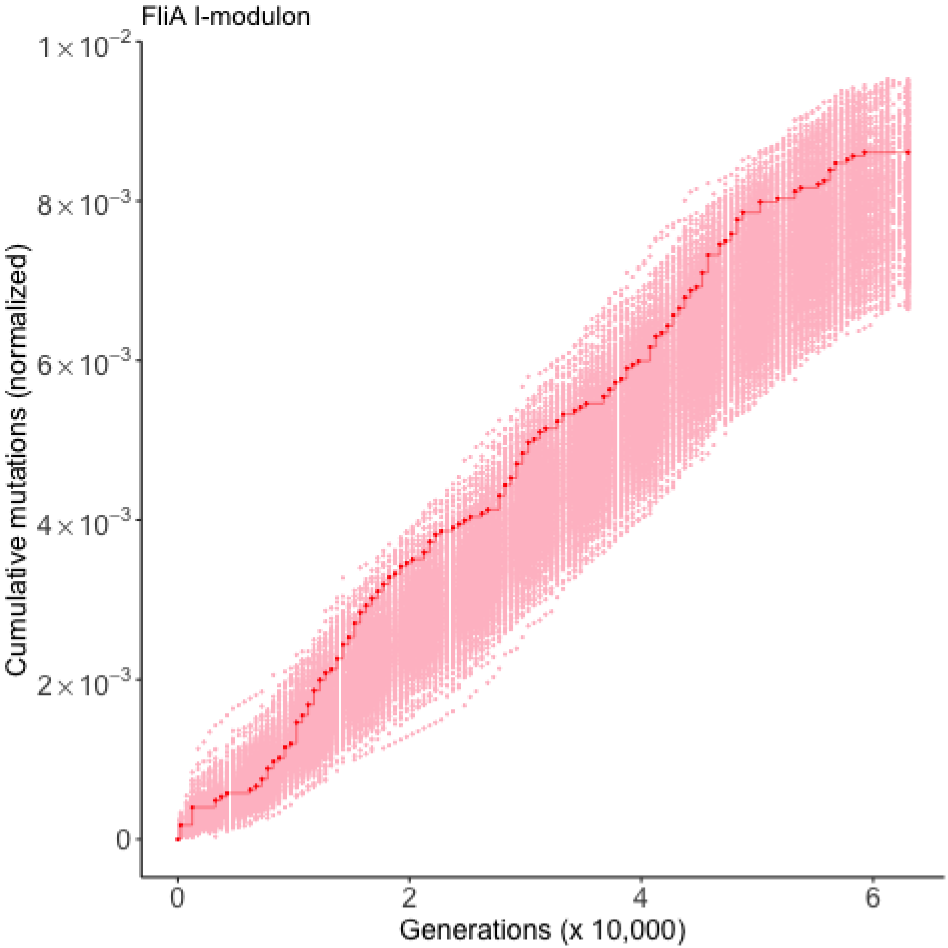

FliA I-modulon
1×10^{-2}
8×10^{-3}
6×10^{-3}
4×10^{-3}
2×10^{-3}
0
Cumulative mutations (normalized)
0
2
4
6
Generations (x 10,000)

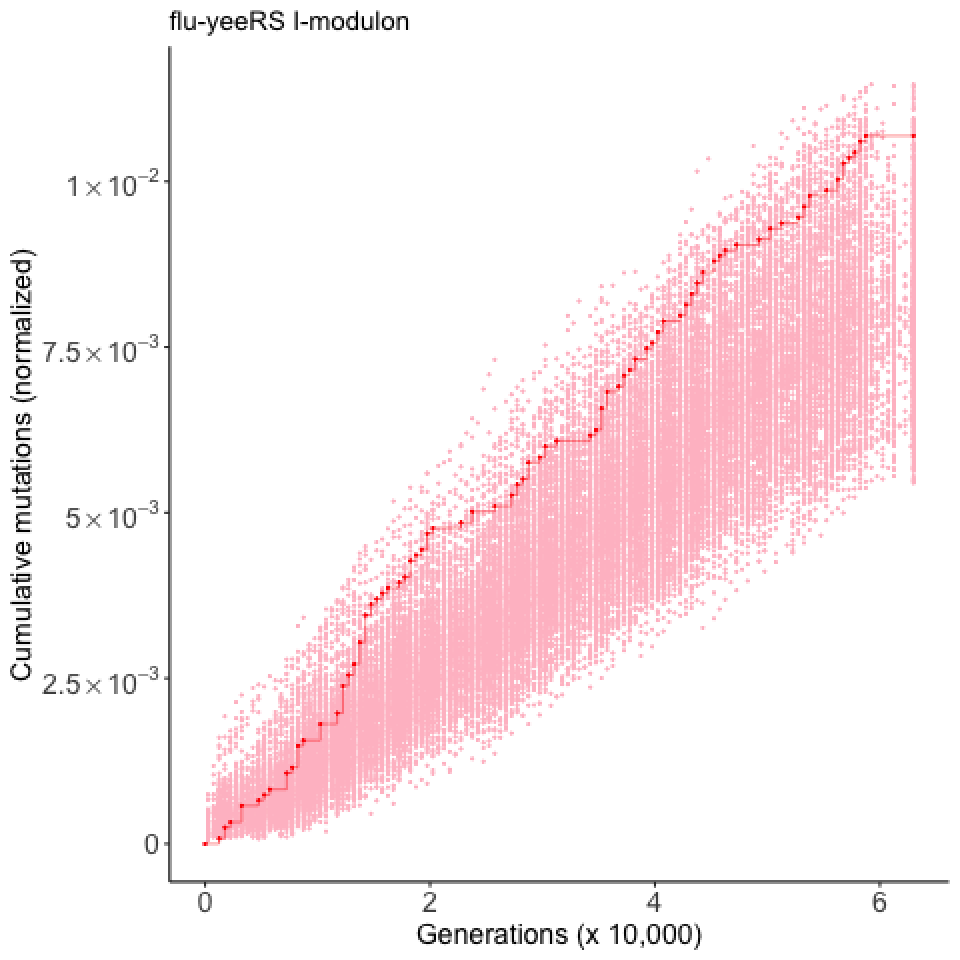

flu-yeeRS I-modulon
Cumulative mutations (normalized)
1×10^{-2}
7.5×10^{-3}
5×10^{-3}
2.5×10^{-3}
0
0
2
4
6
Generations (x 10,000)

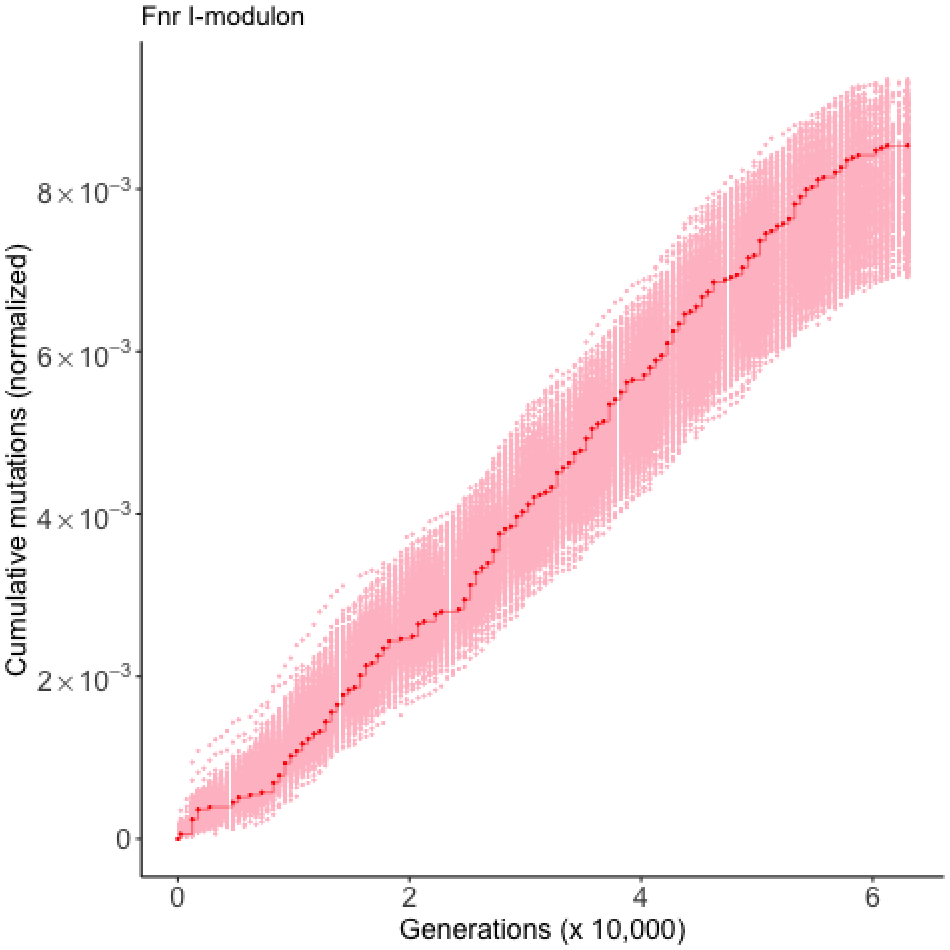

Fnr I-modulon
Cumulative mutations (normalized)
8×10^{-3}
6×10^{-3}
4×10^{-3}
2×10^{-3}
0
0
2
4
6
Generations (x 10,000)

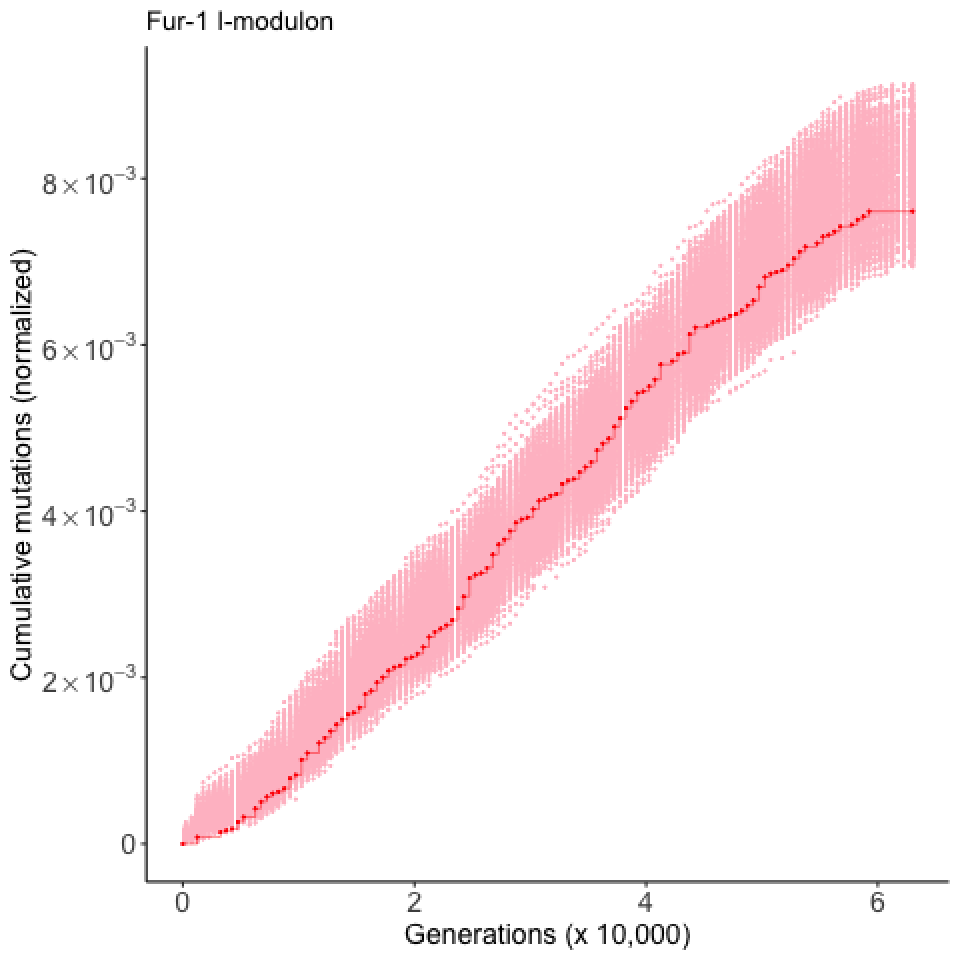

Fur-1 I-modulon
Cumulative mutations (normalized)
8×10^−3
6×10^−3
4×10^−3
2×10^−3
0
0
2
4
6
Generations (x 10,000)

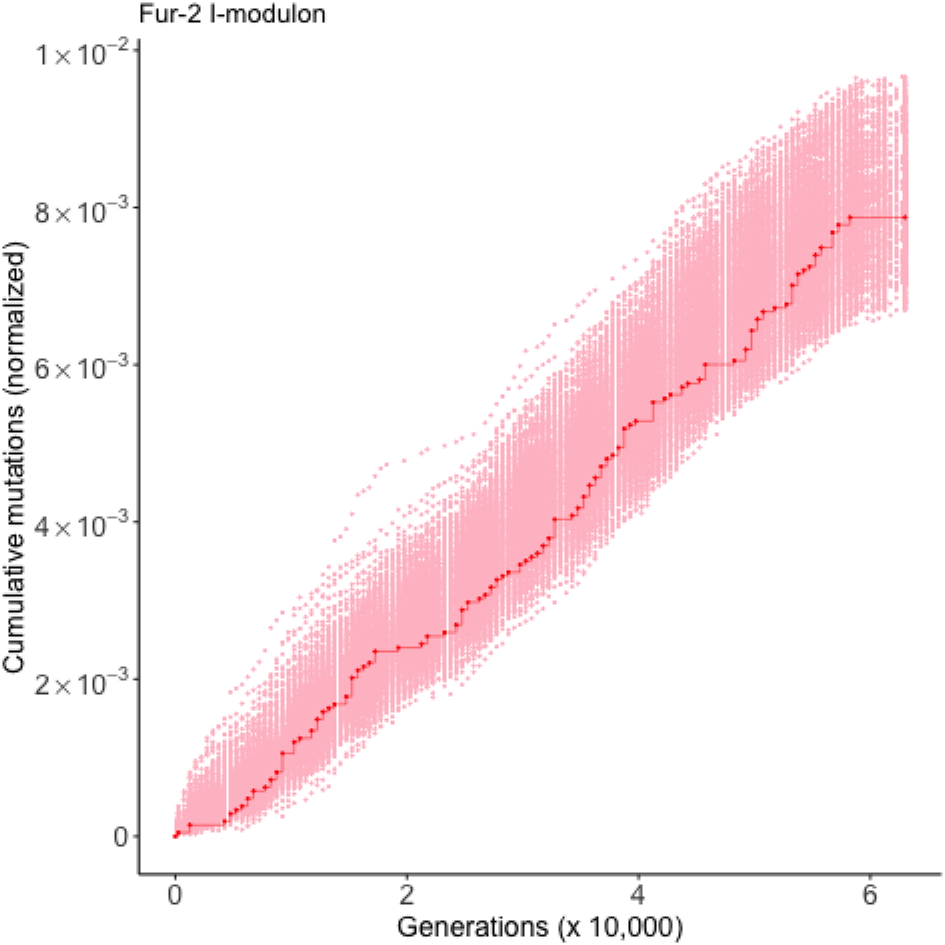

Fur-2 I-modulon
Cumulative mutations (normalized)
1×10^{-2}
8×10^{-3}
6×10^{-3}
4×10^{-3}
2×10^{-3}
0
0
2
4
6
Generations (x 10,000)

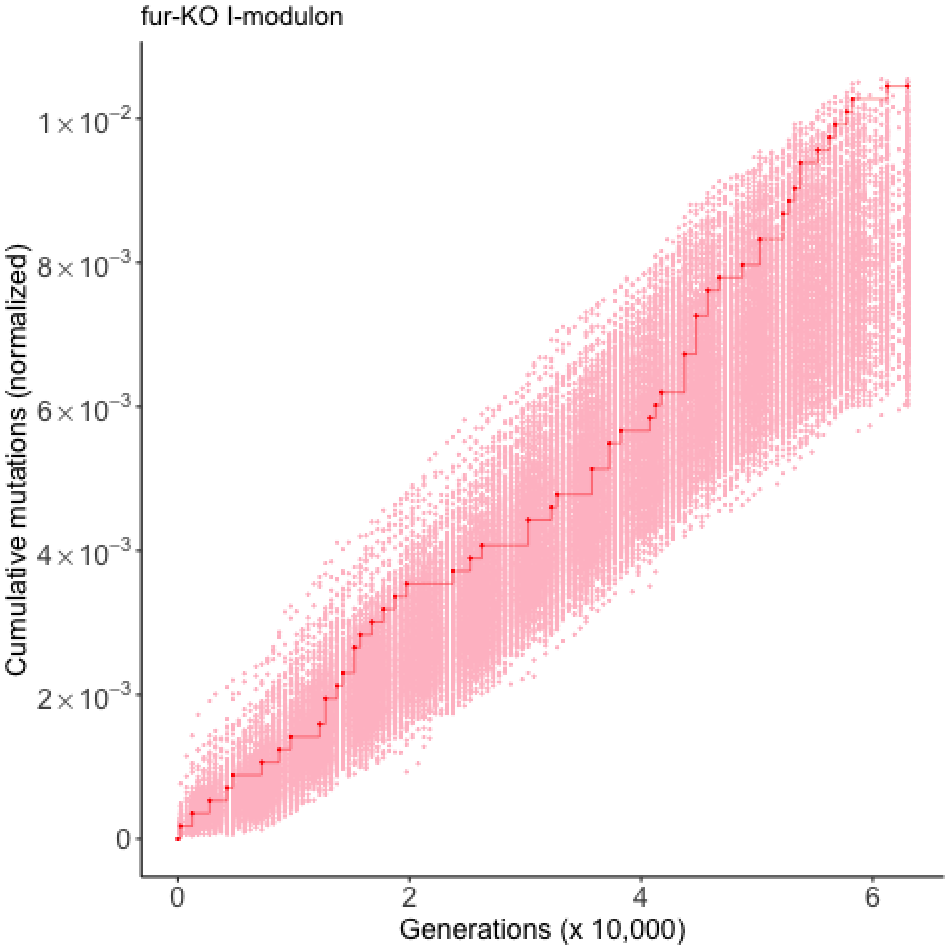

fur-KO I-modulon
Cumulative mutations (normalized)
1×10^{-2}
8×10^{-3}
6×10^{-3}
4×10^{-3}
2×10^{-3}
0
0
2
4
6
Generations (x 10,000)

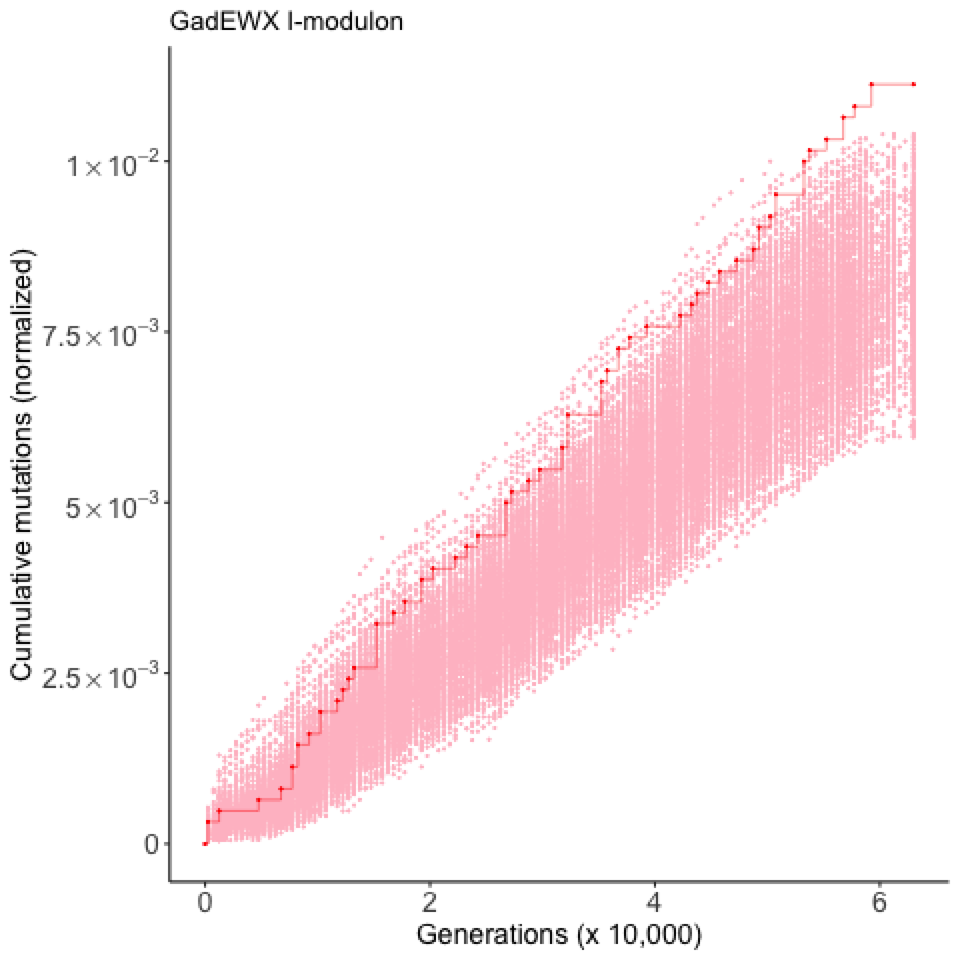

GadEWX I-modulon
Cumulative mutations (normalized)
1×10^-2
7.5×10^-3
5×10^-3
2.5×10^-3
0
0
2
4
6
Generations (x 10,000)

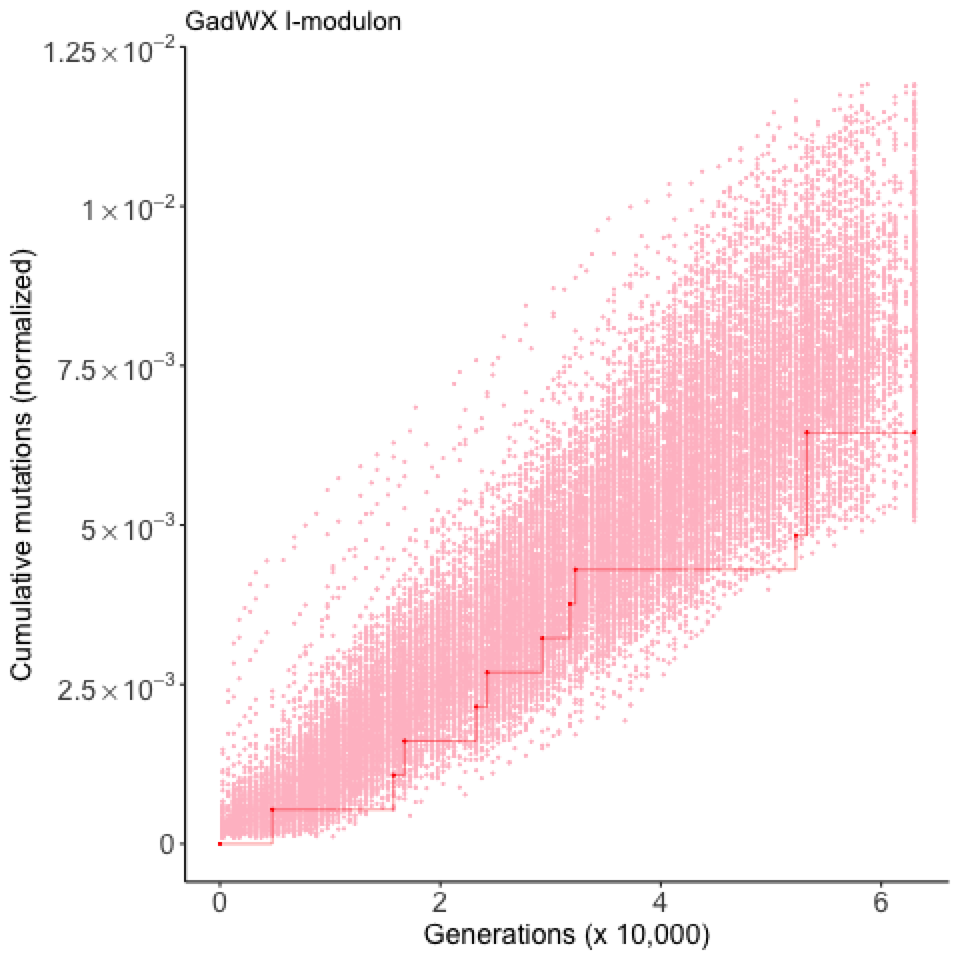

GadWX I-modulon
1.25 × 10^-2
1 × 10^-2
7.5 × 10^-3
5 × 10^-3
2.5 × 10^-3
0
Cumulative mutations (normalized)
0
2
4
6
Generations (x 10,000)

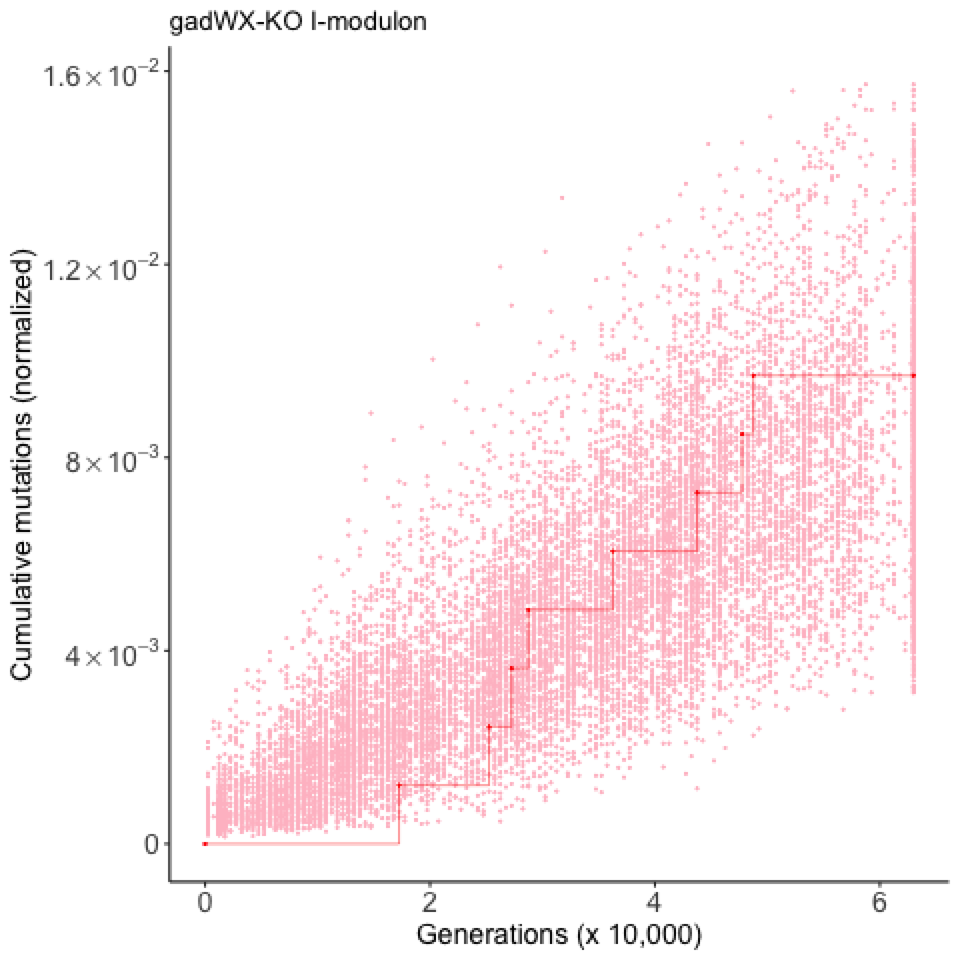
gadWX-KO I-modulon
1.6×10^{-2}
1.2×10^{-2}
8×10^{-3}
4×10^{-3}
0
Cumulative mutations (normalized)
0
2
4
6
Generations (x 10,000)

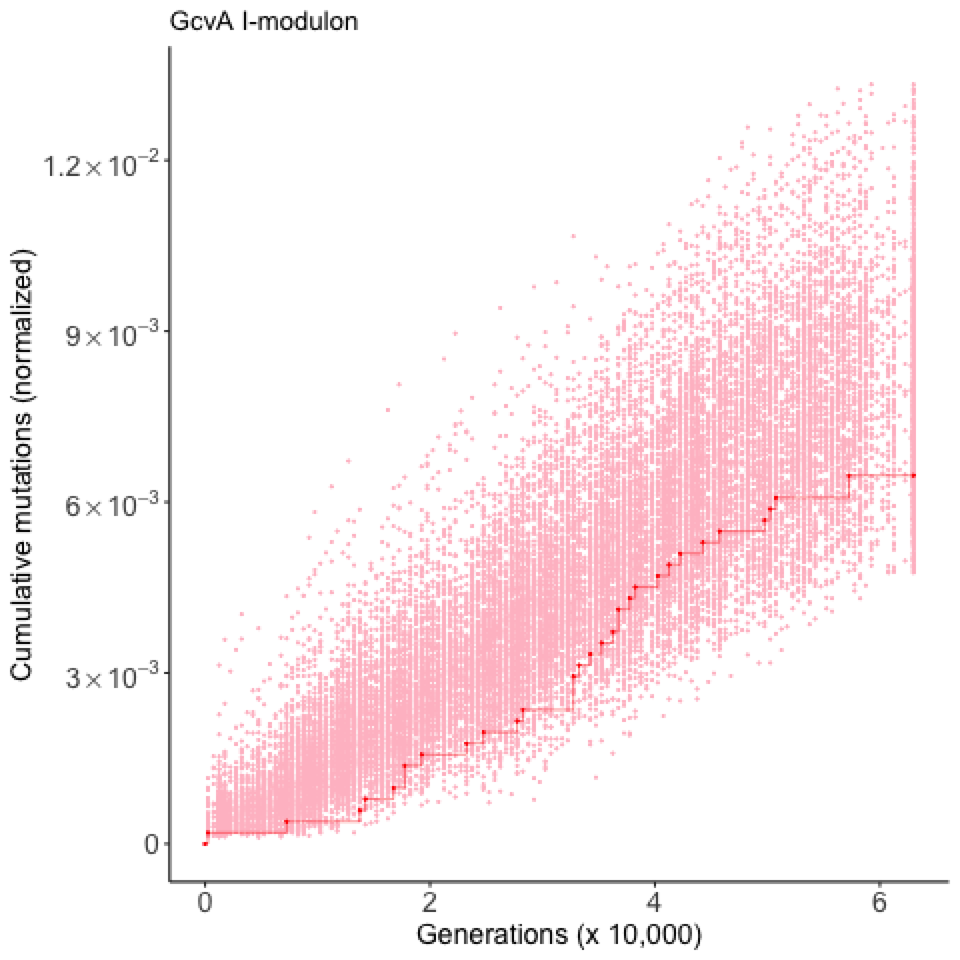
GcvA I-modulon
Cumulative mutations (normalized)
1.2×10^{-2}
9×10^{-3}
6×10^{-3}
3×10^{-3}
0
0
2
4
6
Generations (x 10,000)

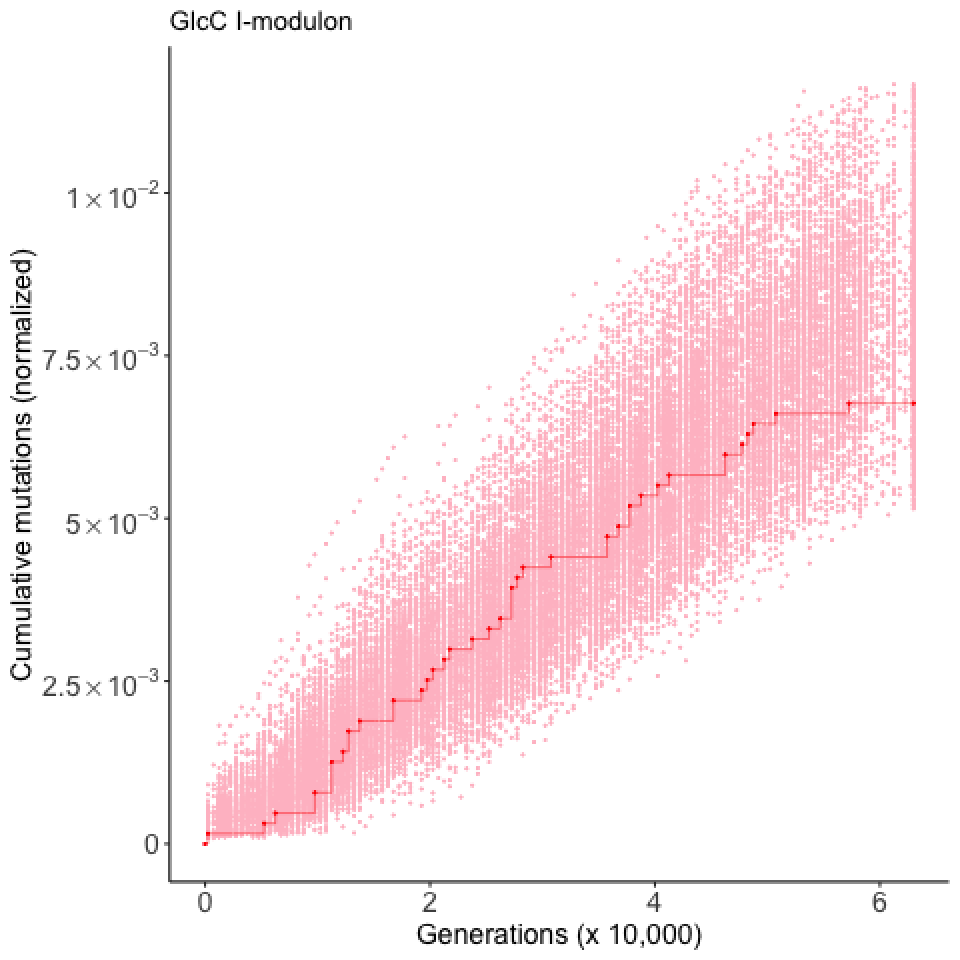

GlcC I-modulon
Cumulative mutations (normalized)
1 × 10^-2
7.5 × 10^-3
5 × 10^-3
2.5 × 10^-3
0
0
2
4
6
Generations (x 10,000)

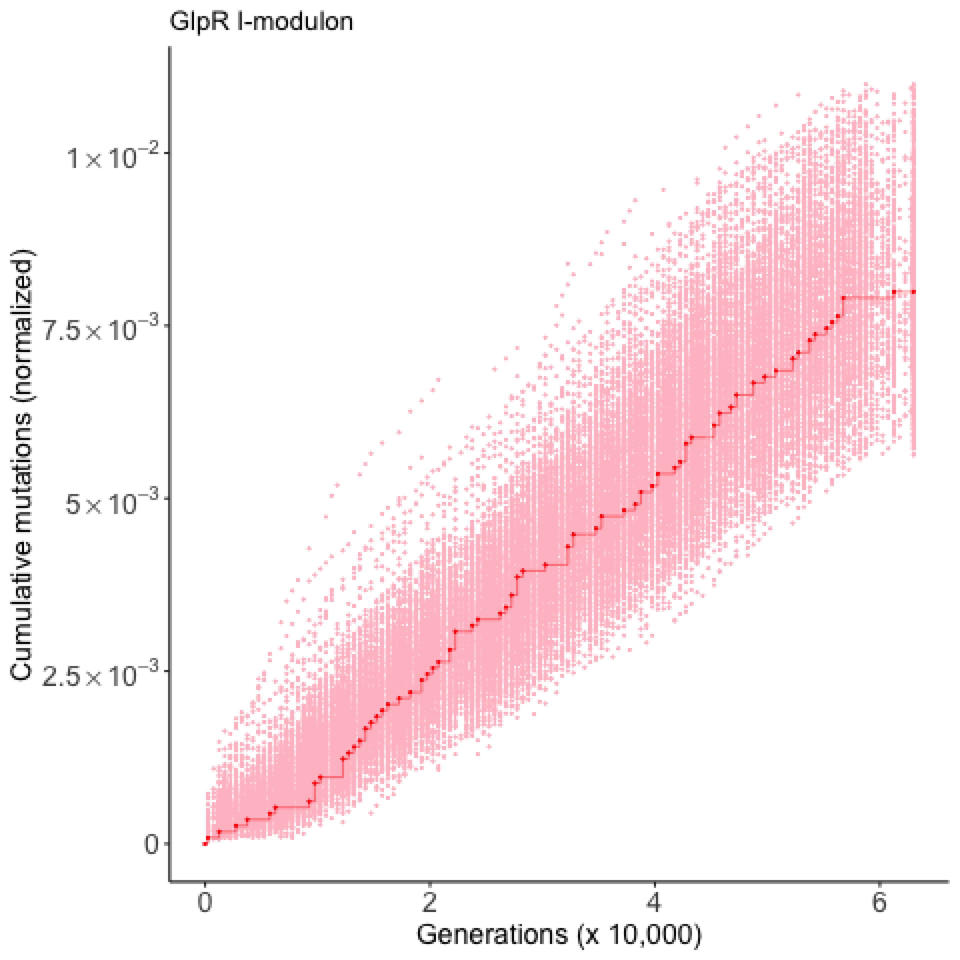

GlpR I-modulon
Cumulative mutations (normalized)
1×10^-2
7.5×10^-3
5×10^-3
2.5×10^-3
0
0
2
4
6
Generations (x 10,000)

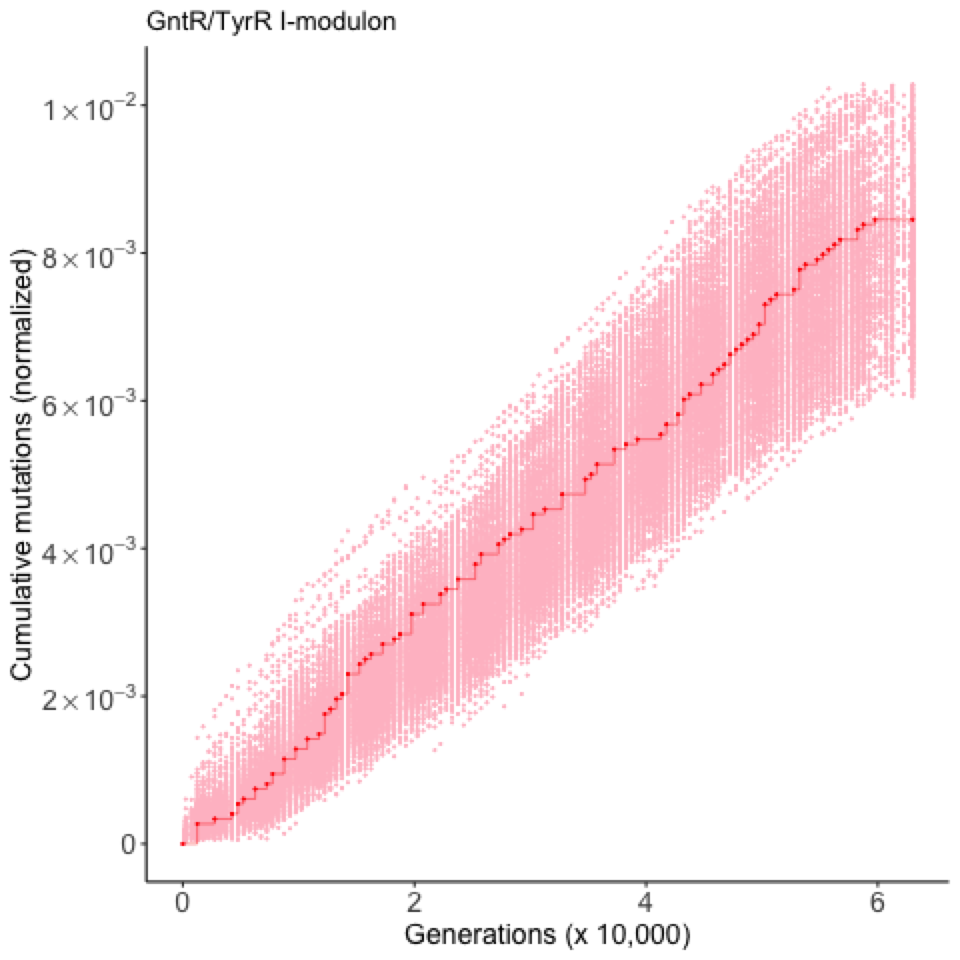
GntR/TyrR I-modulon
Cumulative mutations (normalized)
1×10⁻²
8×10⁻³
6×10⁻³
4×10⁻³
2×10⁻³
0
0
2
4
6
Generations (x 10,000)

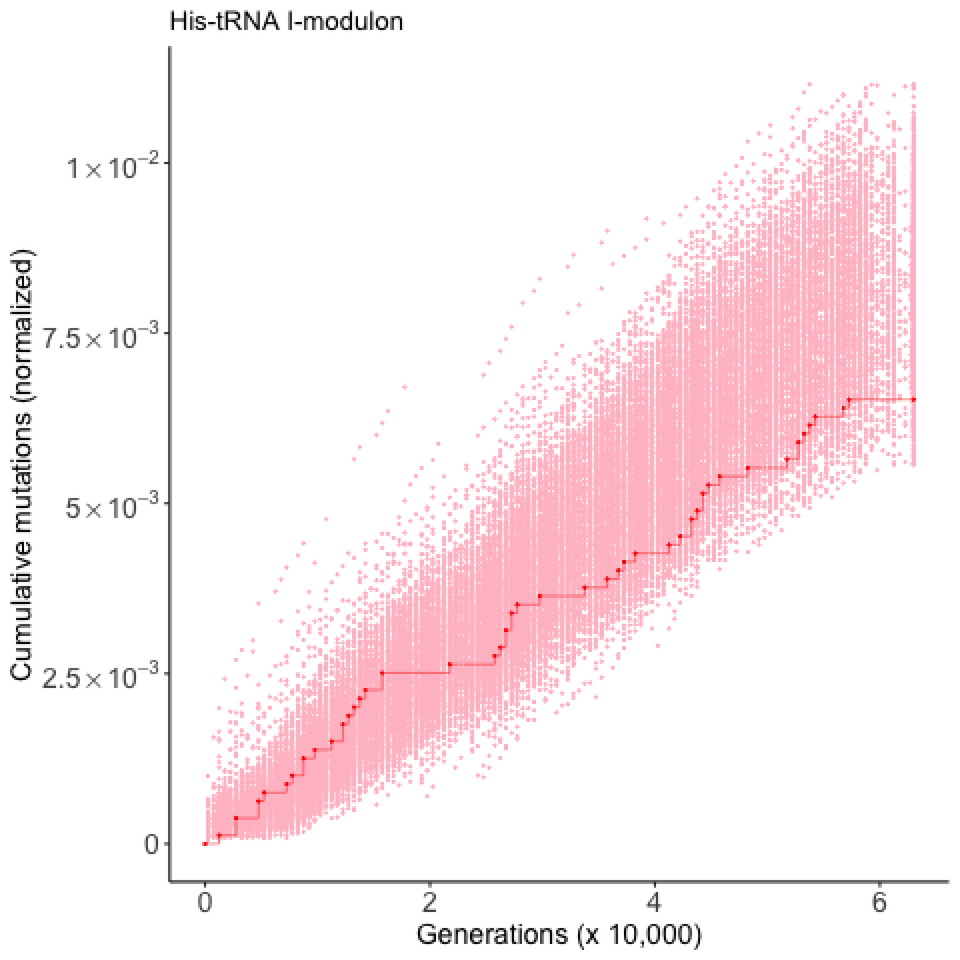

His-tRNA I-modulon
Cumulative mutations (normalized)
1×10^{-2}
7.5×10^{-3}
5×10^{-3}
2.5×10^{-3}
0
0
2
4
6
Generations (x 10,000)

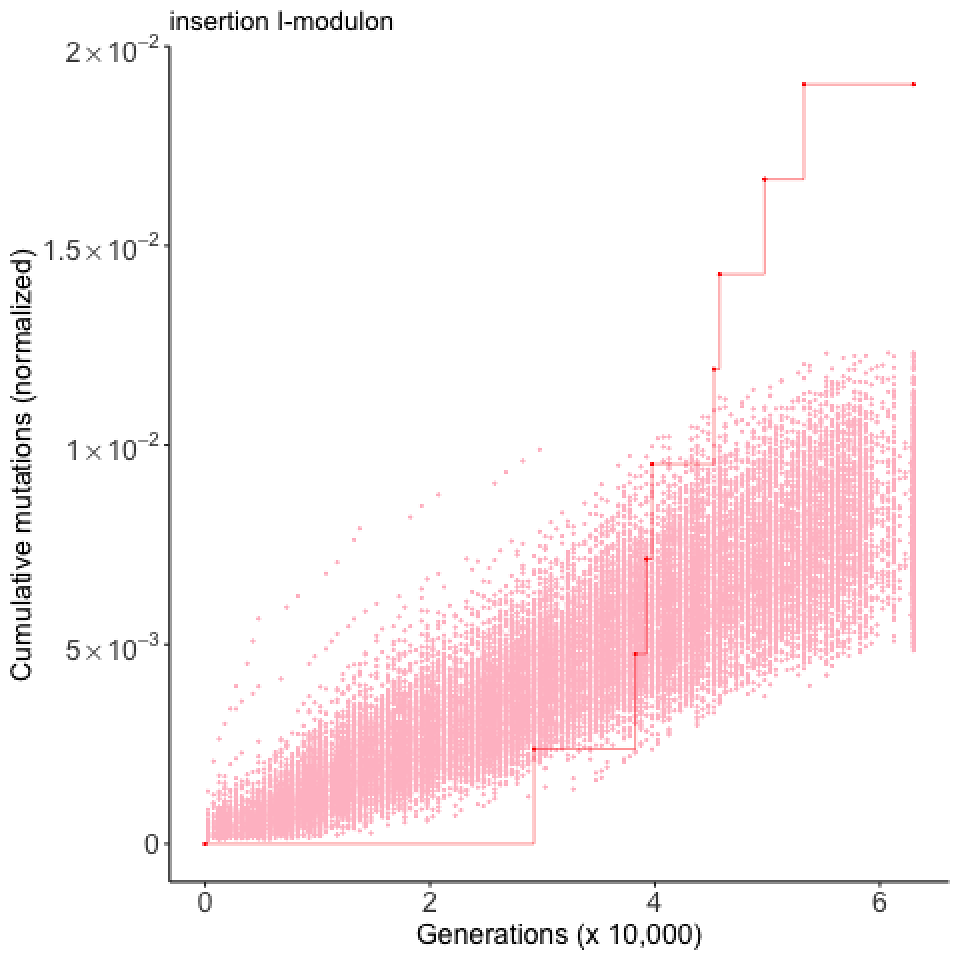
insertion I-modulon
Cumulative mutations (normalized)
2×10⁻²
1.5×10⁻²
1×10⁻²
5×10⁻³
0
0
2
4
6
Generations (x 10,000)

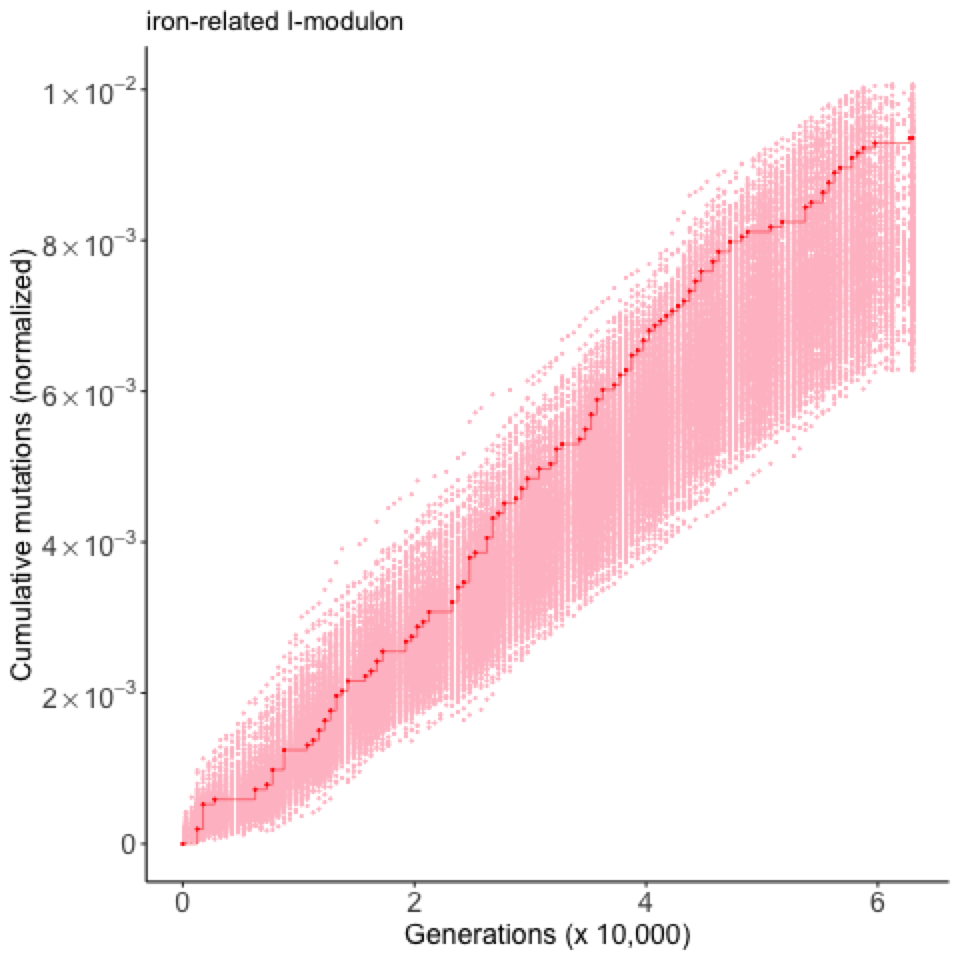

iron-related I-modulon
Cumulative mutations (normalized)
1×10^{-2}
8×10^{-3}
6×10^{-3}
4×10^{-3}
2×10^{-3}
0
0
2
4
6
Generations (x 10,000)

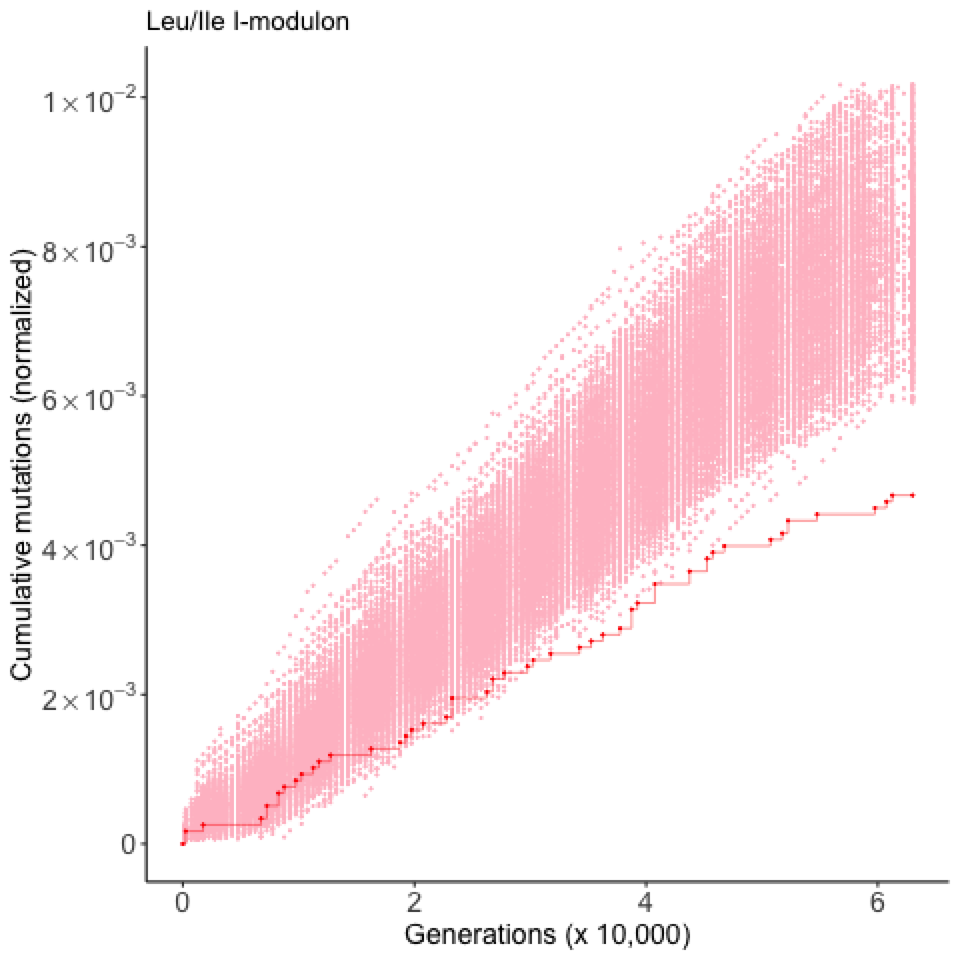

Leu/Ile I-modulon
Cumulative mutations (normalized)
1×10^{-2}
8×10^{-3}
6×10^{-3}
4×10^{-3}
2×10^{-3}
0
0
2
4
6
Generations (x 10,000)

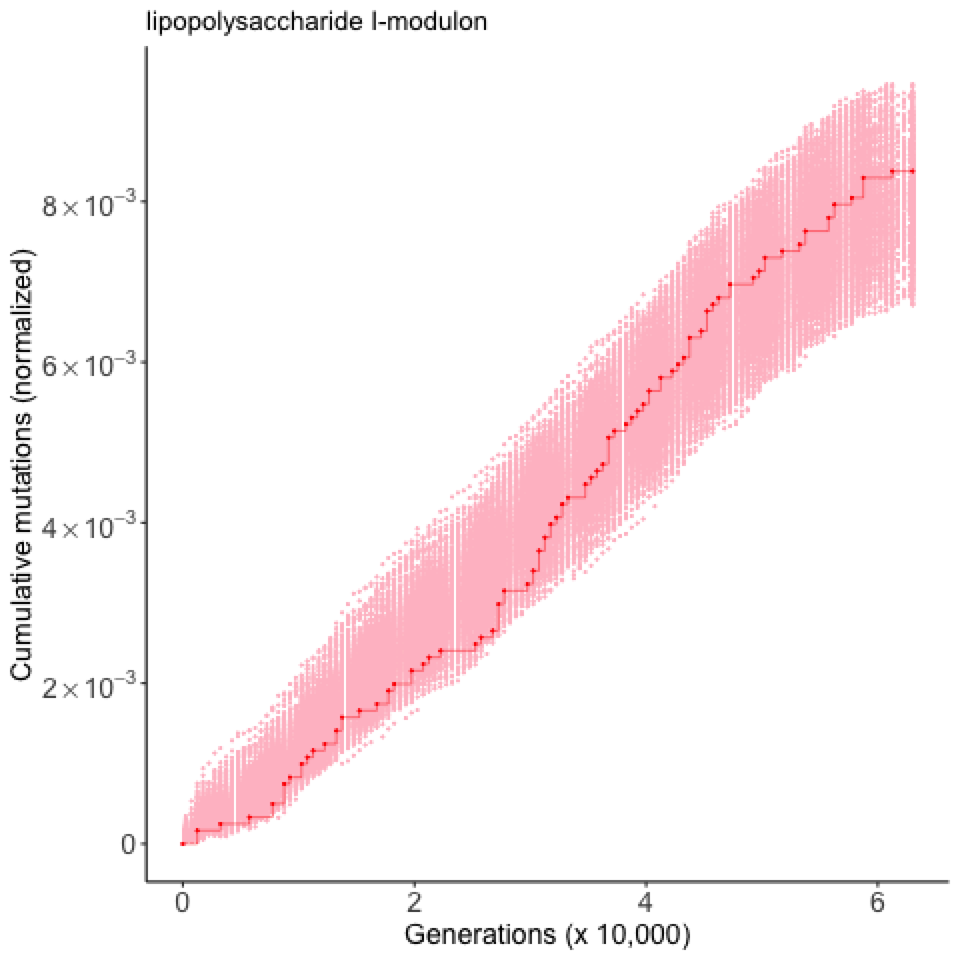

lipopolysaccharide I-modulon
Cumulative mutations (normalized)
8×10^{-3}
6×10^{-3}
4×10^{-3}
2×10^{-3}
0
0
2
4
6
Generations (x 10,000)

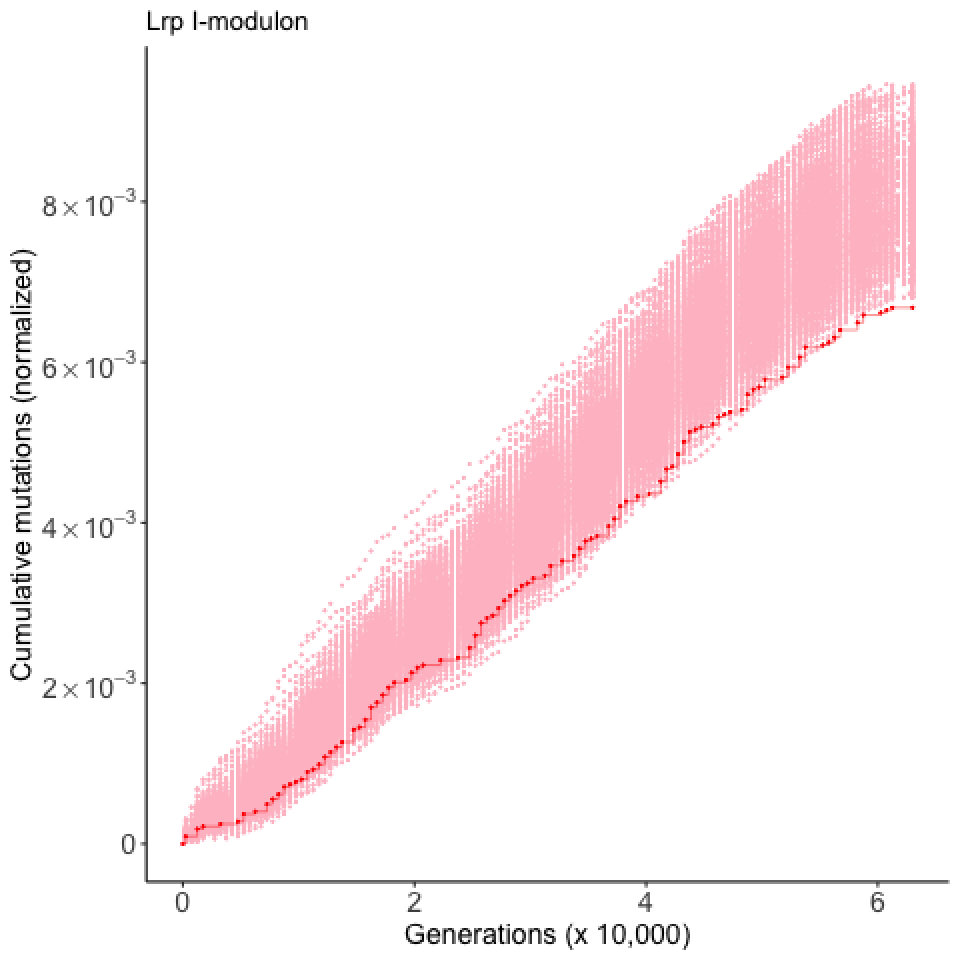

Lrp I-modulon
Cumulative mutations (normalized)
8×10^{-3}
6×10^{-3}
4×10^{-3}
2×10^{-3}
0
0
2
4
6
Generations (x 10,000)

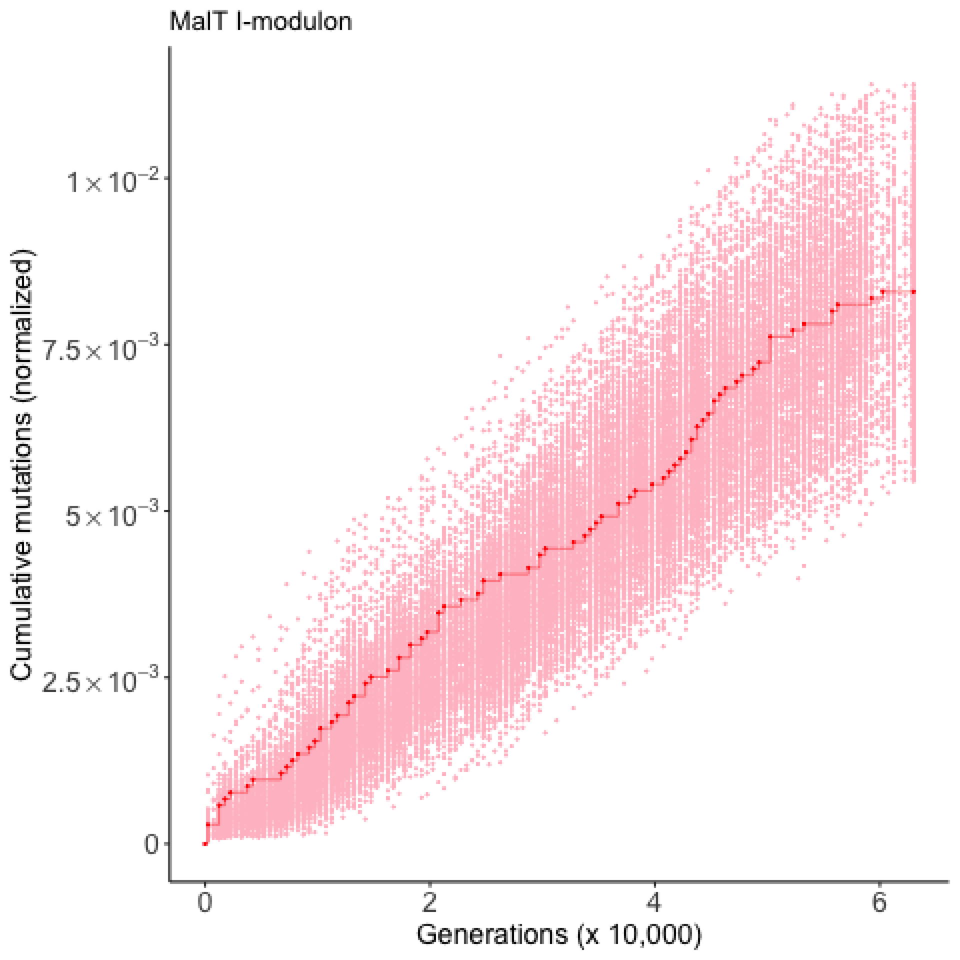

MalT I-modulon
Cumulative mutations (normalized)
1×10^{-2}
7.5×10^{-3}
5×10^{-3}
2.5×10^{-3}
0
0
2
4
6
Generations (x 10,000)

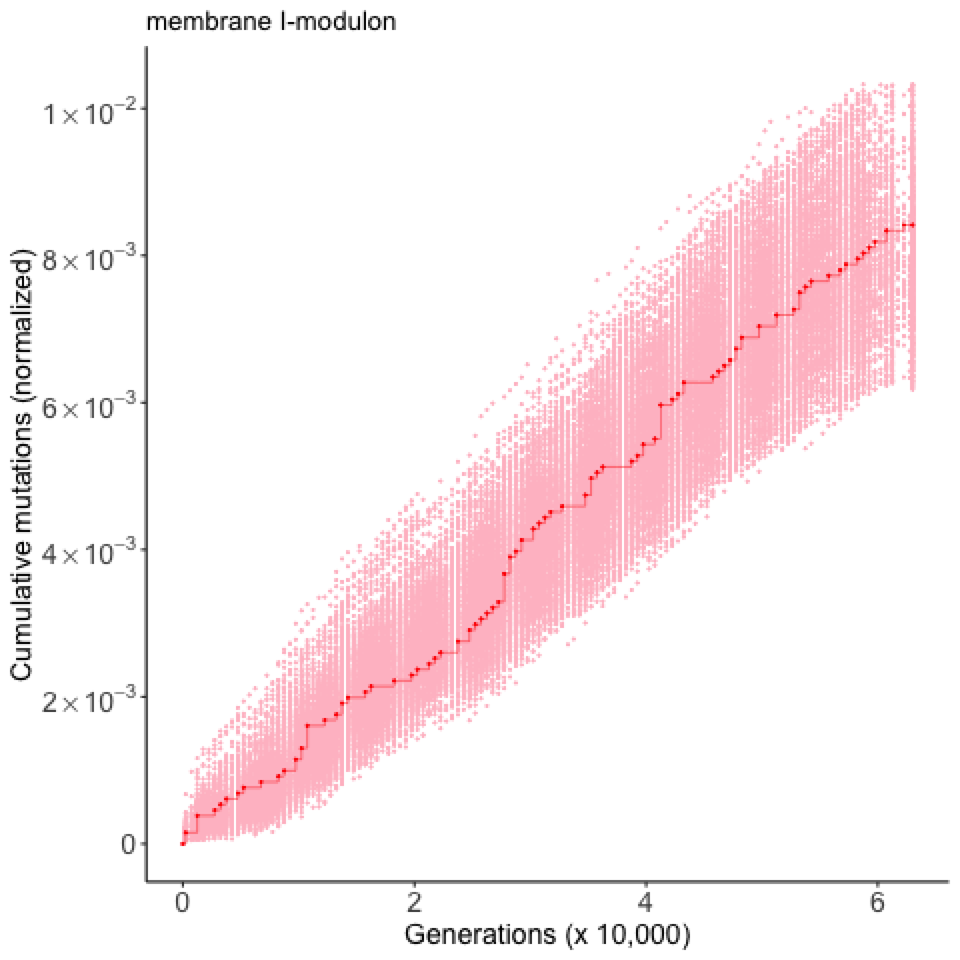

membrane I-modulon
Cumulative mutations (normalized)
1×10⁻²
8×10⁻³
6×10⁻³
4×10⁻³
2×10⁻³
0
0
2
4
6
Generations (x 10,000)

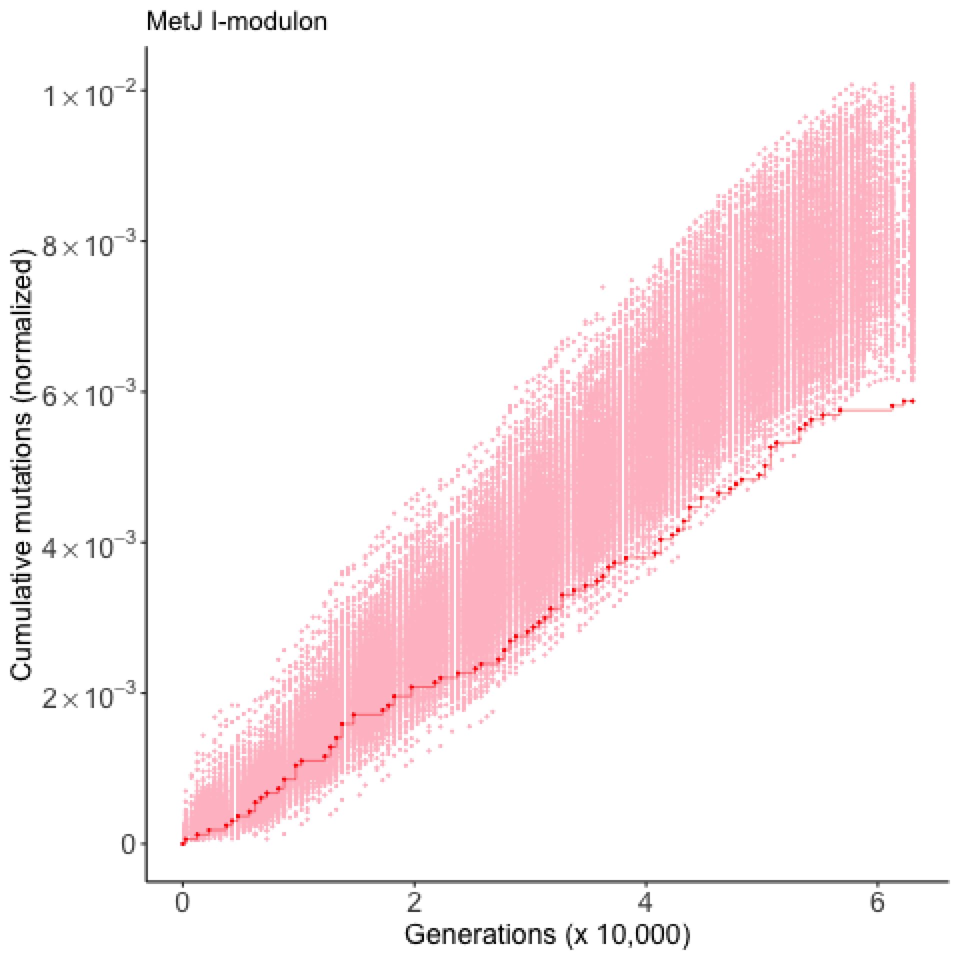

MetJ I-modulon
Cumulative mutations (normalized)
Generations (x 10,000)
1×10^{-2}
8×10^{-3}
6×10^{-3}
4×10^{-3}
2×10^{-3}
0
0
2
4
6

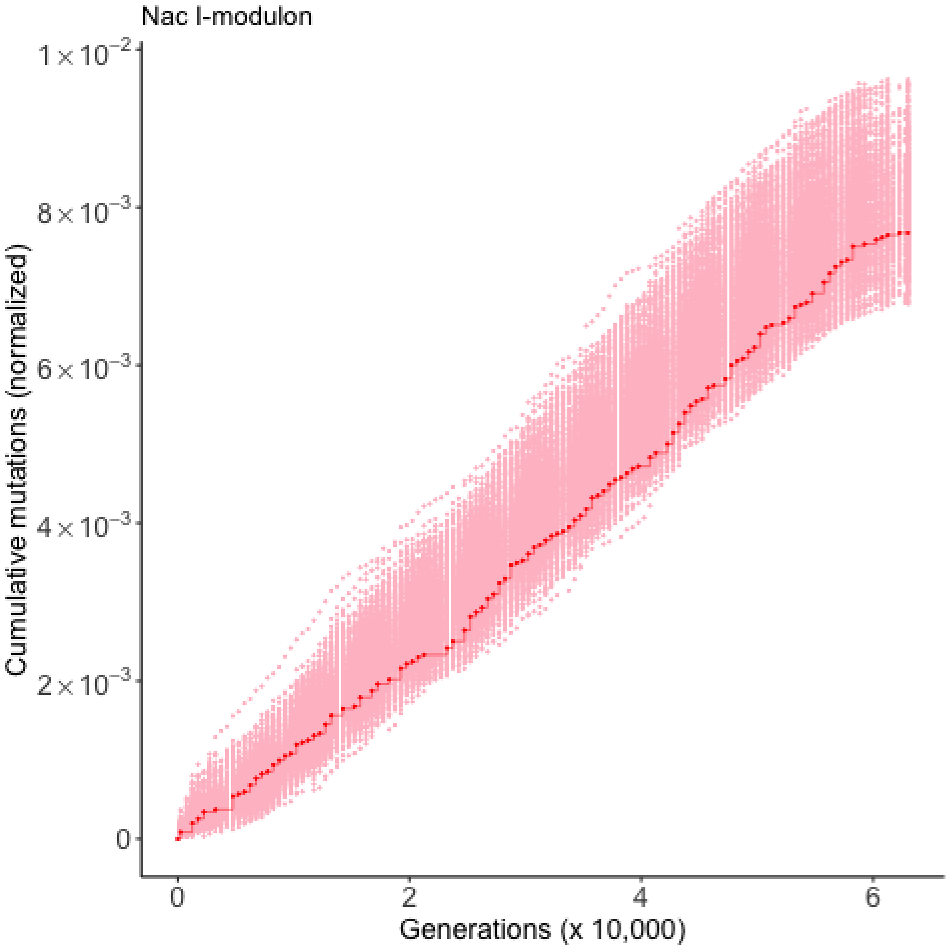

Nac I-modulon
1×10^−2
8×10^−3
6×10^−3
4×10^−3
2×10^−3
0
Cumulative mutations (normalized)
0
2
4
6
Generations (x 10,000)

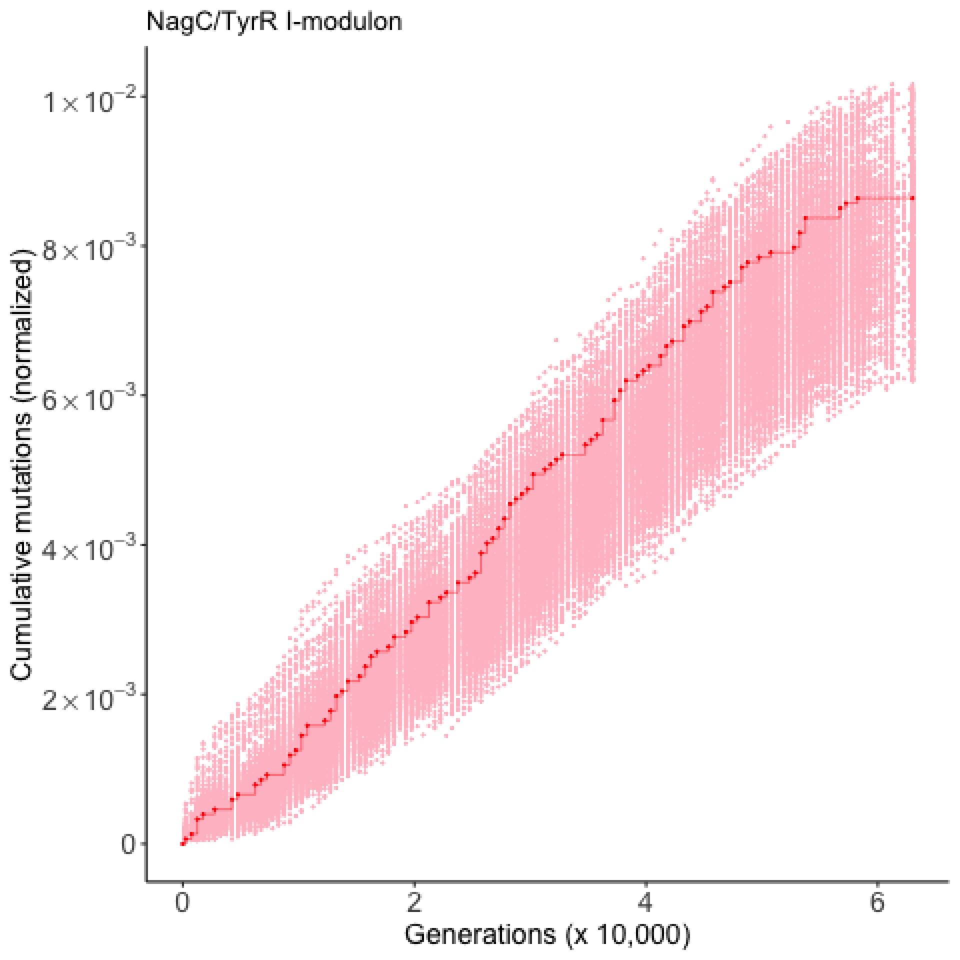

NagC/TyrR I-modulon
Cumulative mutations (normalized)
1×10^{-2}
8×10^{-3}
6×10^{-3}
4×10^{-3}
2×10^{-3}
0
0
2
4
6
Generations (x 10,000)

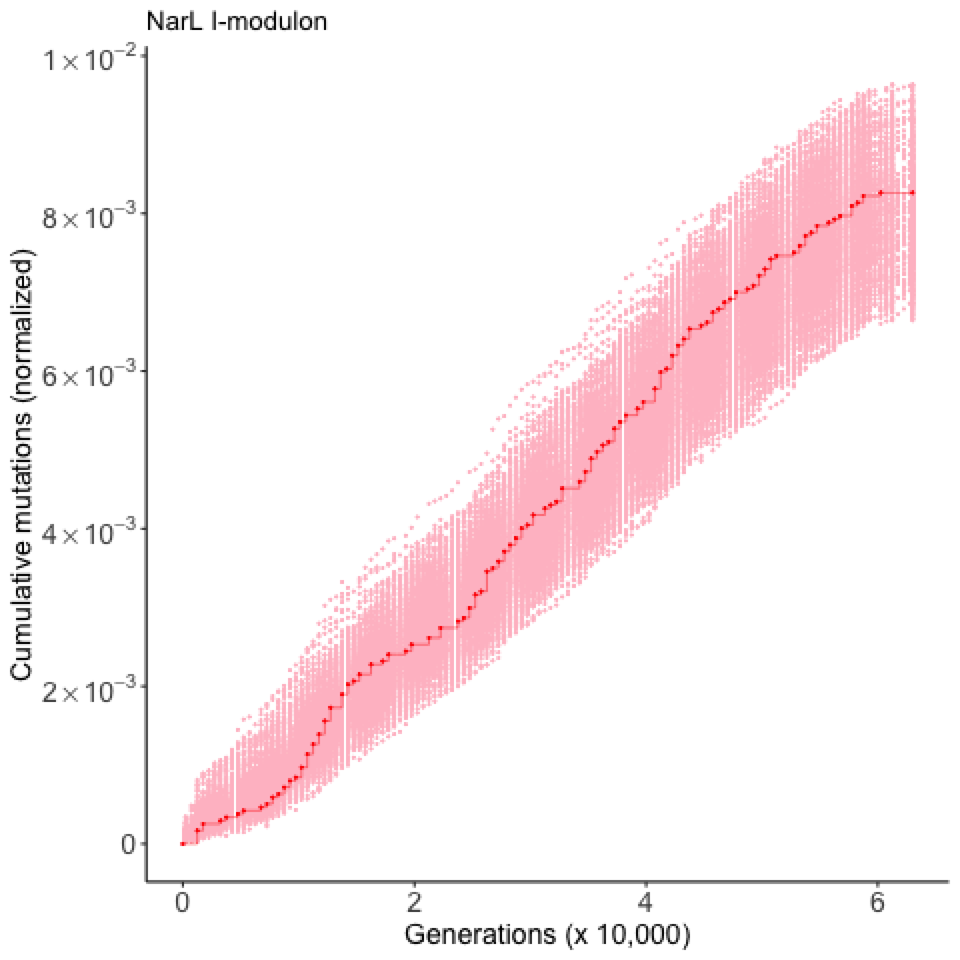

NarL I-modulon
Cumulative mutations (normalized)
1×10^{-2}
8×10^{-3}
6×10^{-3}
4×10^{-3}
2×10^{-3}
0
0
2
4
6
Generations (x 10,000)

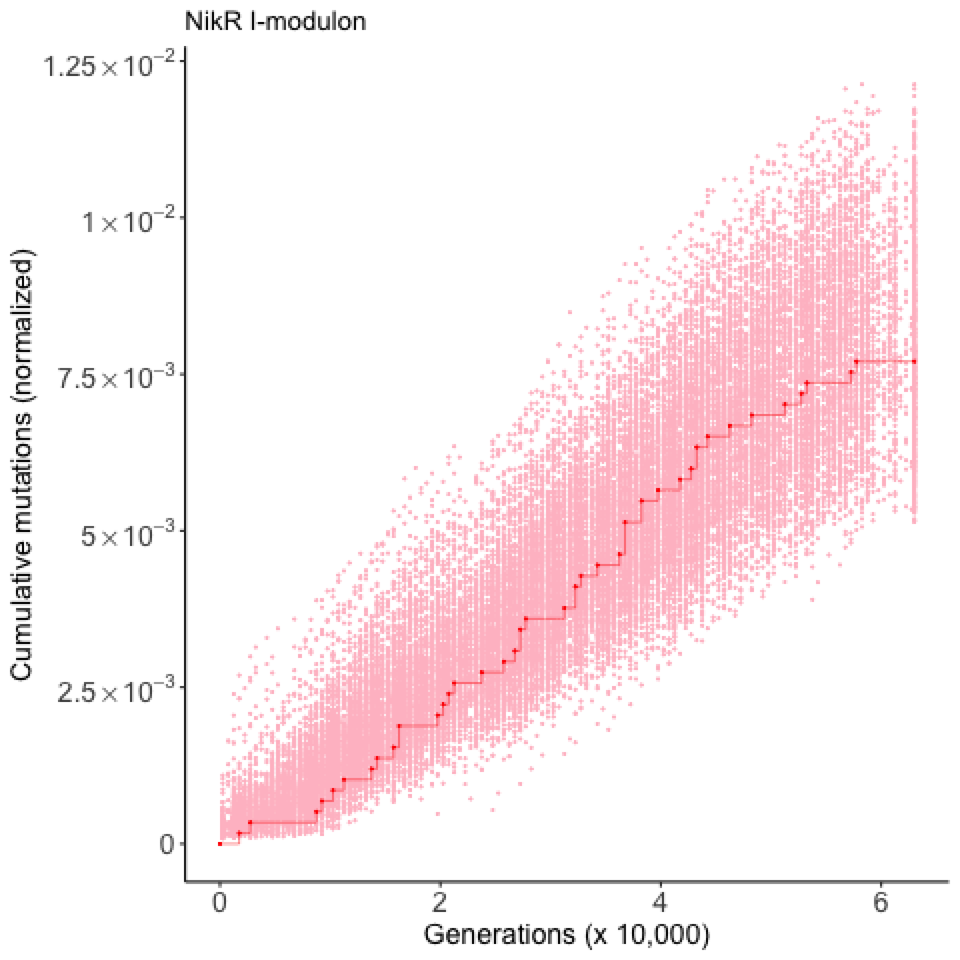

NikR I-modulon
Cumulative mutations (normalized)
1.25×10^−2
1×10^−2
7.5×10^−3
5×10^−3
2.5×10^−3
0
0
2
4
6
Generations (x 10,000)

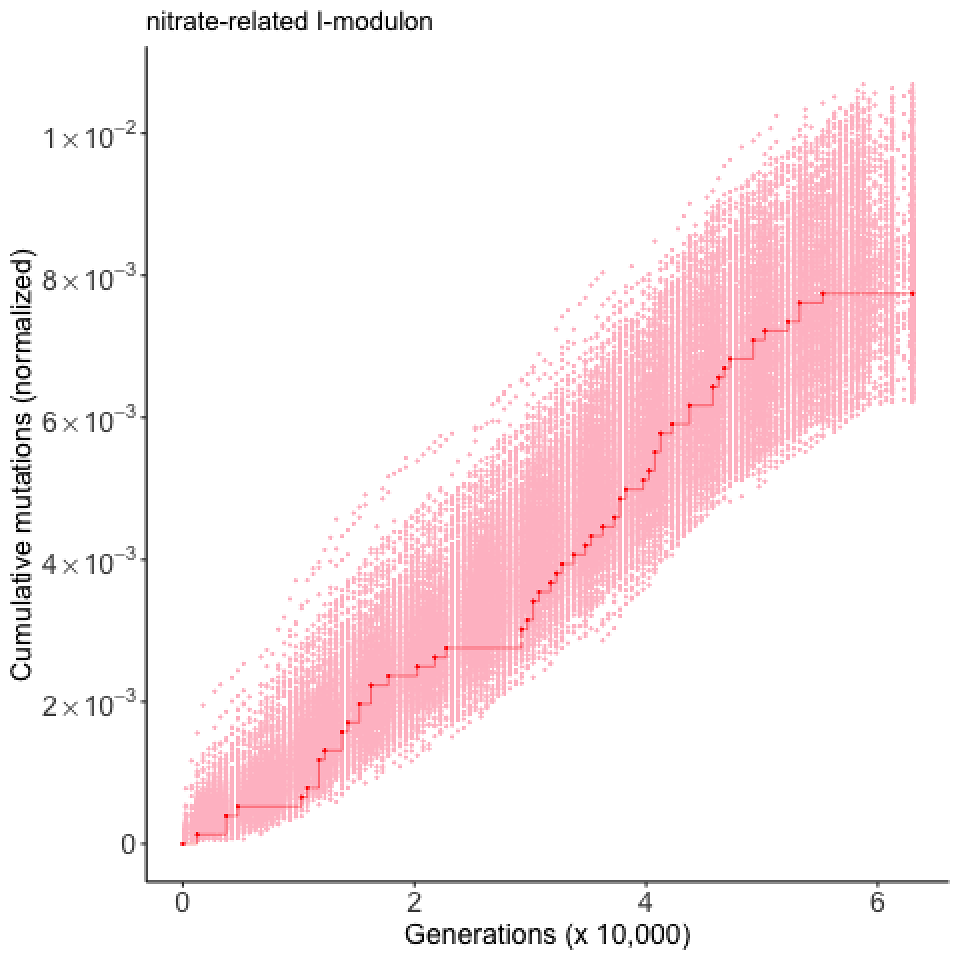

nitrate-related I-modulon
Cumulative mutations (normalized)
1×10^{-2}
8×10^{-3}
6×10^{-3}
4×10^{-3}
2×10^{-3}
0
0
2
4
6
Generations (x 10,000)

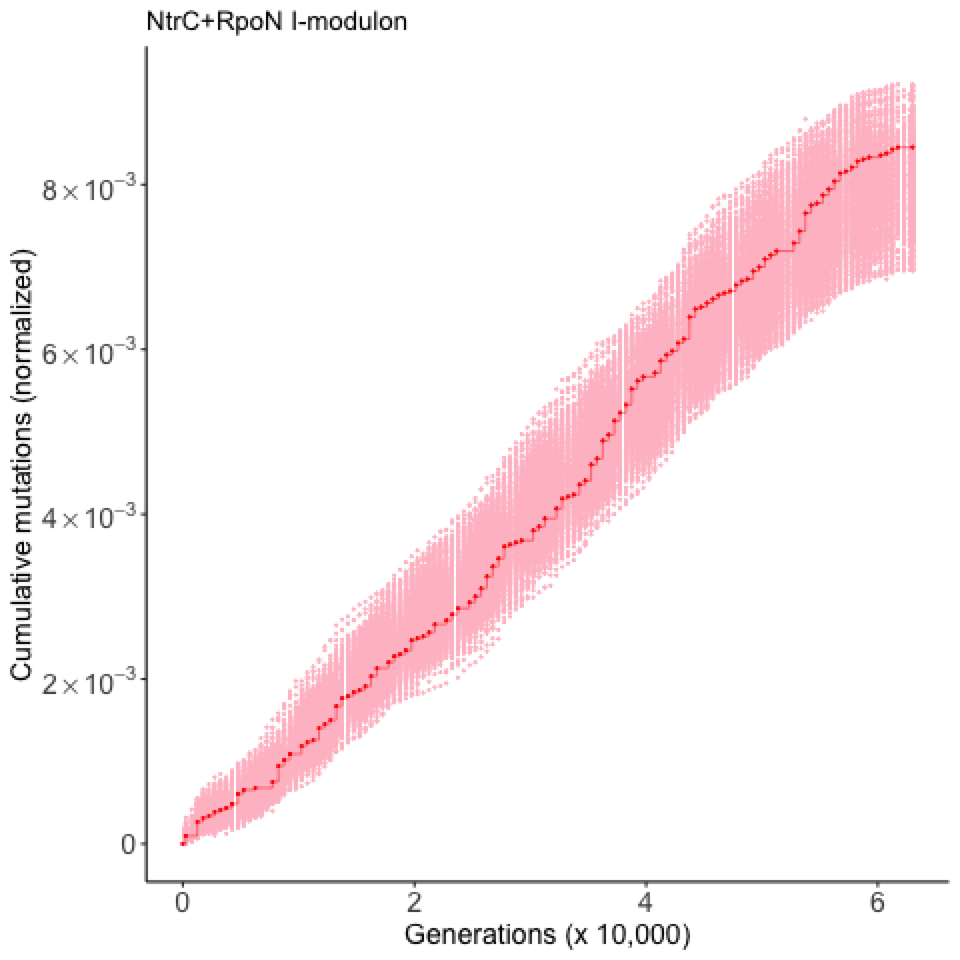
NtrC+RpoN I-modulon
Cumulative mutations (normalized)
8×10^-3
6×10^-3
4×10^-3
2×10^-3
0
0
2
4
6
Generations (x 10,000)

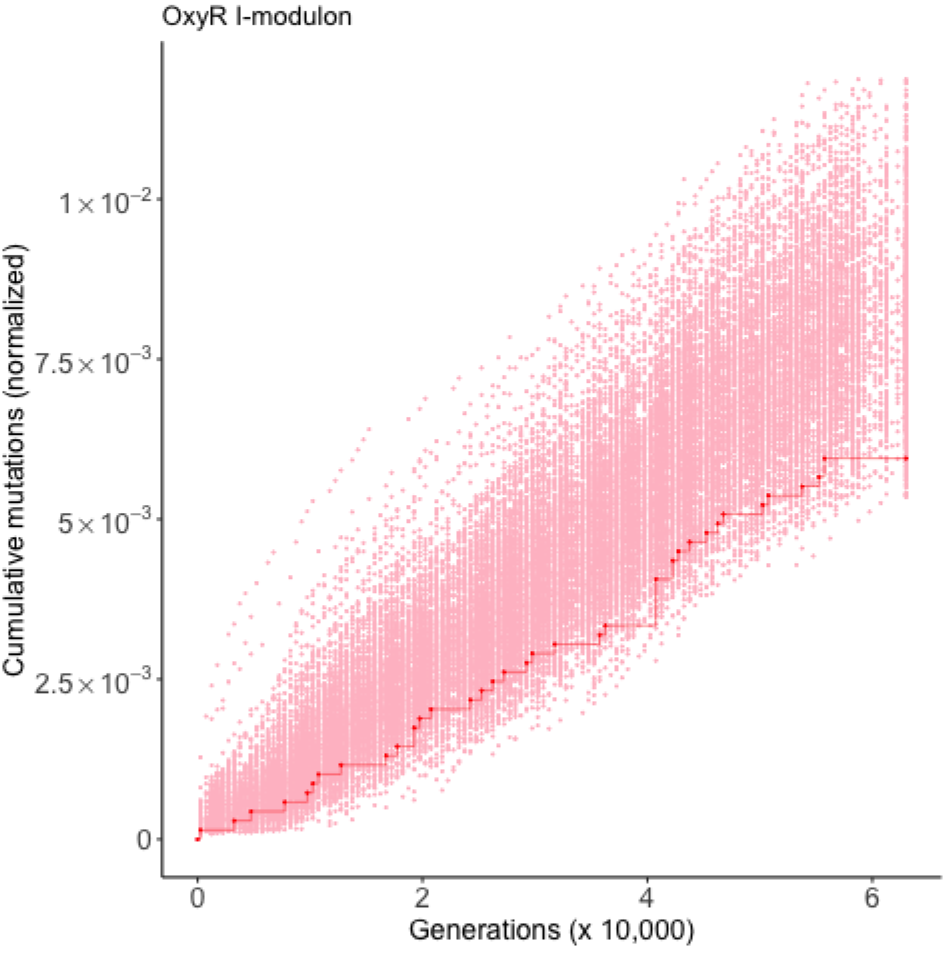

OxyR I-modulon
Cumulative mutations (normalized)
1×10⁻²
7.5×10⁻³
5×10⁻³
2.5×10⁻³
0
0
2
4
6
Generations (x 10,000)

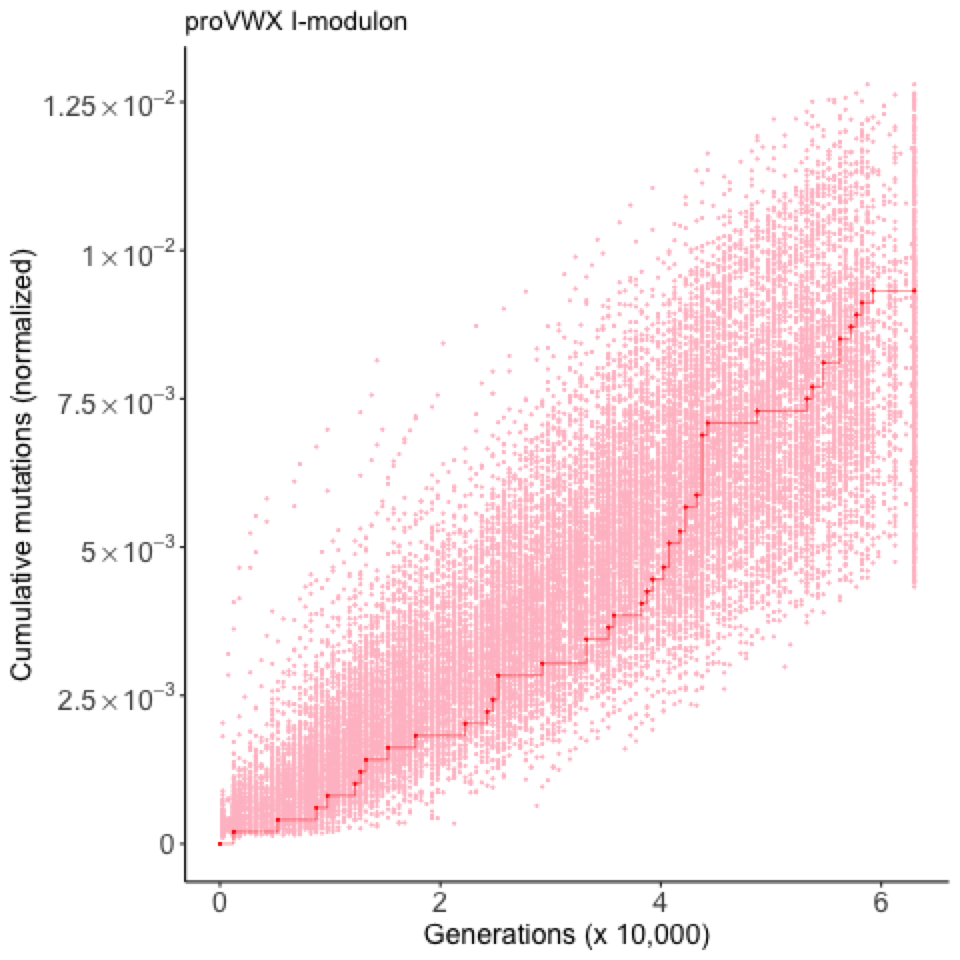

proVWX I-modulon
Cumulative mutations (normalized)
1.25×10⁻²
1×10⁻²
7.5×10⁻³
5×10⁻³
2.5×10⁻³
0
0
2
4
6
Generations (x 10,000)

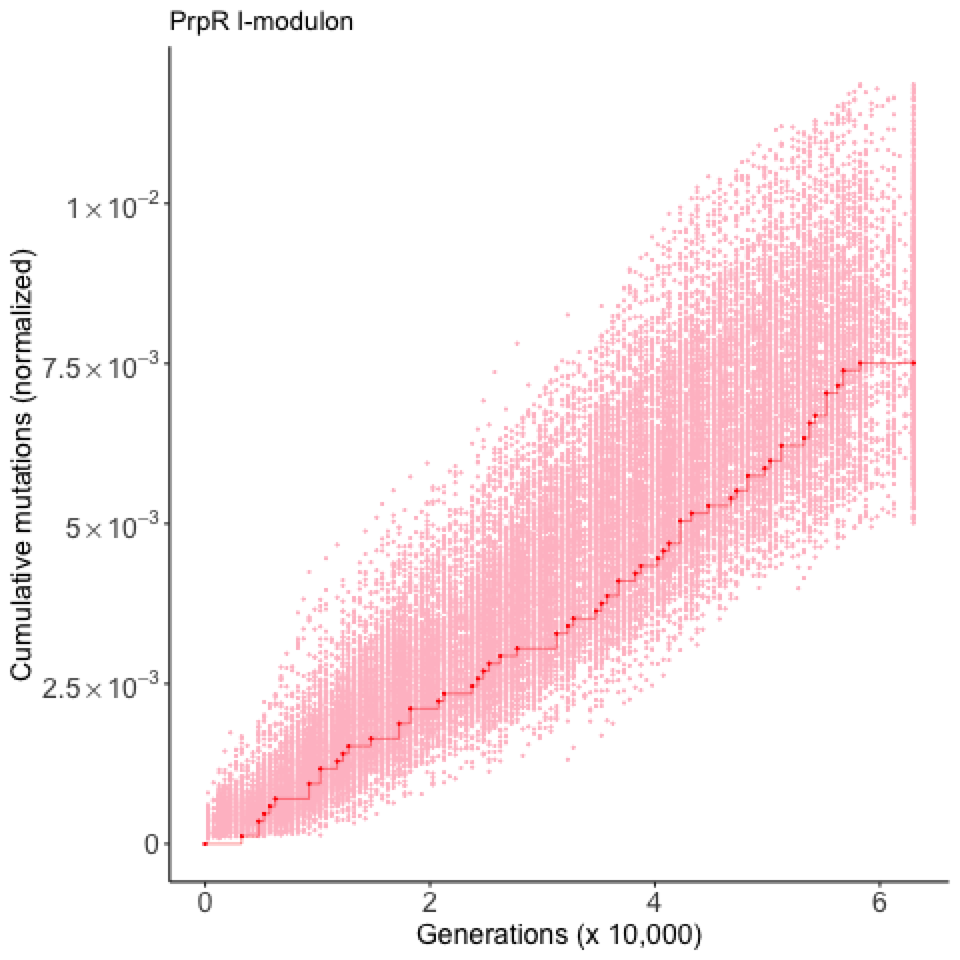

PrpR I-modulon
Cumulative mutations (normalized)
1×10^-2
7.5×10^-3
5×10^-3
2.5×10^-3
0
0
2
4
6
Generations (x 10,000)

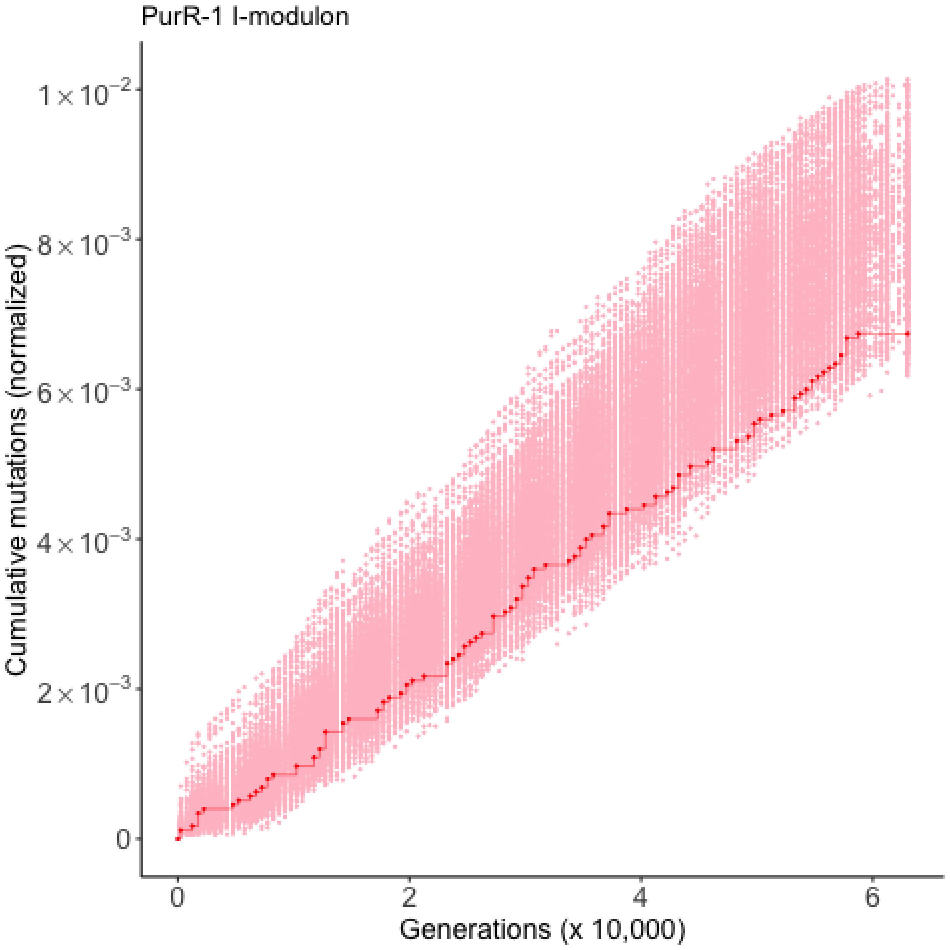
PurR-1 I-modulon
1×10^{-2}
8×10^{-3}
6×10^{-3}
4×10^{-3}
2×10^{-3}
0
Cumulative mutations (normalized)
0
2
4
6
Generations (x 10,000)

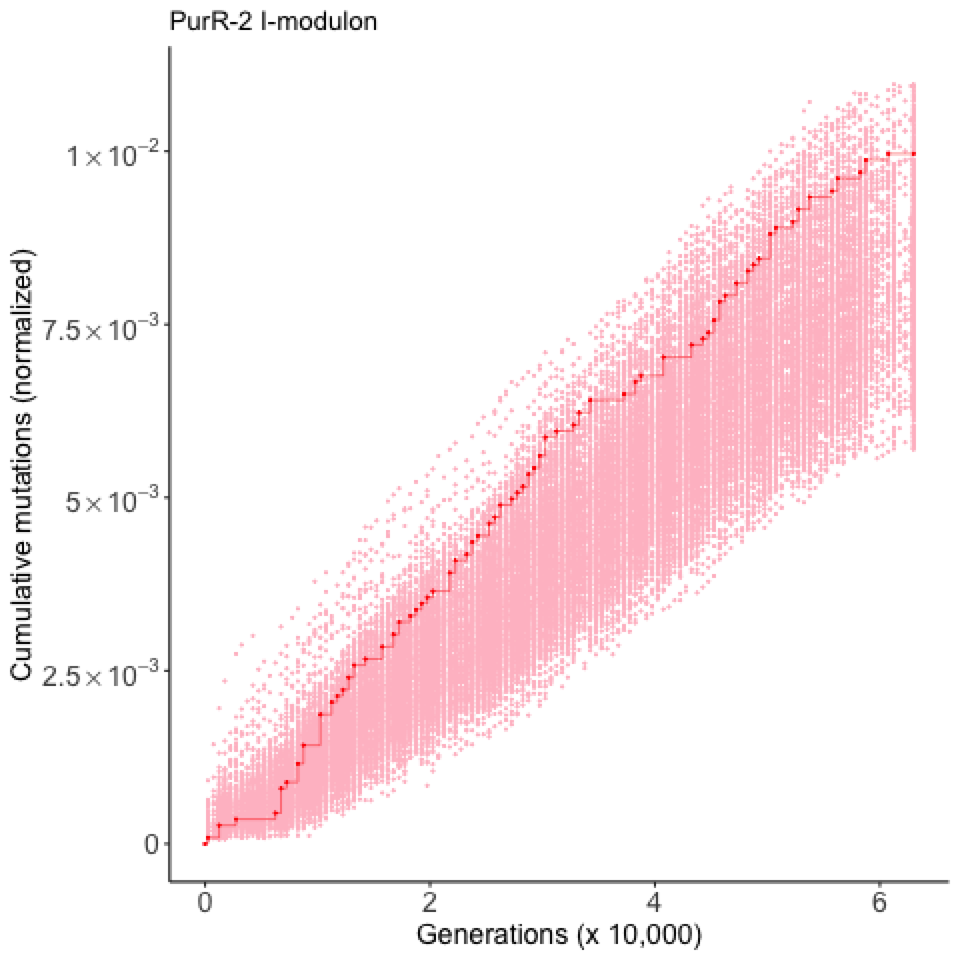
PurR-2 I-modulon
Cumulative mutations (normalized)
1×10^{-2}
7.5×10^{-3}
5×10^{-3}
2.5×10^{-3}
0
0
2
4
6
Generations (x 10,000)

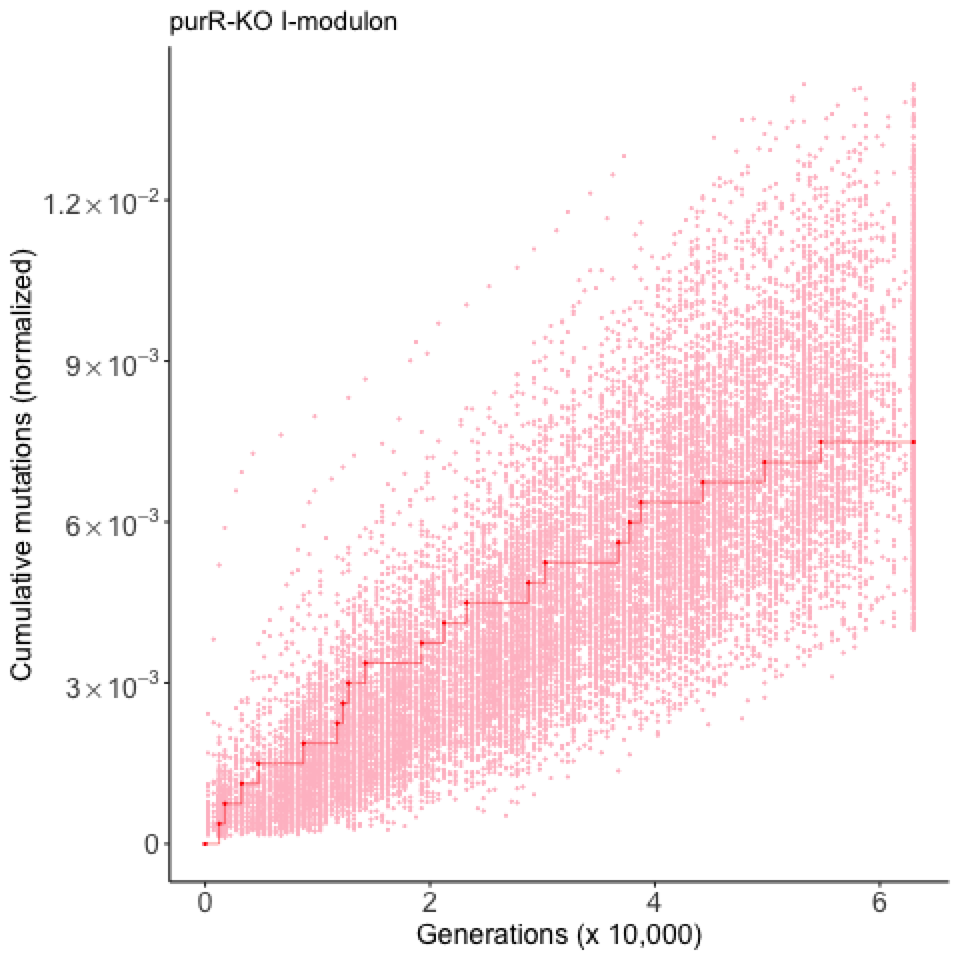
purR-KO I-modulon
Cumulative mutations (normalized)
1.2×10^-2
9×10^-3
6×10^-3
3×10^-3
0
0
2
4
6
Generations (x 10,000)

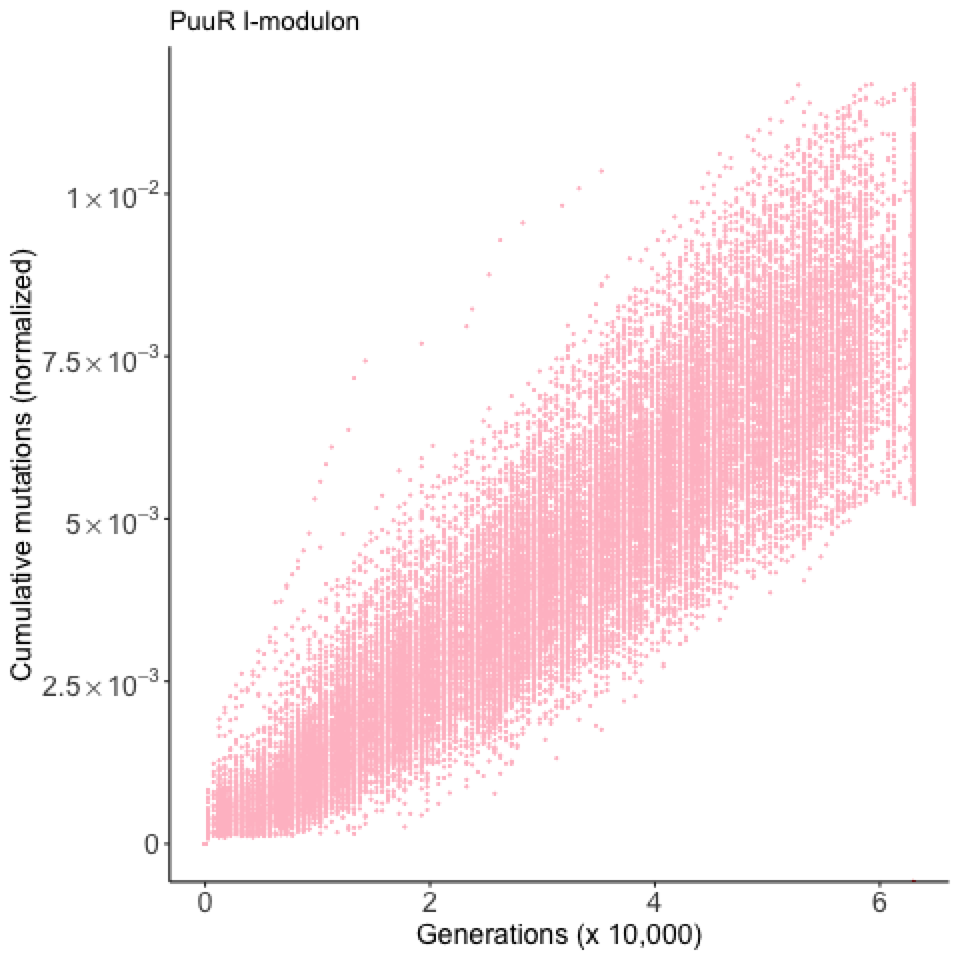

PuuR I-modulon
Cumulative mutations (normalized)
1×10^{-2}
7.5×10^{-3}
5×10^{-3}
2.5×10^{-3}
0
0
2
4
6
Generations (x 10,000)

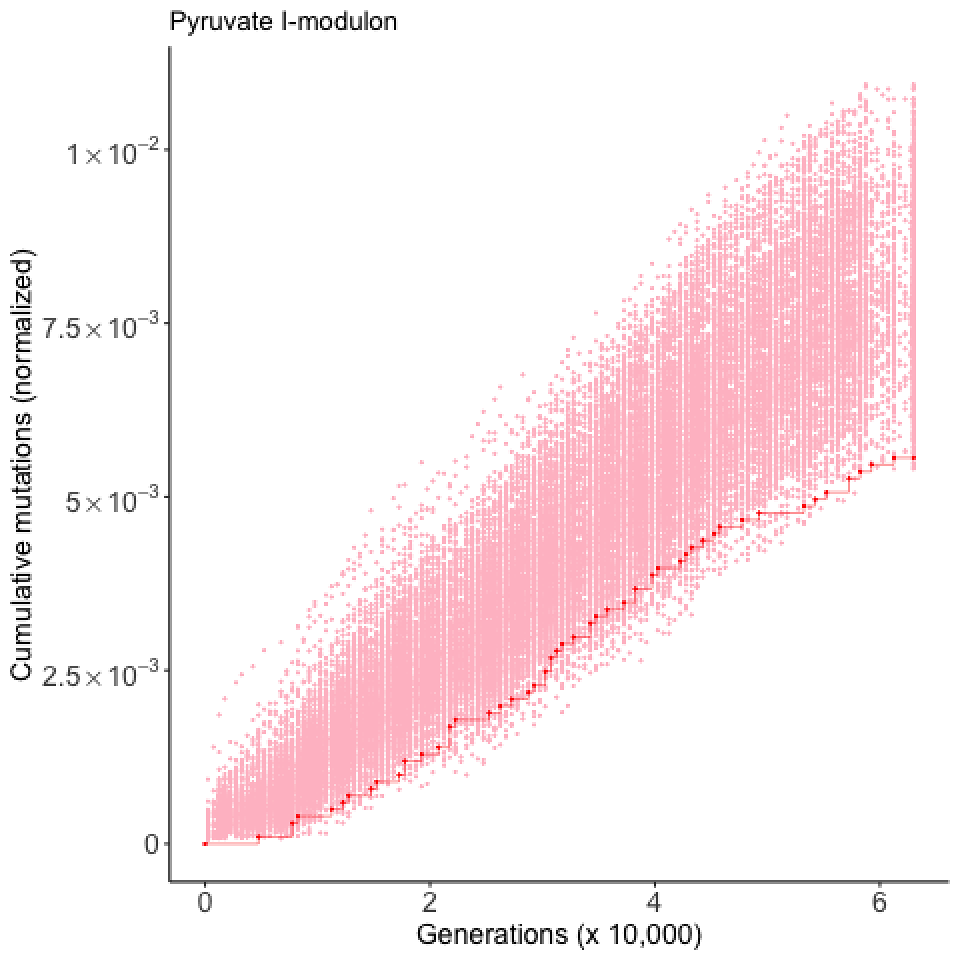

Pyruvate I-modulon
Cumulative mutations (normalized)
1×10^{-2}
7.5×10^{-3}
5×10^{-3}
2.5×10^{-3}
0
0
2
4
6
Generations (x 10,000)

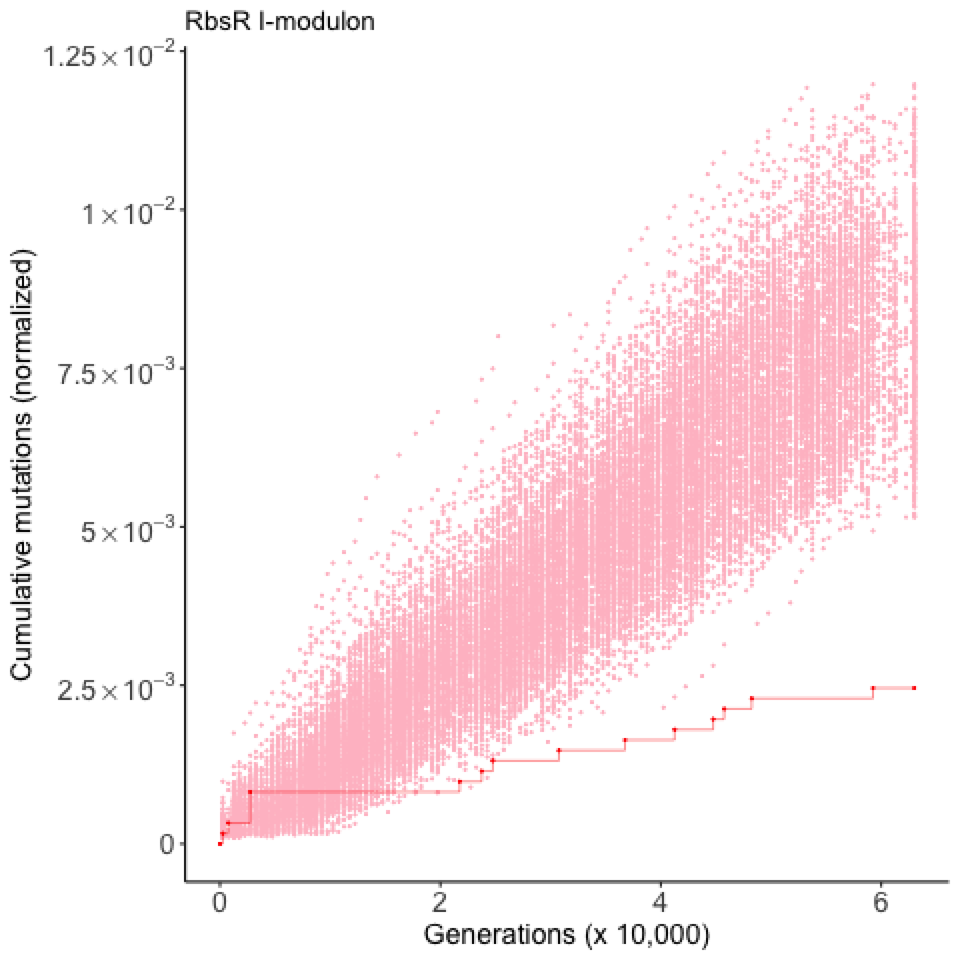

RbsR I-modulon
1.25 × 10⁻²
1 × 10⁻²
7.5 × 10⁻³
5 × 10⁻³
2.5 × 10⁻³
0
Cumulative mutations (normalized)
0
2
4
6
Generations (x 10,000)

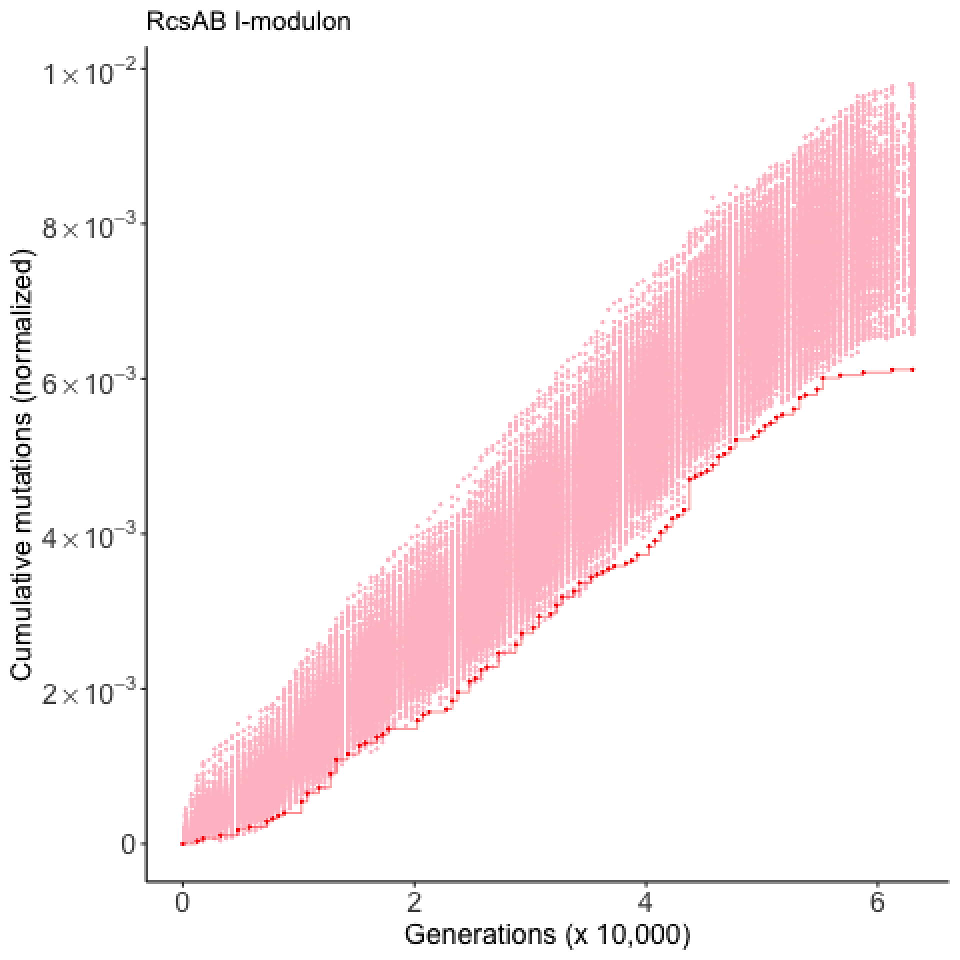

RcsAB I-modulon
Cumulative mutations (normalized)
1×10^{-2}
8×10^{-3}
6×10^{-3}
4×10^{-3}
2×10^{-3}
0
0
2
4
6
Generations (x 10,000)

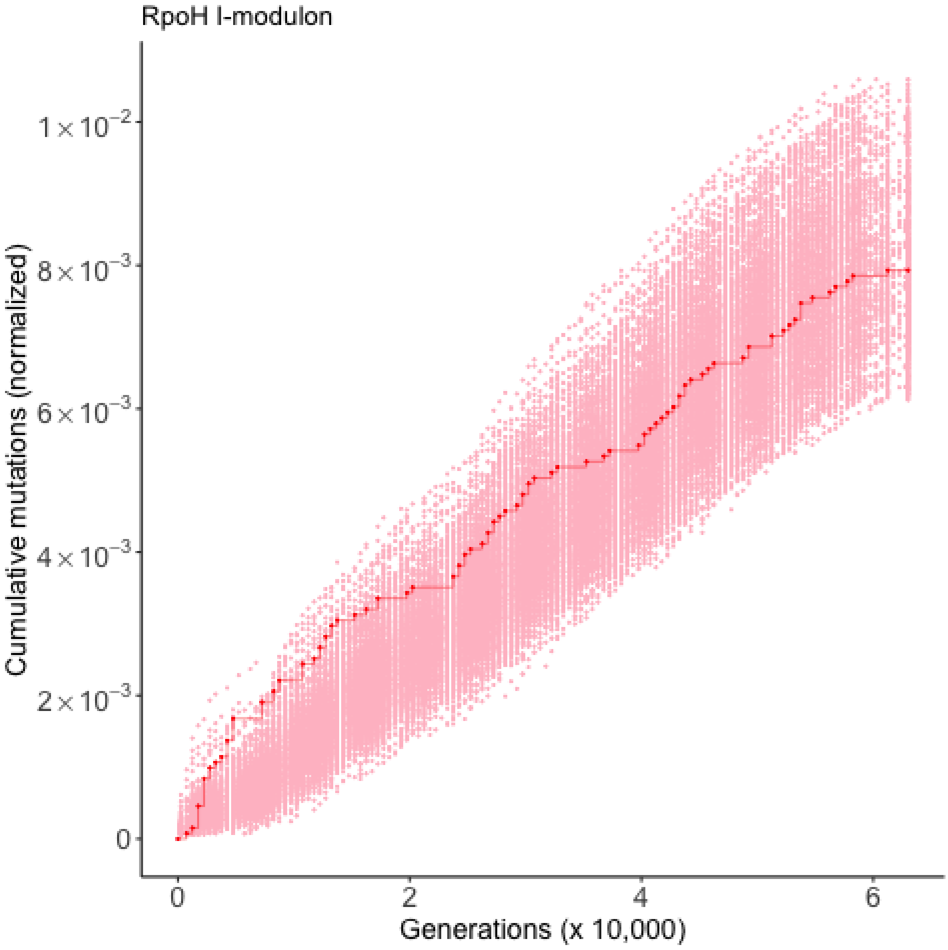

RpoH I-modulon
Cumulative mutations (normalized)
1×10^{-2}
8×10^{-3}
6×10^{-3}
4×10^{-3}
2×10^{-3}
0
0
2
4
6
Generations (x 10,000)

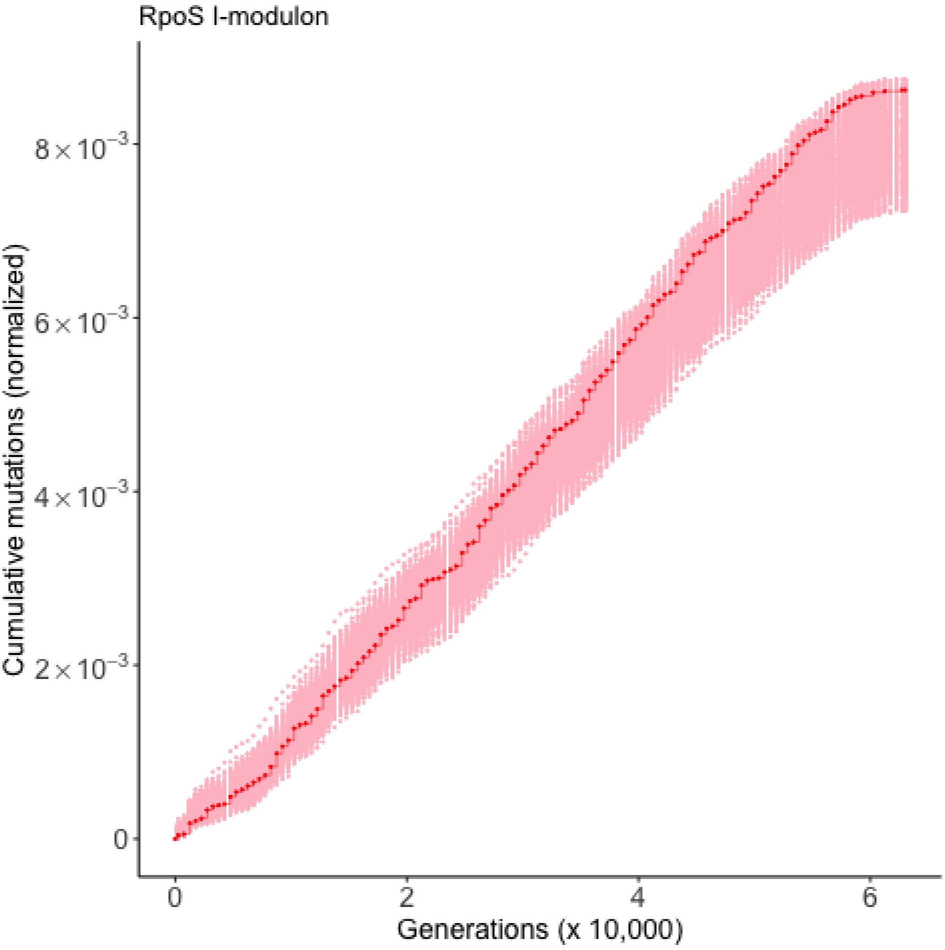

RpoS I-modulon
Cumulative mutations (normalized)
8×10^−3
6×10^−3
4×10^−3
2×10^−3
0
0
2
4
6
Generations (x 10,000)

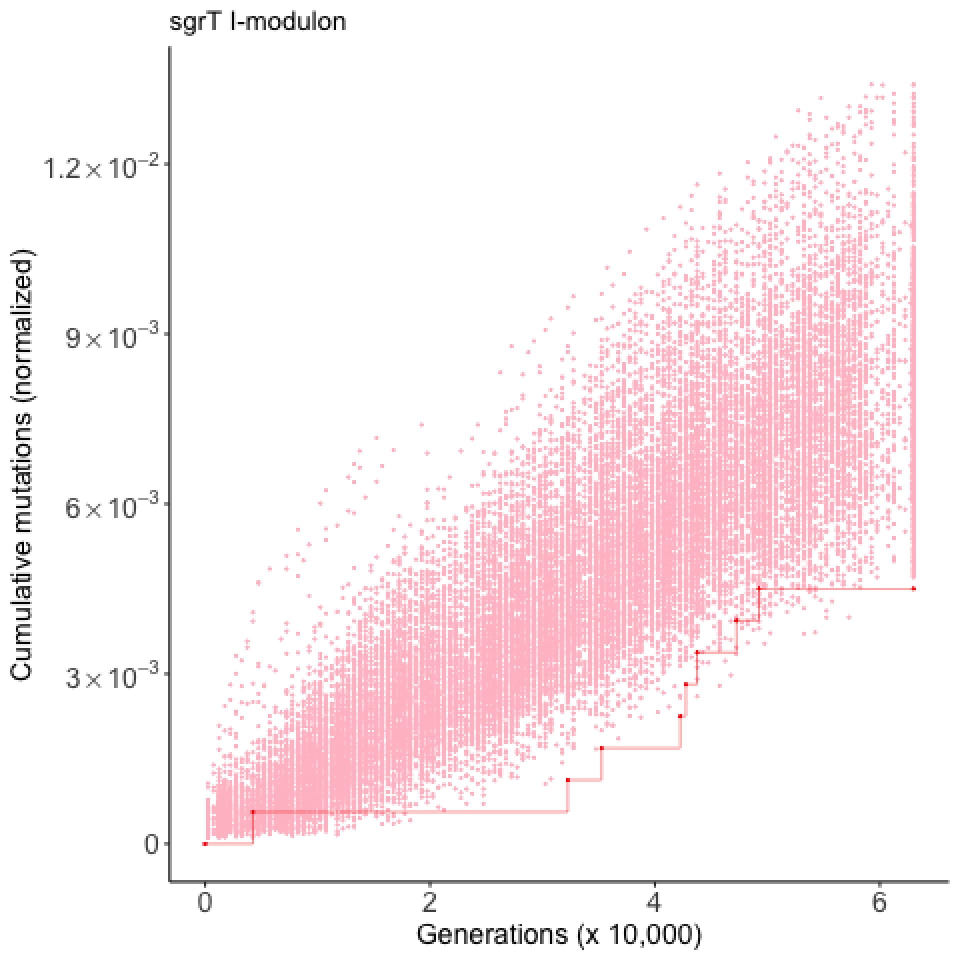

sgrT I-modulon
Cumulative mutations (normalized)
1.2×10^{-2}
9×10^{-3}
6×10^{-3}
3×10^{-3}
0
0
2
4
6
Generations (x 10,000)

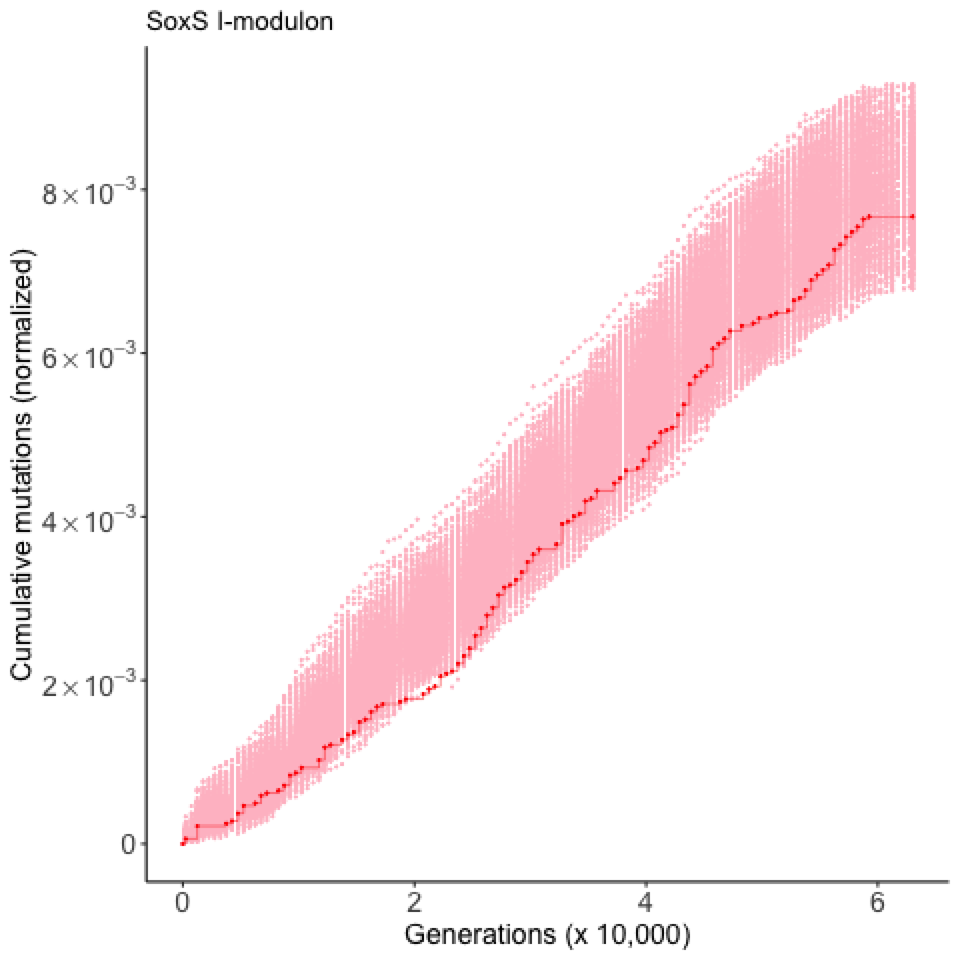

SoxS I-modulon
Cumulative mutations (normalized)
8×10^{-3}
6×10^{-3}
4×10^{-3}
2×10^{-3}
0
0
2
4
6
Generations (x 10,000)

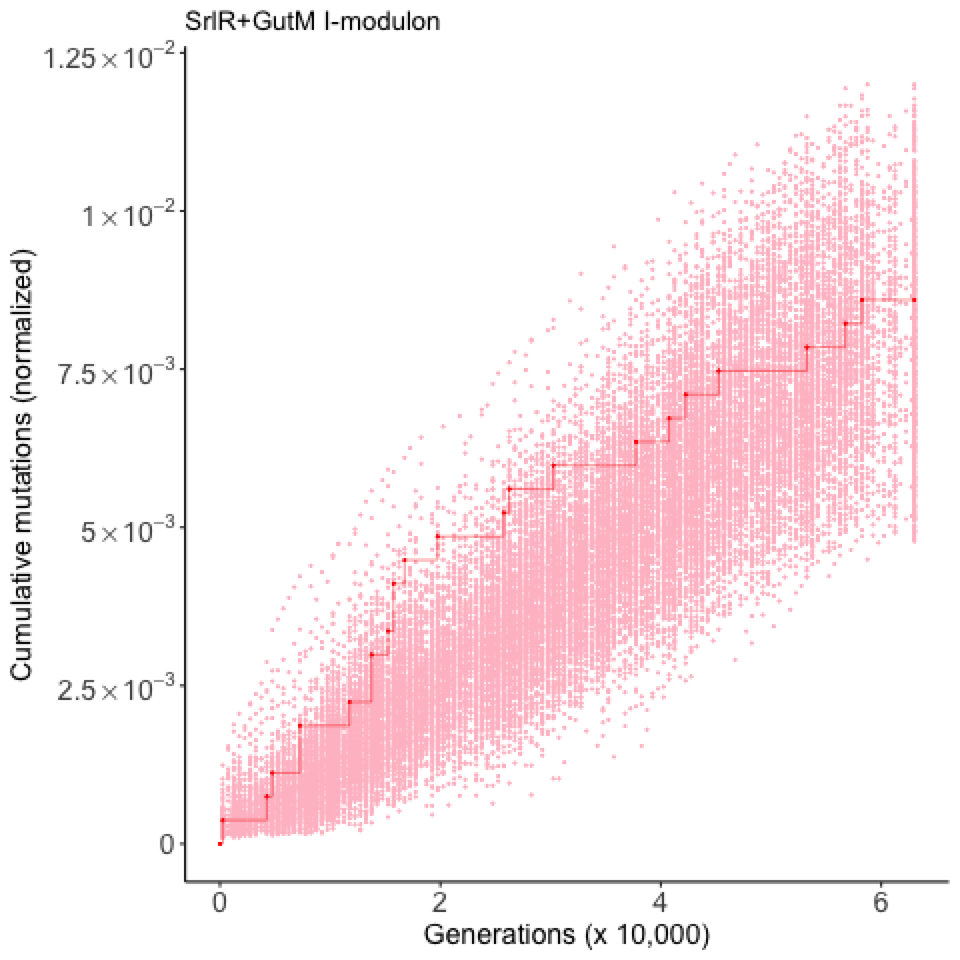
SrlR+GutM I-modulon
Cumulative mutations (normalized)
1.25×10^{-2}
1×10^{-2}
7.5×10^{-3}
5×10^{-3}
2.5×10^{-3}
0
0
2
4
6
Generations (x 10,000)

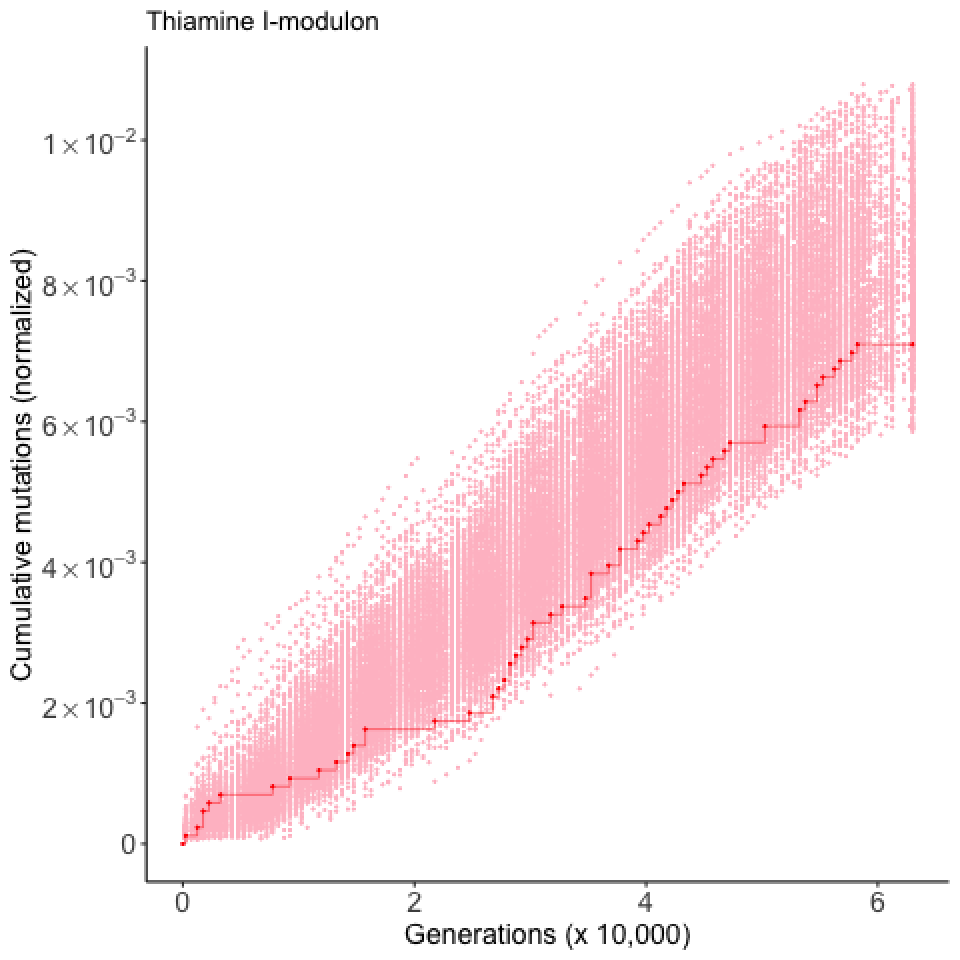

Thiamine I-modulon
Cumulative mutations (normalized)
1×10^{-2}
8×10^{-3}
6×10^{-3}
4×10^{-3}
2×10^{-3}
0
0
2
4
6
Generations (x 10,000)

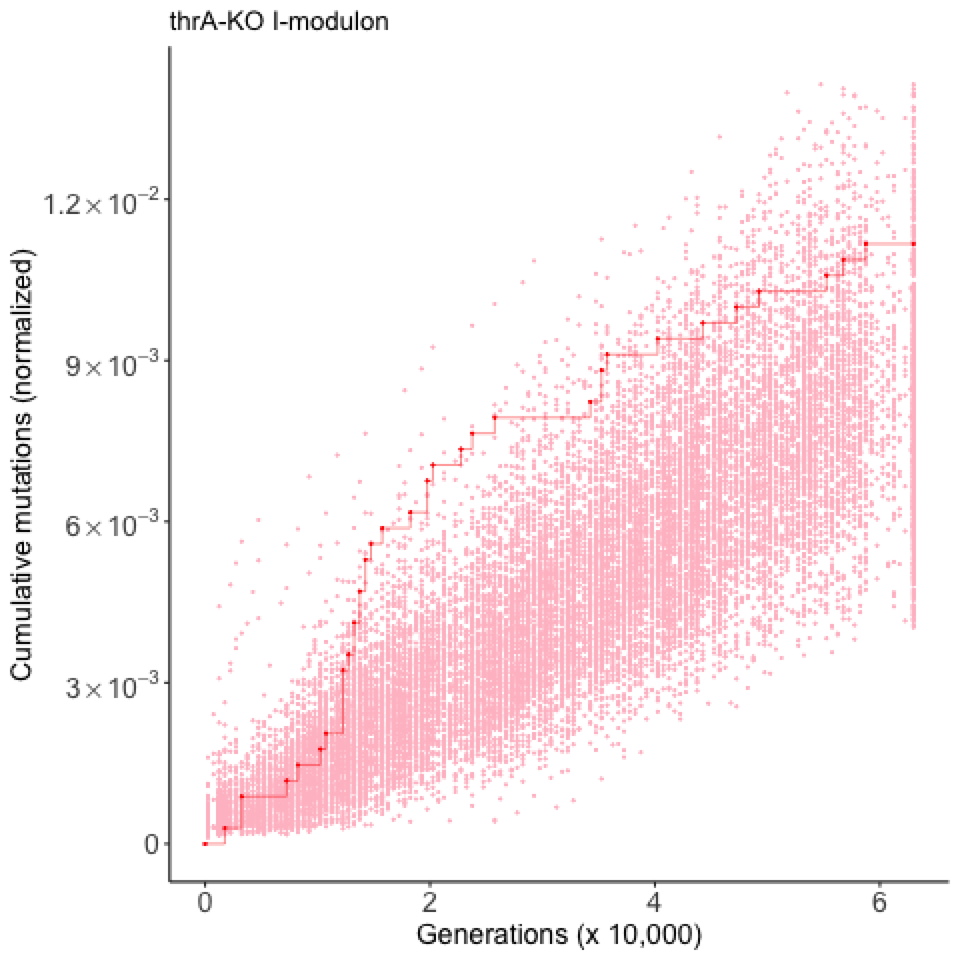
thrA-KO I-modulon
Cumulative mutations (normalized)
1.2×10^{-2}
9×10^{-3}
6×10^{-3}
3×10^{-3}
0
0
2
4
6
Generations (x 10,000)

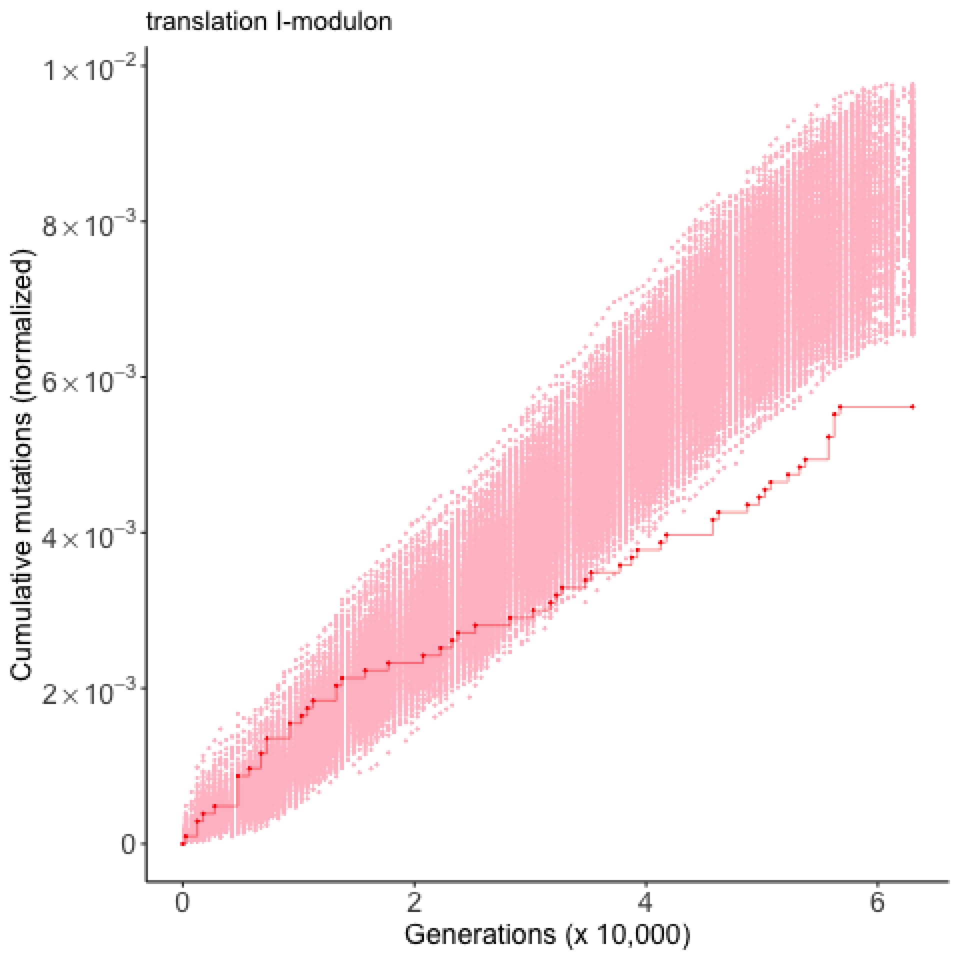

translation I-modulon
Cumulative mutations (normalized)
1×10^−2
8×10^−3
6×10^−3
4×10^−3
2×10^−3
0
0
2
4
6
Generations (x 10,000)

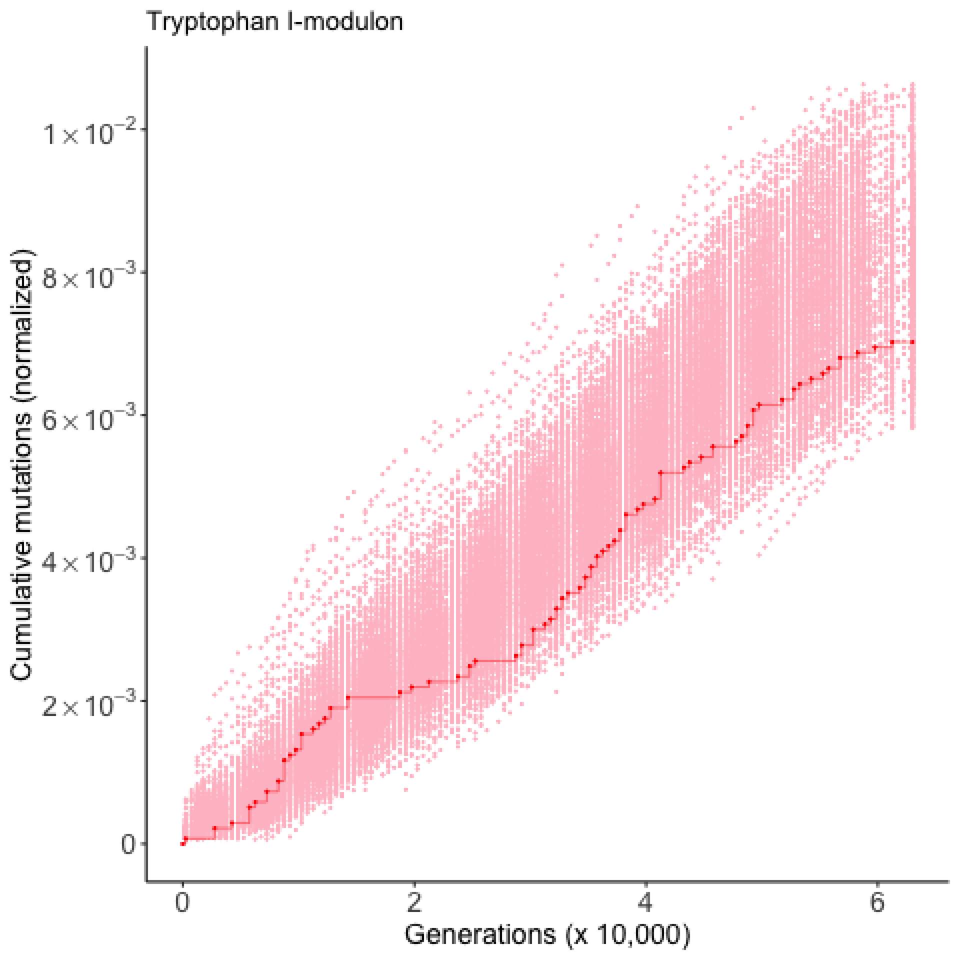

Tryptophan I-modulon
Cumulative mutations (normalized)
1×10⁻²
8×10⁻³
6×10⁻³
4×10⁻³
2×10⁻³
0
0
2
4
6
Generations (x 10,000)

uncharacterized-1 I-modulon

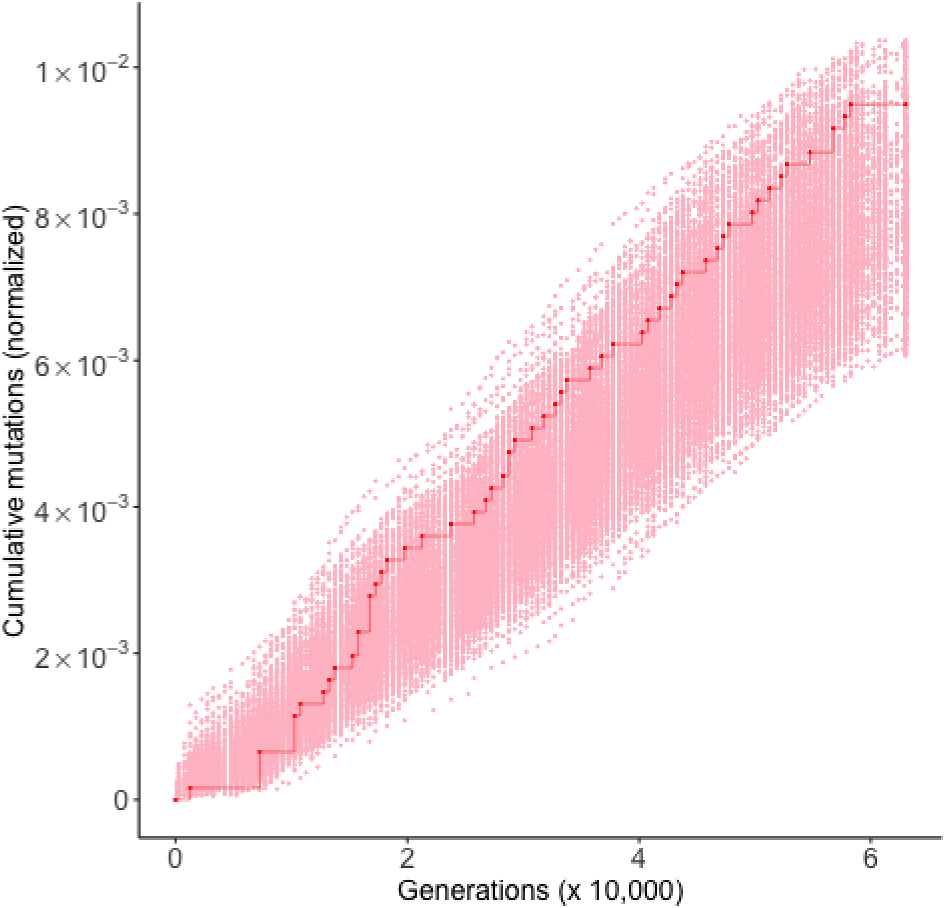

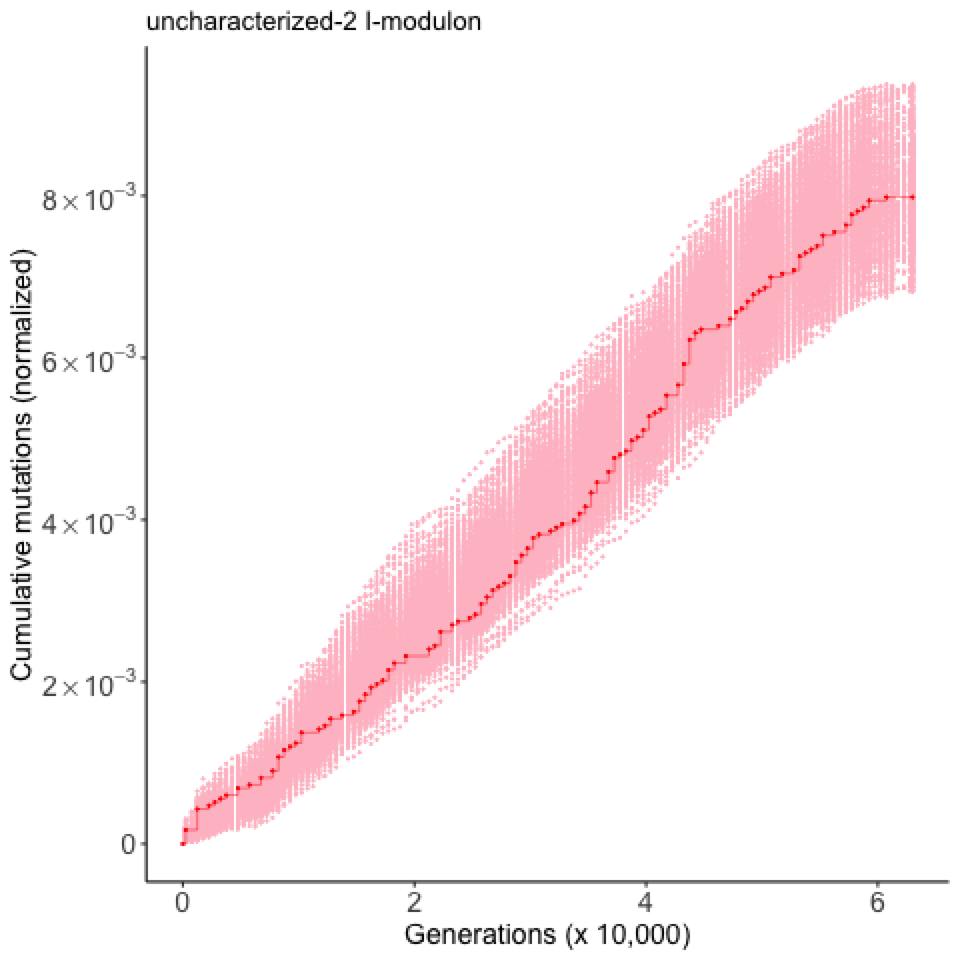
uncharacterized-2 I-modulon
Cumulative mutations (normalized)
8×10^{-3}
6×10^{-3}
4×10^{-3}
2×10^{-3}
0
0
2
4
6
Generations (x 10,000)

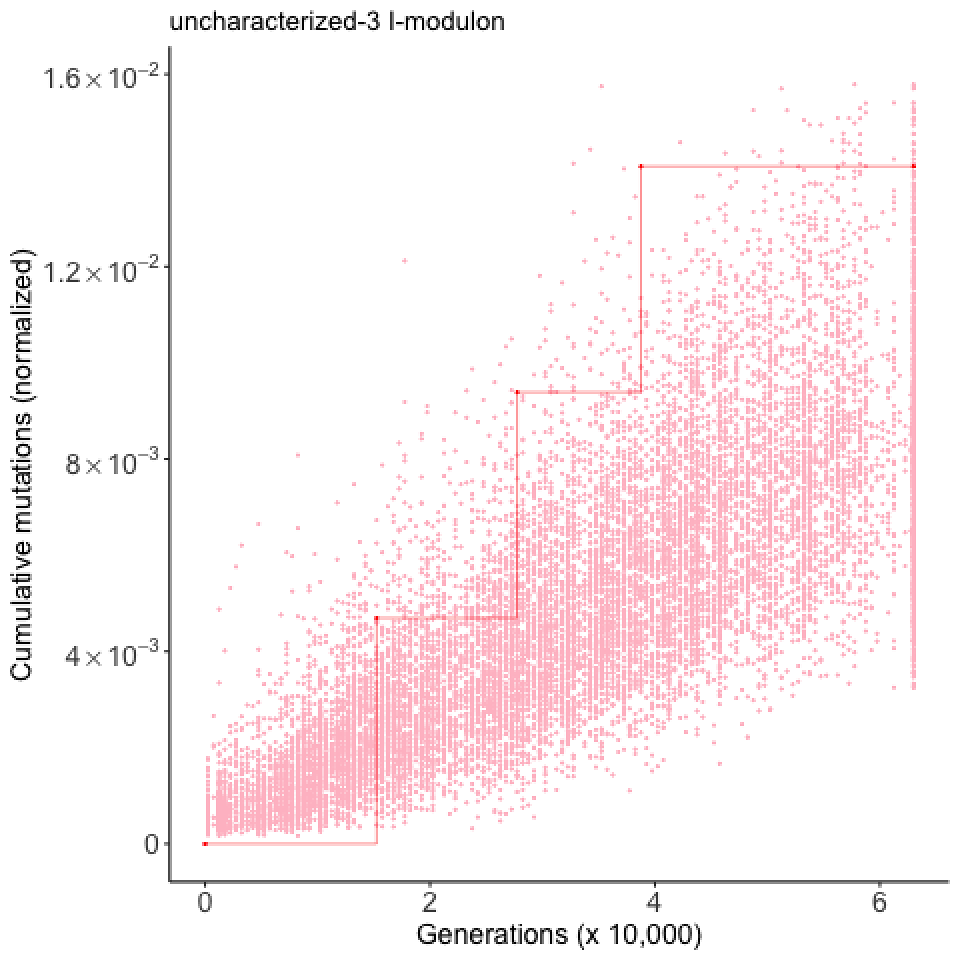

uncharacterized-3 I-modulon
Cumulative mutations (normalized)
1.6×10^{-2}
1.2×10^{-2}
8×10^{-3}
4×10^{-3}
0
0
2
4
6
Generations (x 10,000)

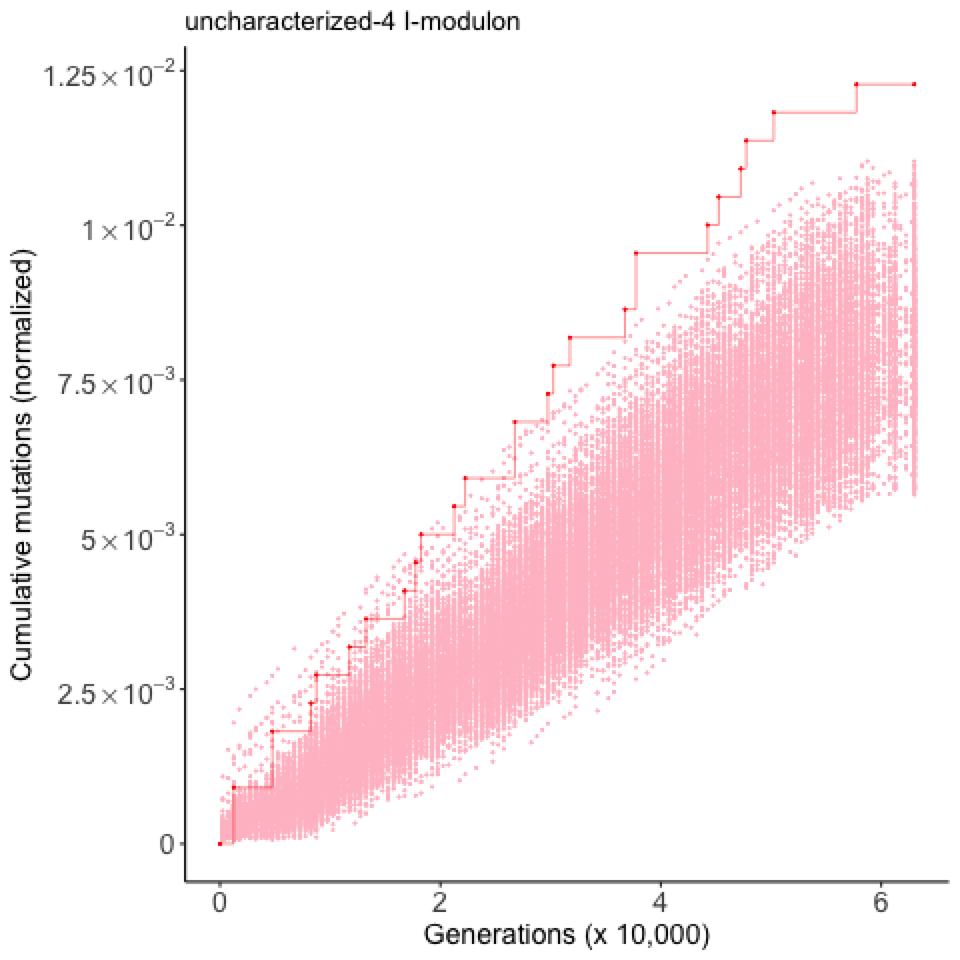

uncharacterized-4 I-modulon
Cumulative mutations (normalized)
1.25×10^-2
1×10^-2
7.5×10^-3
5×10^-3
2.5×10^-3
0
0
2
4
6
Generations (x 10,000)

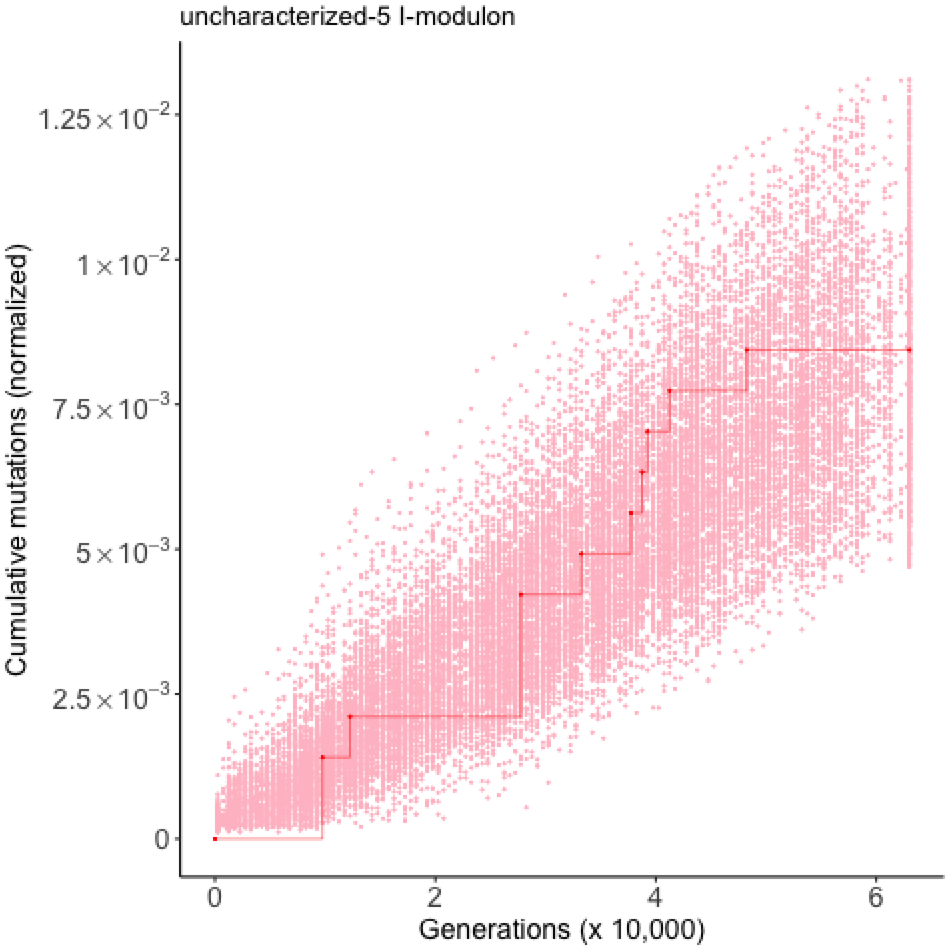
uncharacterized-5 I-modulon
Cumulative mutations (normalized)
1.25×10^{-2}
1×10^{-2}
7.5×10^{-3}
5×10^{-3}
2.5×10^{-3}
0
0
2
4
6
Generations (x 10,000)

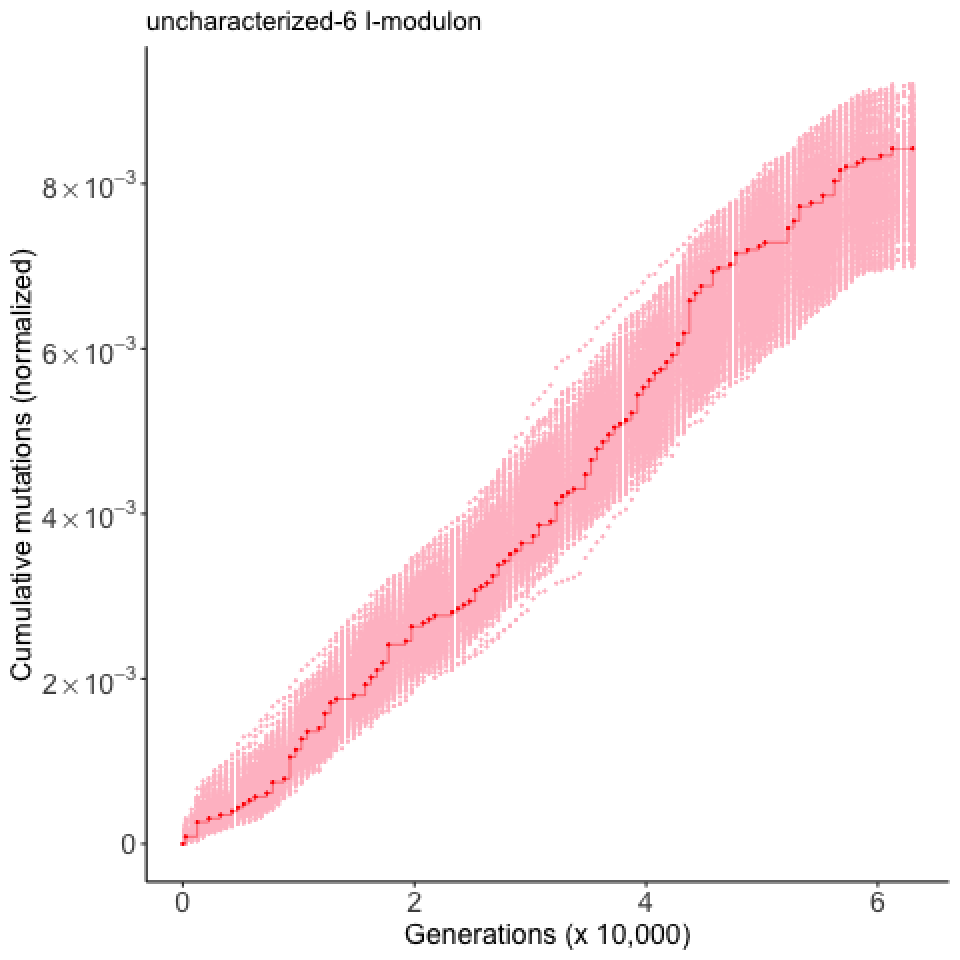
uncharacterized-6 I-modulon
Cumulative mutations (normalized)
8×10^{-3}
6×10^{-3}
4×10^{-3}
2×10^{-3}
0
0
2
4
6
Generations (x 10,000)

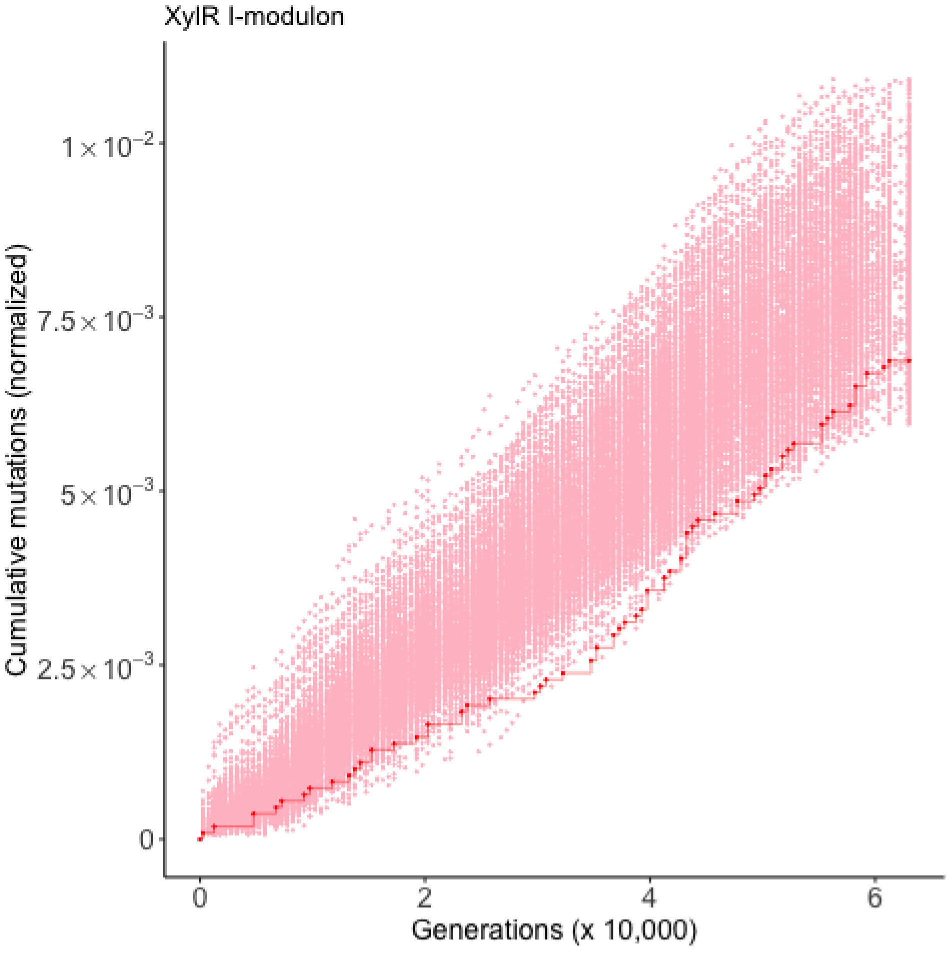

XylR I-modulon
Cumulative mutations (normalized)
1×10^{-2}
7.5×10^{-3}
5×10^{-3}
2.5×10^{-3}
0
0
2
4
6
Generations (x 10,000)

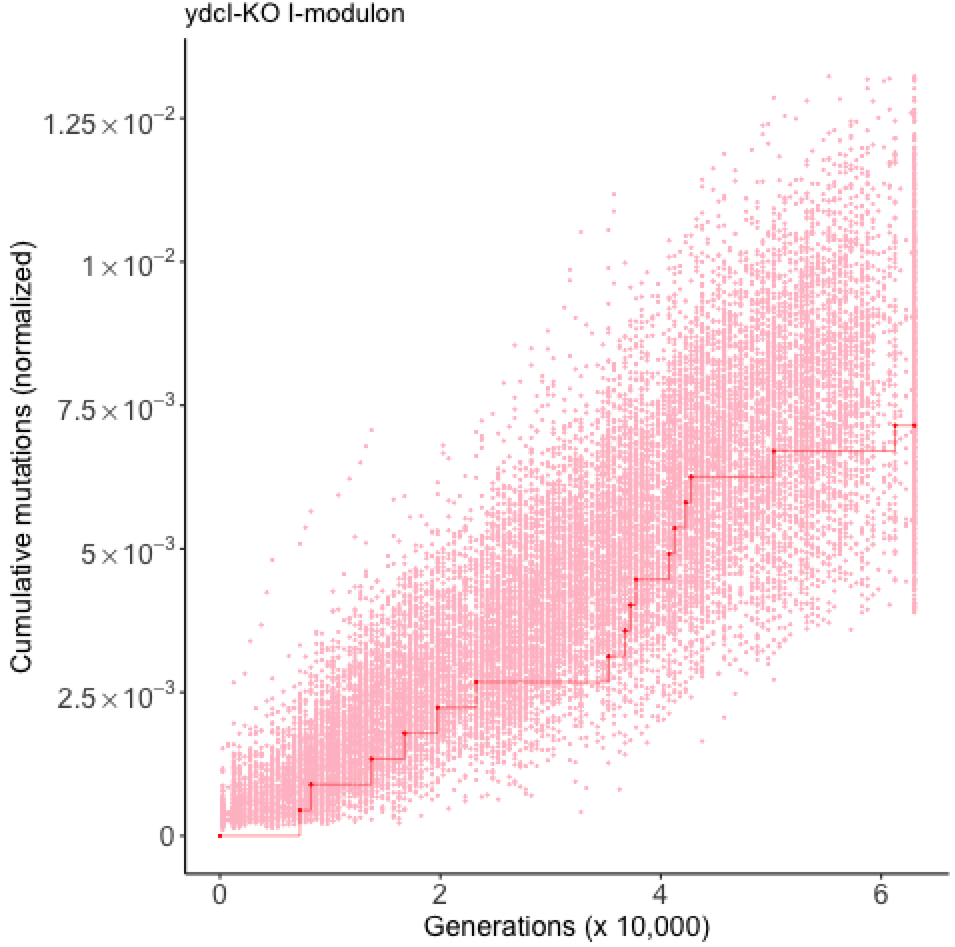

ydcI-KO I-modulon
Cumulative mutations (normalized)
1.25×10^-2
1×10^-2
7.5×10^-3
5×10^-3
2.5×10^-3
0
0
2
4
6
Generations (x 10,000)

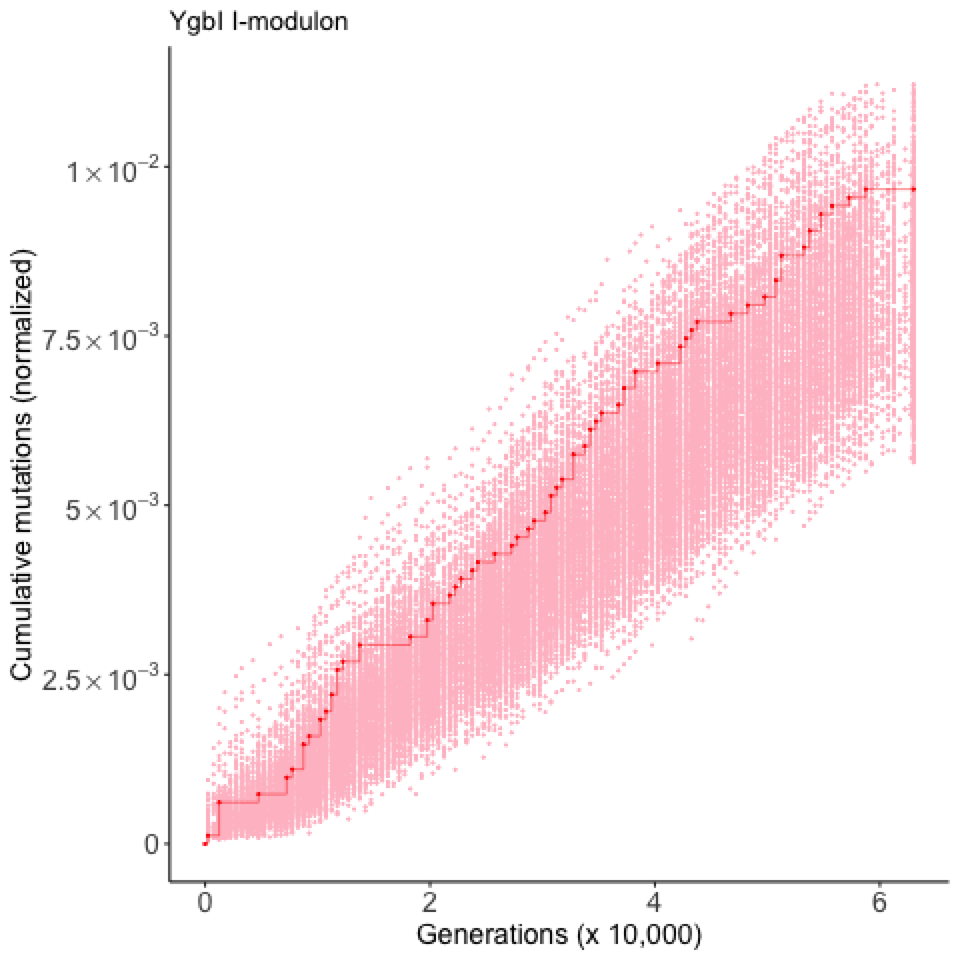

YgbI I-modulon
Cumulative mutations (normalized)
1×10^-2
7.5×10^-3
5×10^-3
2.5×10^-3
0
0
2
4
6
Generations (x 10,000)

yheO-KO I-modulon

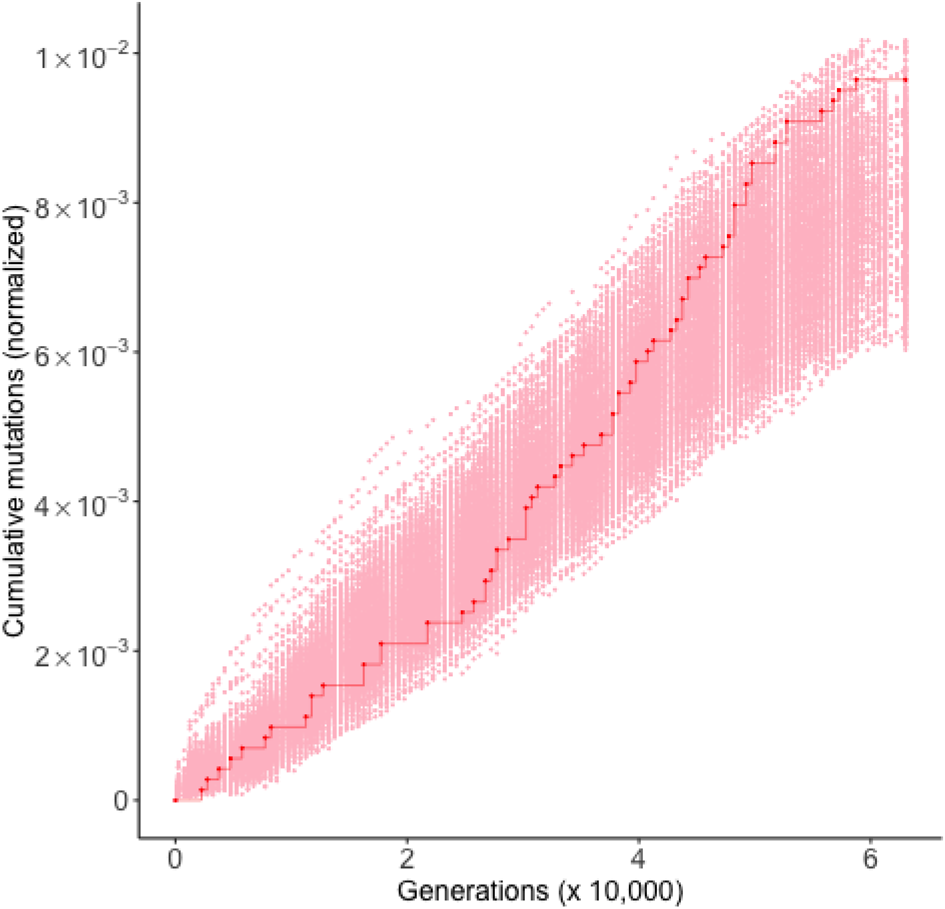

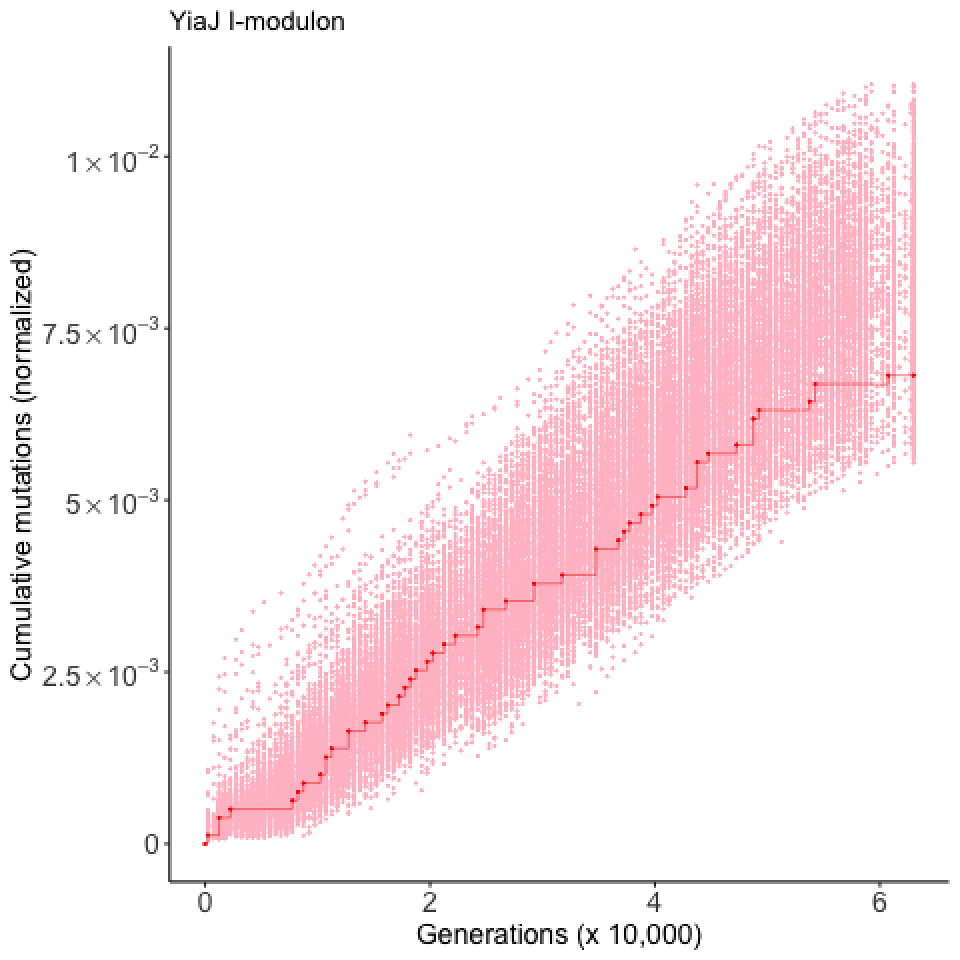
YiaJ I-modulon
Cumulative mutations (normalized)
1×10^{-2}
7.5×10^{-3}
5×10^{-3}
2.5×10^{-3}
0
0
2
4
6
Generations (x 10,000)

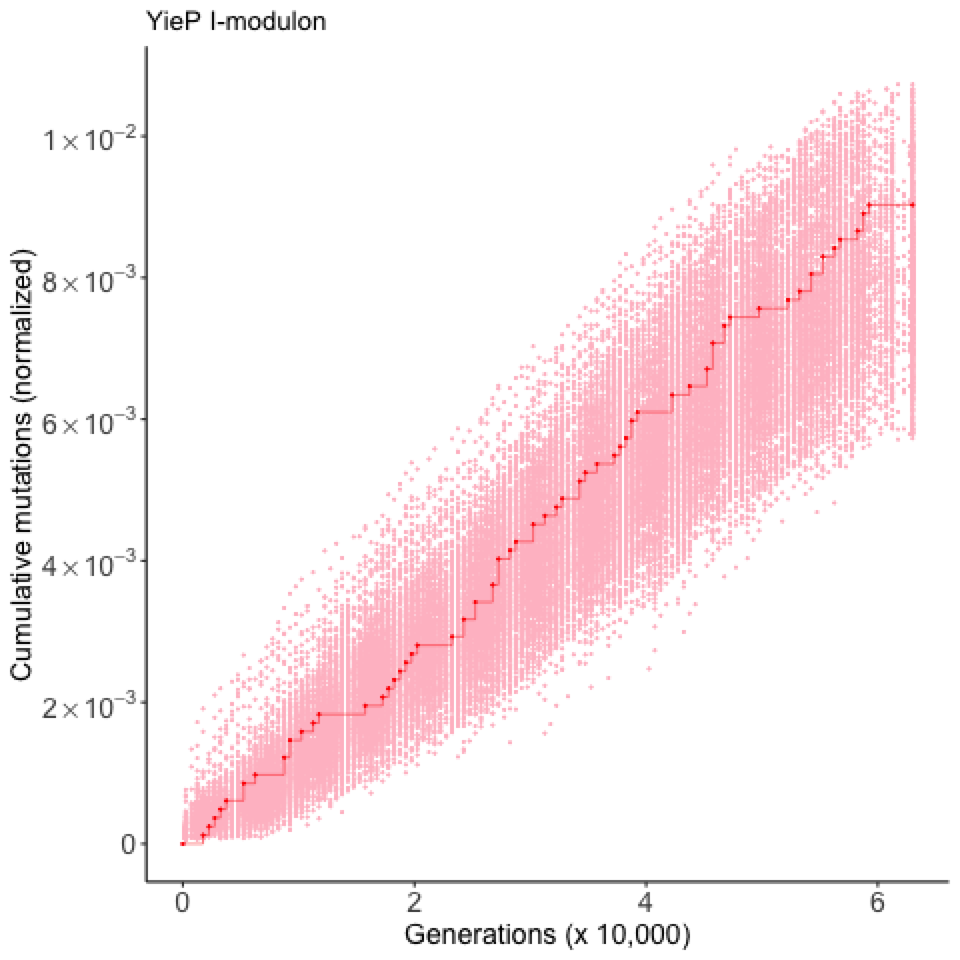
YieP I-modulon
Cumulative mutations (normalized)
1×10⁻²
8×10⁻³
6×10⁻³
4×10⁻³
2×10⁻³
0
0
2
4
6
Generations (x 10,000)

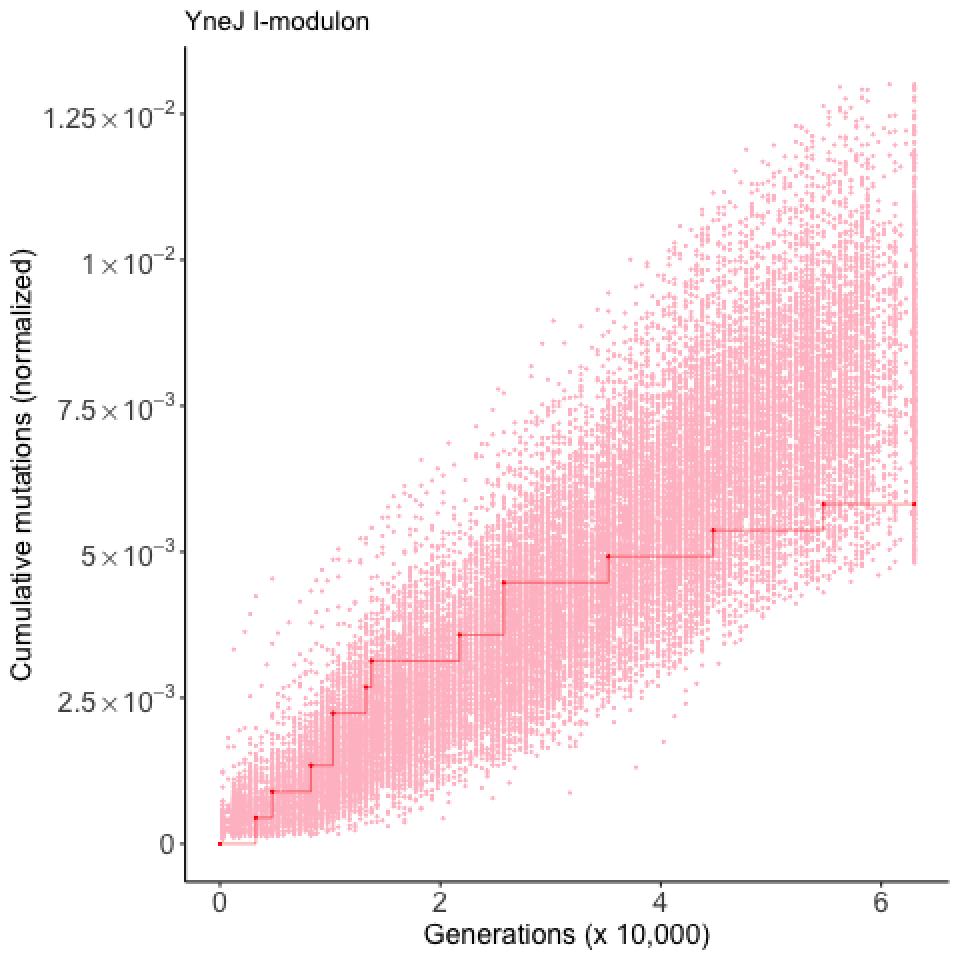
YneJ I-modulon
Cumulative mutations (normalized)
1.25×10^{-2}
1×10^{-2}
7.5×10^{-3}
5×10^{-3}
2.5×10^{-3}
0
0
2
4
6
Generations (x 10,000)

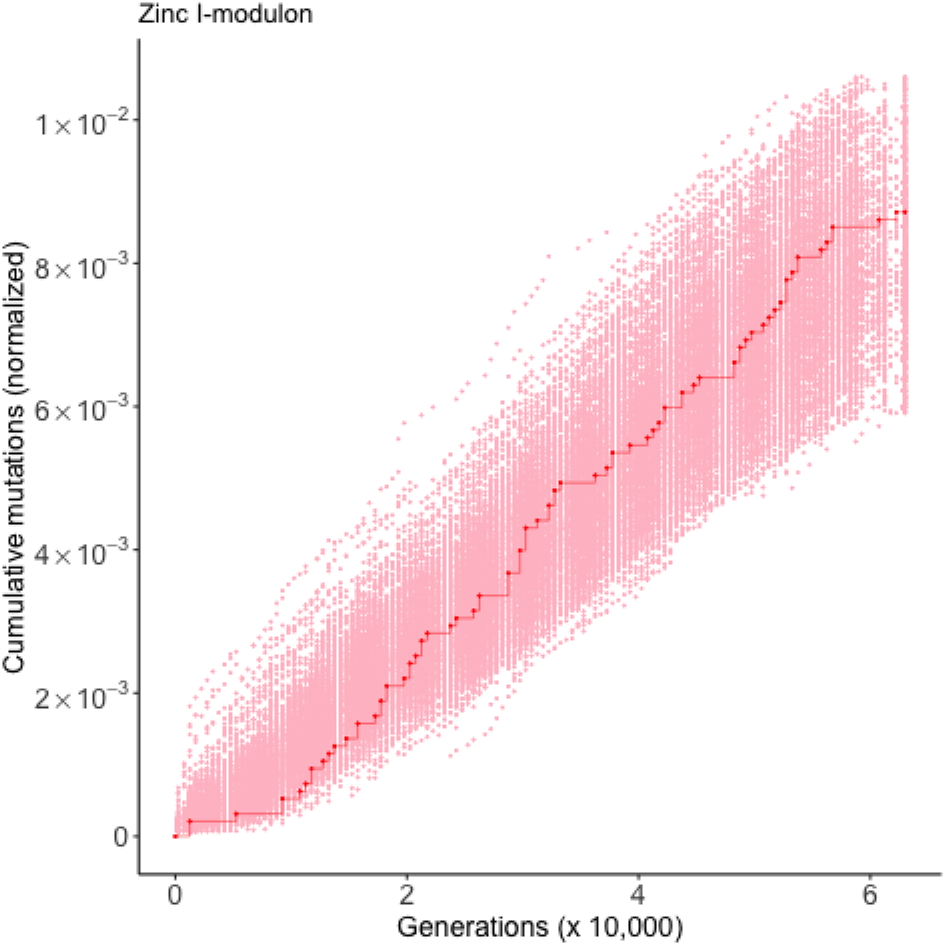
Zinc I-modulon
Cumulative mutations (normalized)
1×10^−2
8×10^−3
6×10^−3
4×10^−3
2×10^−3
0
0
2
4
6
Generations (x 10,000)
