# Supplementary material for: Discovery of positive and purifying selection in metagenomic time series of hypermutator microbial populations
Source: PLoS Genet. 2022 Aug 18;18(8):e1010324. doi: 10.1371/journal.pgen.1010324 (PMC9426924; doi:10.1371/journal.pgen.1010324)

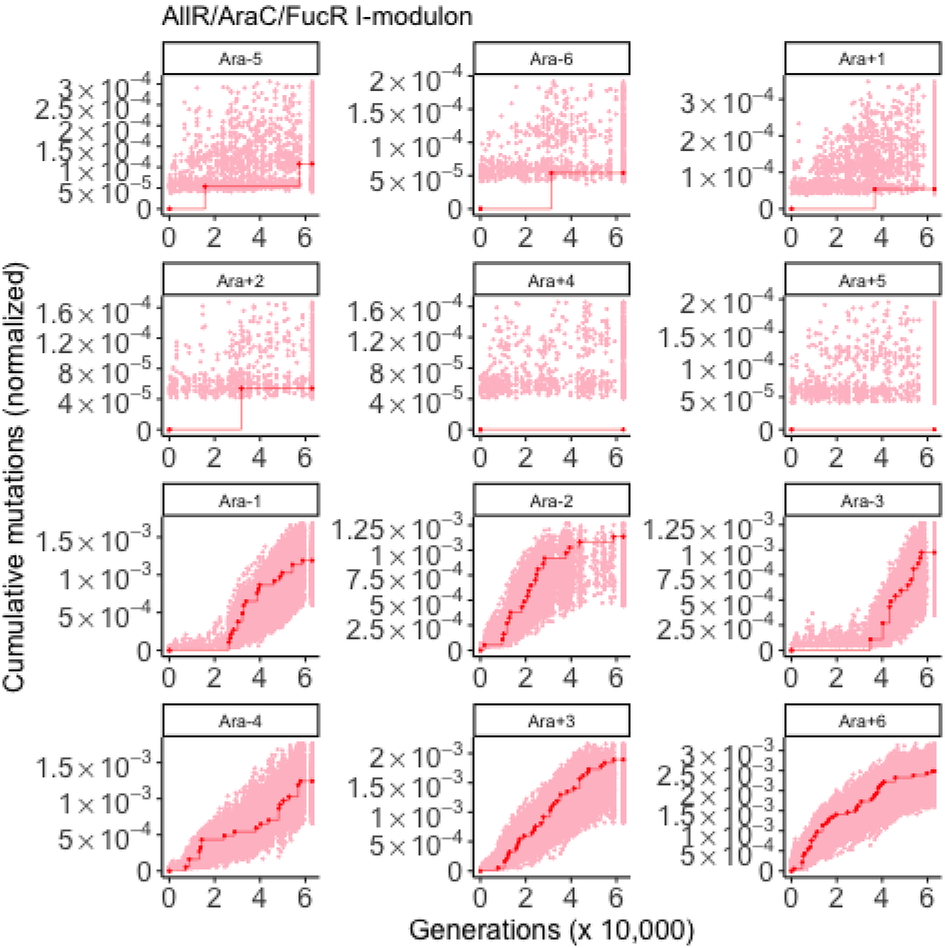

AllR/AraC/FucR I-modulon
Ara-5
Ara-6
Ara+1
Ara+2
Ara+4
Ara+5
Ara-1
Ara-2
Ara-3
Ara-4
Ara+3
Ara+6
Cumulative mutations (normalized)
Generations (x 10,000)

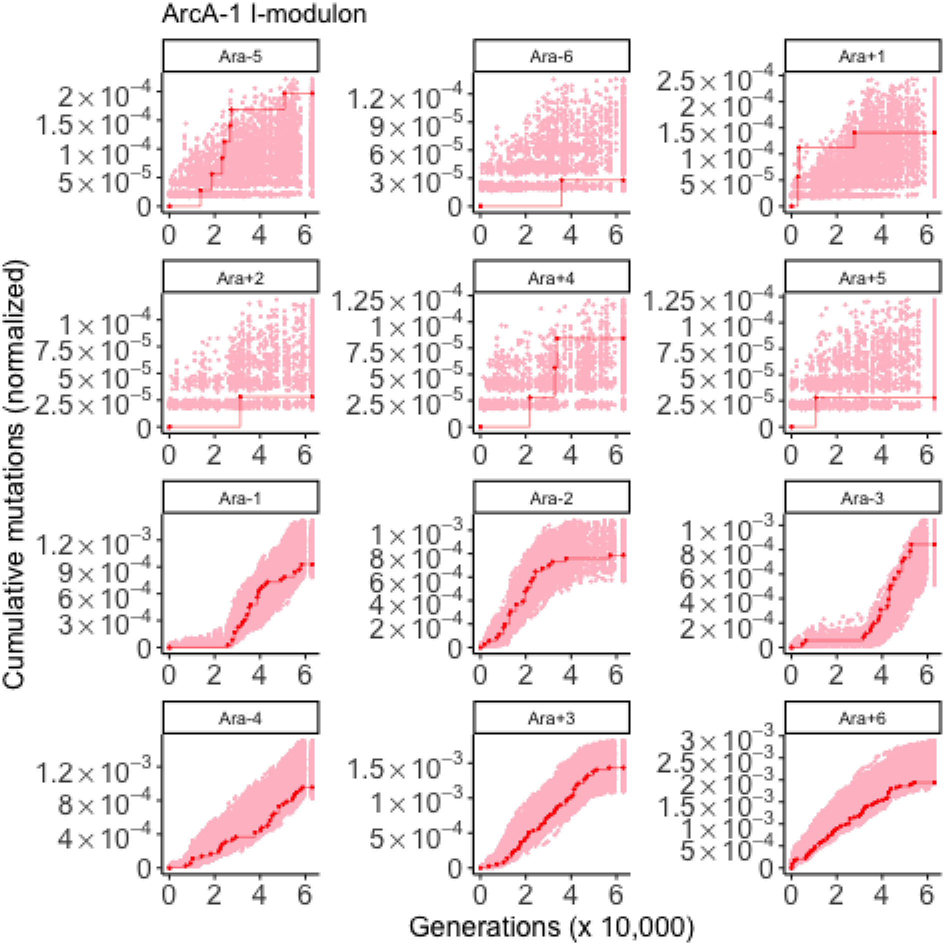
ArcA-1 I-modulon
Ara-5
Ara-6
Ara+1
Ara+2
Ara+4
Ara+5
Ara-1
Ara-2
Ara-3
Ara-4
Ara+3
Ara+6
Cumulative mutations (normalized)
Generations (x 10,000)

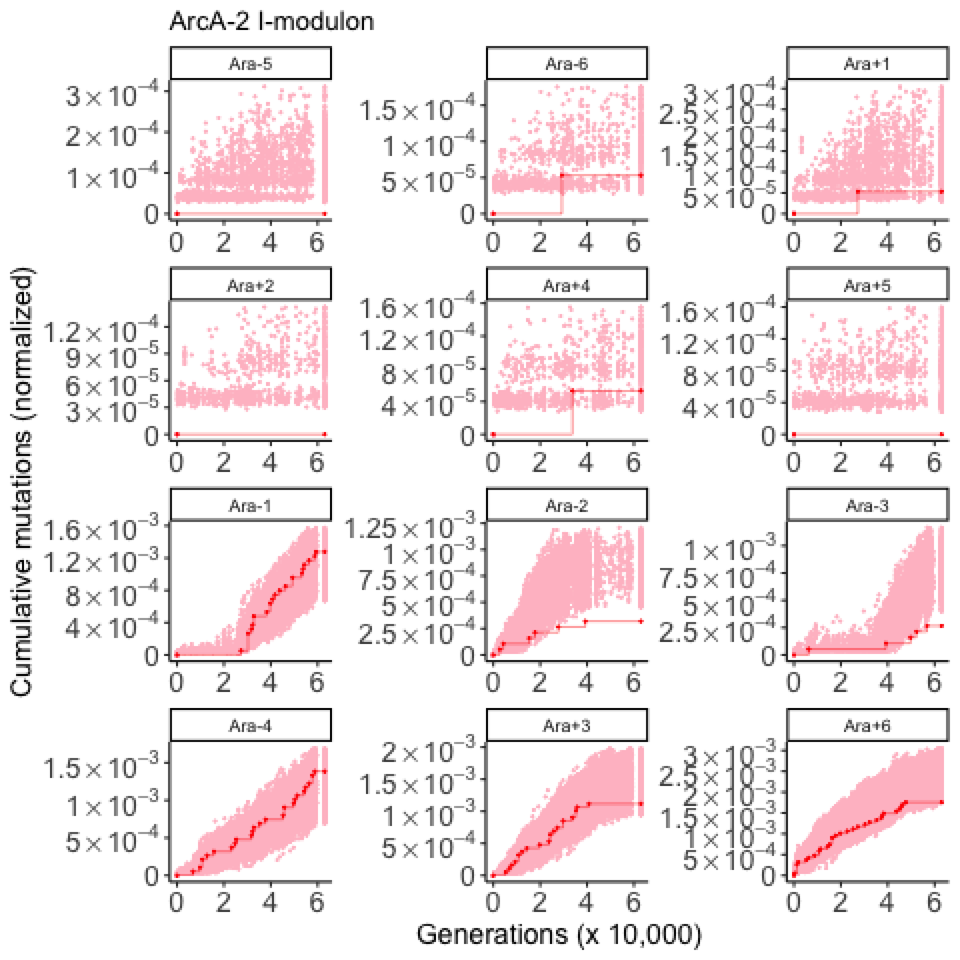

ArcA-2 I-modulon
Ara-5
Ara-6
Ara+1
Ara+2
Ara+4
Ara+5
Ara-1
Ara-2
Ara-3
Ara-4
Ara+3
Ara+6
Cumulative mutations (normalized)
Generations (x 10,000)

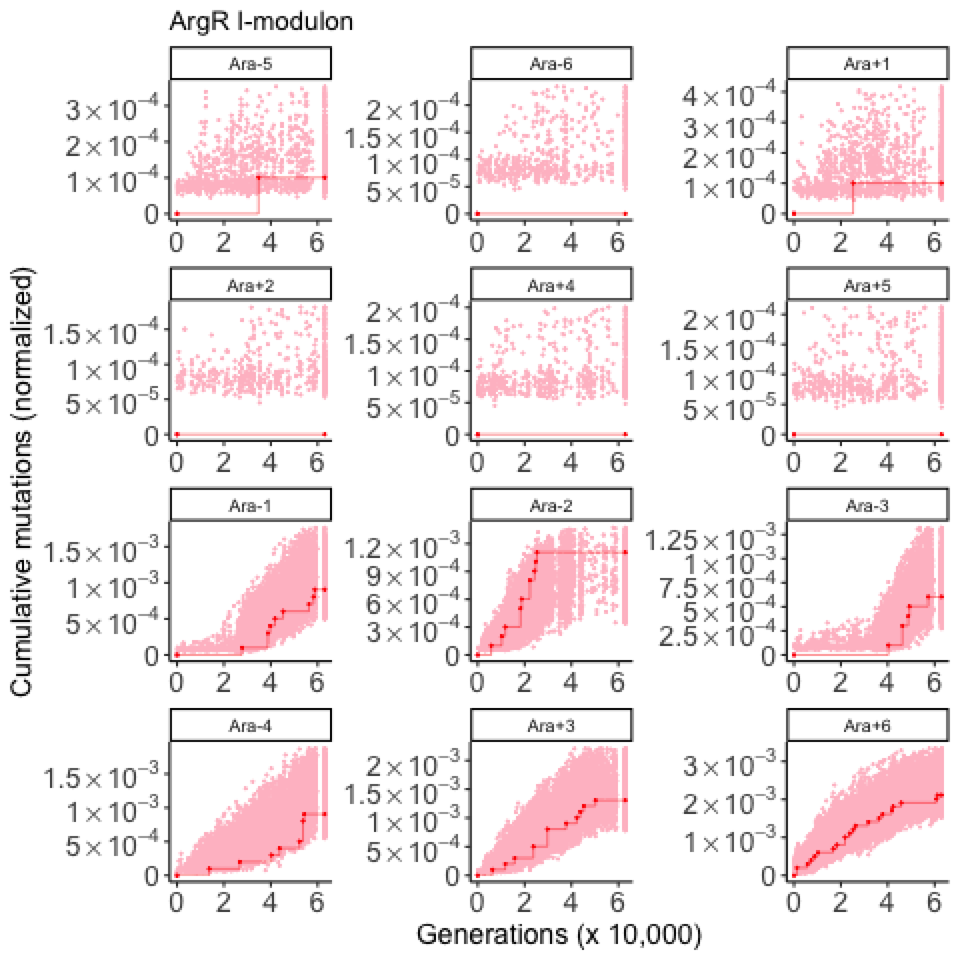

ArgR I-modulon
Ara-5
Ara-6
Ara+1
Ara+2
Ara+4
Ara+5
Ara-1
Ara-2
Ara-3
Ara-4
Ara+3
Ara+6
Cumulative mutations (normalized)
Generations (x 10,000)

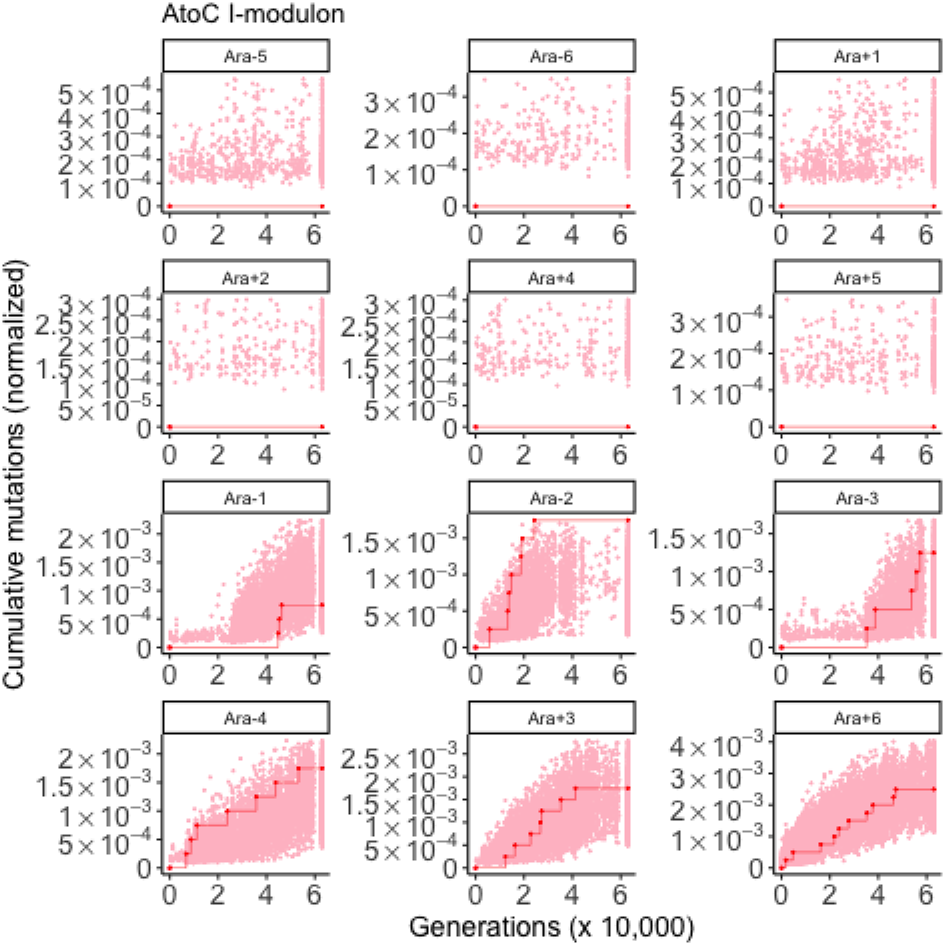

AtoC I-modulon
Ara-5
Ara-6
Ara+1
Ara+2
Ara+4
Ara+5
Ara-1
Ara-2
Ara-3
Ara-4
Ara+3
Ara+6
Cumulative mutations (normalized)
Generations (x 10,000)

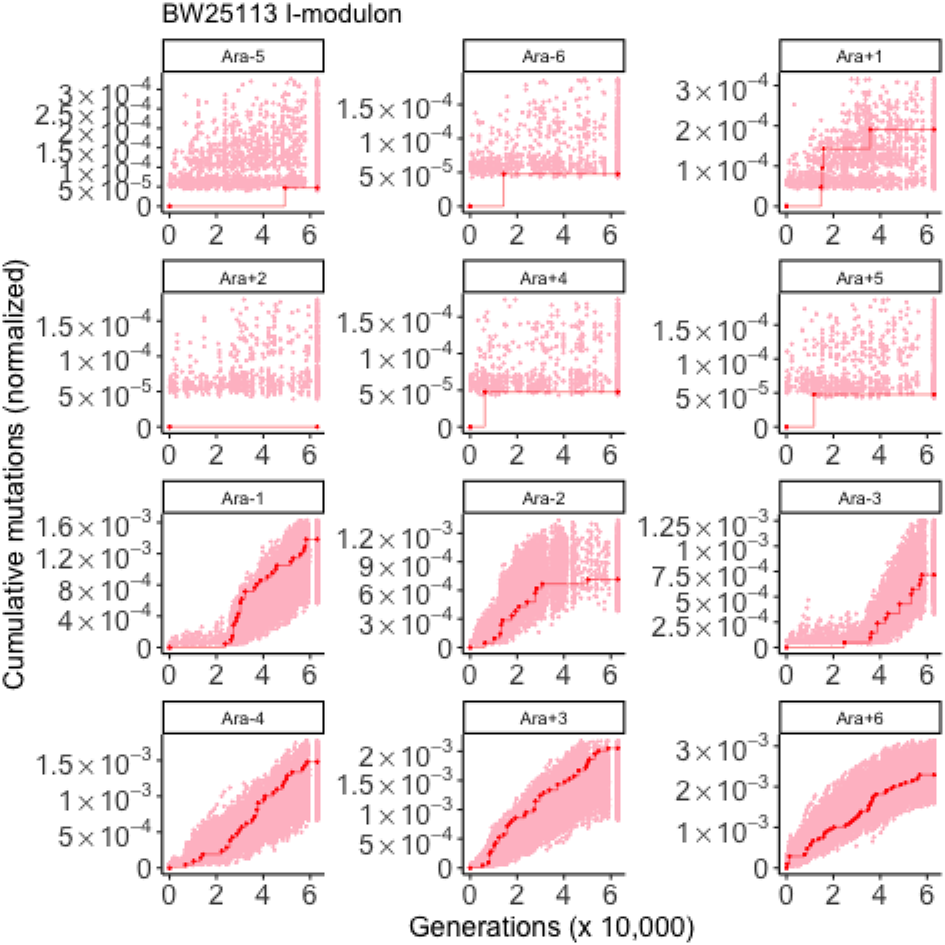

BW25113 I-modulon
Ara-5
Ara-6
Ara+1
Ara+2
Ara+4
Ara+5
Ara-1
Ara-2
Ara-3
Ara-4
Ara+3
Ara+6
Cumulative mutations (normalized)
Generations (x 10,000)

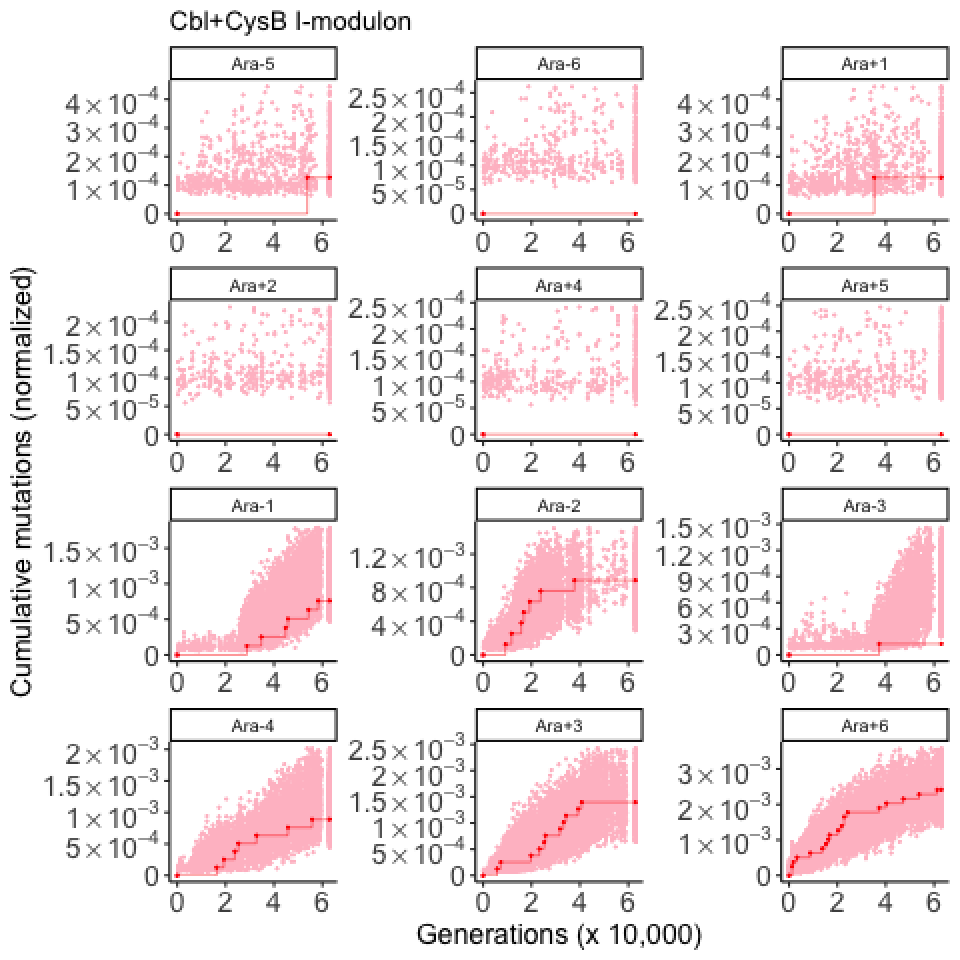

Cbl+CysB I-modulon
Ara-5
Ara-6
Ara+1
Ara+2
Ara+4
Ara+5
Ara-1
Ara-2
Ara-3
Ara-4
Ara+3
Ara+6
Cumulative mutations (normalized)
Generations (x 10,000)

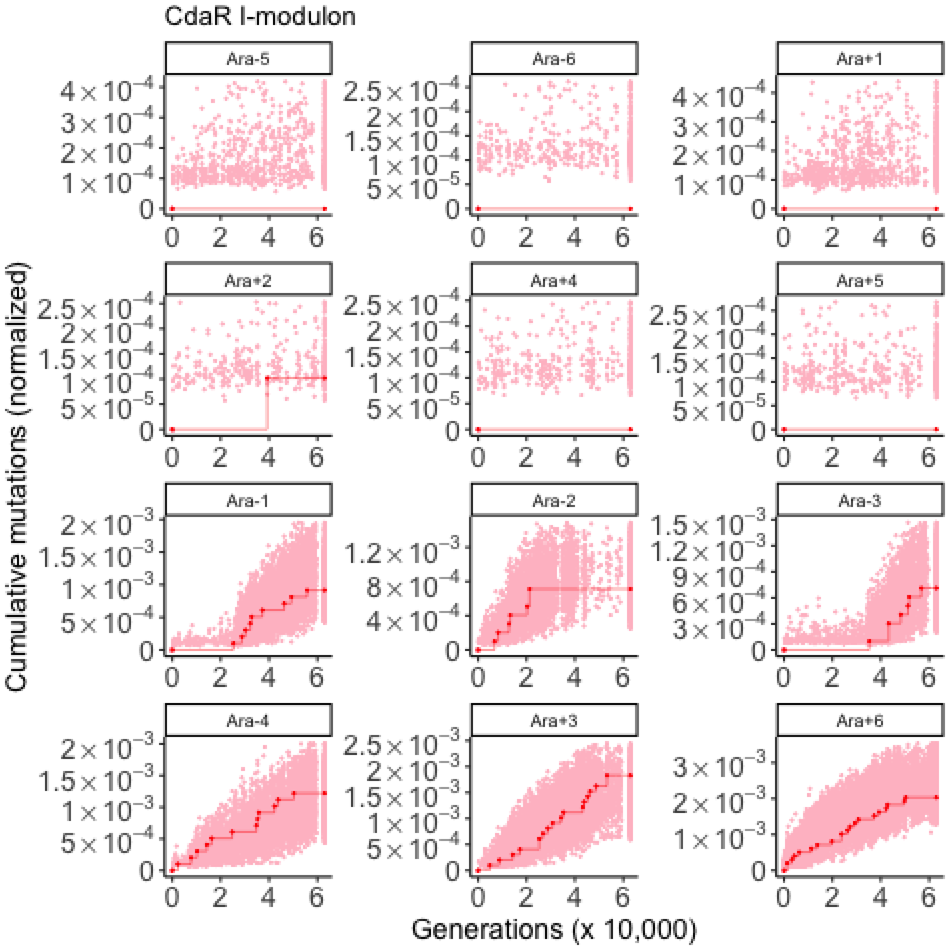
CdaR I-modulon
Ara-5
Ara-6
Ara+1
Ara+2
Ara+4
Ara+5
Ara-1
Ara-2
Ara-3
Ara-4
Ara+3
Ara+6
Cumulative mutations (normalized)
Generations (x 10,000)

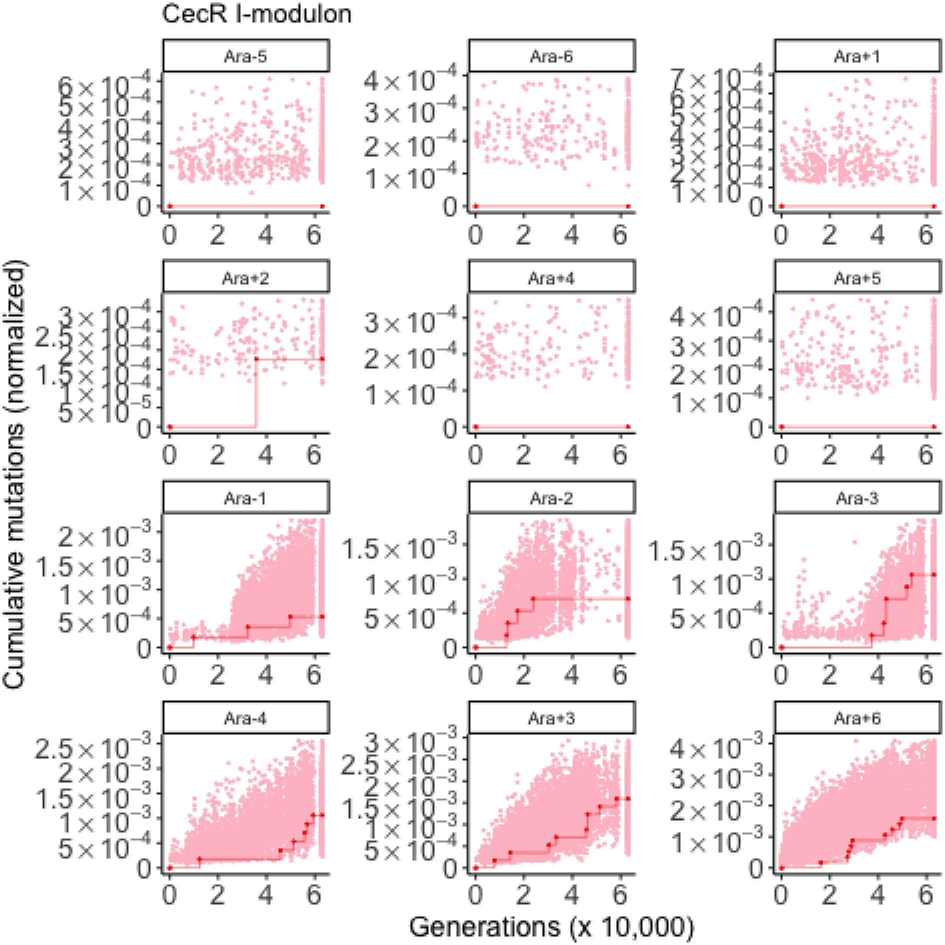

CecR I-modulon
Ara-5
Ara-6
Ara+1
Ara+2
Ara+4
Ara+5
Ara-1
Ara-2
Ara-3
Ara-4
Ara+3
Ara+6
Cumulative mutations (normalized)
Generations (x 10,000)

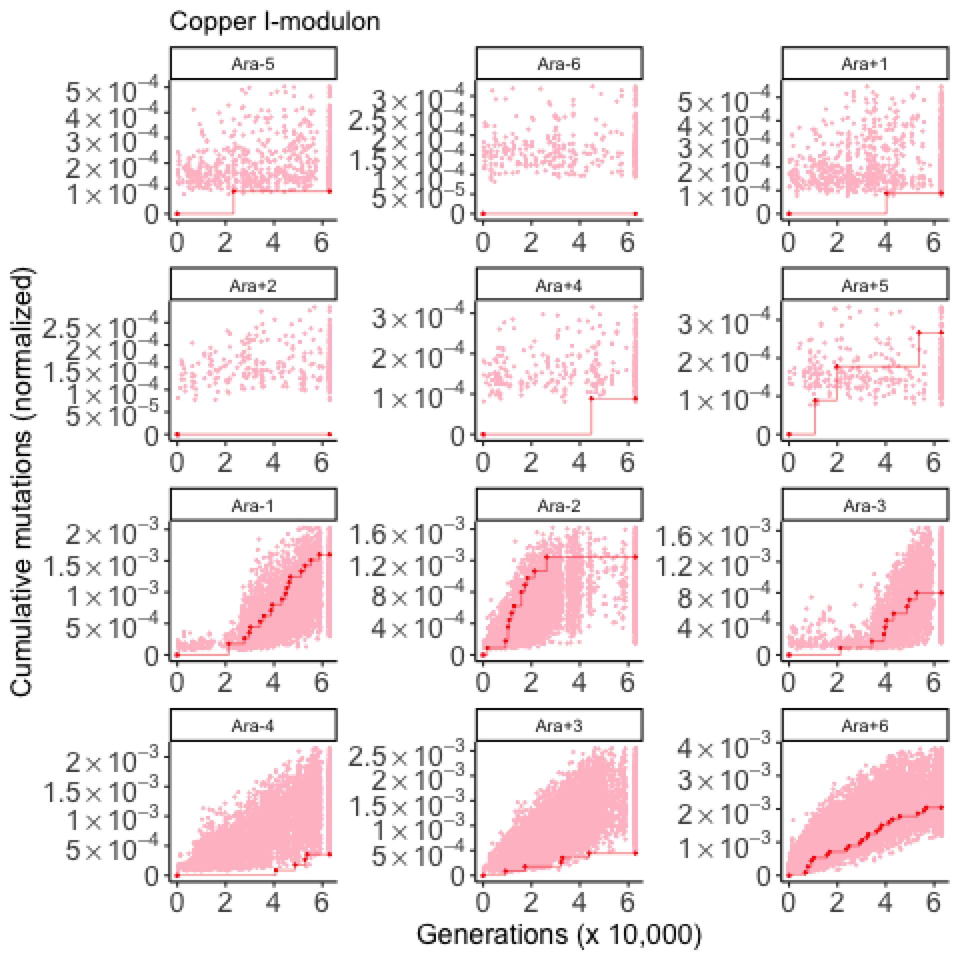

Copper I-modulon
Ara-5
Ara-6
Ara+1
Ara+2
Ara+4
Ara+5
Ara-1
Ara-2
Ara-3
Ara-4
Ara+3
Ara+6
Cumulative mutations (normalized)
Generations (x 10,000)

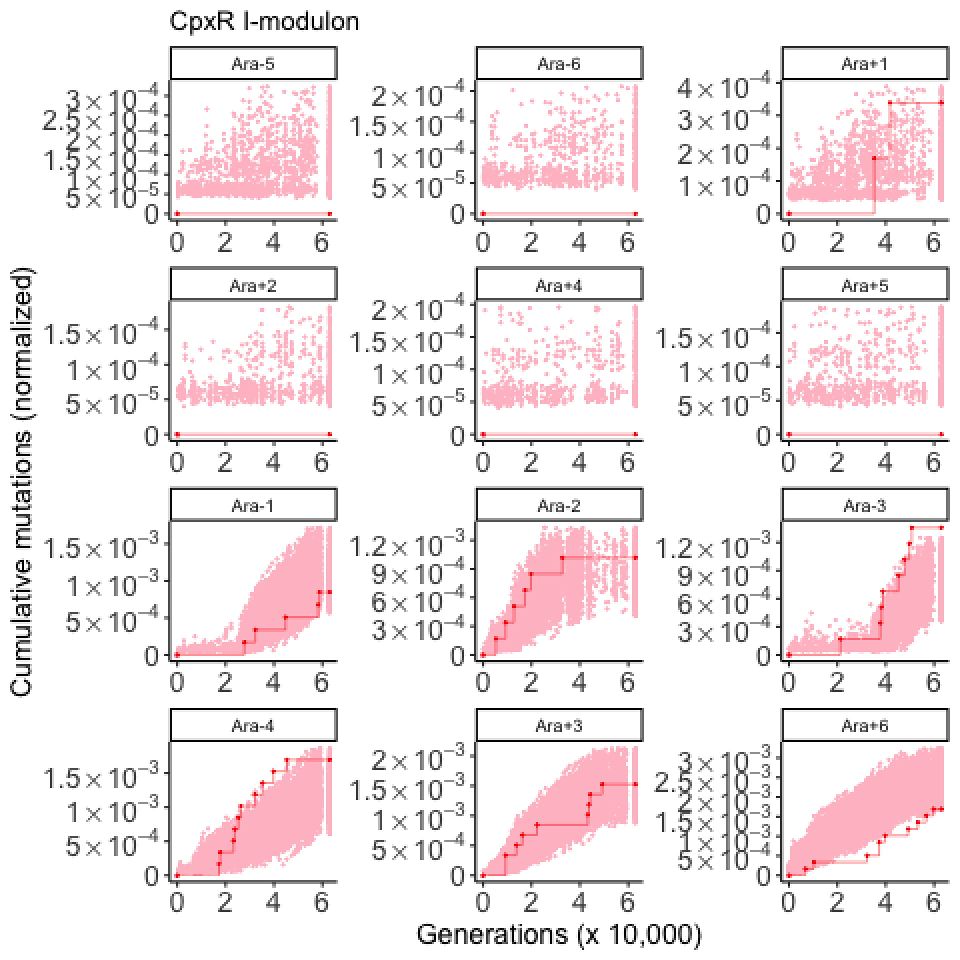
CpxR I-modulon
Ara-5
Ara-6
Ara+1
Ara+2
Ara+4
Ara+5
Ara-1
Ara-2
Ara-3
Ara-4
Ara+3
Ara+6
Cumulative mutations (normalized)
Generations (x 10,000)

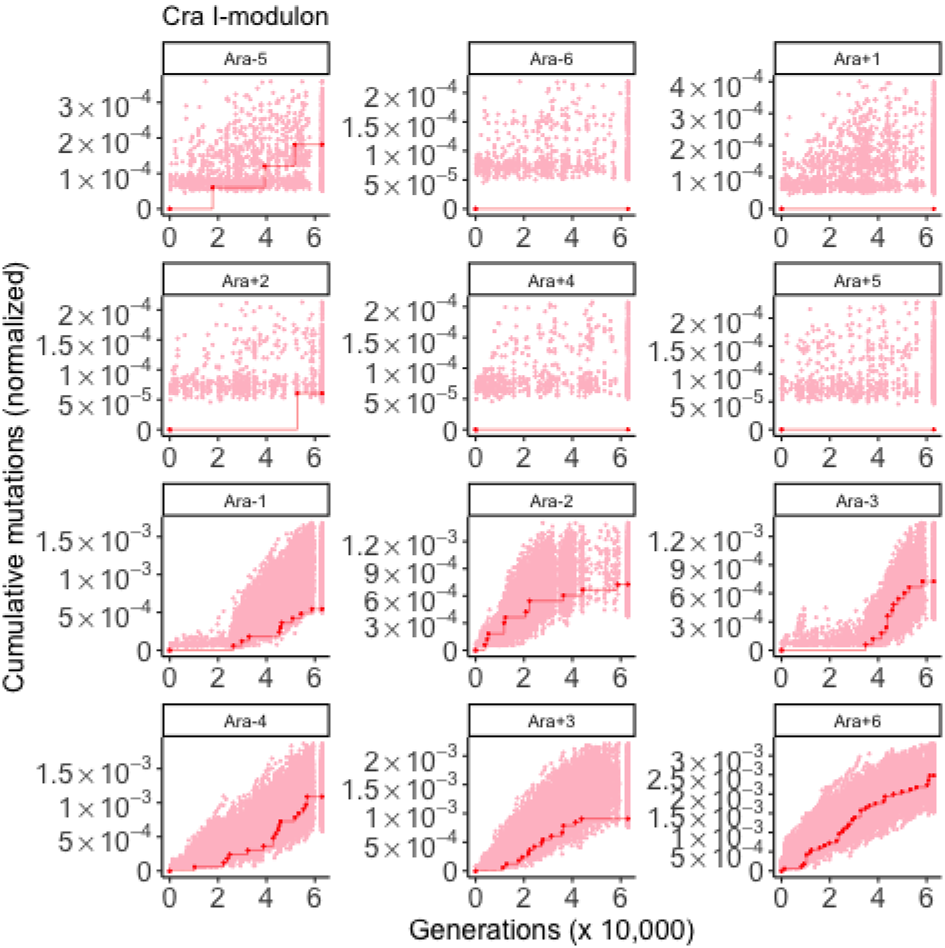
Cra I-modulon
Ara-5
Ara-6
Ara+1
Ara+2
Ara+4
Ara+5
Ara-1
Ara-2
Ara-3
Ara-4
Ara+3
Ara+6
Cumulative mutations (normalized)
Generations (x 10,000)

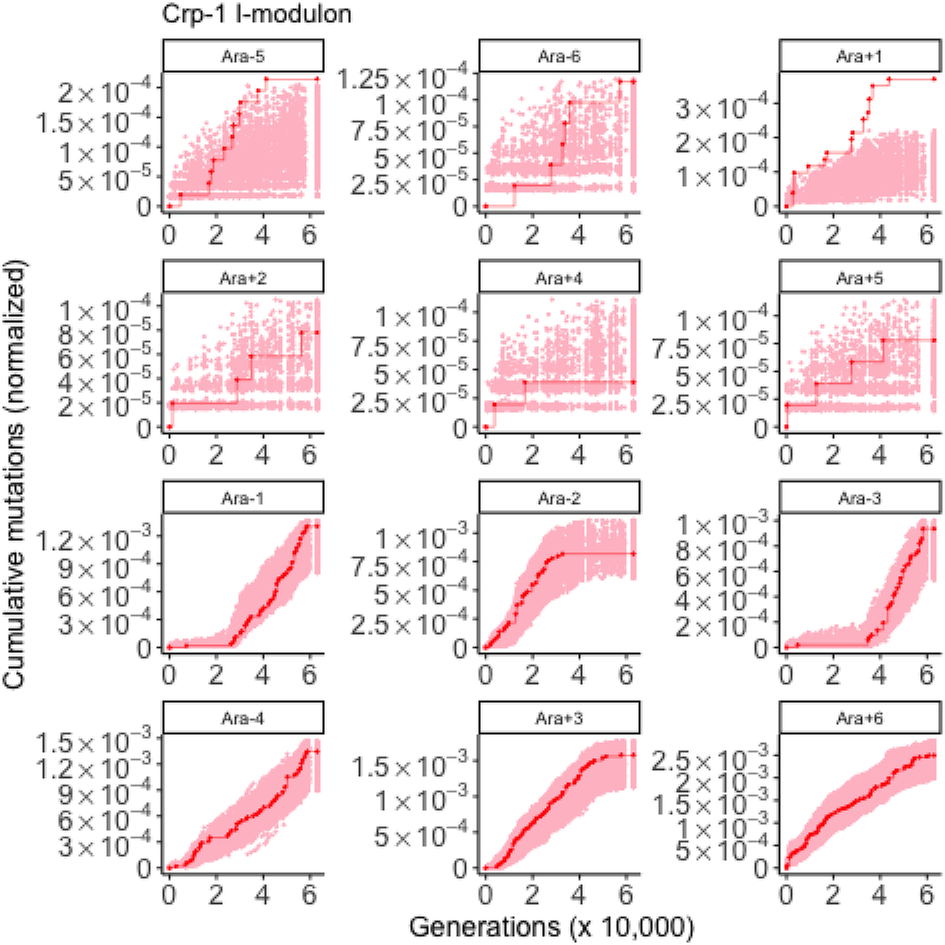

Crp-1 I-modulon
Ara-5
Ara-6
Ara+1
Ara+2
Ara+4
Ara+5
Ara-1
Ara-2
Ara-3
Ara-4
Ara+3
Ara+6
Cumulative mutations (normalized)
Generations (x 10,000)

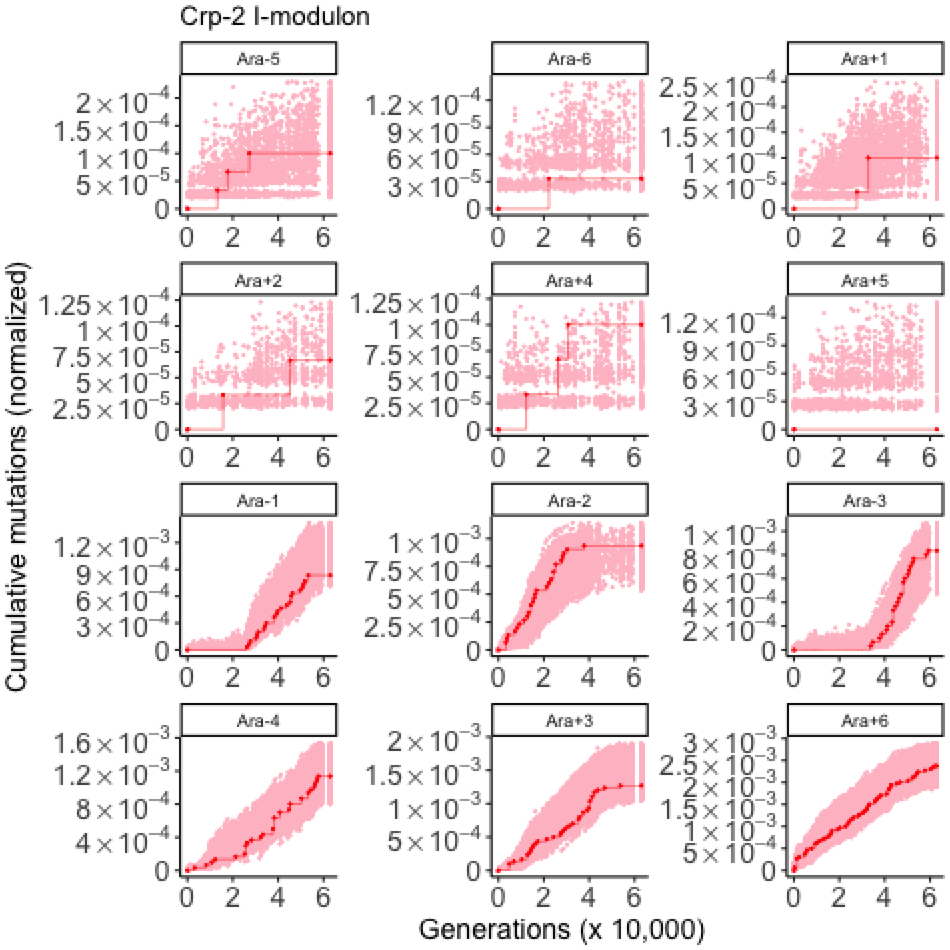

Crp-2 I-modulon
Ara-5
Ara-6
Ara+1
Ara+2
Ara+4
Ara+5
Ara-1
Ara-2
Ara-3
Ara-4
Ara+3
Ara+6
Cumulative mutations (normalized)
Generations (x 10,000)

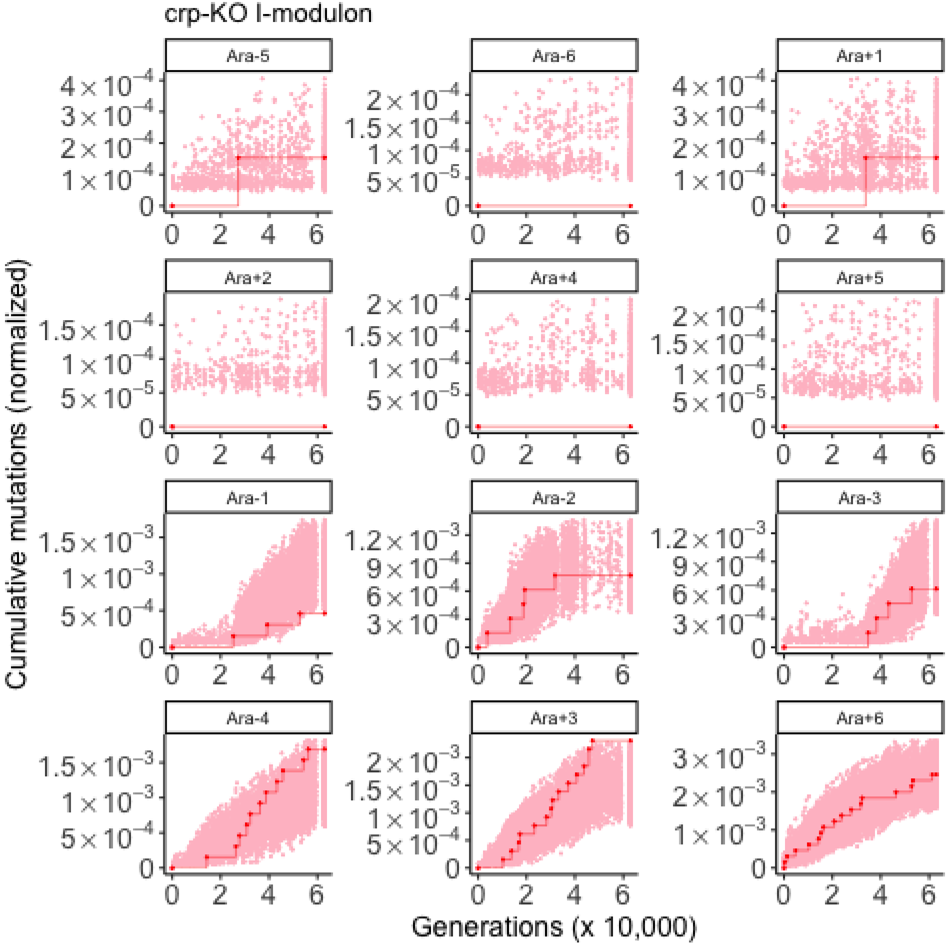

crp-KO I-modulon
Ara-5
Ara-6
Ara+1
Ara+2
Ara+4
Ara+5
Ara-1
Ara-2
Ara-3
Ara-4
Ara+3
Ara+6
Cumulative mutations (normalized)
Generations (x 10,000)

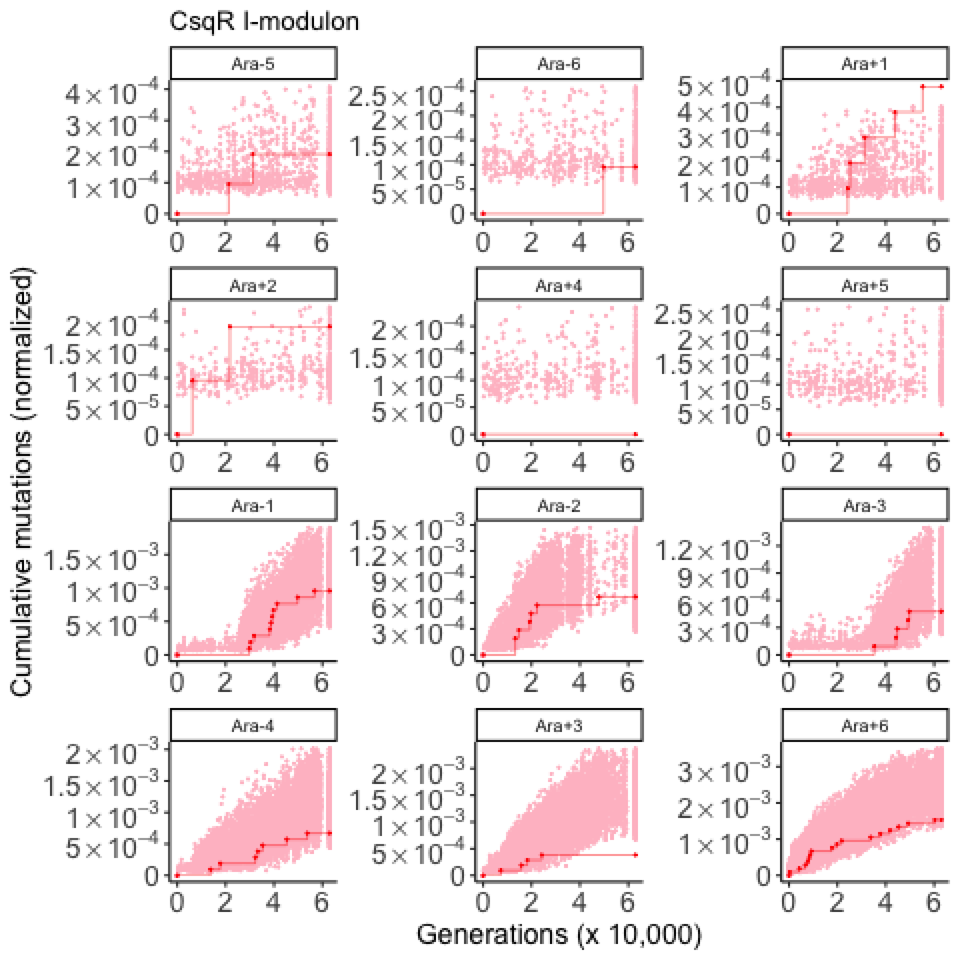
CsqR I-modulon
Ara-5
Ara-6
Ara+1
Ara+2
Ara+4
Ara+5
Ara-1
Ara-2
Ara-3
Ara-4
Ara+3
Ara+6
Cumulative mutations (normalized)
Generations (x 10,000)

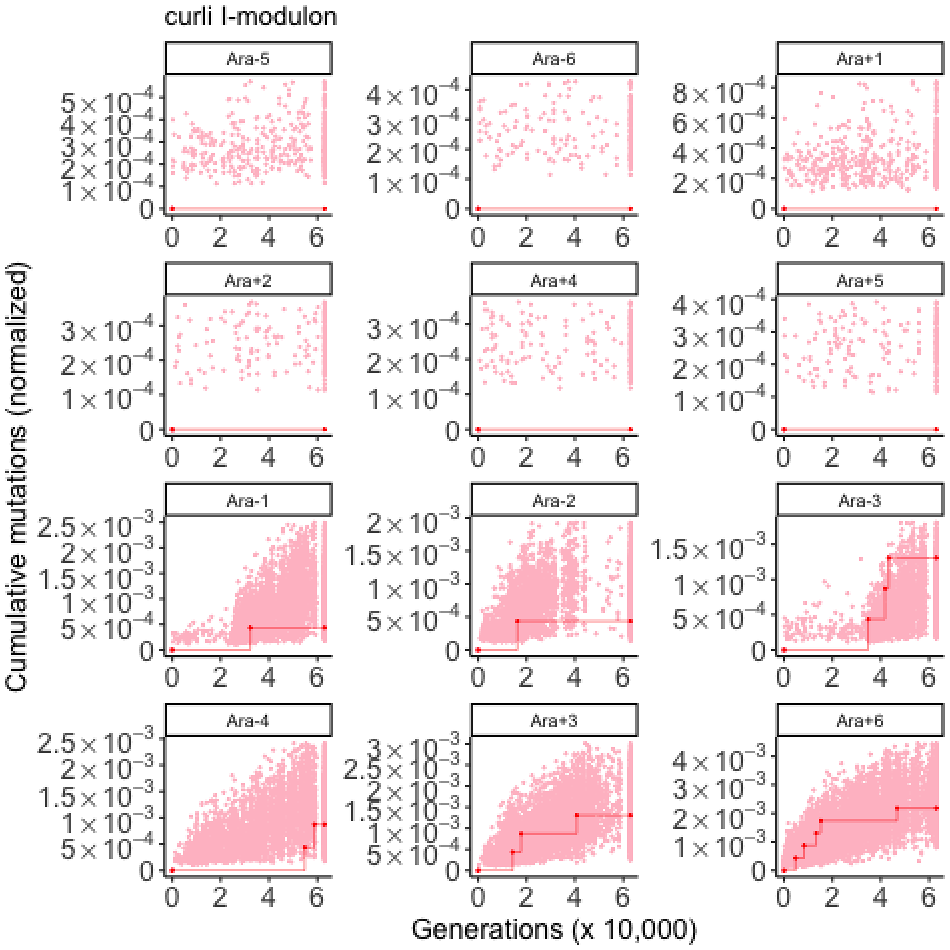
curli I-modulon
Ara-5
Ara-6
Ara+1
Ara+2
Ara+4
Ara+5
Ara-1
Ara-2
Ara-3
Ara-4
Ara+3
Ara+6
Cumulative mutations (normalized)
Generations (x 10,000)

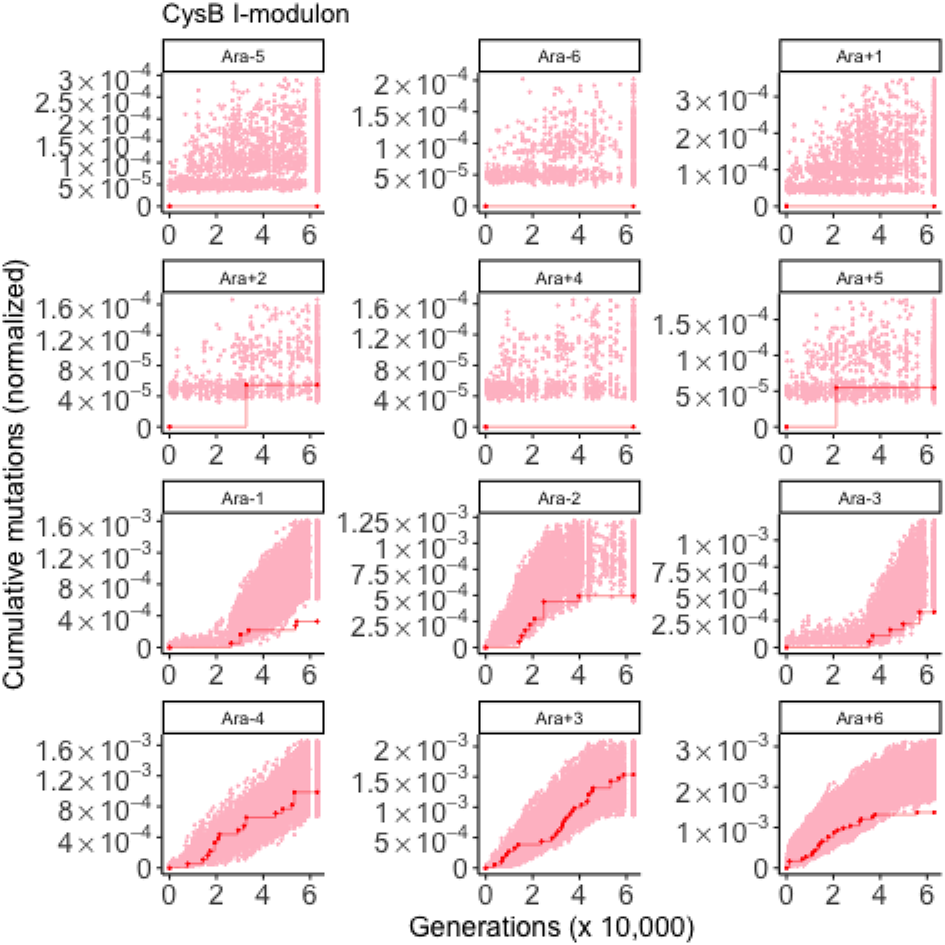

CysB I-modulon
Ara-5
Ara-6
Ara+1
Ara+2
Ara+4
Ara+5
Ara-1
Ara-2
Ara-3
Ara-4
Ara+3
Ara+6
Cumulative mutations (normalized)
Generations (x 10,000)

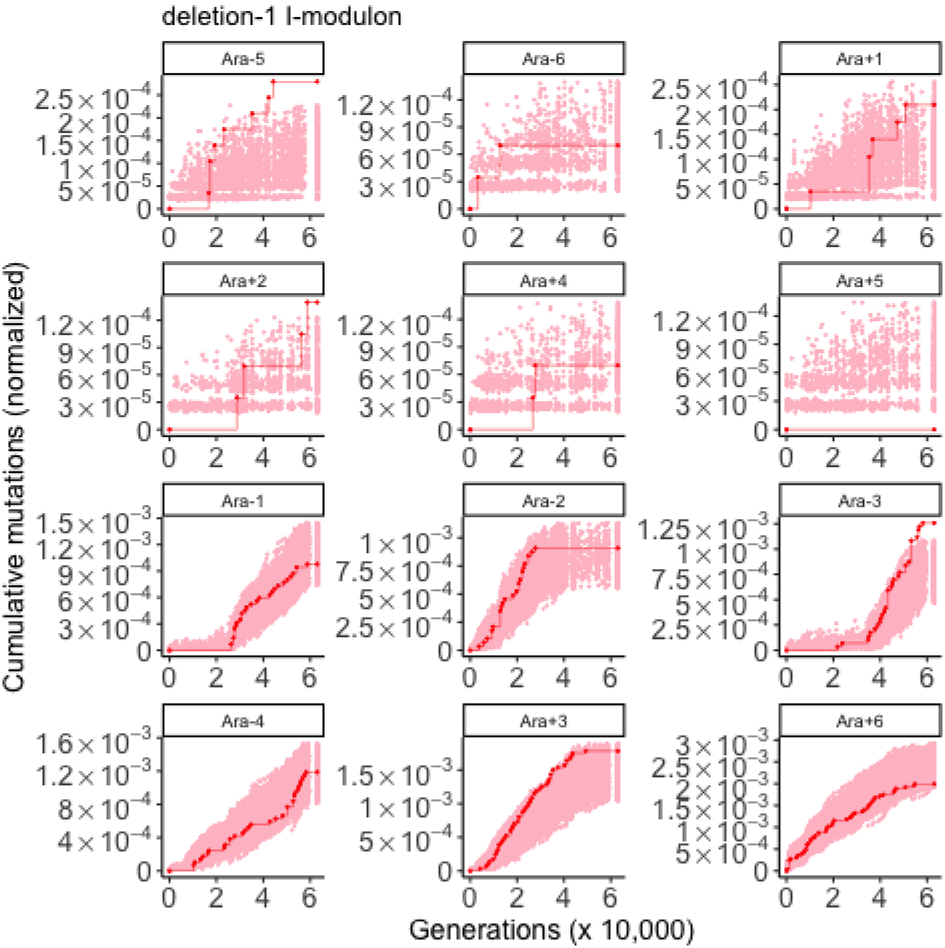
deletion-1 I-modulon
Ara-5
Ara-6
Ara+1
Ara+2
Ara+4
Ara+5
Ara-1
Ara-2
Ara-3
Ara-4
Ara+3
Ara+6
Cumulative mutations (normalized)
Generations (x 10,000)

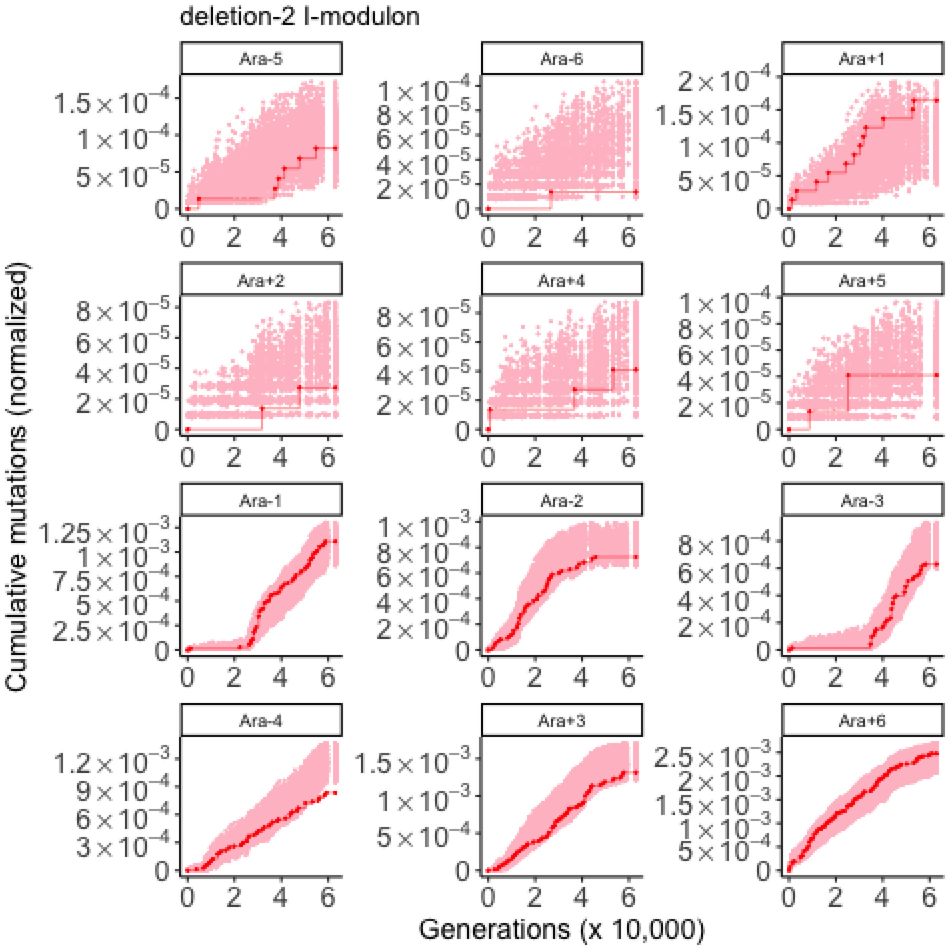

deletion-2 I-modulon
Ara-5
Ara-6
Ara+1
Ara+2
Ara+4
Ara+5
Ara-1
Ara-2
Ara-3
Ara-4
Ara+3
Ara+6
Cumulative mutations (normalized)
Generations (x 10,000)

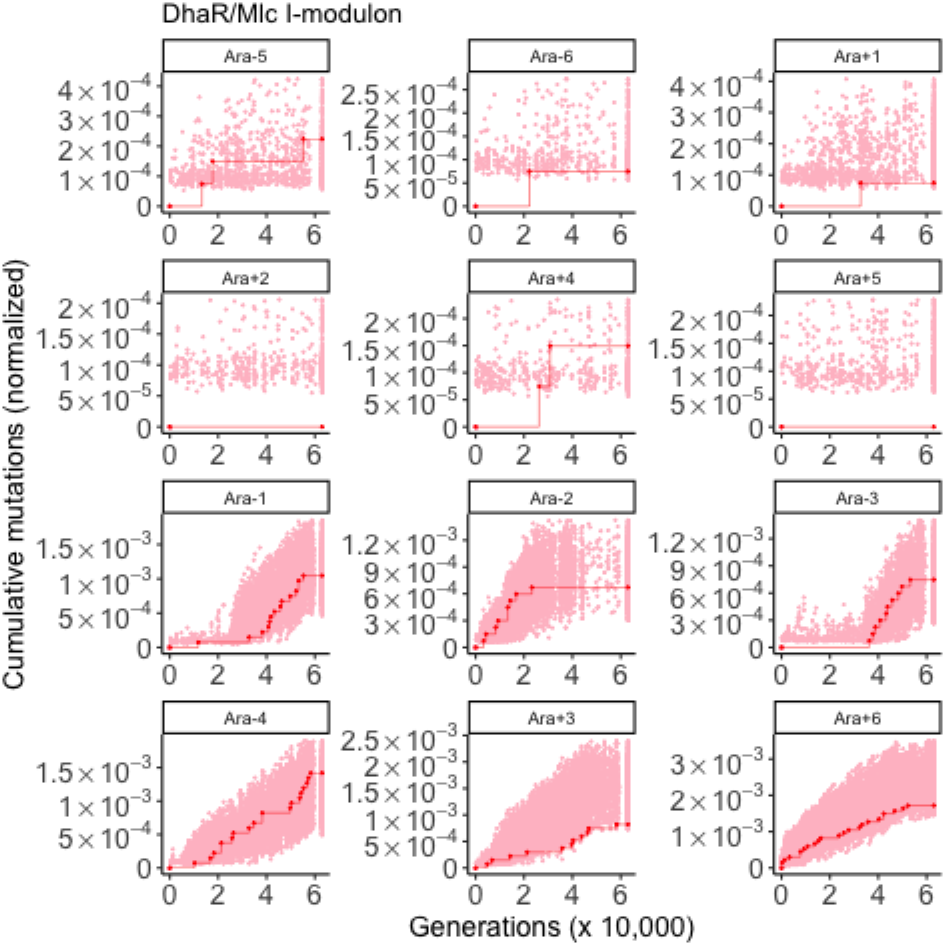
DhaR/Mlc I-modulon
Ara-5
Ara-6
Ara+1
Ara+2
Ara+4
Ara+5
Ara-1
Ara-2
Ara-3
Ara-4
Ara+3
Ara+6
Cumulative mutations (normalized)
Generations (x 10,000)
0
2
4
6
1×10^{-4}
2×10^{-4}
3×10^{-4}
4×10^{-4}
5×10^{-5}
1.5×10^{-4}
2.5×10^{-4}
5×10^{-4}
1×10^{-3}
1.5×10^{-3}
2×10^{-3}
2.5×10^{-3}
3×10^{-3}
6×10^{-4}
9×10^{-4}
1.2×10^{-3}

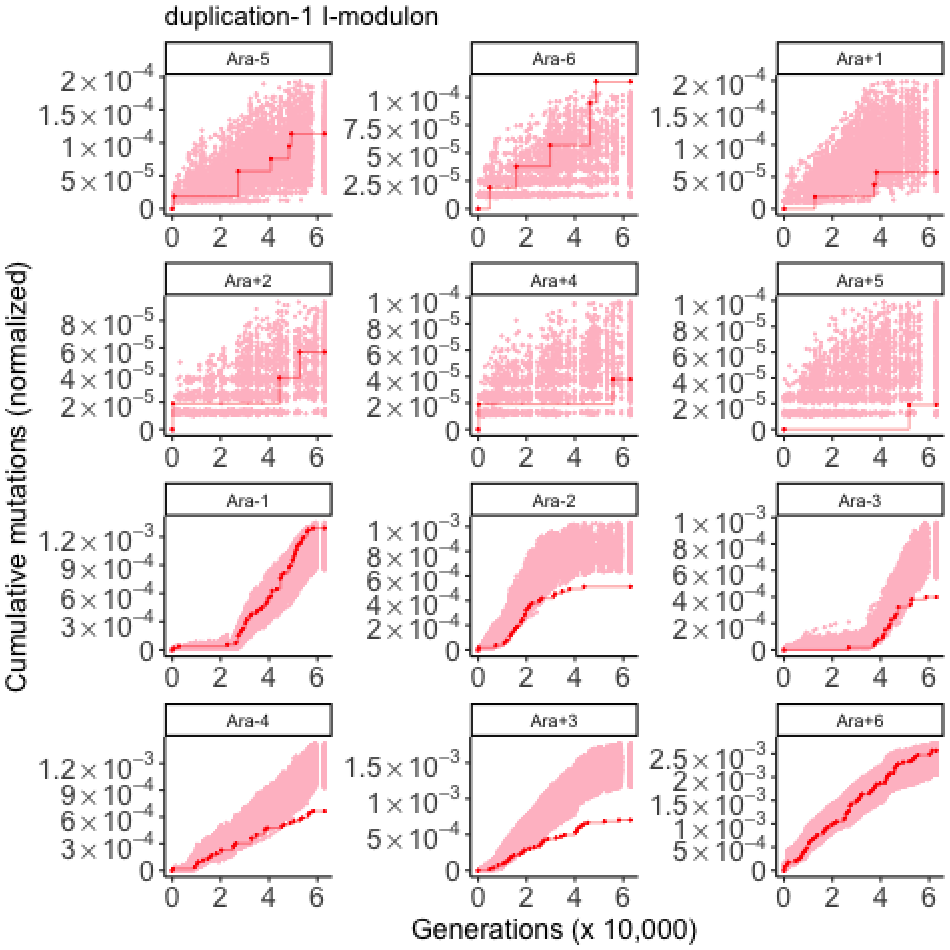
duplication-1 I-modulon
Ara-5
Ara-6
Ara+1
Ara+2
Ara+4
Ara+5
Ara-1
Ara-2
Ara-3
Ara-4
Ara+3
Ara+6
Cumulative mutations (normalized)
Generations (x 10,000)

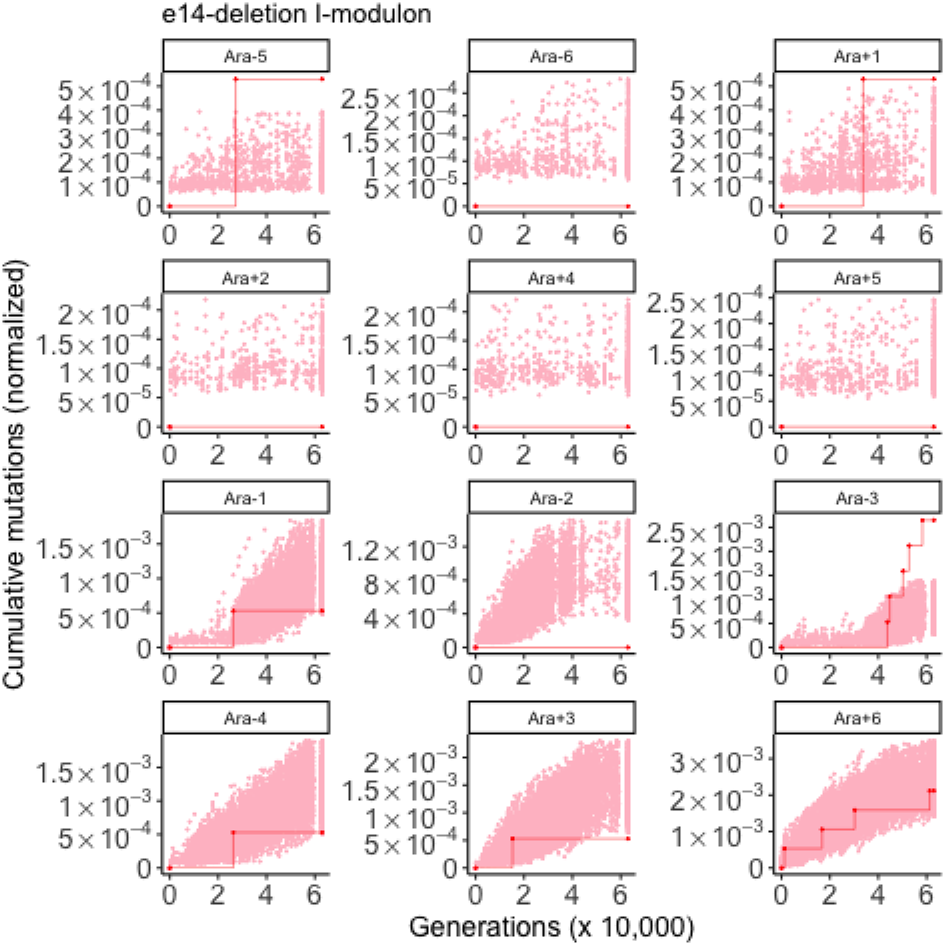

e14-deletion I-modulon
Ara-5
Ara-6
Ara+1
Ara+2
Ara+4
Ara+5
Ara-1
Ara-2
Ara-3
Ara-4
Ara+3
Ara+6
Cumulative mutations (normalized)
Generations (x 10,000)

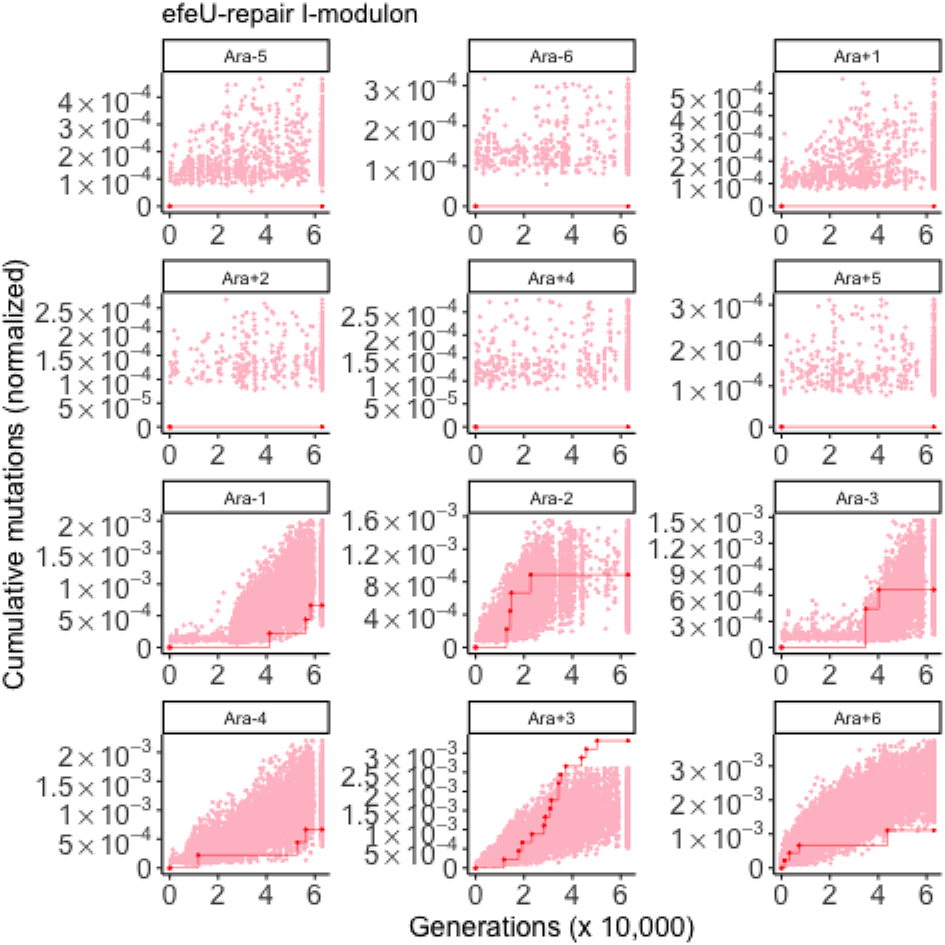

efeU-repair I-modulon
Ara-5
Ara-6
Ara+1
Ara+2
Ara+4
Ara+5
Ara-1
Ara-2
Ara-3
Ara-4
Ara+3
Ara+6
Cumulative mutations (normalized)
Generations (x 10,000)

entC-menF-KO I-modulon

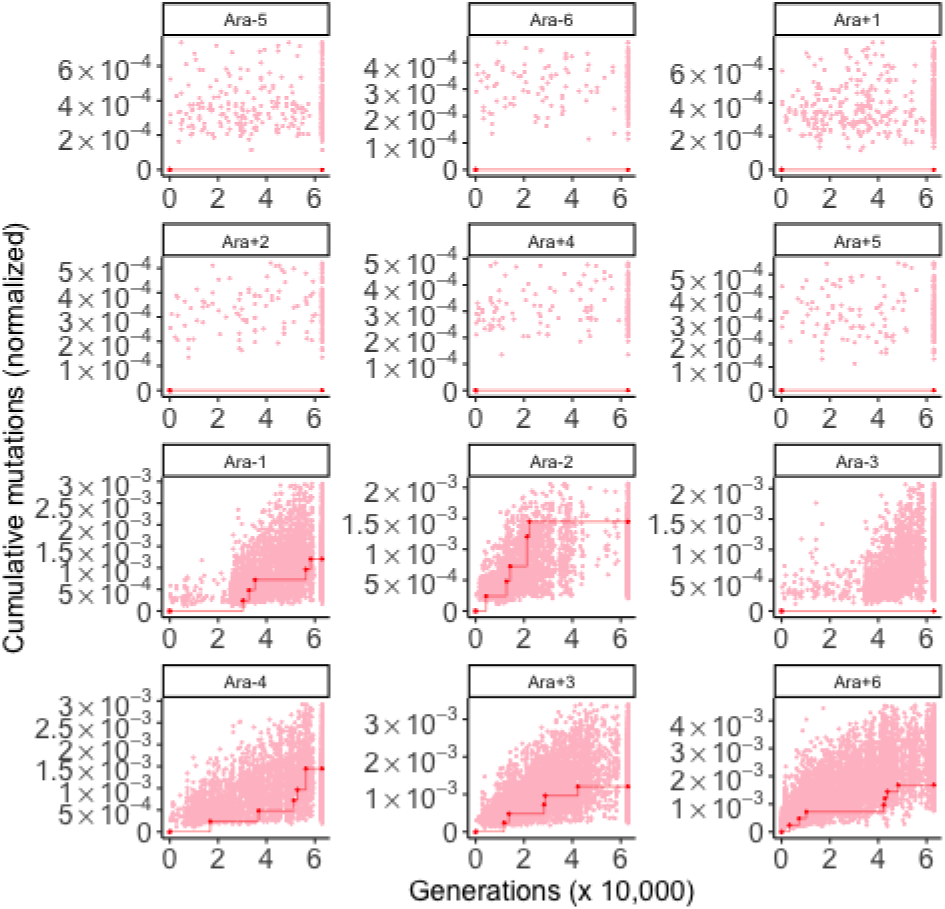

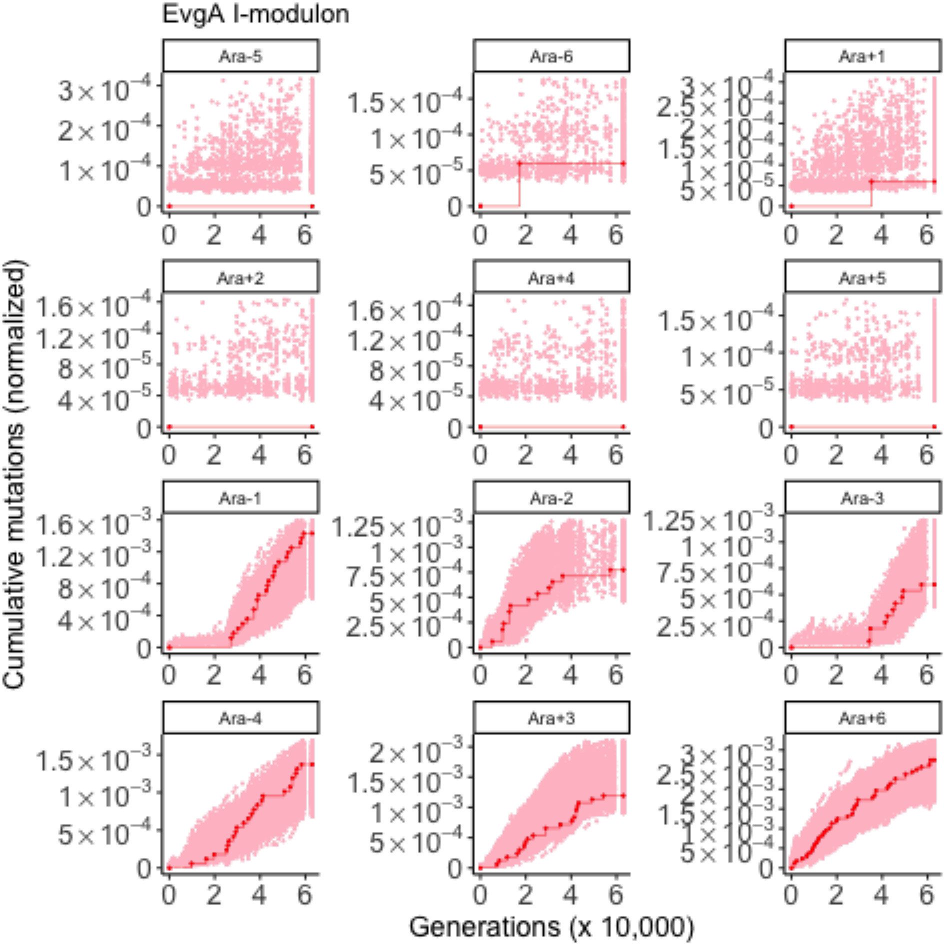
EvgA I-modulon
Ara-5
Ara-6
Ara+1
Ara+2
Ara+4
Ara+5
Ara-1
Ara-2
Ara-3
Ara-4
Ara+3
Ara+6
Cumulative mutations (normalized)
Generations (x 10,000)

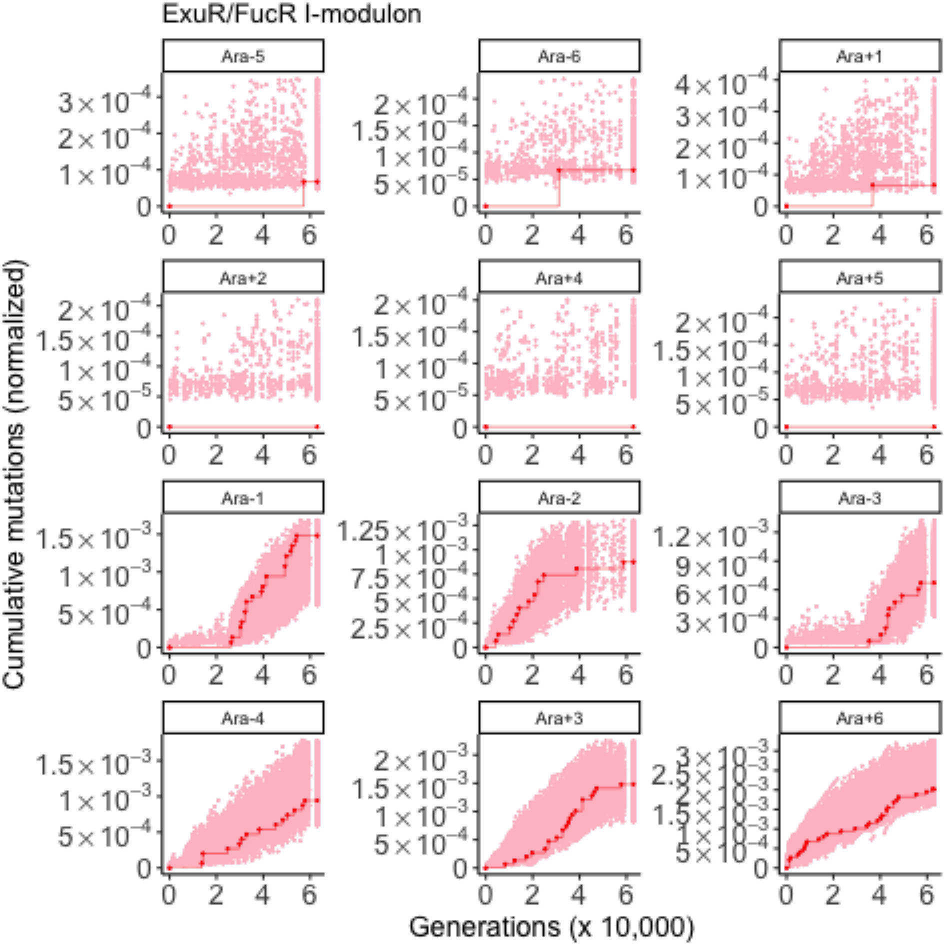

ExuR/FucR I-modulon
Ara-5
Ara-6
Ara+1
Ara+2
Ara+4
Ara+5
Ara-1
Ara-2
Ara-3
Ara-4
Ara+3
Ara+6
Cumulative mutations (normalized)
Generations (x 10,000)

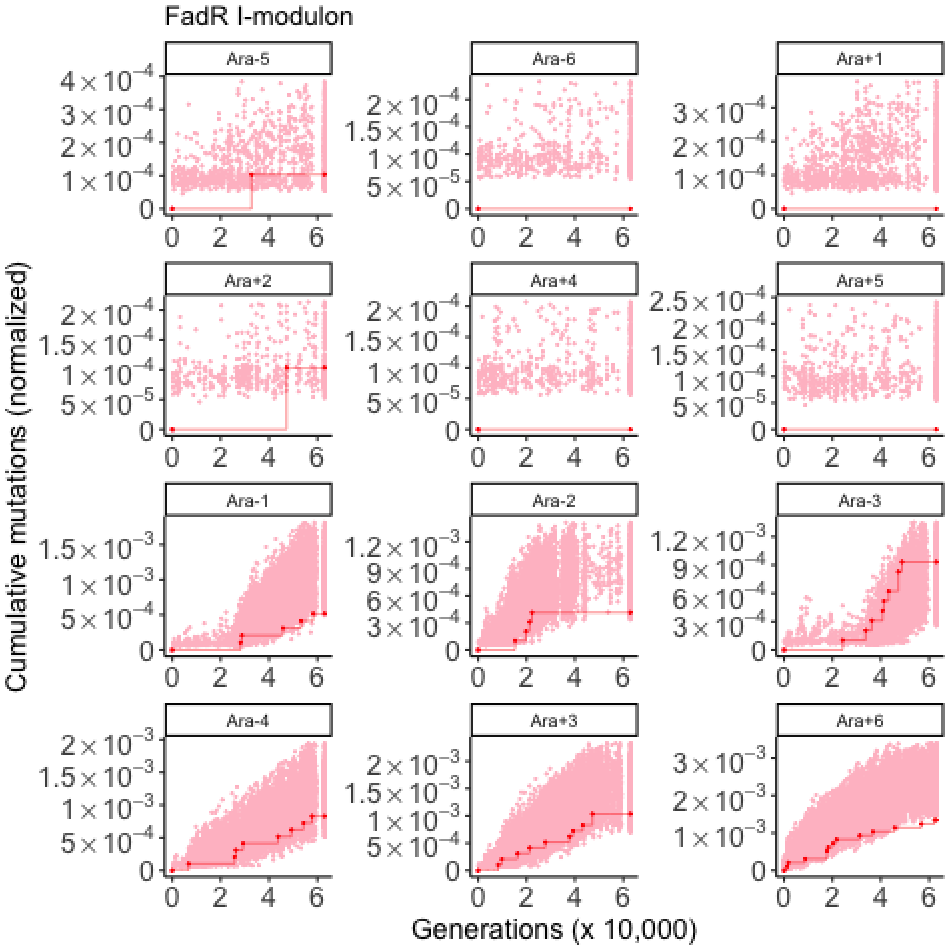
FadR I-modulon
Ara-5
Ara-6
Ara+1
Ara+2
Ara+4
Ara+5
Ara-1
Ara-2
Ara-3
Ara-4
Ara+3
Ara+6
Cumulative mutations (normalized)
Generations (x 10,000)

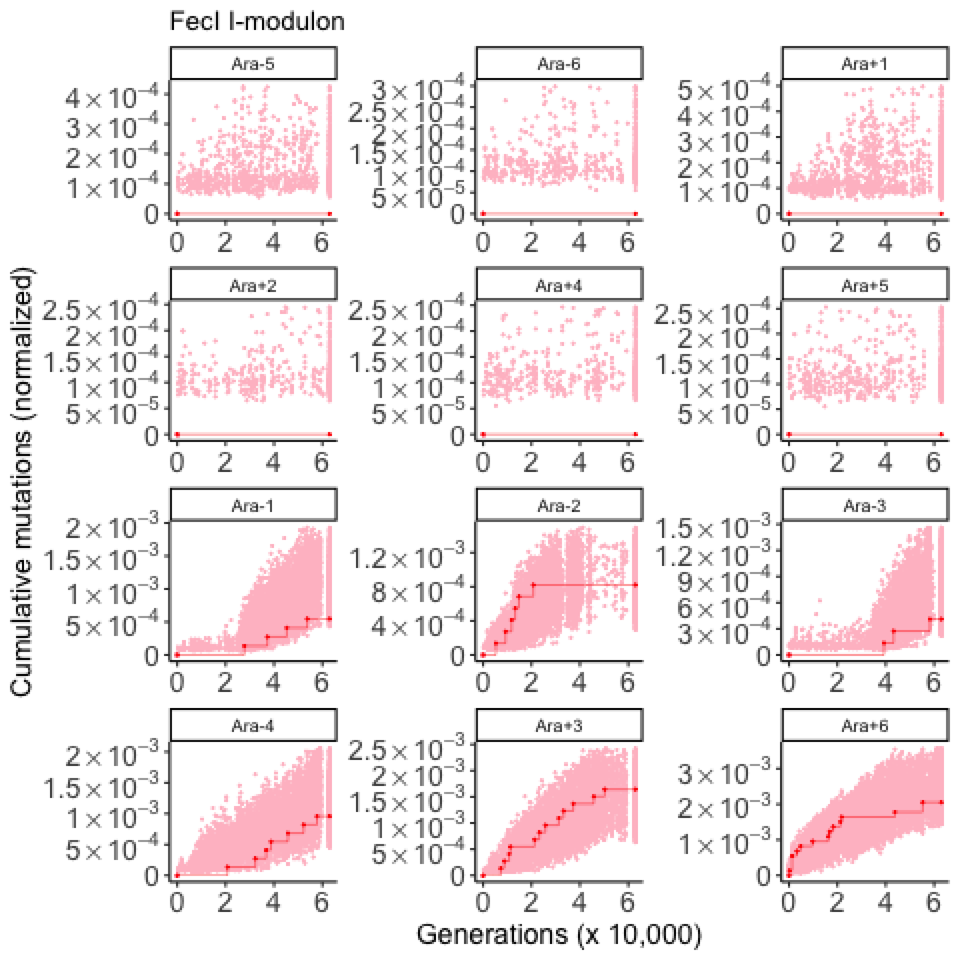

FecI I-modulon
Ara-5
Ara-6
Ara+1
Ara+2
Ara+4
Ara+5
Ara-1
Ara-2
Ara-3
Ara-4
Ara+3
Ara+6
Cumulative mutations (normalized)
Generations (x 10,000)

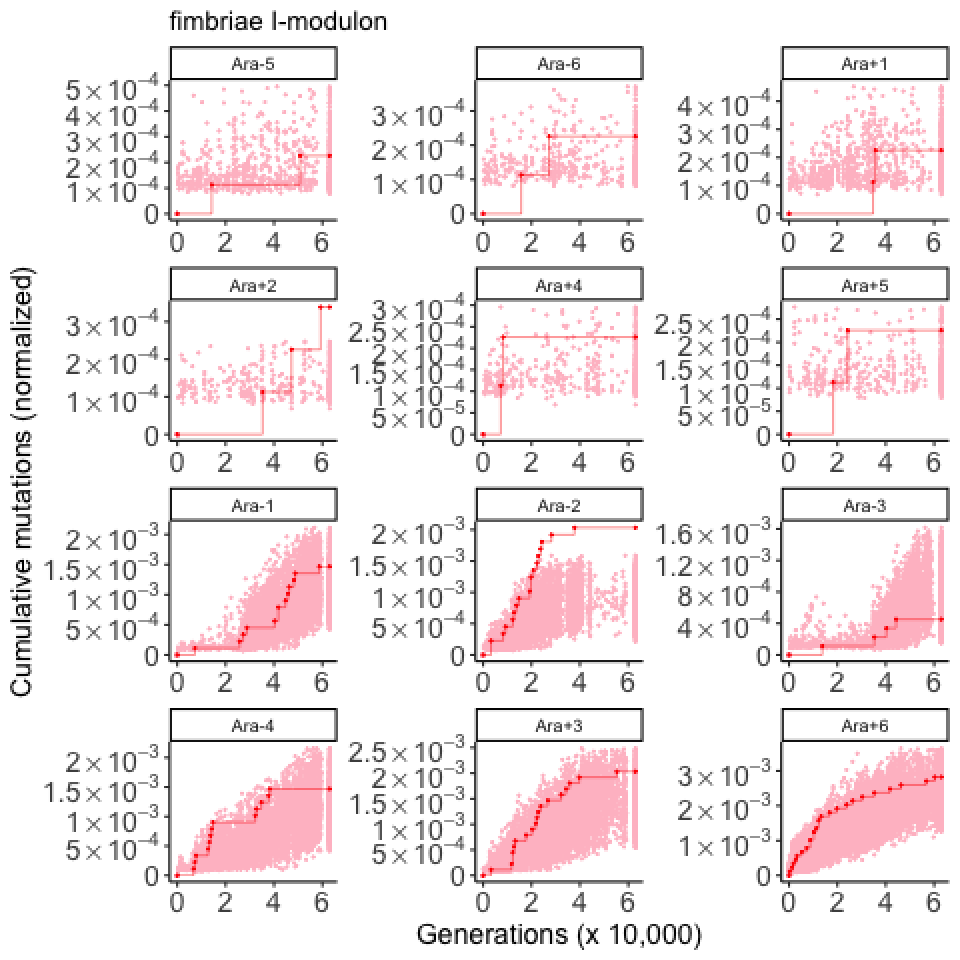
fimbriae I-modulon
Ara-5
Ara-6
Ara+1
Ara+2
Ara+4
Ara+5
Ara-1
Ara-2
Ara-3
Ara-4
Ara+3
Ara+6
Cumulative mutations (normalized)
Generations (x 10,000)

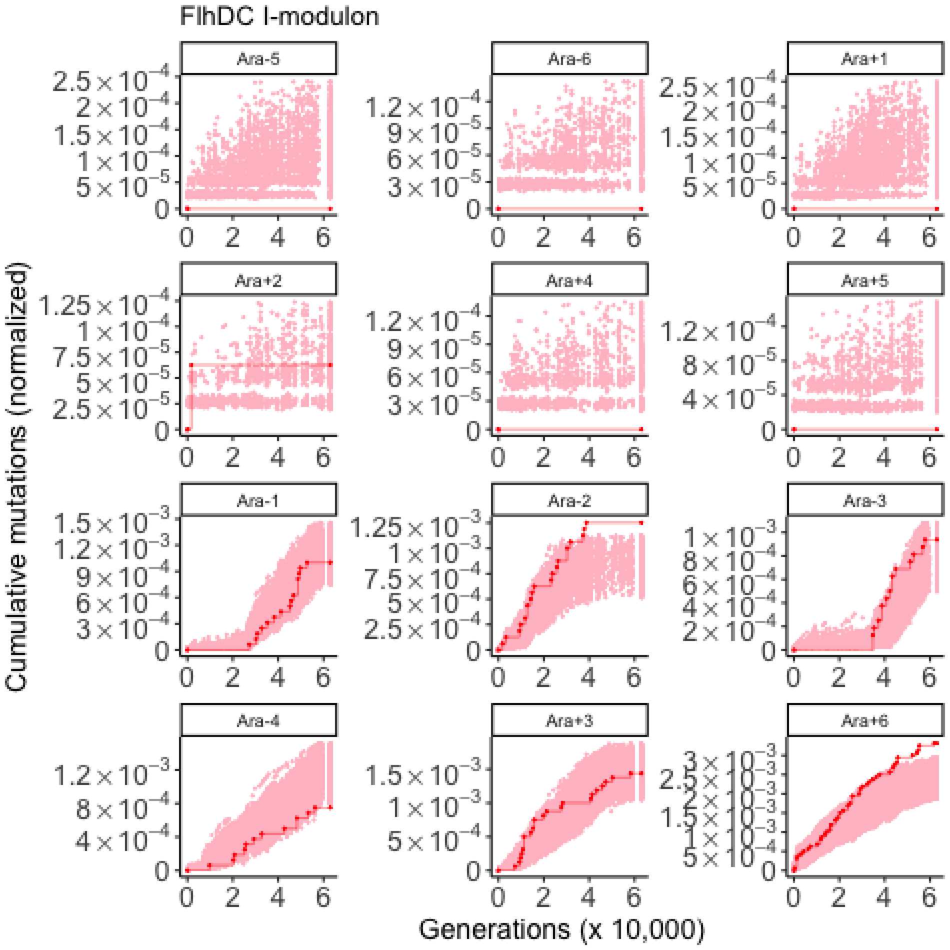

FlhDC I-modulon
Ara-5
Ara-6
Ara+1
Ara+2
Ara+4
Ara+5
Ara-1
Ara-2
Ara-3
Ara-4
Ara+3
Ara+6
Cumulative mutations (normalized)
Generations (x 10,000)

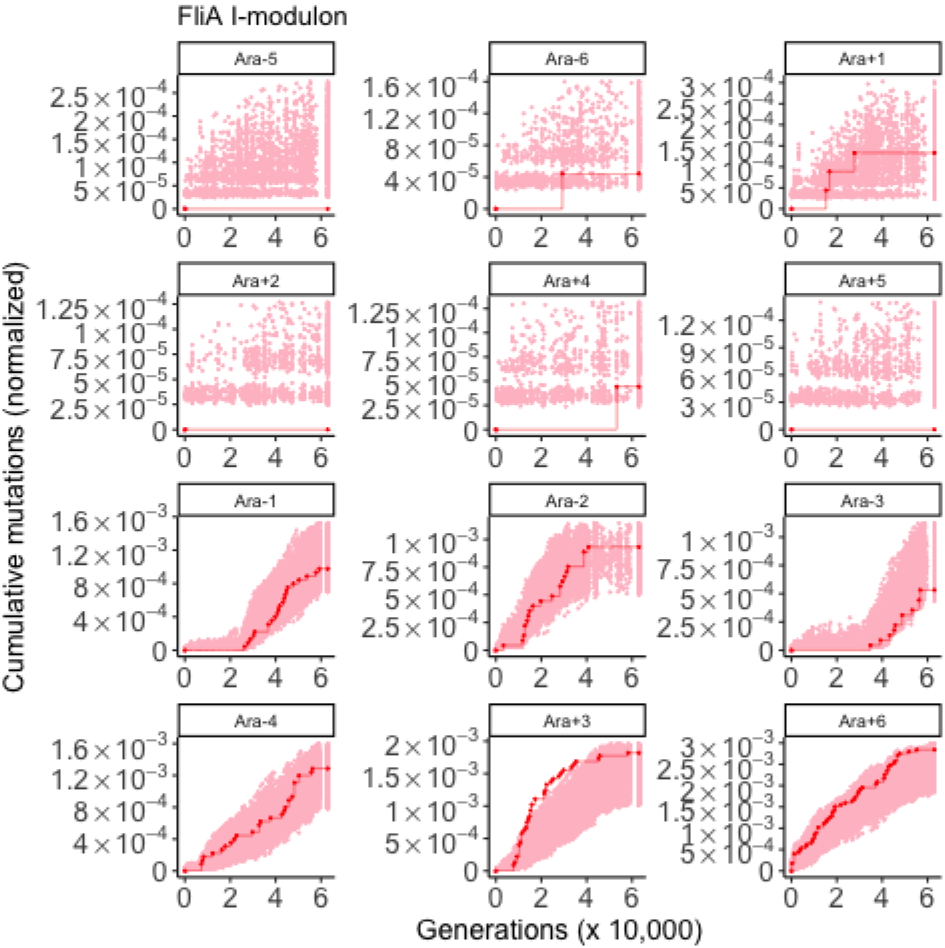
FliA I-modulon
Ara-5
Ara-6
Ara+1
Ara+2
Ara+4
Ara+5
Ara-1
Ara-2
Ara-3
Ara-4
Ara+3
Ara+6
Cumulative mutations (normalized)
Generations (x 10,000)

flu-yeeRS I-modulon

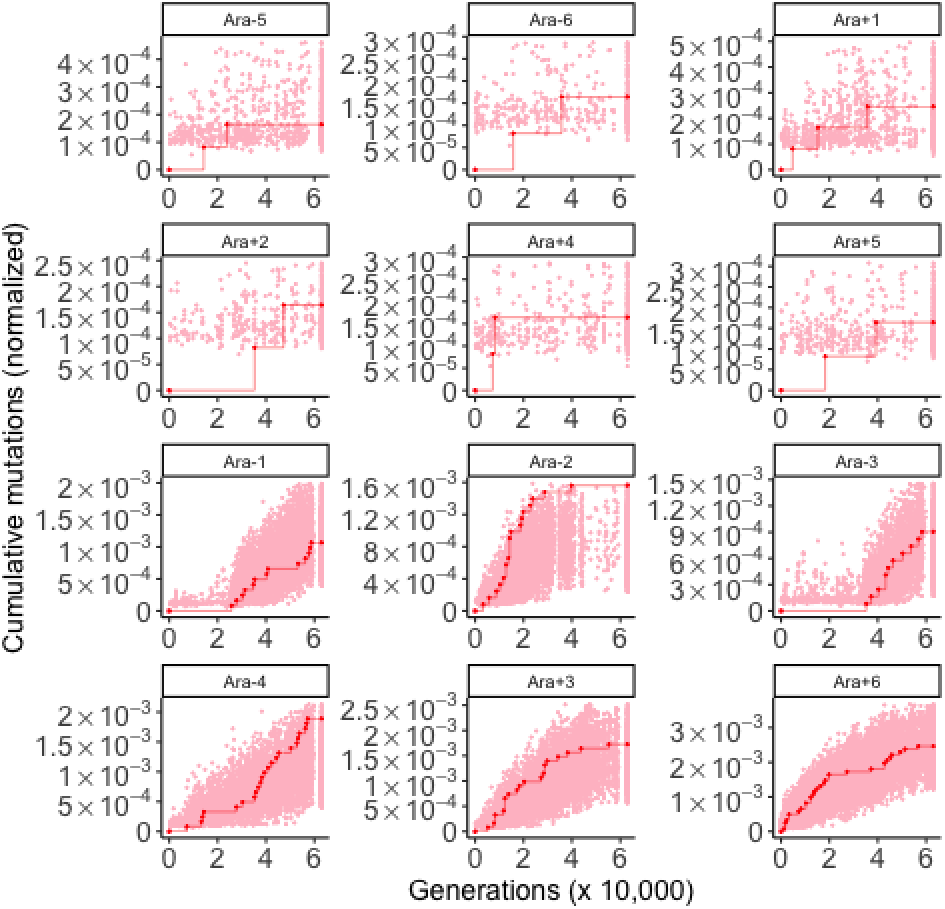

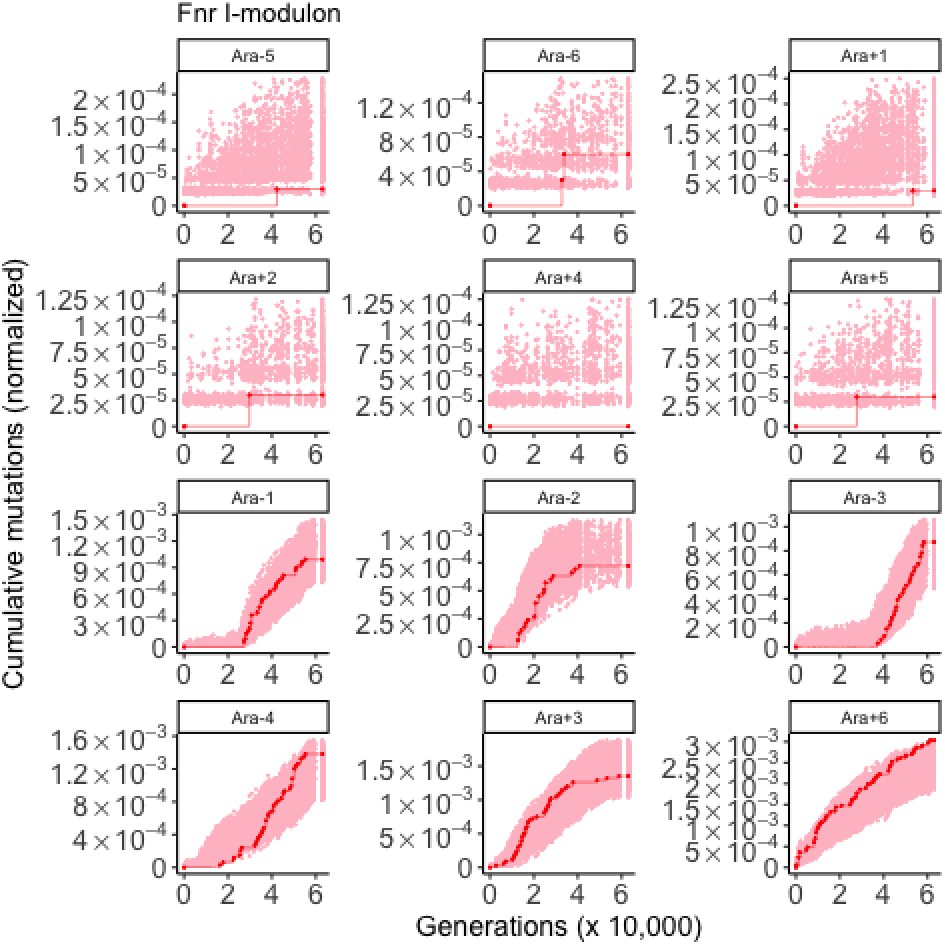

Fnr I-modulon
Ara-5
Ara-6
Ara+1
Ara+2
Ara+4
Ara+5
Ara-1
Ara-2
Ara-3
Ara-4
Ara+3
Ara+6
Cumulative mutations (normalized)
Generations (x 10,000)

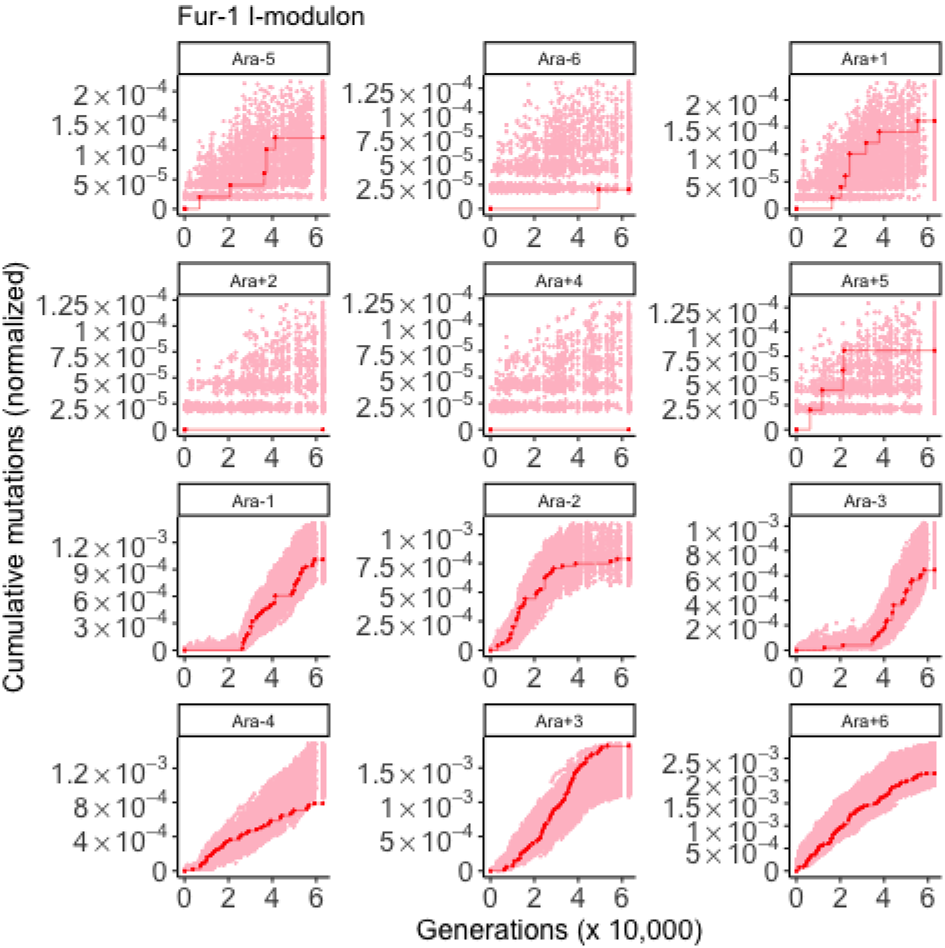

Fur-1 I-modulon
Ara-5
Ara-6
Ara+1
Ara+2
Ara+4
Ara+5
Ara-1
Ara-2
Ara-3
Ara-4
Ara+3
Ara+6
Cumulative mutations (normalized)
Generations (x 10,000)

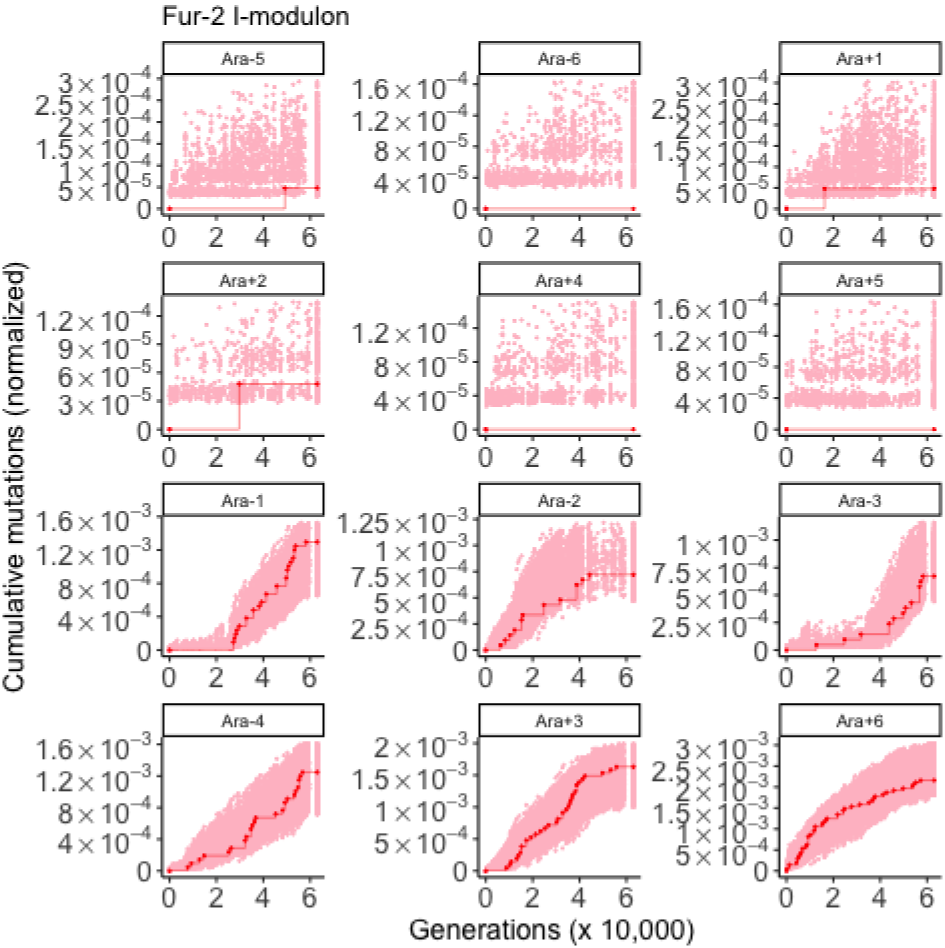

Fur-2 I-modulon
Ara-5
Ara-6
Ara+1
Ara+2
Ara+4
Ara+5
Ara-1
Ara-2
Ara-3
Ara-4
Ara+3
Ara+6
Cumulative mutations (normalized)
Generations (x 10,000)

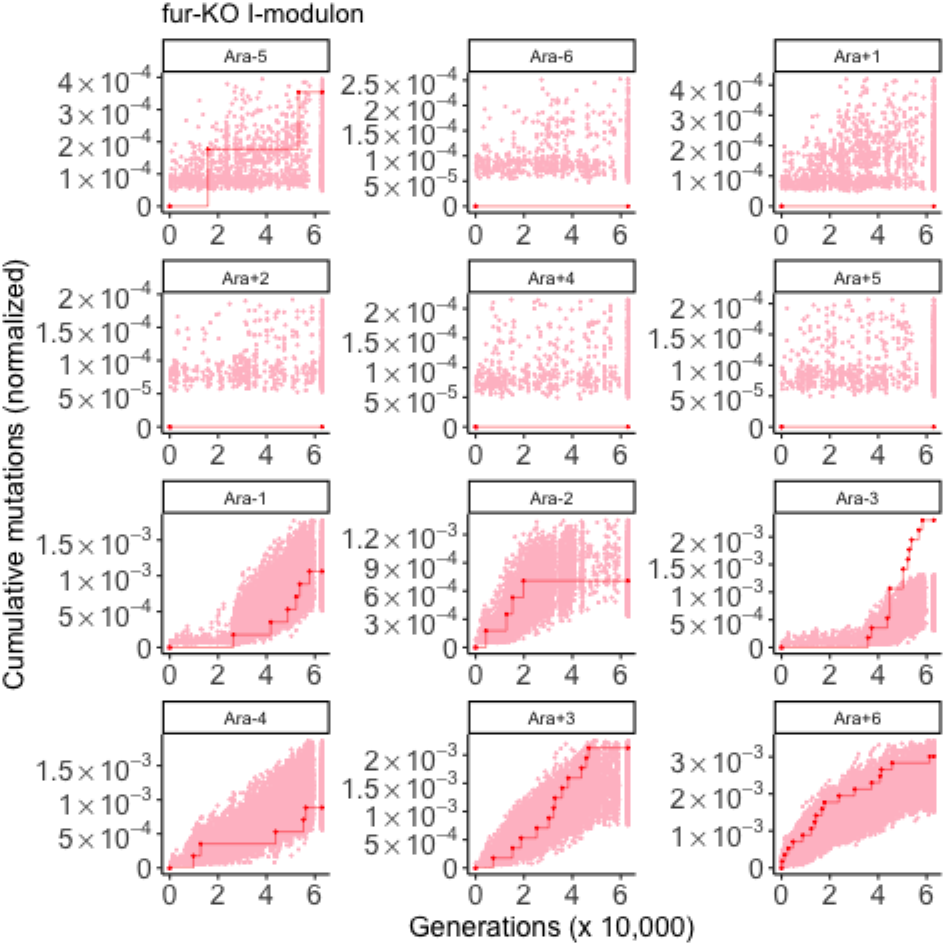
fur-KO I-modulon
Ara-5
Ara-6
Ara+1
Ara+2
Ara+4
Ara+5
Ara-1
Ara-2
Ara-3
Ara-4
Ara+3
Ara+6
Cumulative mutations (normalized)
Generations (x 10,000)

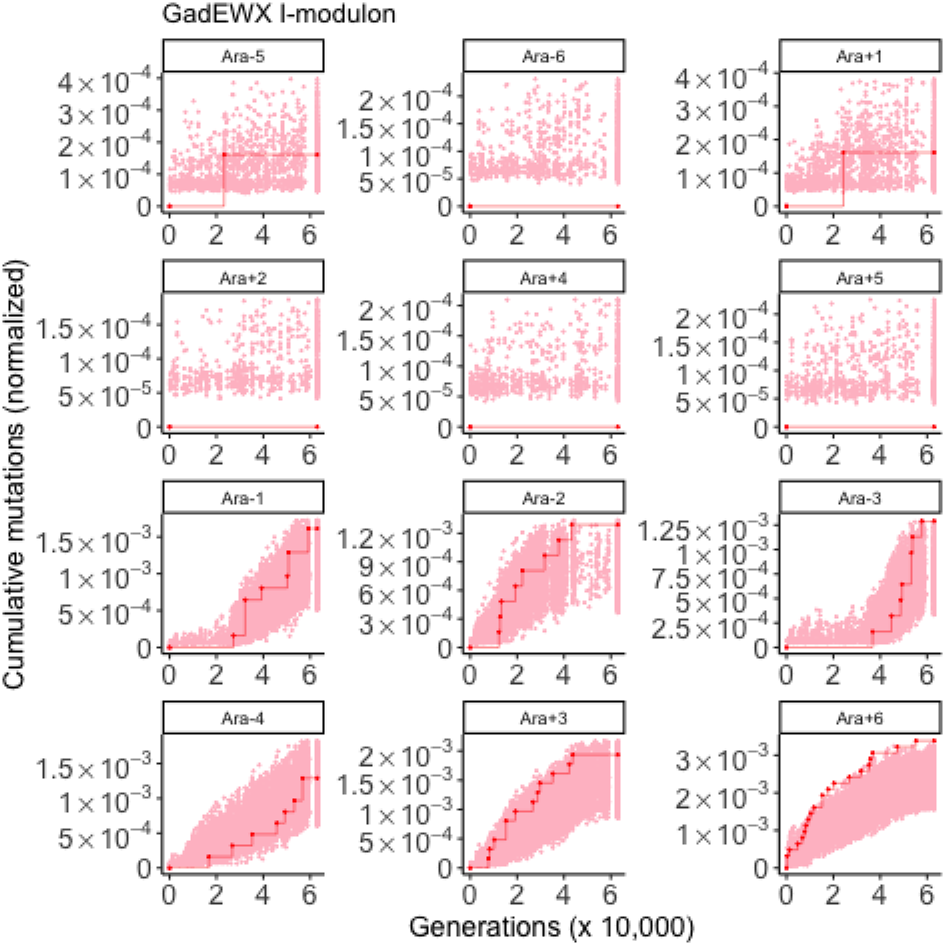

GadEWX I-modulon
Ara-5
Ara-6
Ara+1
Ara+2
Ara+4
Ara+5
Ara-1
Ara-2
Ara-3
Ara-4
Ara+3
Ara+6
Cumulative mutations (normalized)
Generations (x 10,000)

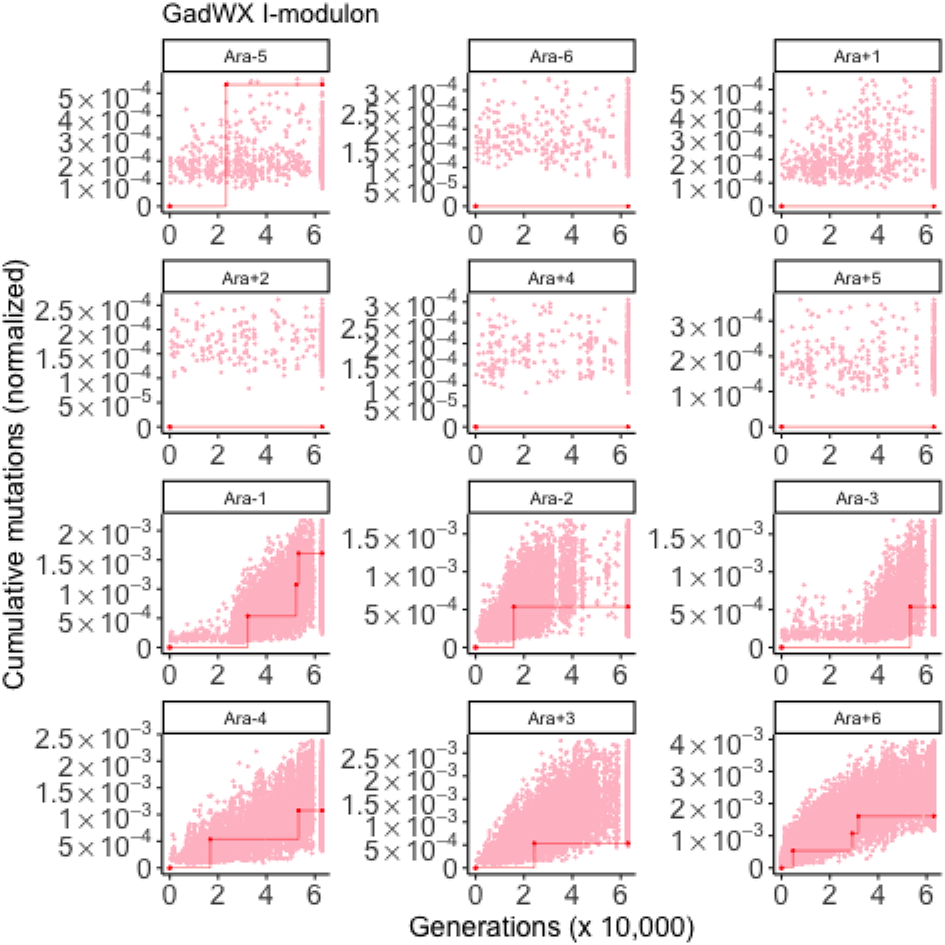
GadWX I-modulon
Ara-5
Ara-6
Ara+1
Ara+2
Ara+4
Ara+5
Ara-1
Ara-2
Ara-3
Ara-4
Ara+3
Ara+6
Cumulative mutations (normalized)
Generations (x 10,000)

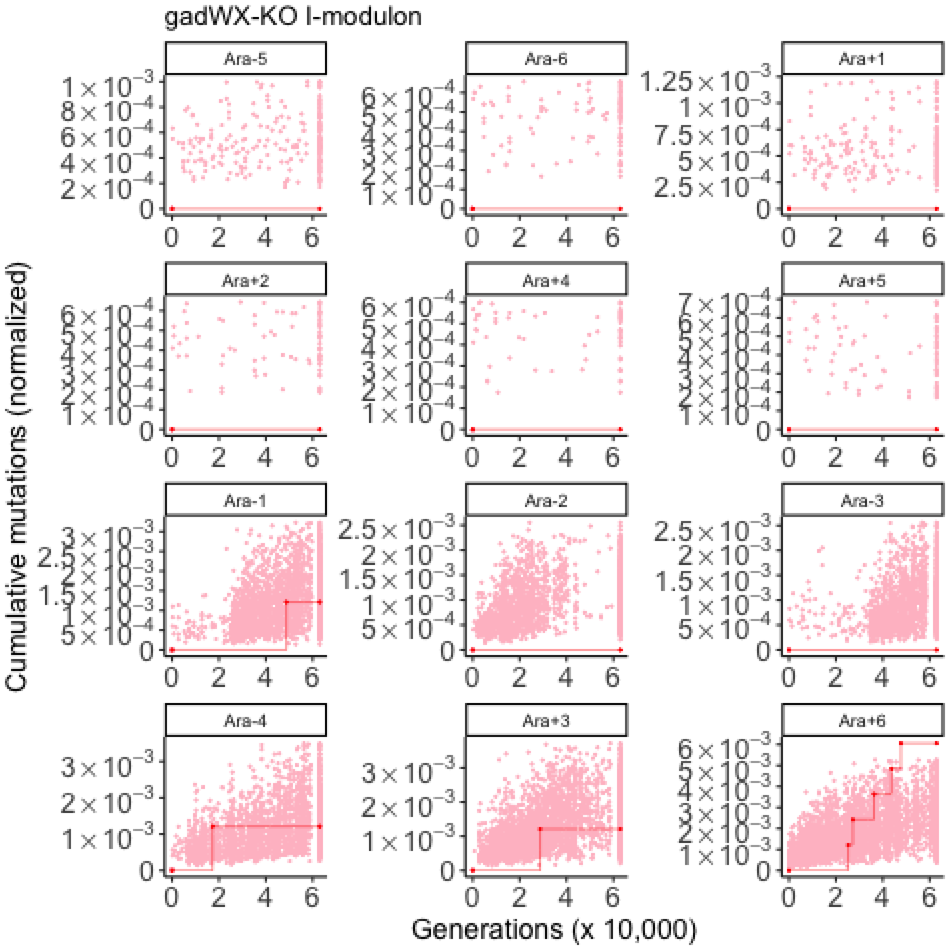
gadWX-KO I-modulon
Ara-5
Ara-6
Ara+1
Ara+2
Ara+4
Ara+5
Ara-1
Ara-2
Ara-3
Ara-4
Ara+3
Ara+6
Cumulative mutations (normalized)
Generations (x 10,000)

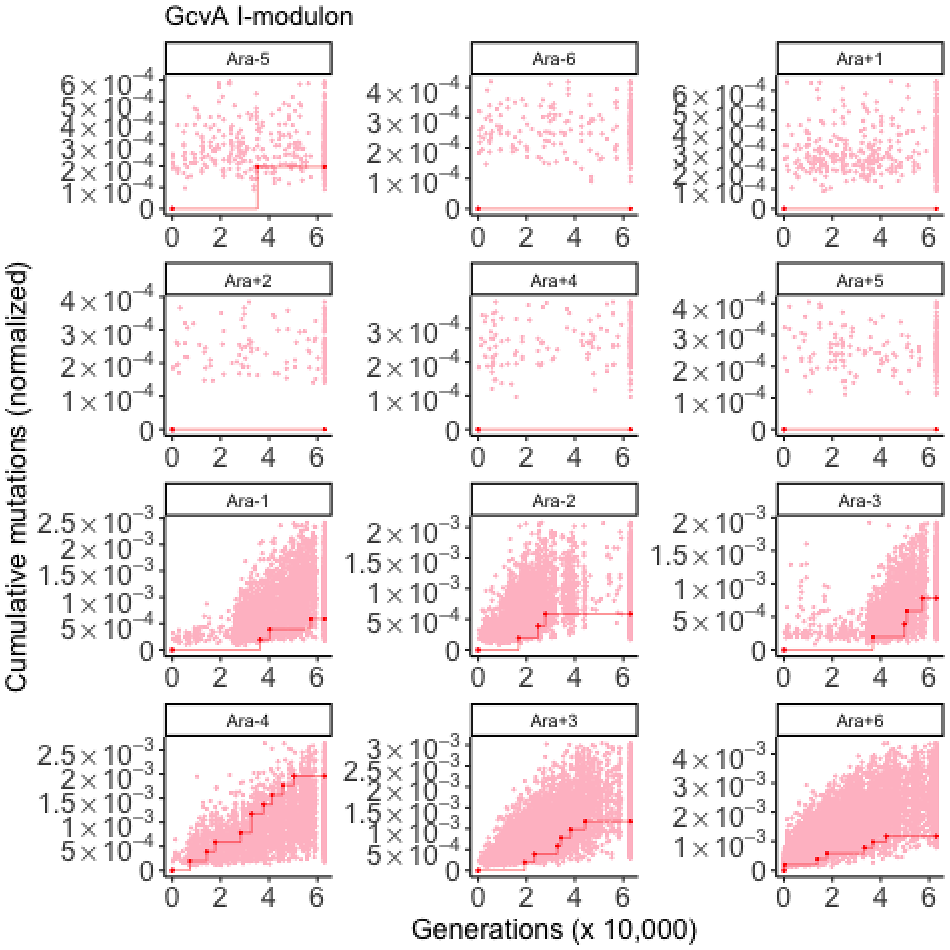
GcvA I-modulon
Ara-5
Ara-6
Ara+1
Ara+2
Ara+4
Ara+5
Ara-1
Ara-2
Ara-3
Ara-4
Ara+3
Ara+6
Cumulative mutations (normalized)
Generations (x 10,000)

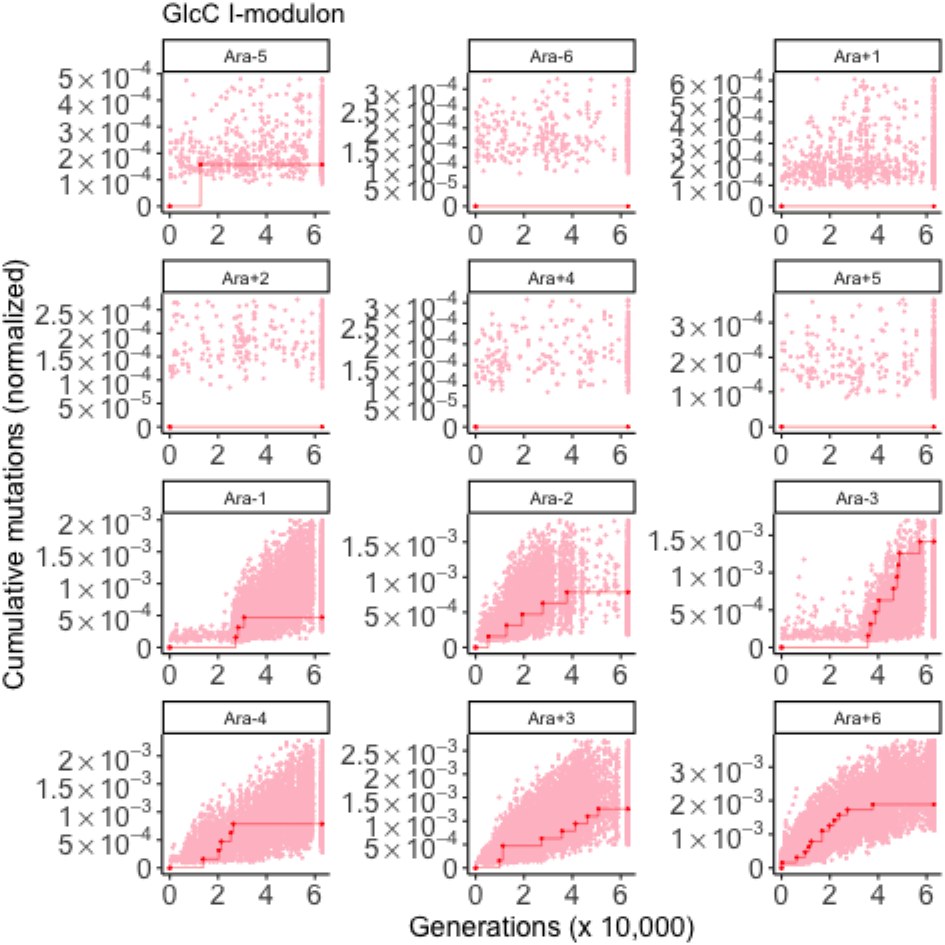

GlcC I-modulon
Ara-5
Ara-6
Ara+1
Ara+2
Ara+4
Ara+5
Ara-1
Ara-2
Ara-3
Ara-4
Ara+3
Ara+6
Cumulative mutations (normalized)
Generations (x 10,000)

GlpR I-modulon

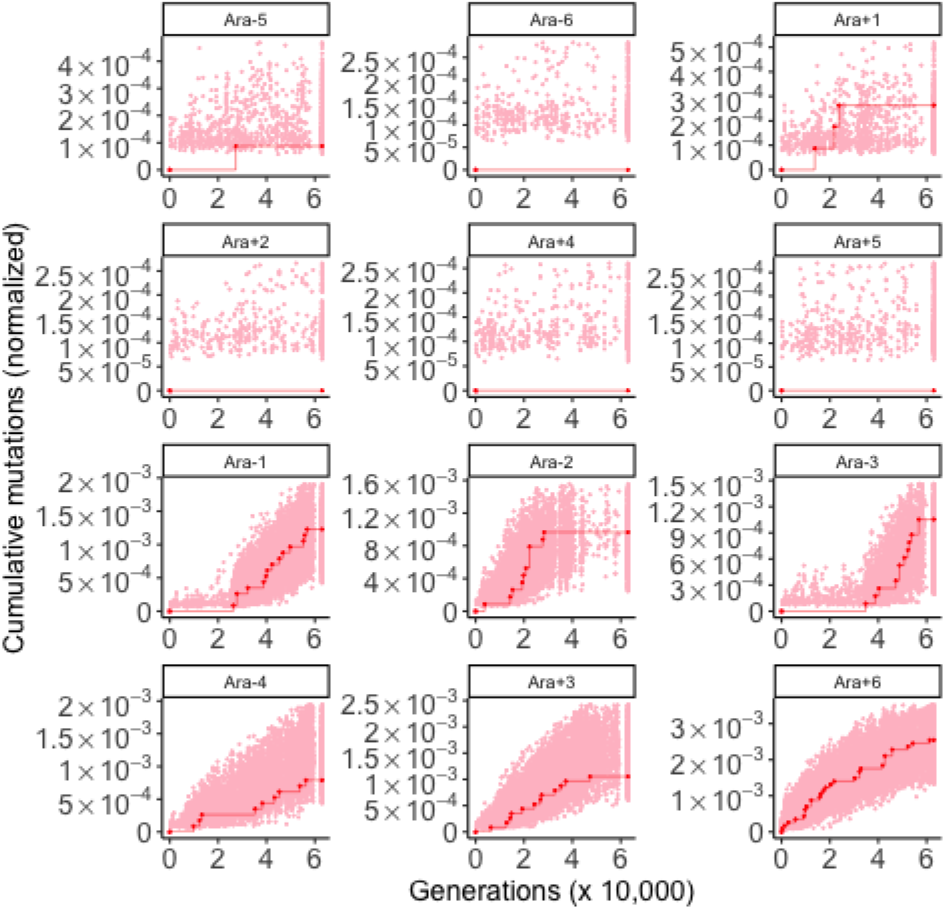

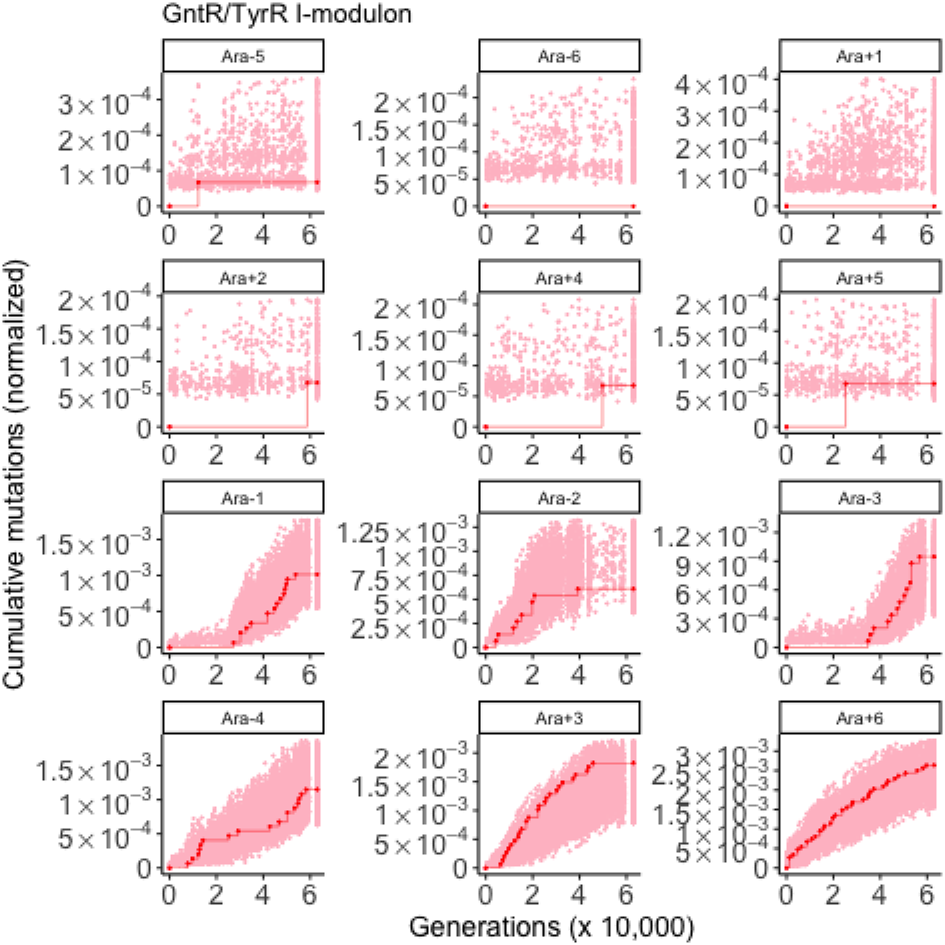
GntR/TyrR I-modulon
Ara-5
Ara-6
Ara+1
Ara+2
Ara+4
Ara+5
Ara-1
Ara-2
Ara-3
Ara-4
Ara+3
Ara+6
Cumulative mutations (normalized)
Generations (x 10,000)

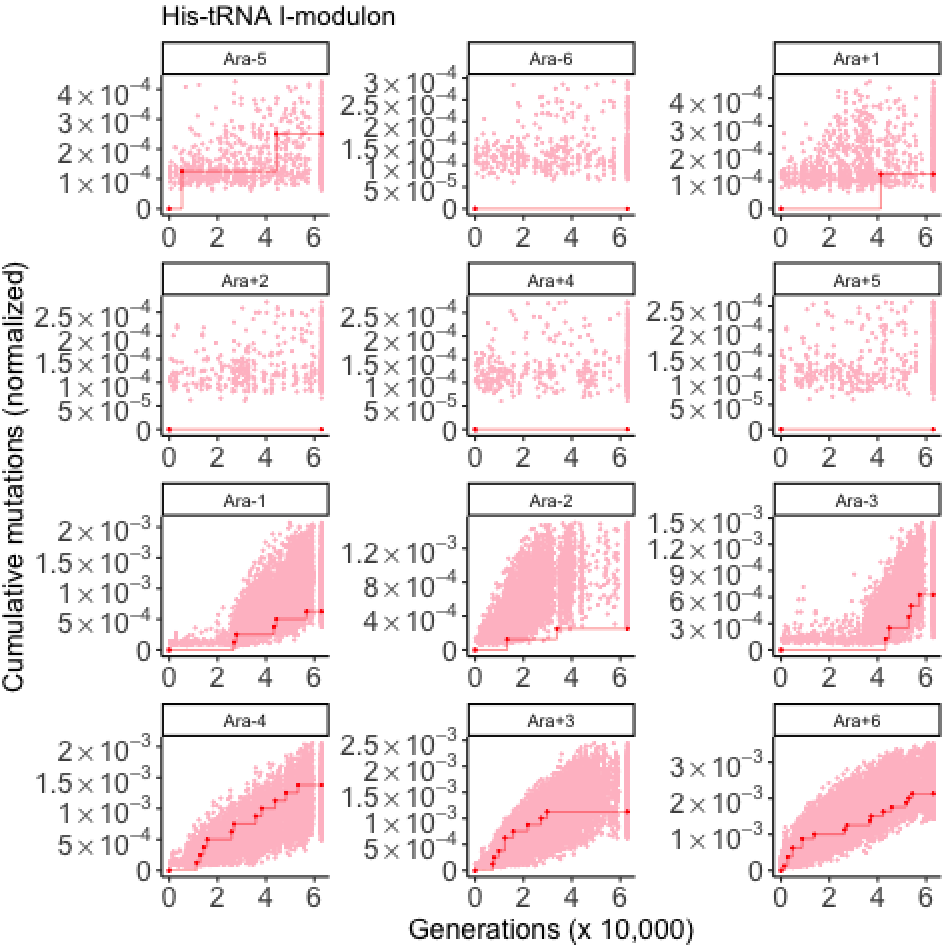

His-tRNA I-modulon
Ara-5
Ara-6
Ara+1
Ara+2
Ara+4
Ara+5
Ara-1
Ara-2
Ara-3
Ara-4
Ara+3
Ara+6
Cumulative mutations (normalized)
Generations (x 10,000)

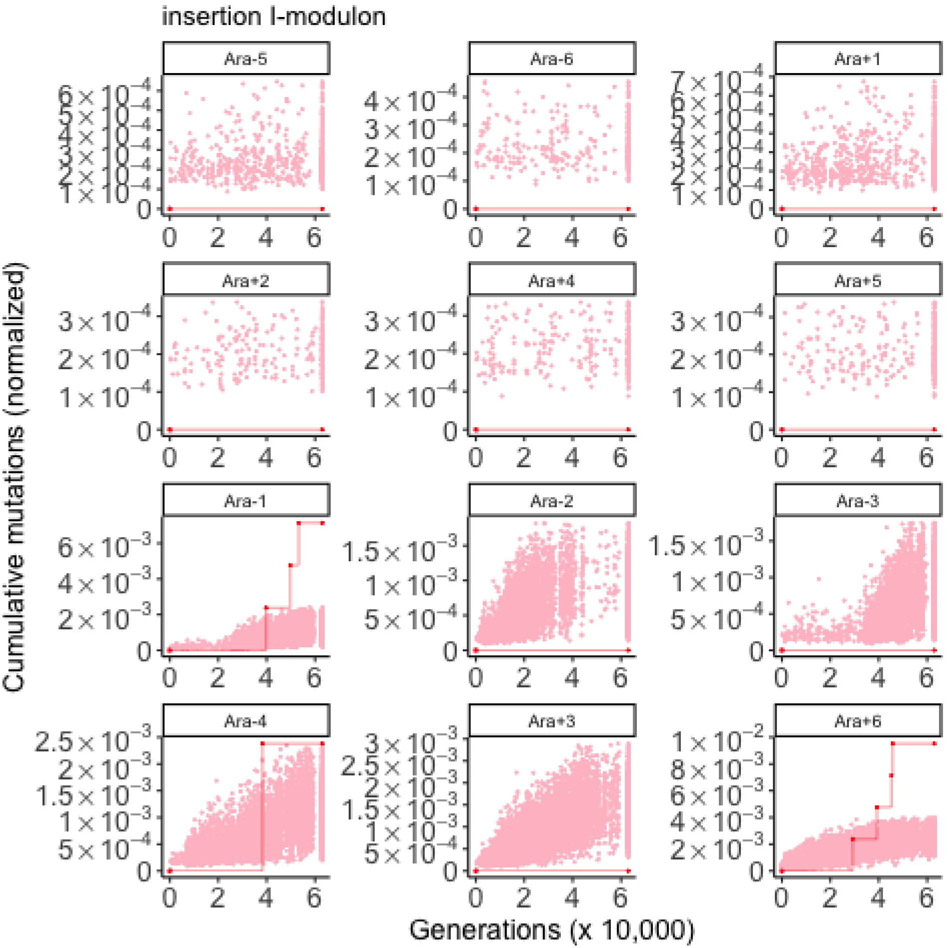
insertion I-modulon
Ara-5
Ara-6
Ara+1
Ara+2
Ara+4
Ara+5
Ara-1
Ara-2
Ara-3
Ara-4
Ara+3
Ara+6
Cumulative mutations (normalized)
Generations (x 10,000)

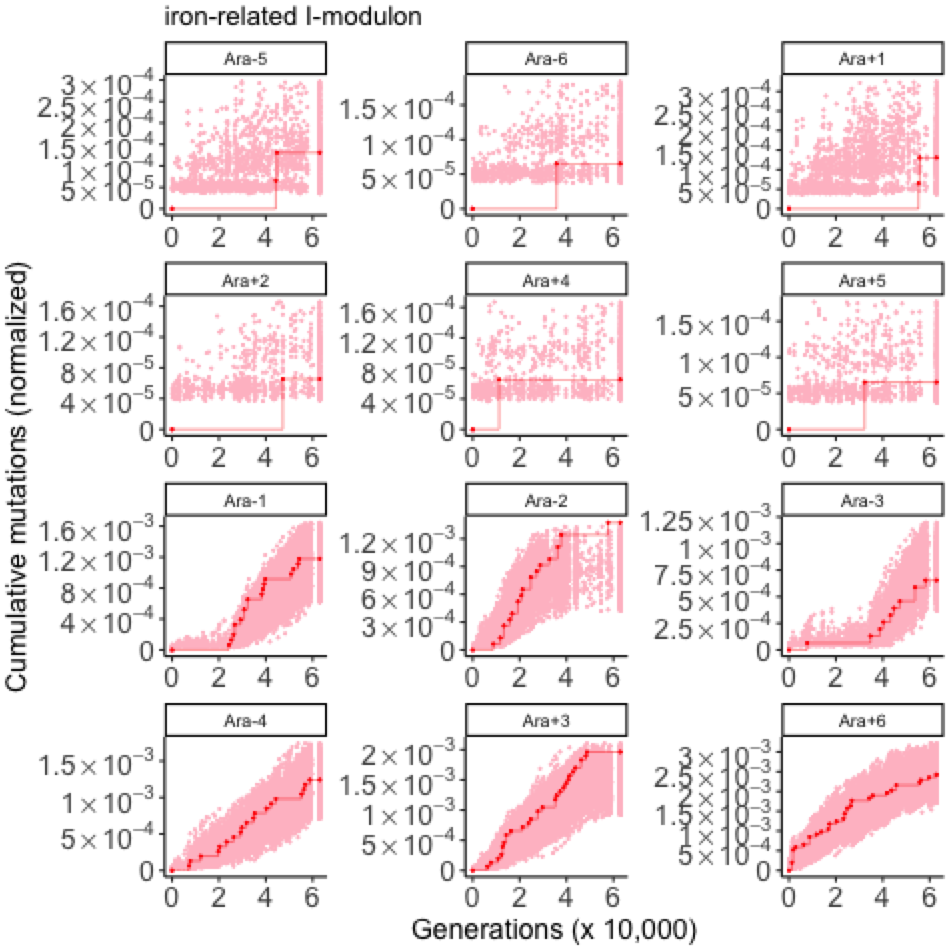
iron-related I-modulon
Ara-5
Ara-6
Ara+1
Ara+2
Ara+4
Ara+5
Ara-1
Ara-2
Ara-3
Ara-4
Ara+3
Ara+6
Cumulative mutations (normalized)
Generations (x 10,000)

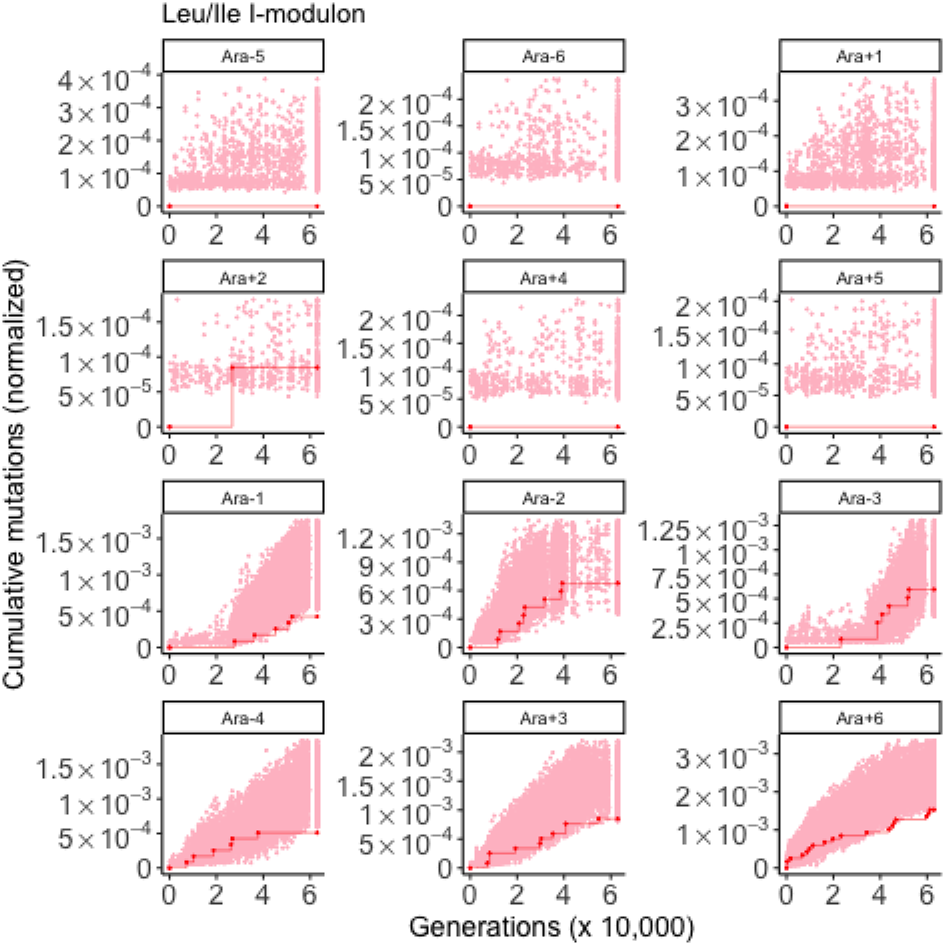

Leu/Ile I-modulon
Ara-5
Ara-6
Ara+1
Ara+2
Ara+4
Ara+5
Ara-1
Ara-2
Ara-3
Ara-4
Ara+3
Ara+6
Cumulative mutations (normalized)
Generations (x 10,000)

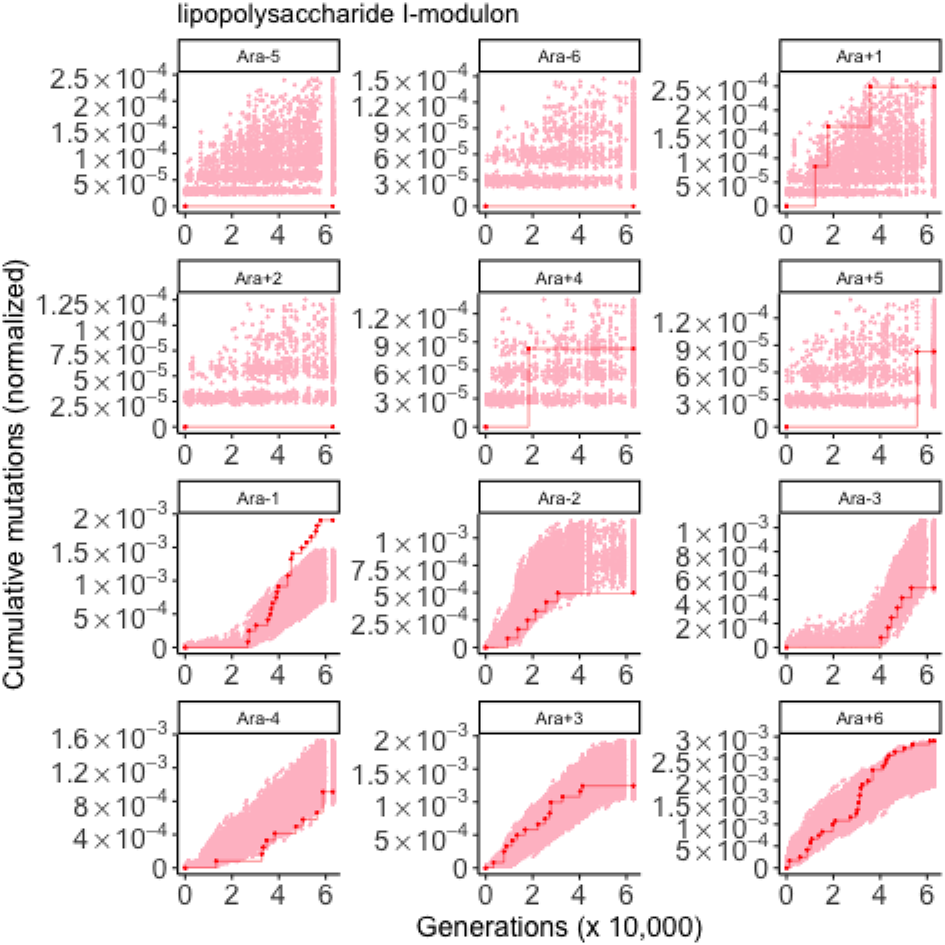

lipopolysaccharide I-modulon
Ara-5
Ara-6
Ara+1
Ara+2
Ara+4
Ara+5
Ara-1
Ara-2
Ara-3
Ara-4
Ara+3
Ara+6
Cumulative mutations (normalized)
Generations (x 10,000)

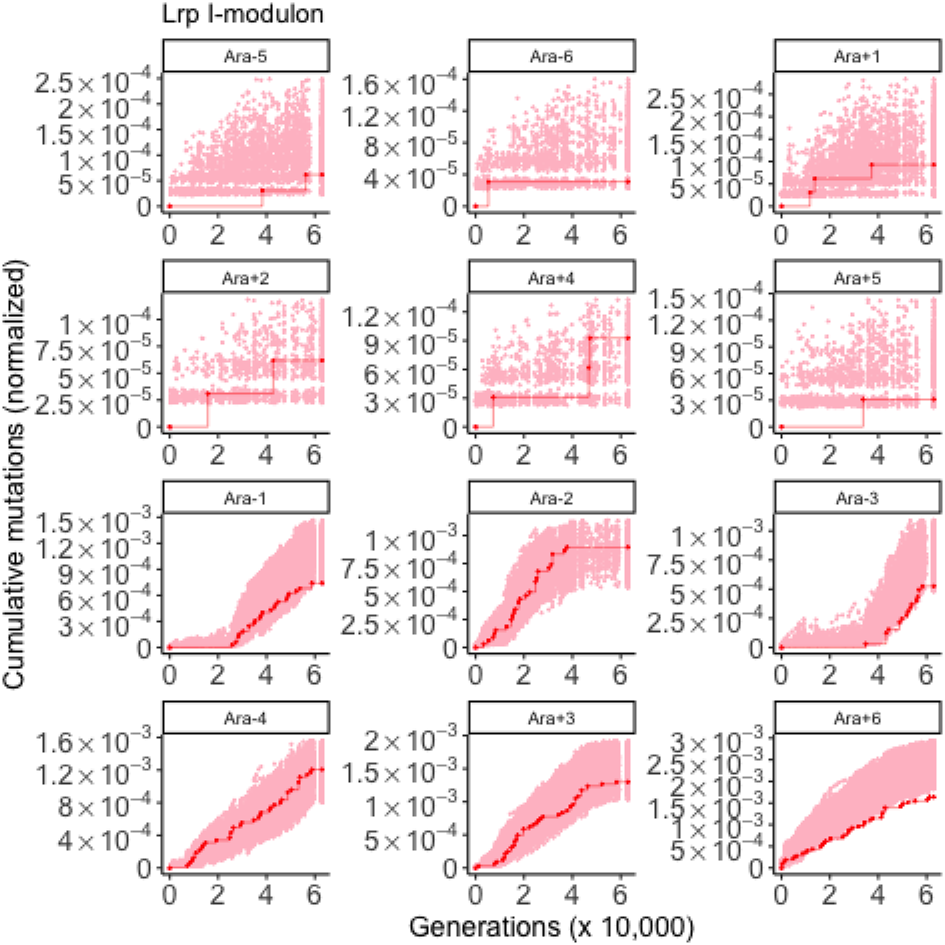

Lrp I-modulon
Ara-5
Ara-6
Ara+1
Ara+2
Ara+4
Ara+5
Ara-1
Ara-2
Ara-3
Ara-4
Ara+3
Ara+6
Cumulative mutations (normalized)
Generations (x 10,000)

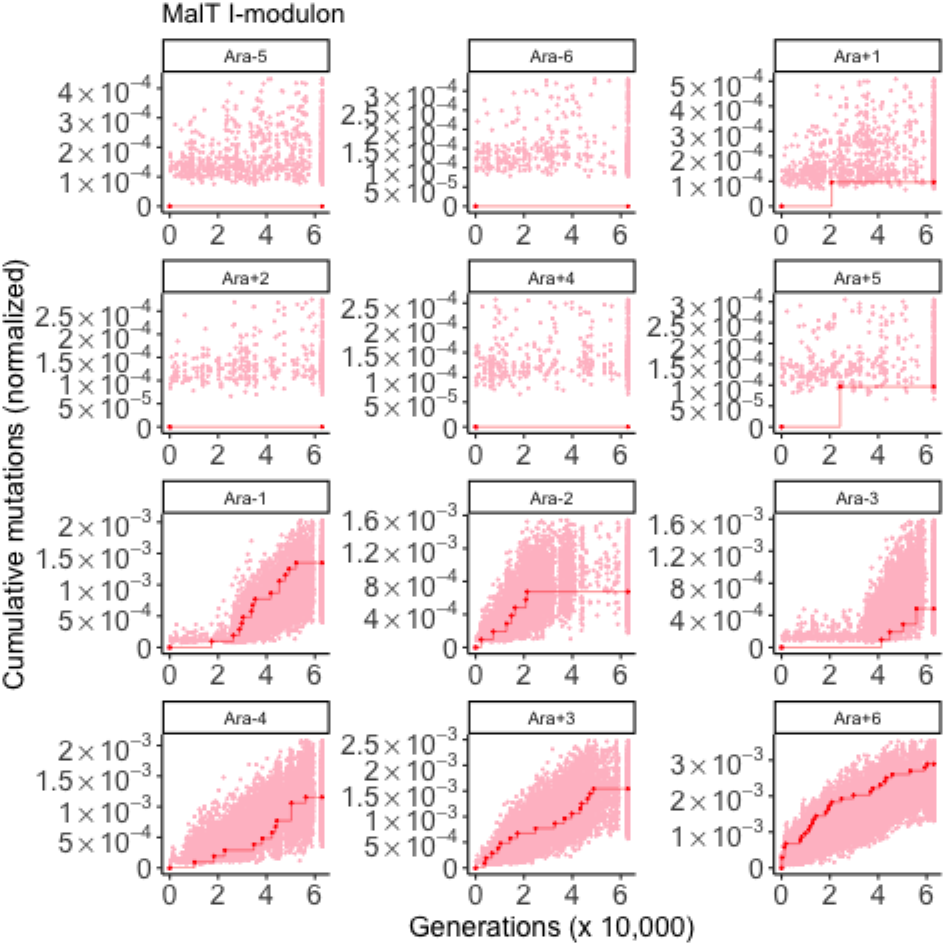
MalT I-modulon
Ara-5
Ara-6
Ara+1
Ara+2
Ara+4
Ara+5
Ara-1
Ara-2
Ara-3
Ara-4
Ara+3
Ara+6
Cumulative mutations (normalized)
Generations (x 10,000)

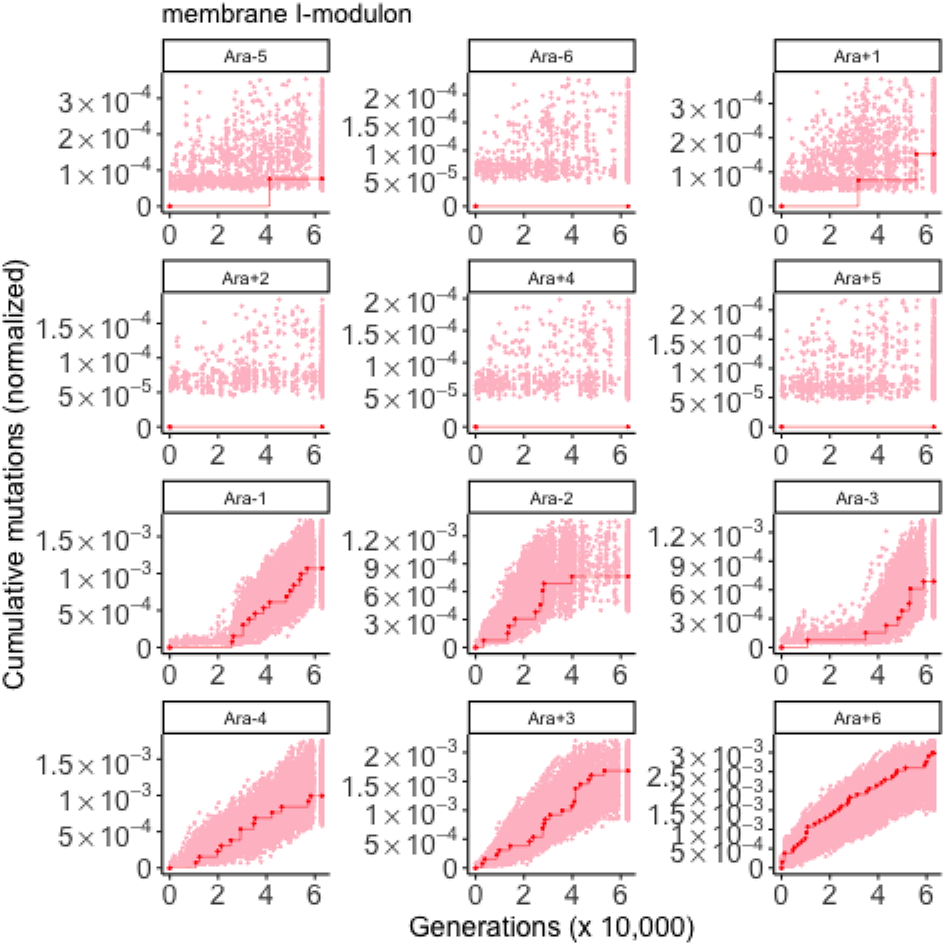

membrane I-modulon
Ara-5
Ara-6
Ara+1
Ara+2
Ara+4
Ara+5
Ara-1
Ara-2
Ara-3
Ara-4
Ara+3
Ara+6
Cumulative mutations (normalized)
Generations (x 10,000)

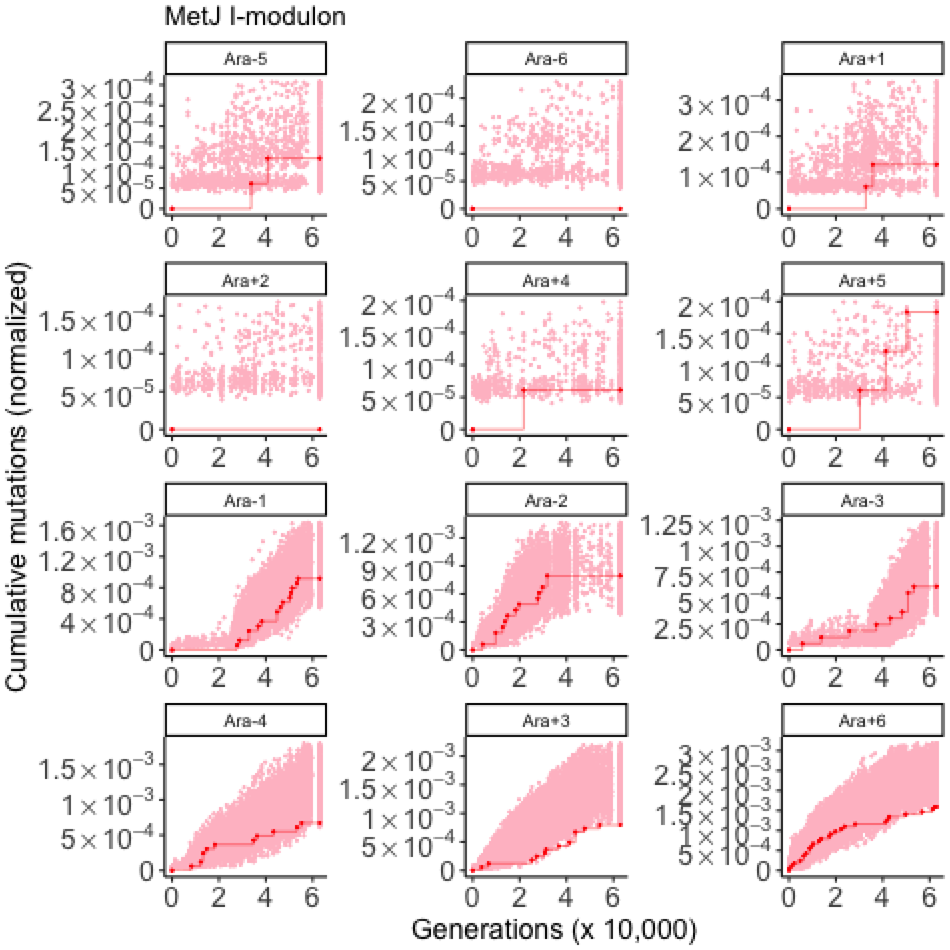

MetJ I-modulon
Ara-5
Ara-6
Ara+1
Ara+2
Ara+4
Ara+5
Ara-1
Ara-2
Ara-3
Ara-4
Ara+3
Ara+6
Cumulative mutations (normalized)
Generations (x 10,000)

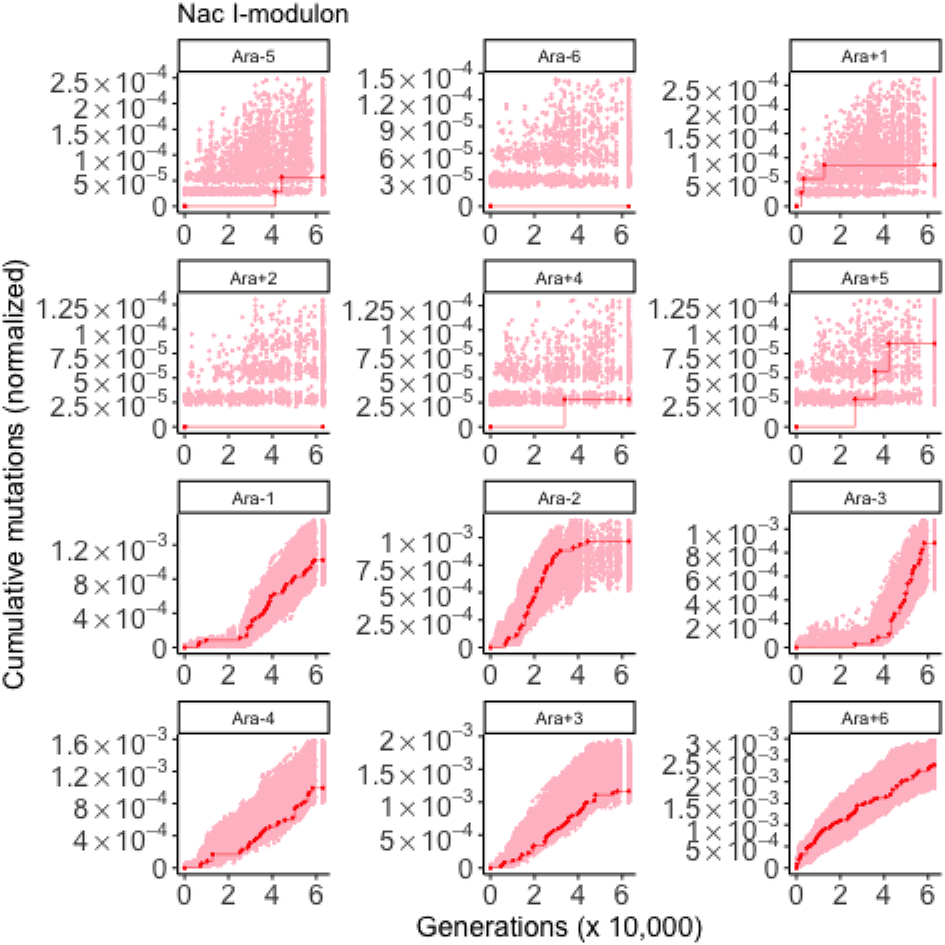
Nac I-modulon
Ara-5
Ara-6
Ara+1
Ara+2
Ara+4
Ara+5
Ara-1
Ara-2
Ara-3
Ara-4
Ara+3
Ara+6
Cumulative mutations (normalized)
Generations (x 10,000)

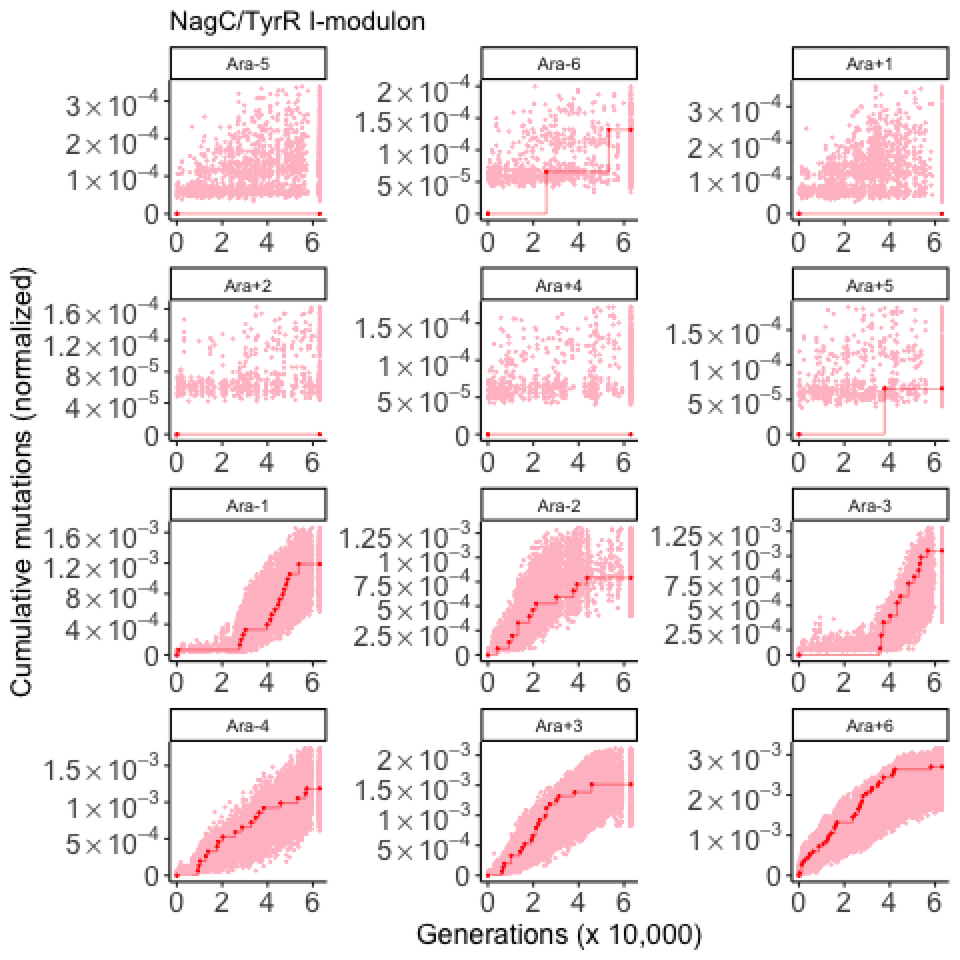
NagC/TyrR I-modulon
Ara-5
Ara-6
Ara+1
Ara+2
Ara+4
Ara+5
Ara-1
Ara-2
Ara-3
Ara-4
Ara+3
Ara+6
Cumulative mutations (normalized)
Generations (x 10,000)

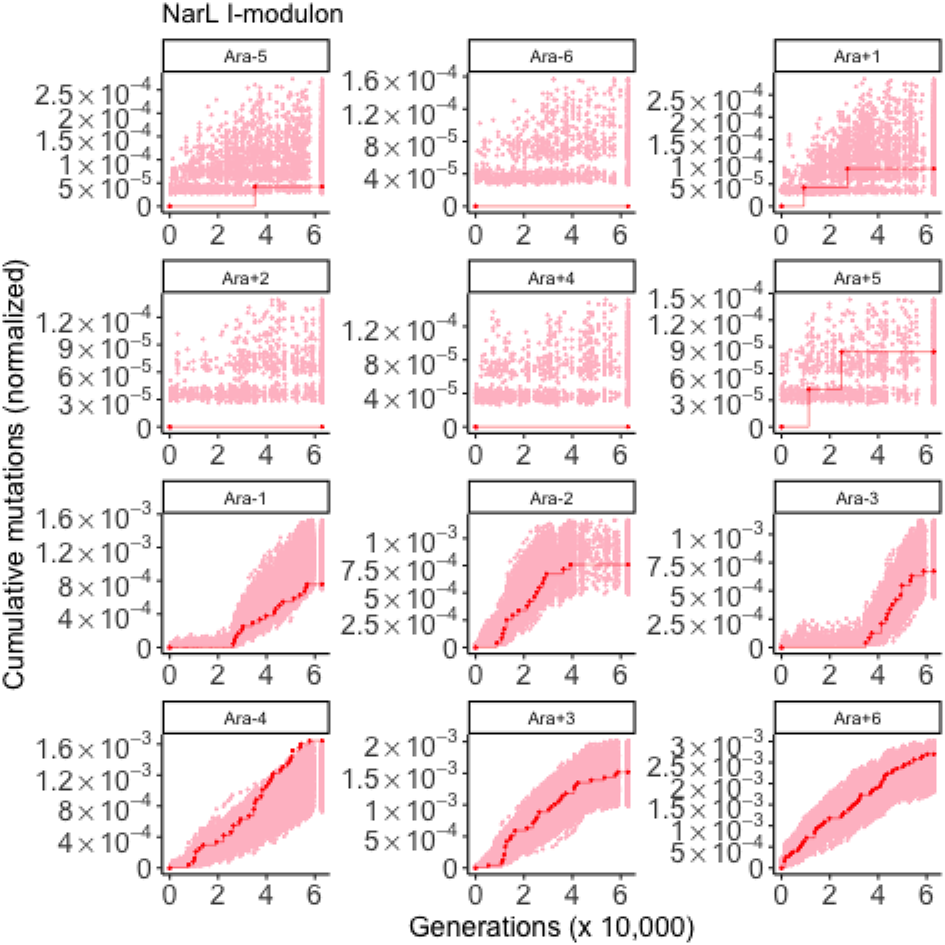
NarL I-modulon
Ara-5
Ara-6
Ara+1
Ara+2
Ara+4
Ara+5
Ara-1
Ara-2
Ara-3
Ara-4
Ara+3
Ara+6
Cumulative mutations (normalized)
Generations (x 10,000)

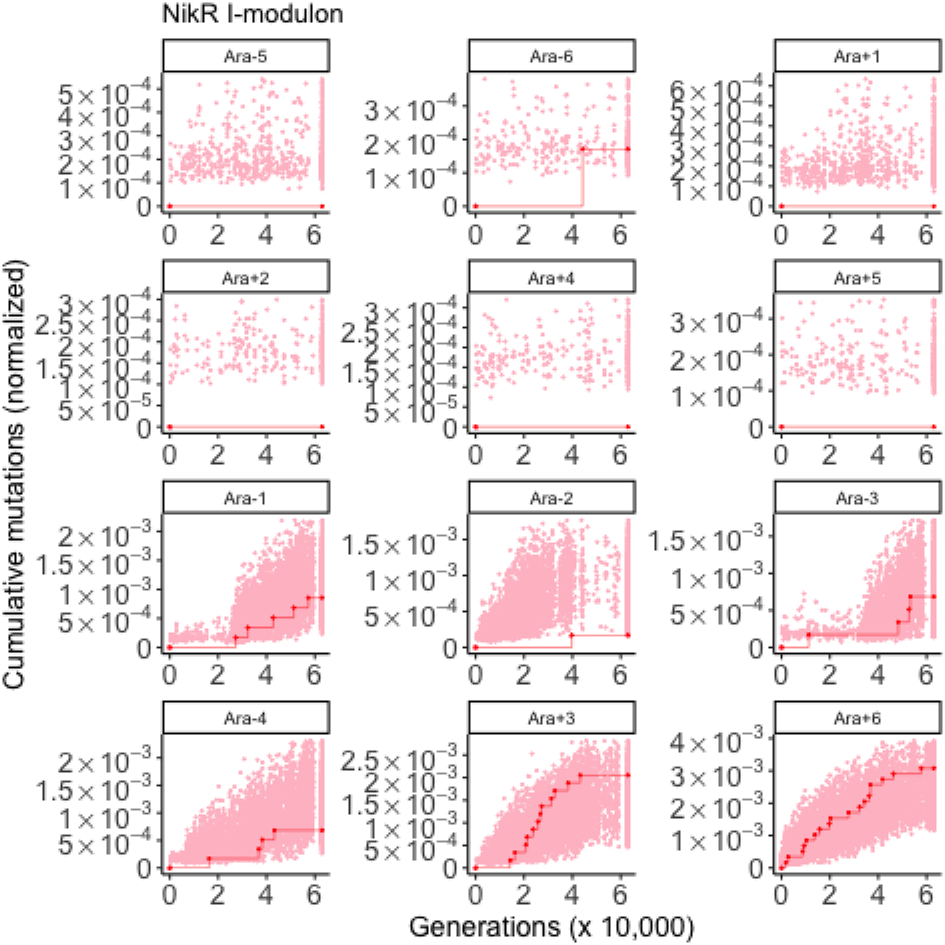
NikR I-modulon
Ara-5
Ara-6
Ara+1
Ara+2
Ara+4
Ara+5
Ara-1
Ara-2
Ara-3
Ara-4
Ara+3
Ara+6
Cumulative mutations (normalized)
Generations (x 10,000)

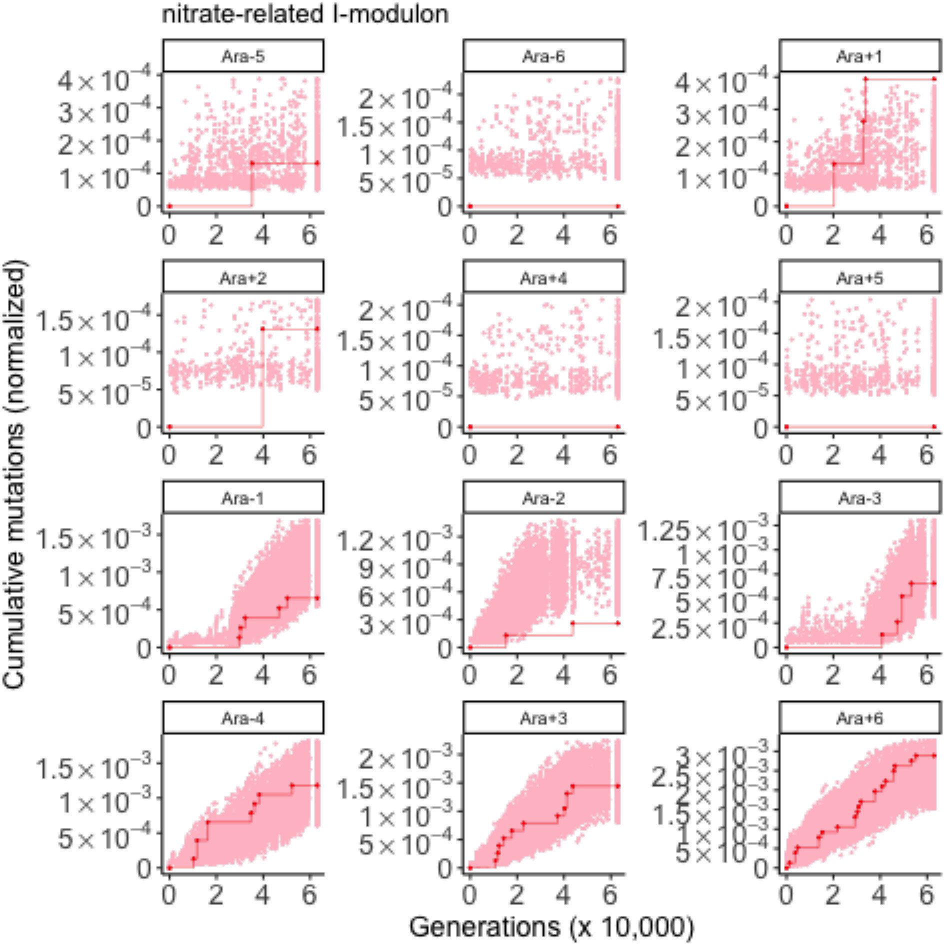

nitrate-related I-modulon
Ara-5
Ara-6
Ara+1
Ara+2
Ara+4
Ara+5
Ara-1
Ara-2
Ara-3
Ara-4
Ara+3
Ara+6
Cumulative mutations (normalized)
Generations (x 10,000)

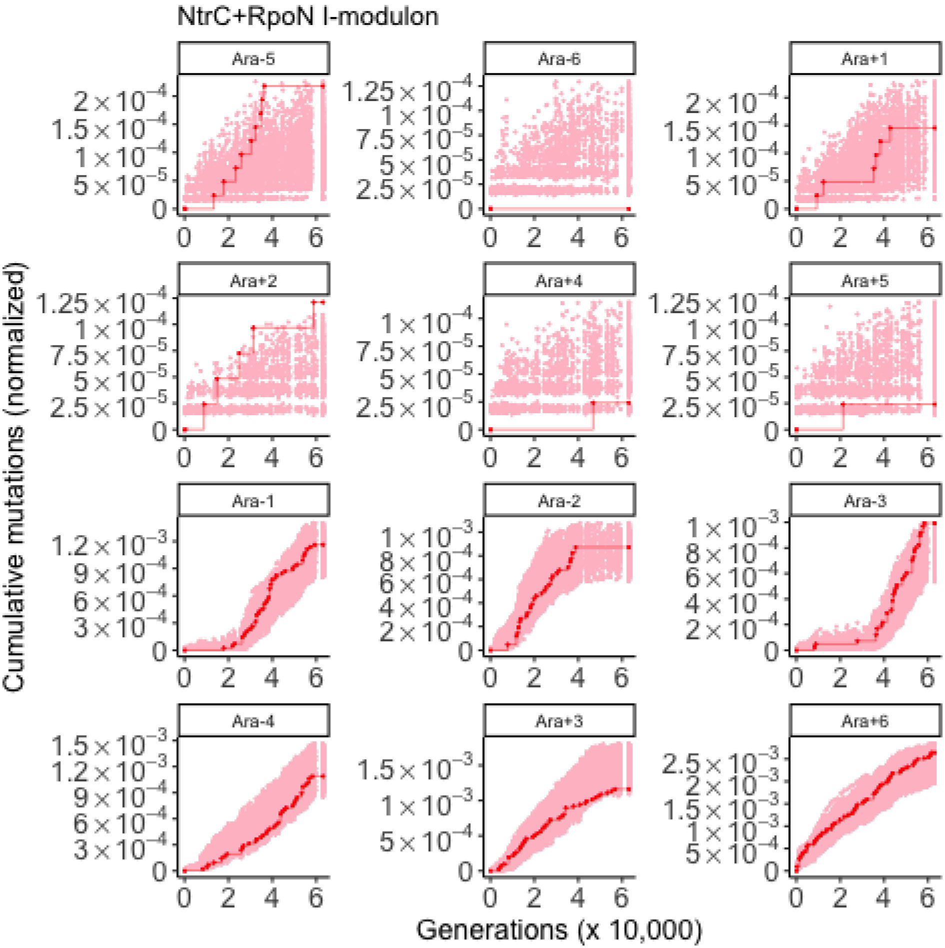

NtrC+RpoN I-modulon
Ara-5
Ara-6
Ara+1
Ara+2
Ara+4
Ara+5
Ara-1
Ara-2
Ara-3
Ara-4
Ara+3
Ara+6
Cumulative mutations (normalized)
Generations (x 10,000)

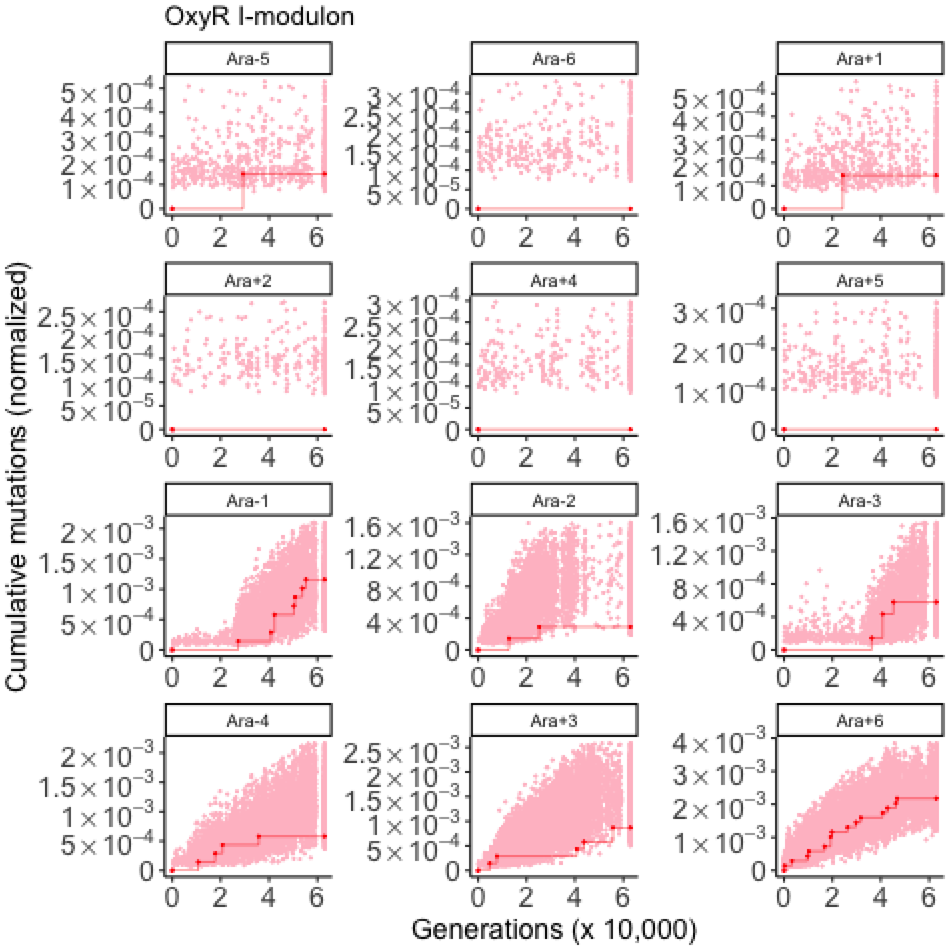
OxyR I-modulon
Ara-5
Ara-6
Ara+1
Ara+2
Ara+4
Ara+5
Ara-1
Ara-2
Ara-3
Ara-4
Ara+3
Ara+6
Cumulative mutations (normalized)
Generations (x 10,000)

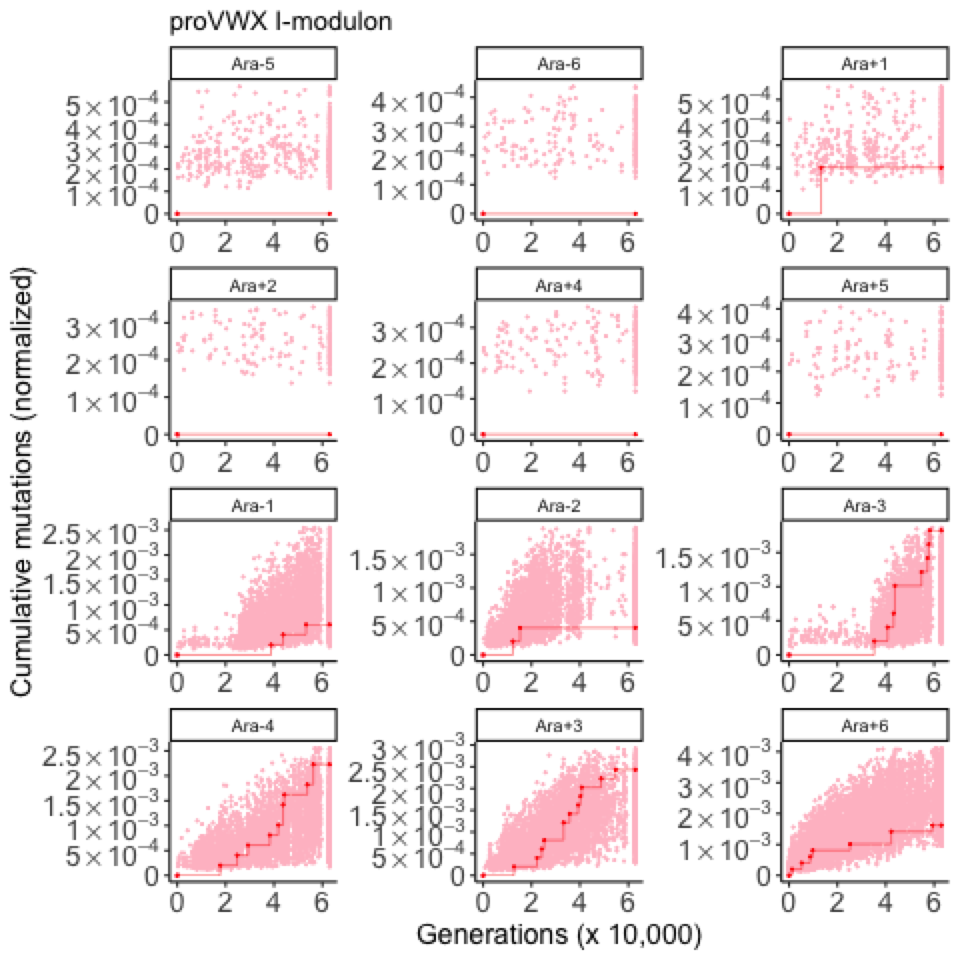

proVWX I-modulon
Ara-5
Ara-6
Ara+1
Ara+2
Ara+4
Ara+5
Ara-1
Ara-2
Ara-3
Ara-4
Ara+3
Ara+6
Cumulative mutations (normalized)
Generations (x 10,000)

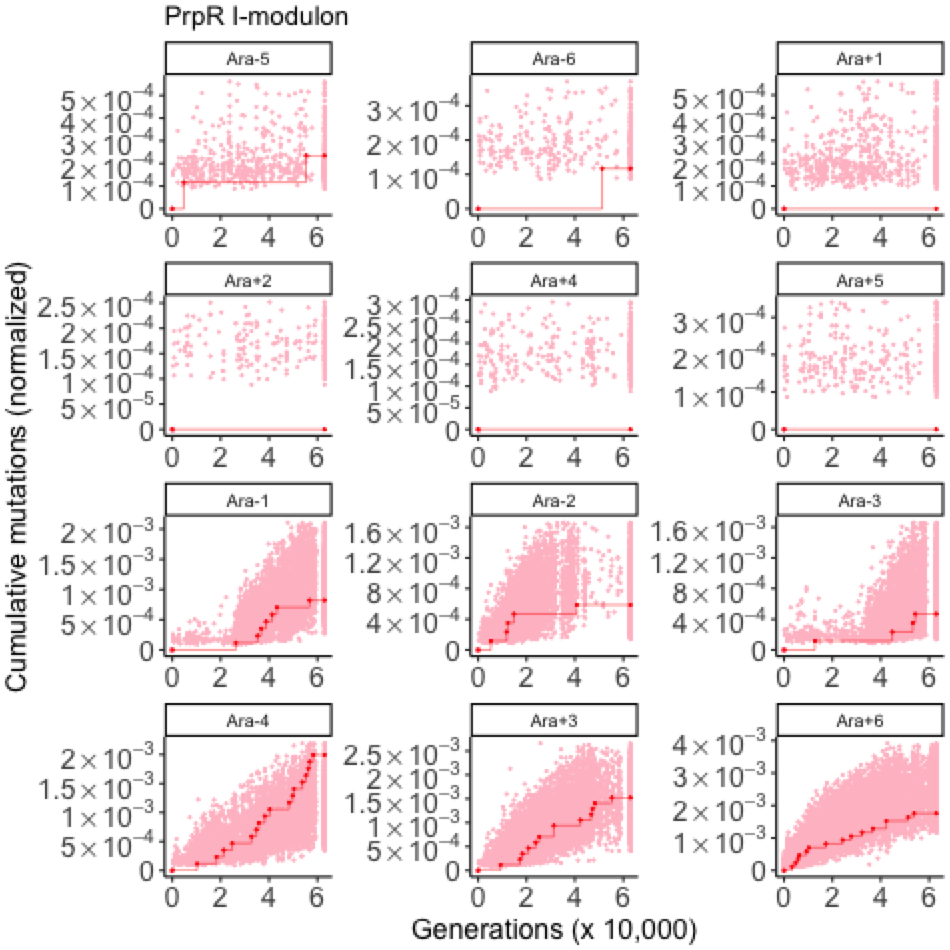
PrpR I-modulon
Ara-5
Ara-6
Ara+1
Ara+2
Ara+4
Ara+5
Ara-1
Ara-2
Ara-3
Ara-4
Ara+3
Ara+6
Cumulative mutations (normalized)
Generations (x 10,000)

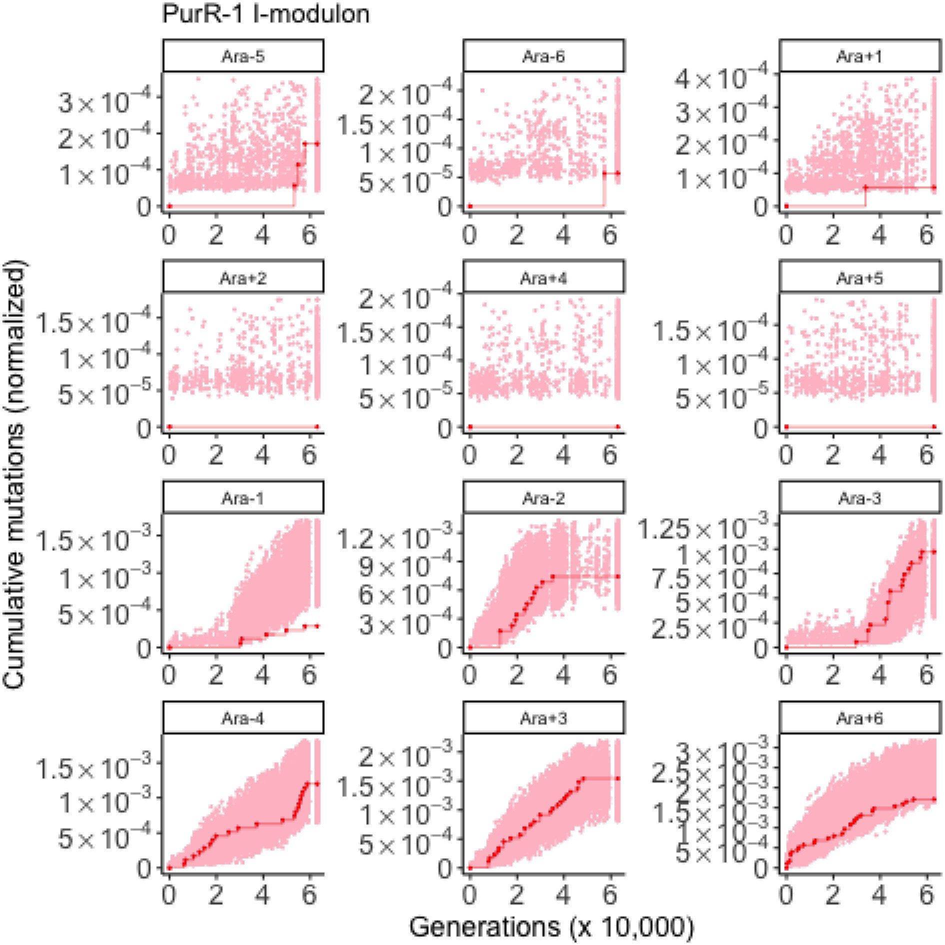

PurR-1 I-modulon
Ara-5
Ara-6
Ara+1
Ara+2
Ara+4
Ara+5
Ara-1
Ara-2
Ara-3
Ara-4
Ara+3
Ara+6
Cumulative mutations (normalized)
Generations (x 10,000)

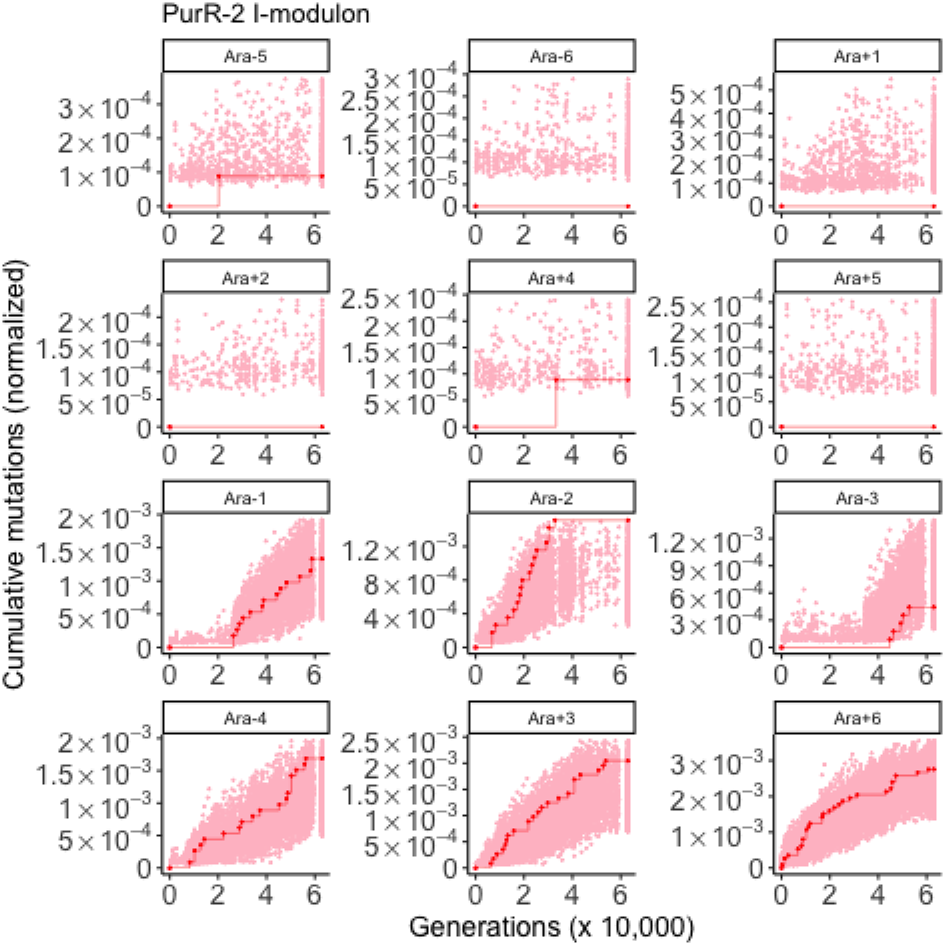
PurR-2 I-modulon
Ara-5
Ara-6
Ara+1
Ara+2
Ara+4
Ara+5
Ara-1
Ara-2
Ara-3
Ara-4
Ara+3
Ara+6
Cumulative mutations (normalized)
Generations (x 10,000)

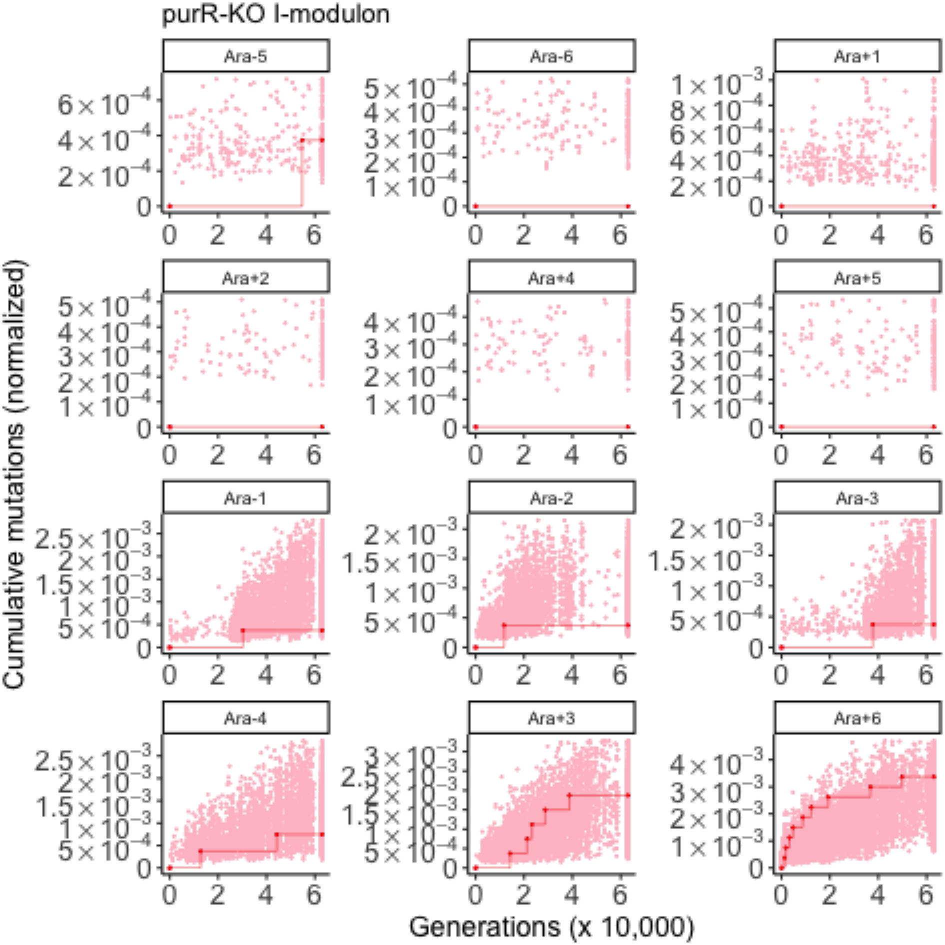

purR-KO I-modulon
Ara-5
Ara-6
Ara+1
Ara+2
Ara+4
Ara+5
Ara-1
Ara-2
Ara-3
Ara-4
Ara+3
Ara+6
Cumulative mutations (normalized)
Generations (x 10,000)

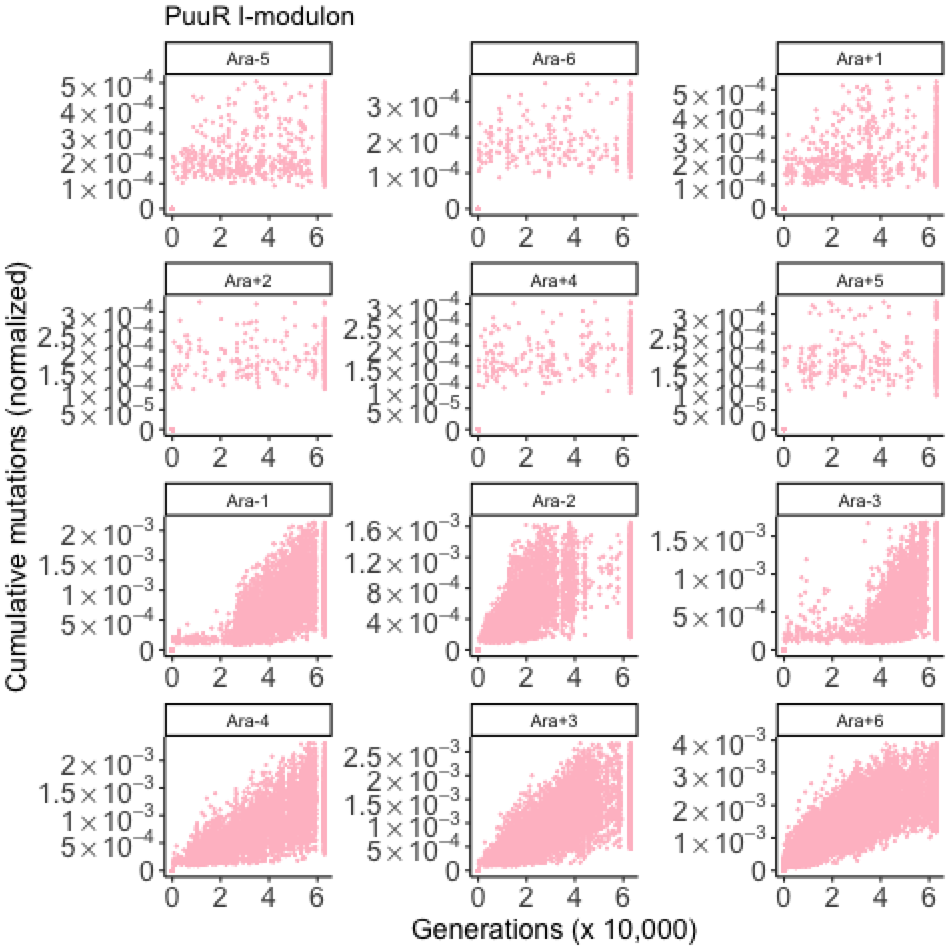
PuuR I-modulon
Ara-5
Ara-6
Ara+1
Ara+2
Ara+4
Ara+5
Ara-1
Ara-2
Ara-3
Ara-4
Ara+3
Ara+6
Cumulative mutations (normalized)
Generations (x 10,000)

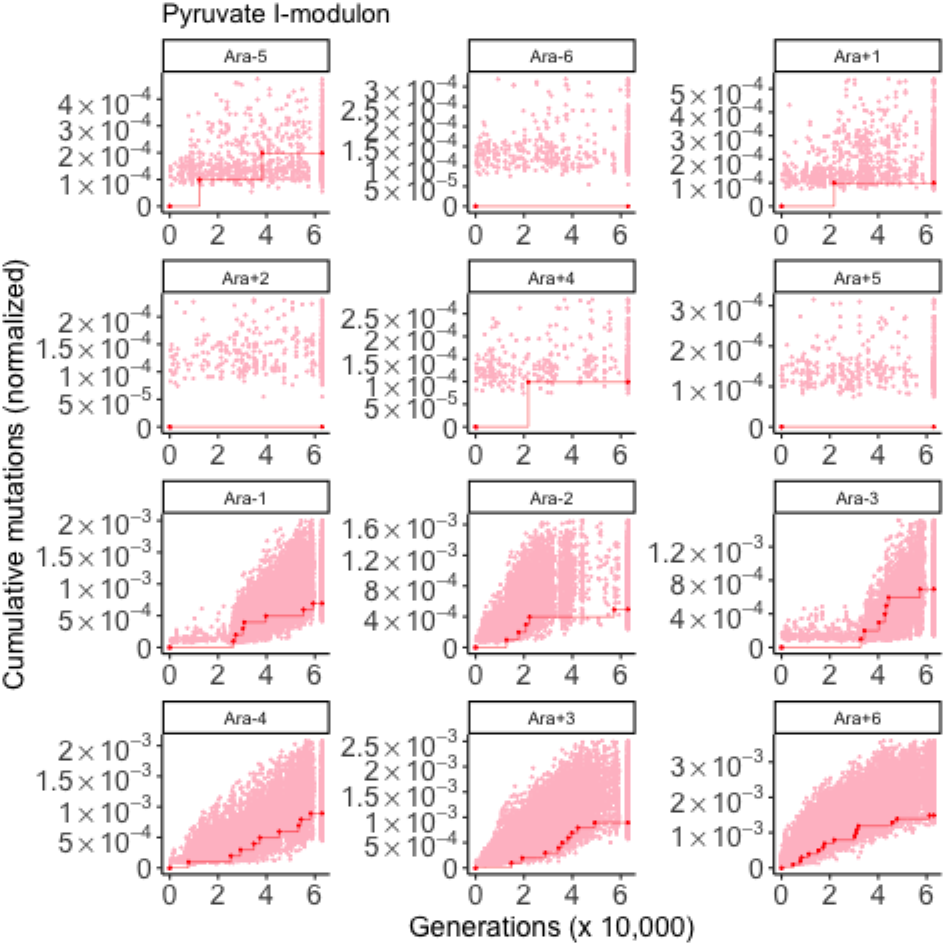
Pyruvate I-modulon
Ara-5
Ara-6
Ara+1
Ara+2
Ara+4
Ara+5
Ara-1
Ara-2
Ara-3
Ara-4
Ara+3
Ara+6
Cumulative mutations (normalized)
Generations (x 10,000)

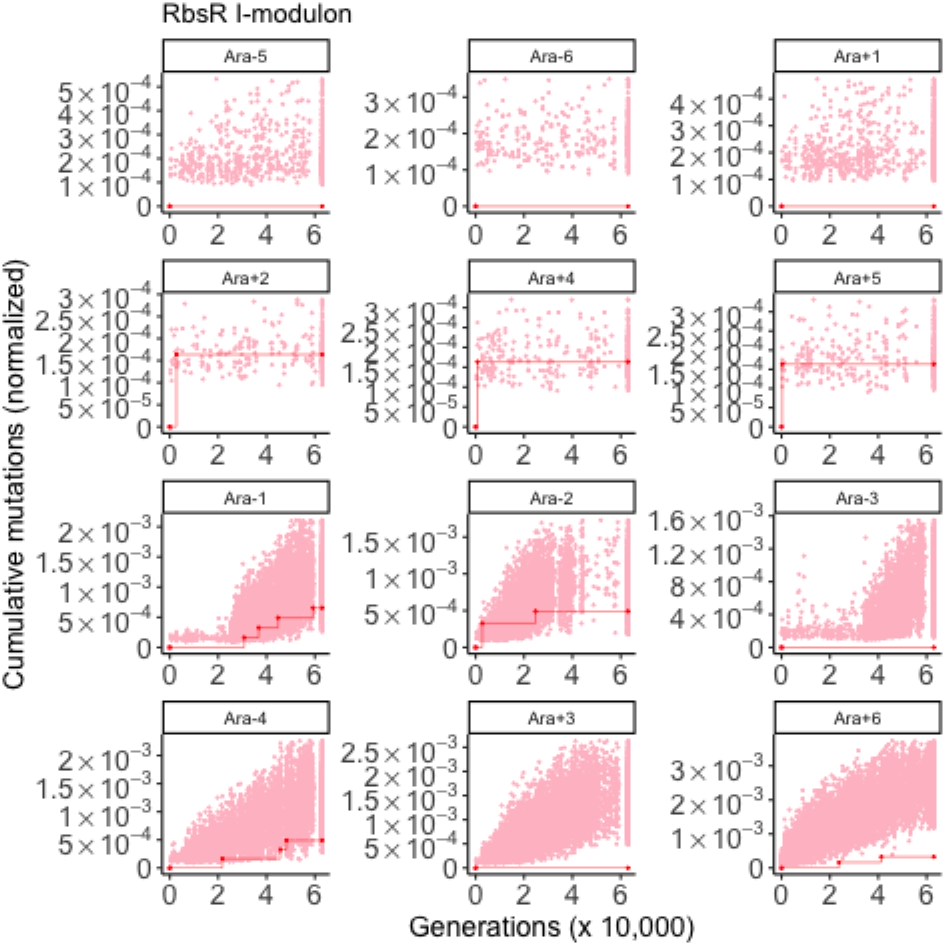
RbsR I-modulon
Ara-5
Ara-6
Ara+1
Ara+2
Ara+4
Ara+5
Ara-1
Ara-2
Ara-3
Ara-4
Ara+3
Ara+6
Cumulative mutations (normalized)
Generations (x 10,000)

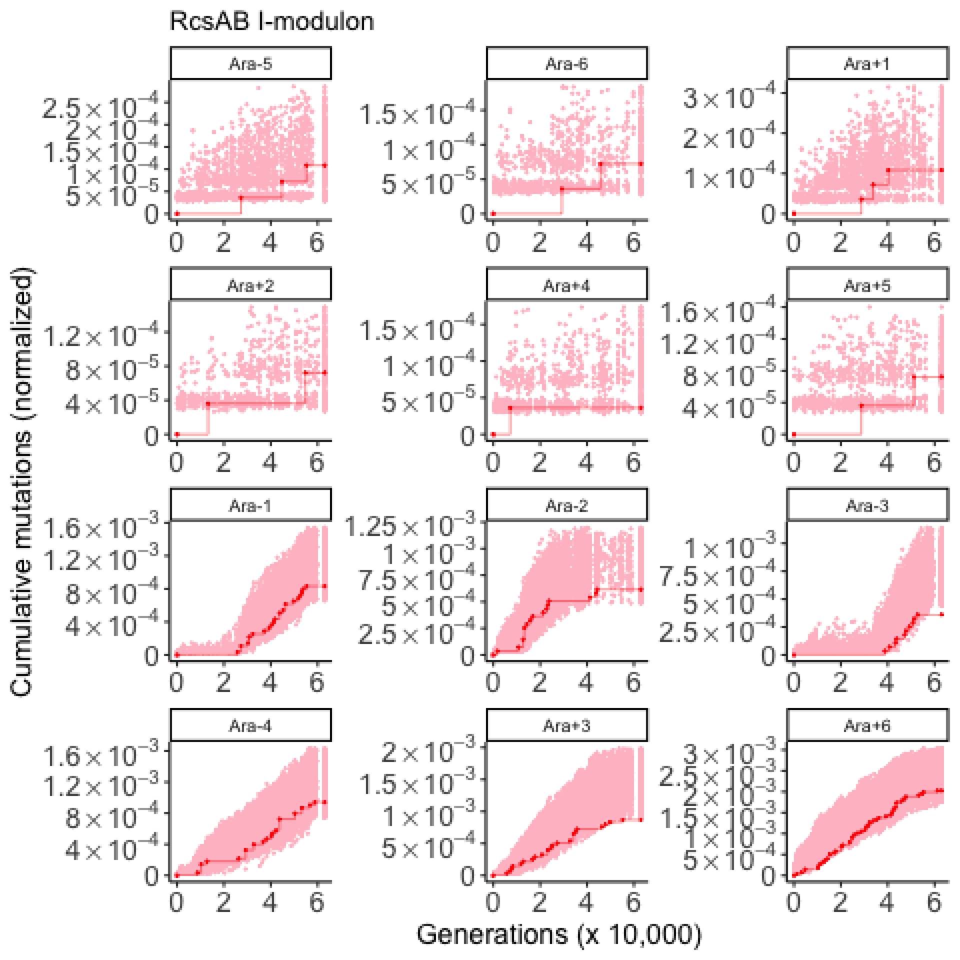

RcsAB I-modulon
Ara-5
Ara-6
Ara+1
Ara+2
Ara+4
Ara+5
Ara-1
Ara-2
Ara-3
Ara-4
Ara+3
Ara+6
Cumulative mutations (normalized)
Generations (x 10,000)

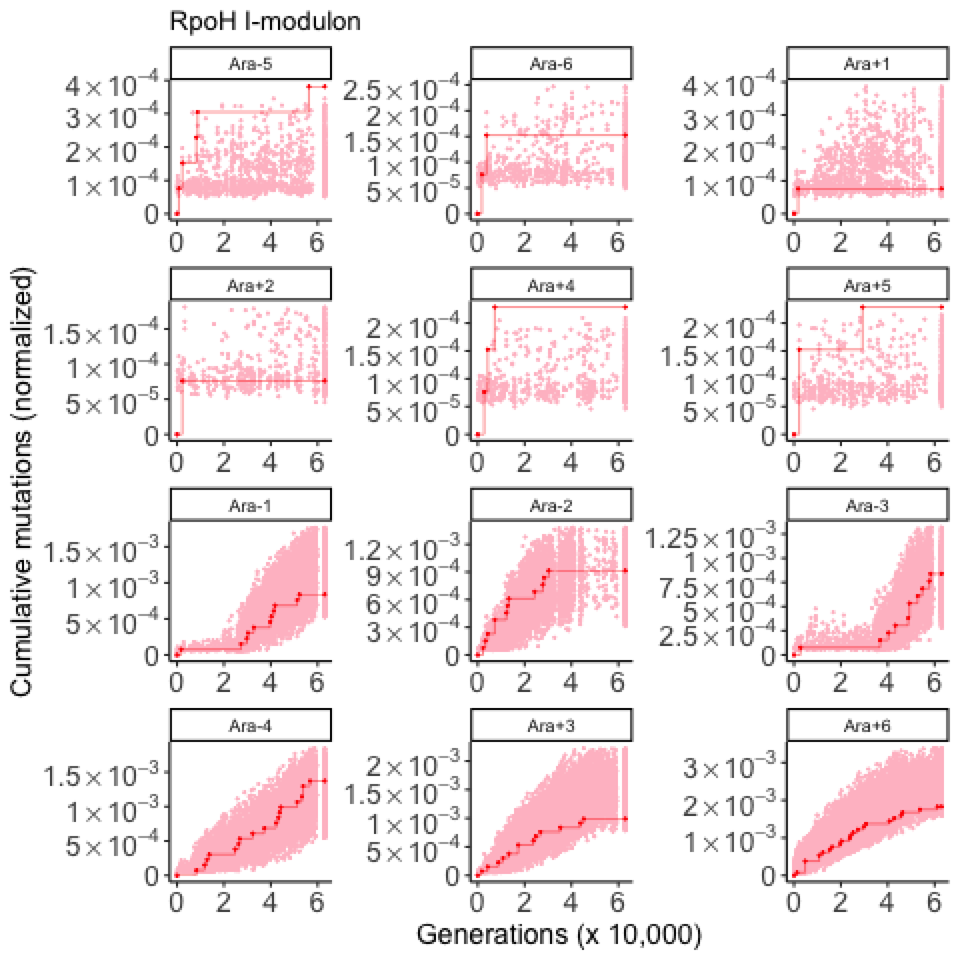
RpoH I-modulon
Ara-5
Ara-6
Ara+1
Ara+2
Ara+4
Ara+5
Ara-1
Ara-2
Ara-3
Ara-4
Ara+3
Ara+6
Cumulative mutations (normalized)
Generations (x 10,000)

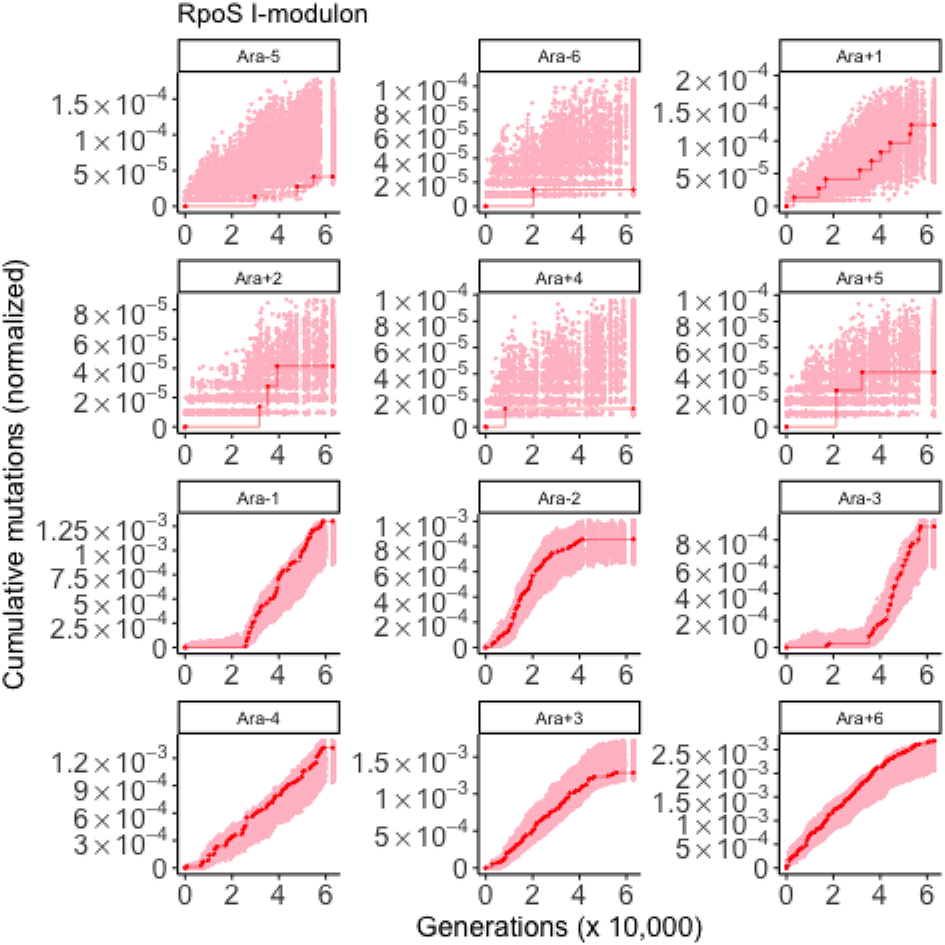
RpoS I-modulon
Ara-5
Ara-6
Ara+1
Ara+2
Ara+4
Ara+5
Ara-1
Ara-2
Ara-3
Ara-4
Ara+3
Ara+6
Cumulative mutations (normalized)
Generations (x 10,000)

sgrT I-modulon

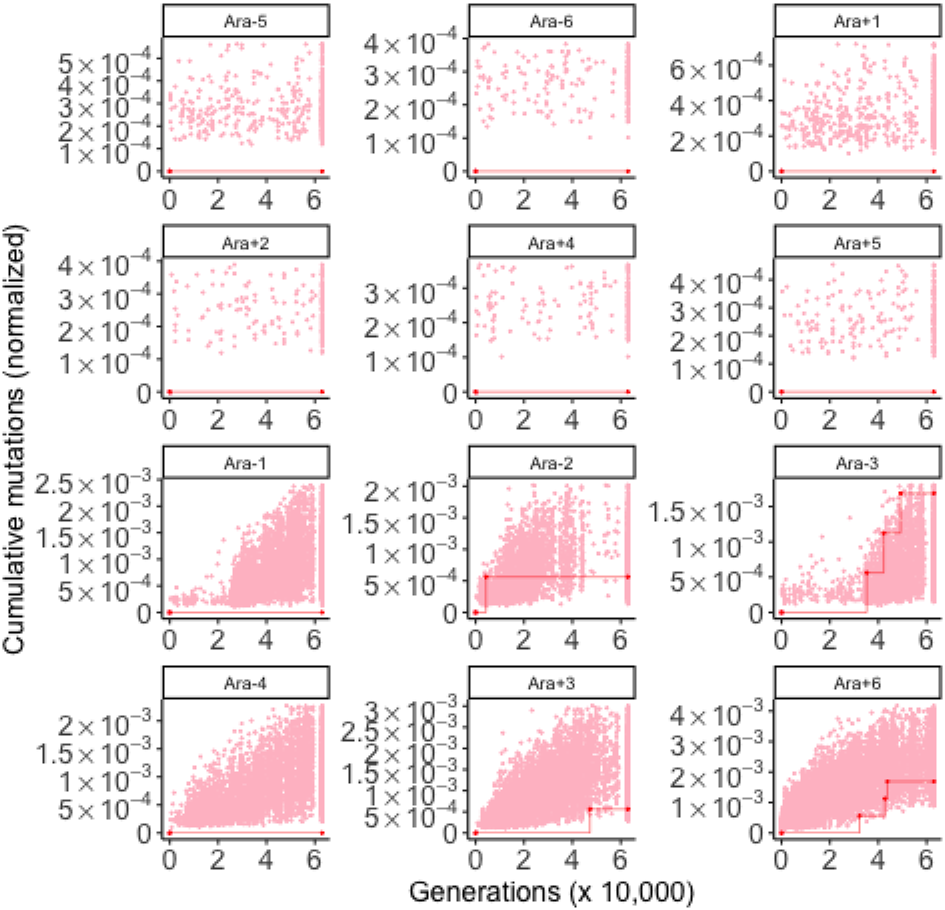

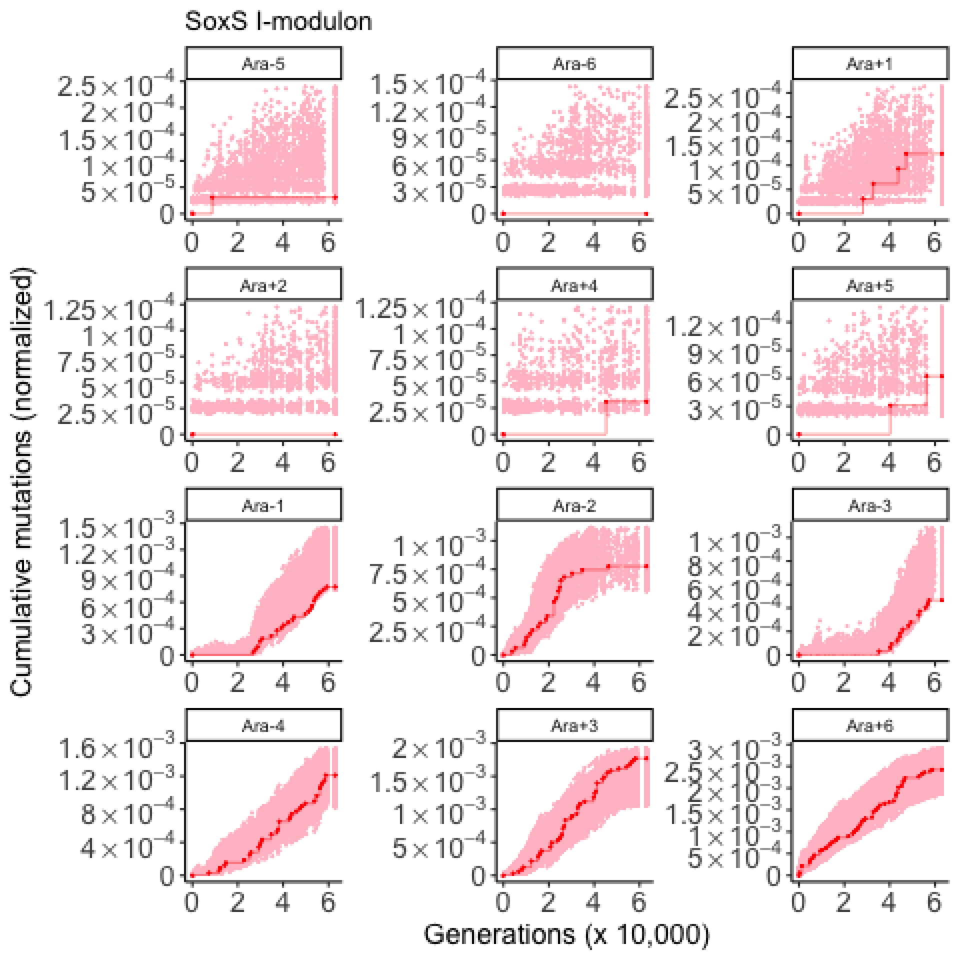

SoxS I-modulon
Ara-5
Ara-6
Ara+1
Ara+2
Ara+4
Ara+5
Ara-1
Ara-2
Ara-3
Ara-4
Ara+3
Ara+6
Cumulative mutations (normalized)
Generations (x 10,000)

SrlR+GutM I-modulon

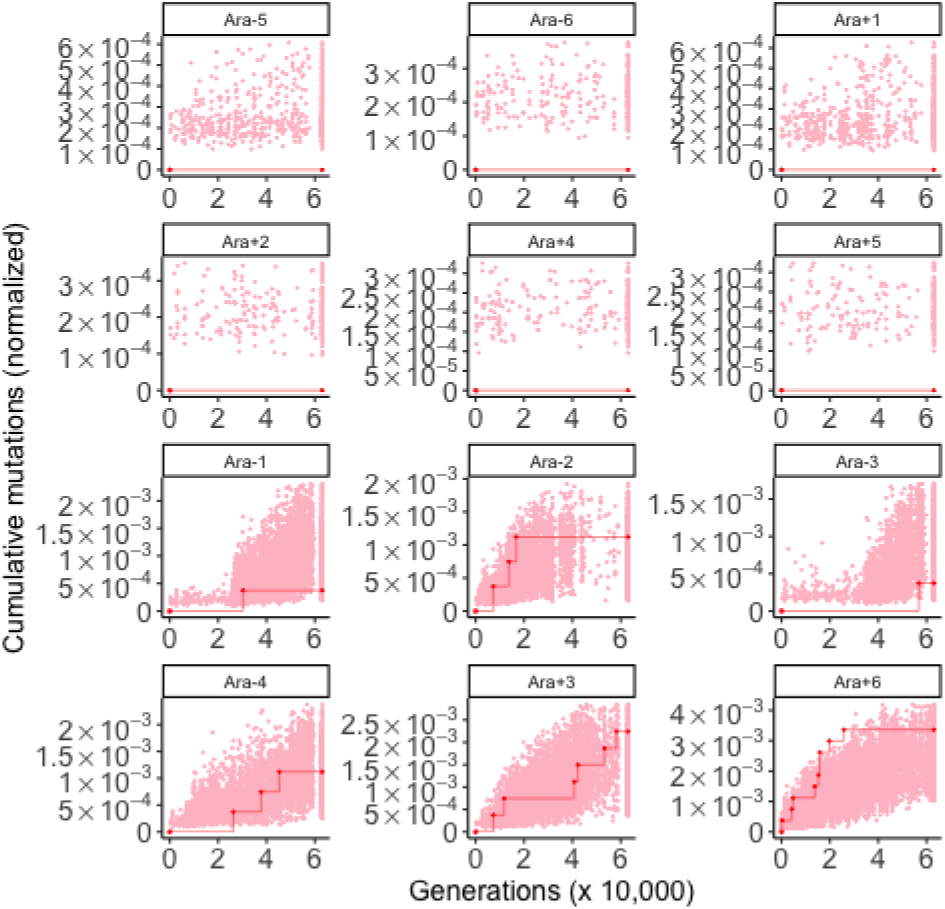

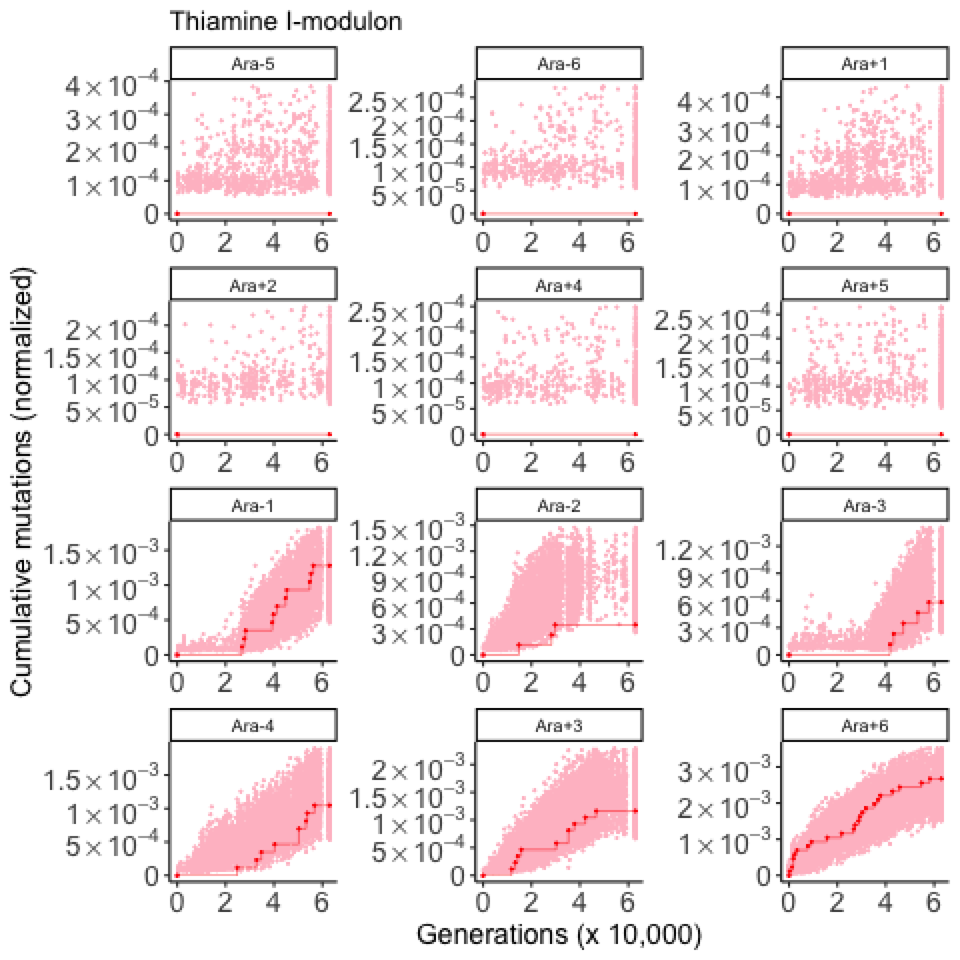
Thiamine I-modulon
Ara-5
Ara-6
Ara+1
Ara+2
Ara+4
Ara+5
Ara-1
Ara-2
Ara-3
Ara-4
Ara+3
Ara+6
Cumulative mutations (normalized)
Generations (x 10,000)

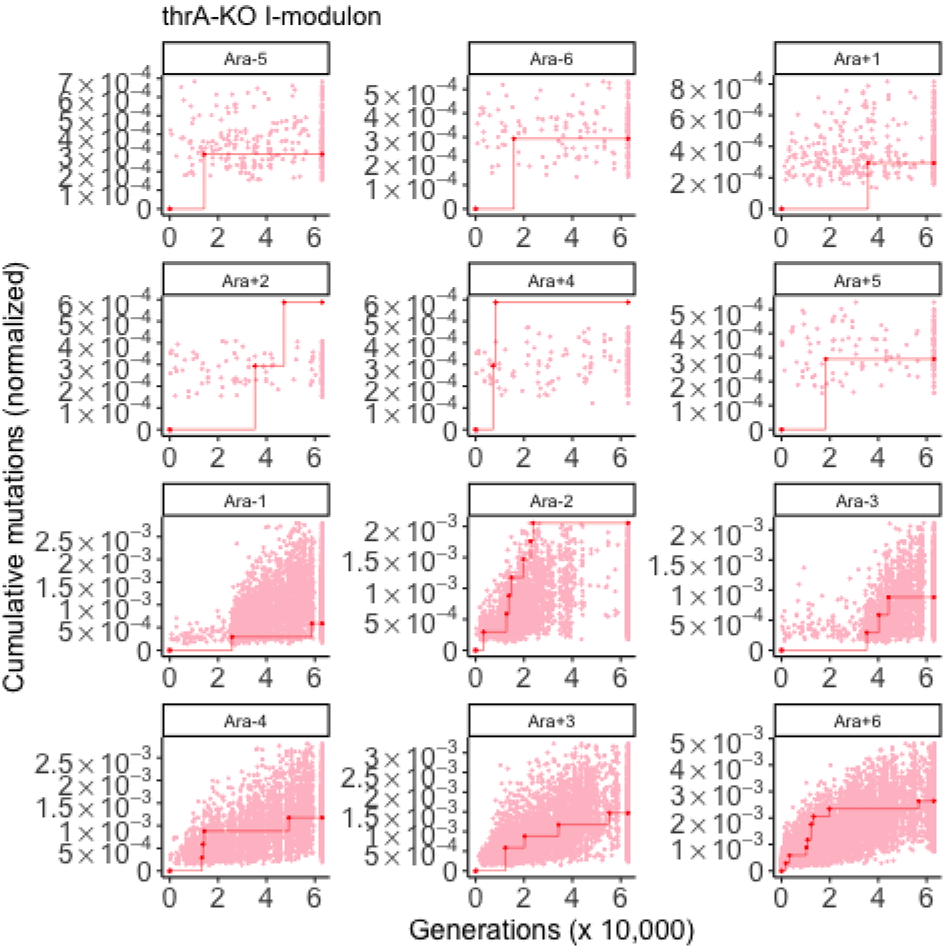

thrA-KO I-modulon
Ara-5
Ara-6
Ara+1
Ara+2
Ara+4
Ara+5
Ara-1
Ara-2
Ara-3
Ara-4
Ara+3
Ara+6
Cumulative mutations (normalized)
Generations (x 10,000)

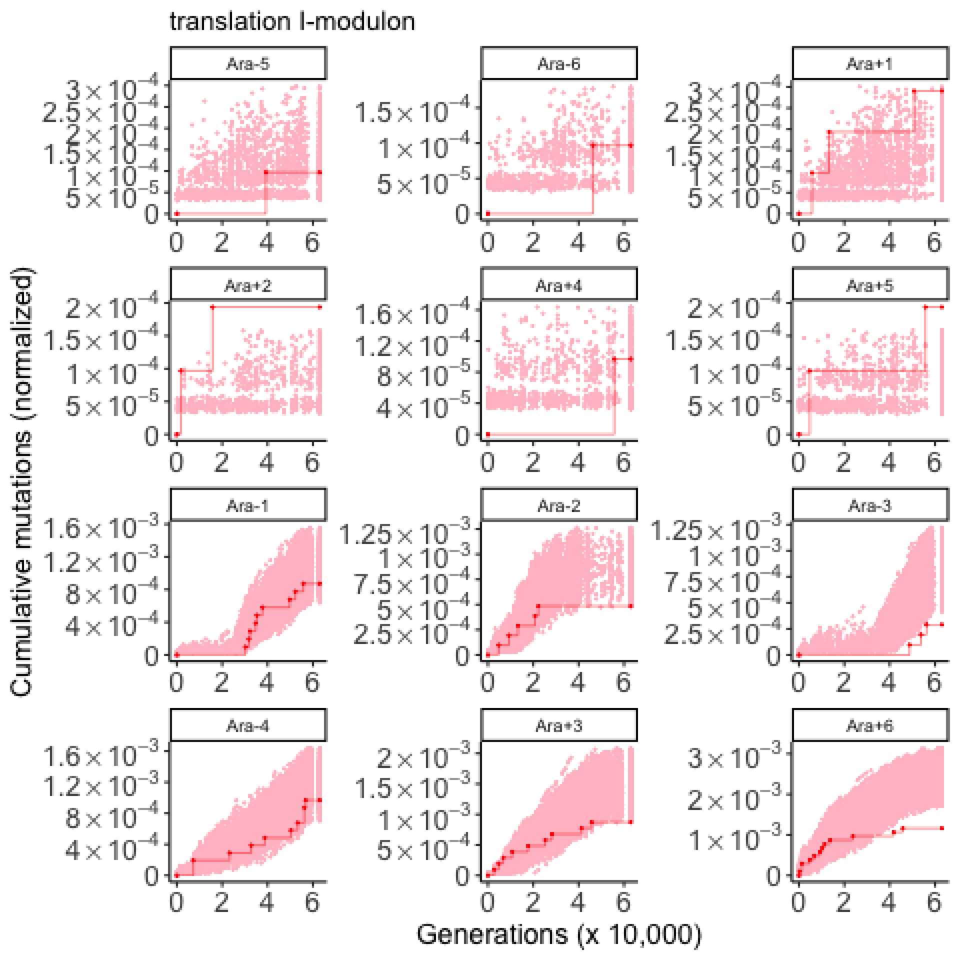

translation I-modulon
Ara-5
Ara-6
Ara+1
Ara+2
Ara+4
Ara+5
Ara-1
Ara-2
Ara-3
Ara-4
Ara+3
Ara+6
Cumulative mutations (normalized)
Generations (x 10,000)

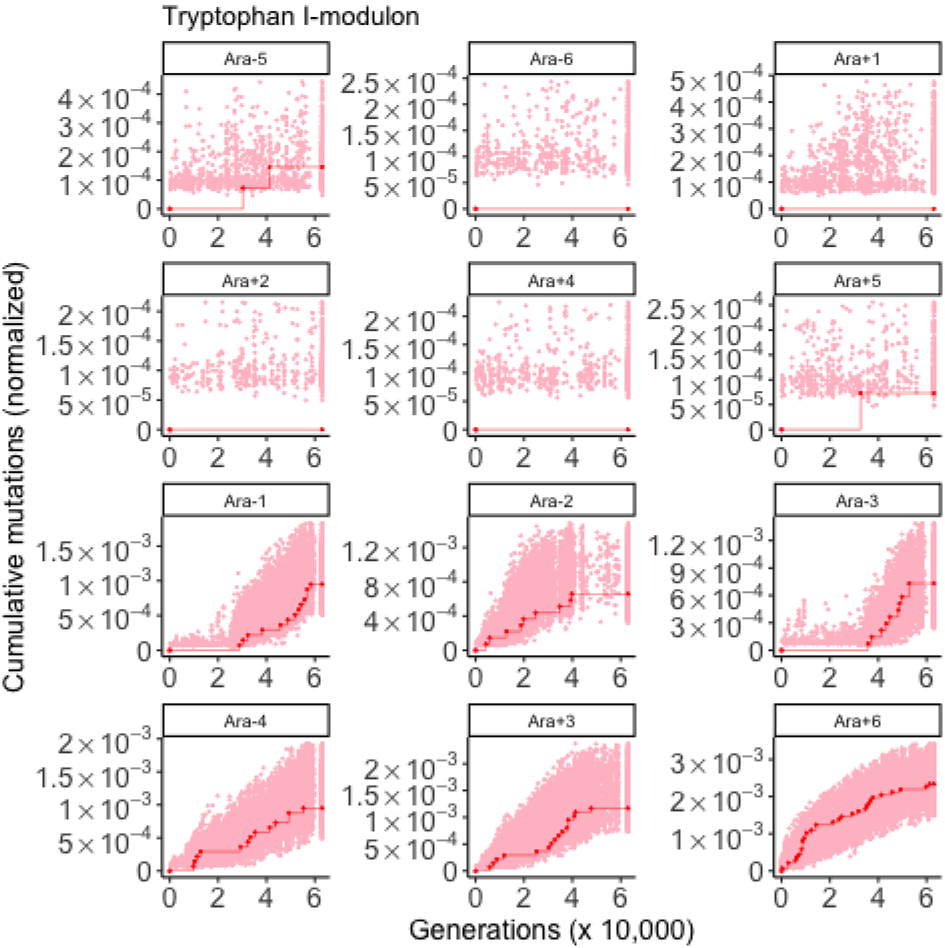

Tryptophan I-modulon
Ara-5
Ara-6
Ara+1
Ara+2
Ara+4
Ara+5
Ara-1
Ara-2
Ara-3
Ara-4
Ara+3
Ara+6
Cumulative mutations (normalized)
Generations (x 10,000)

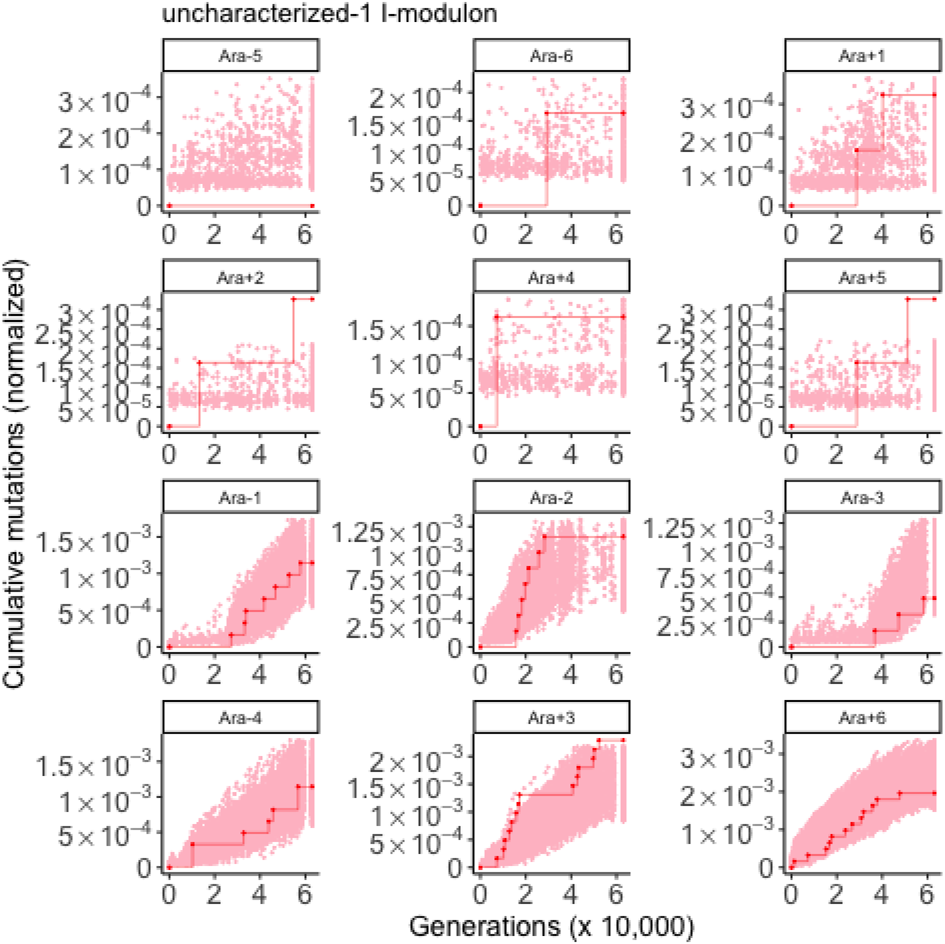
uncharacterized-1 I-modulon
Ara-5
Ara-6
Ara+1
Ara+2
Ara+4
Ara+5
Ara-1
Ara-2
Ara-3
Ara-4
Ara+3
Ara+6
Cumulative mutations (normalized)
Generations (x 10,000)

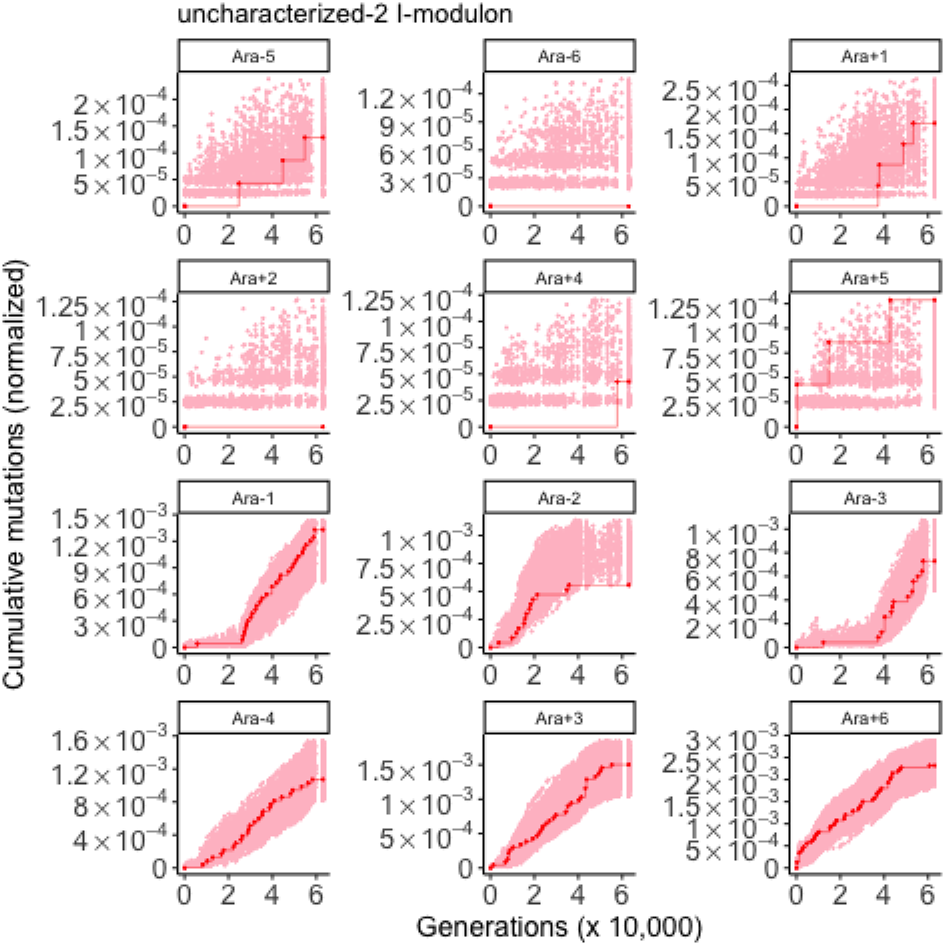
uncharacterized-2 I-modulon
Ara-5
Ara-6
Ara+1
Ara+2
Ara+4
Ara+5
Ara-1
Ara-2
Ara-3
Ara-4
Ara+3
Ara+6
Cumulative mutations (normalized)
Generations (x 10,000)

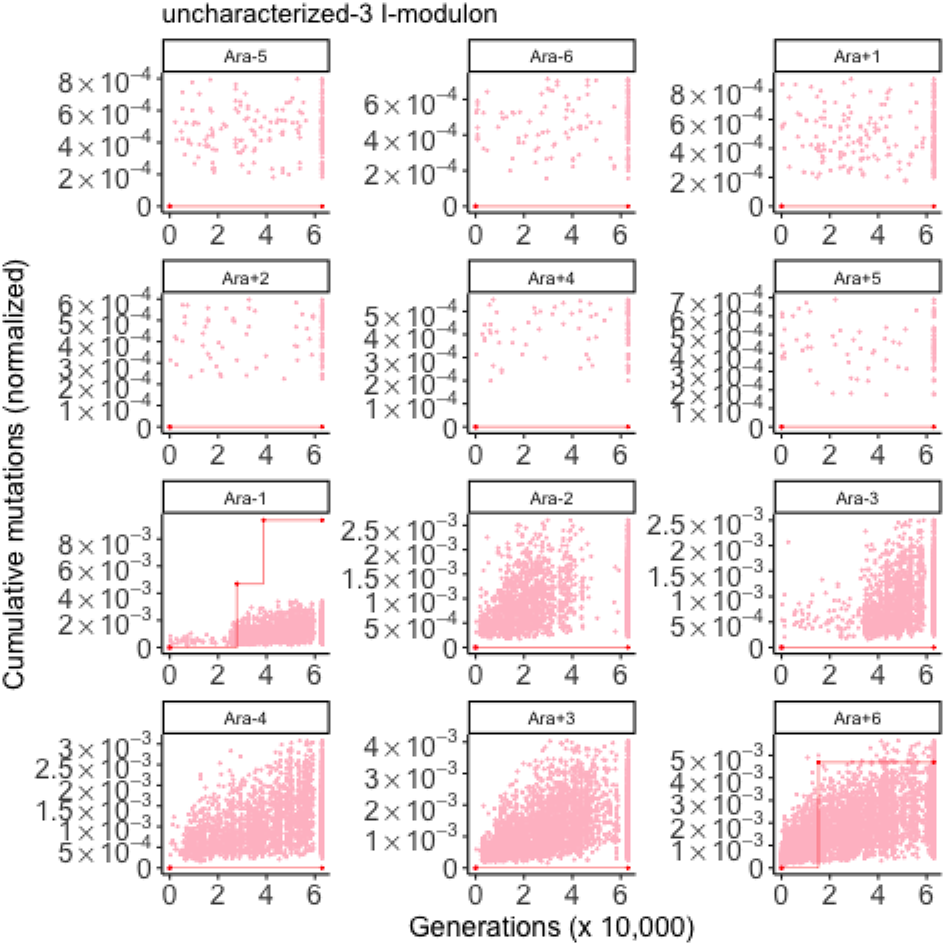
uncharacterized-3 I-modulon
Ara-5
Ara-6
Ara+1
Ara+2
Ara+4
Ara+5
Ara-1
Ara-2
Ara-3
Ara-4
Ara+3
Ara+6
Cumulative mutations (normalized)
Generations (x 10,000)

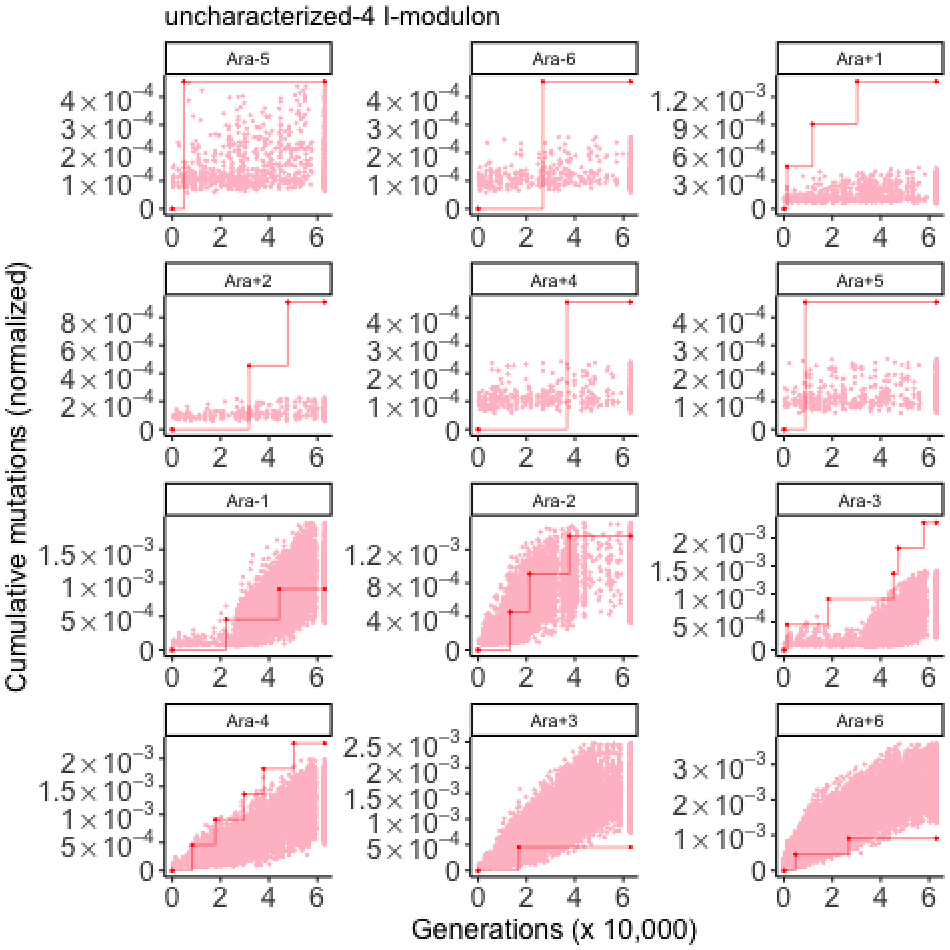

uncharacterized-4 I-modulon
Ara-5
Ara-6
Ara+1
Ara+2
Ara+4
Ara+5
Ara-1
Ara-2
Ara-3
Ara-4
Ara+3
Ara+6
Cumulative mutations (normalized)
Generations (x 10,000)

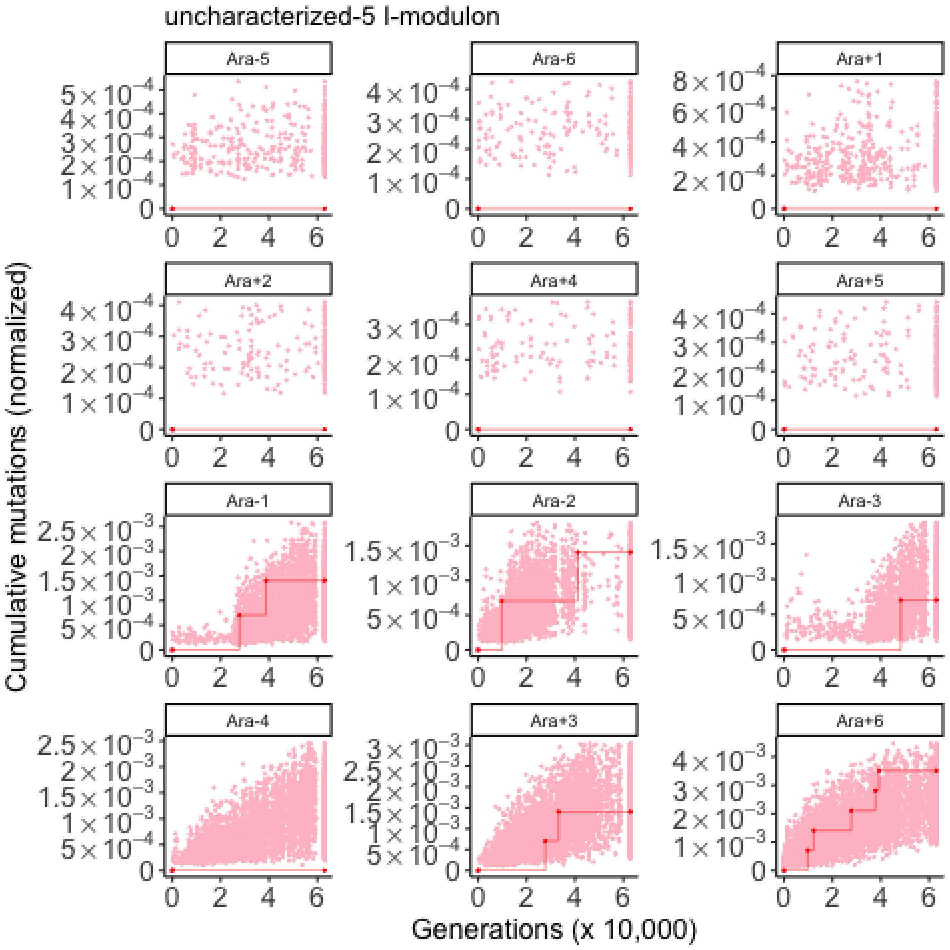
uncharacterized-5 I-modulon
Ara-5
Ara-6
Ara+1
Ara+2
Ara+4
Ara+5
Ara-1
Ara-2
Ara-3
Ara-4
Ara+3
Ara+6
Cumulative mutations (normalized)
Generations (x 10,000)

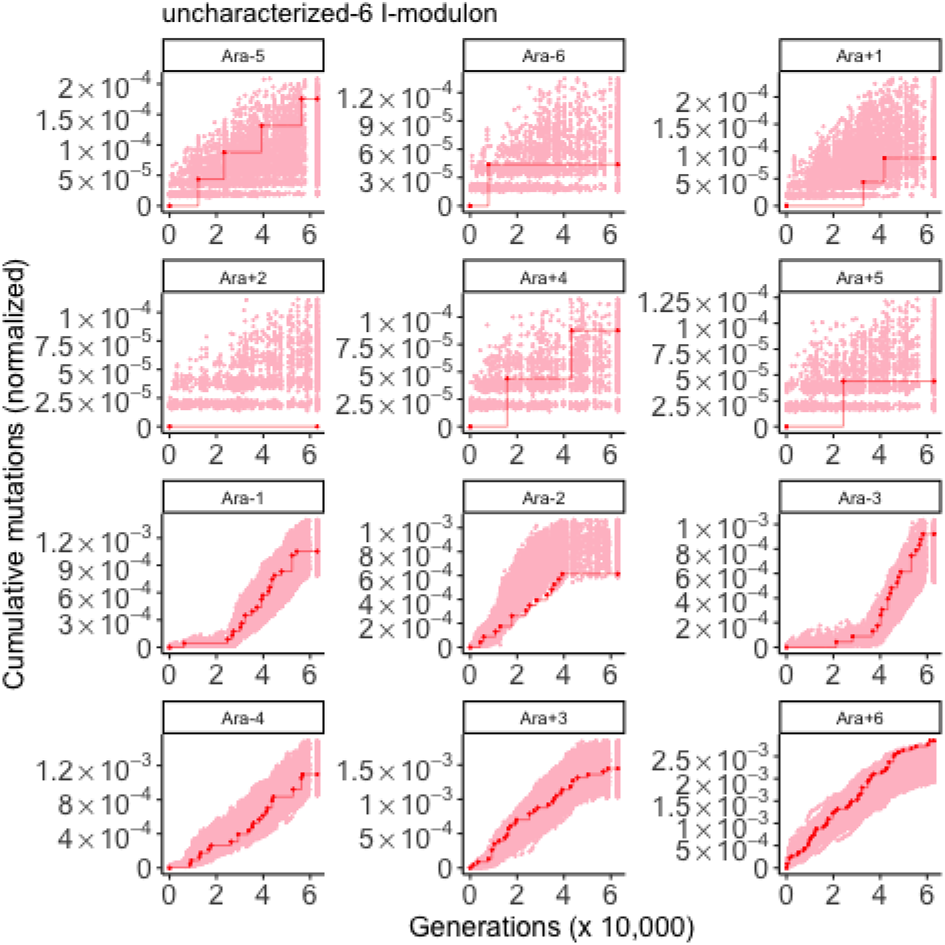

uncharacterized-6 I-modulon
Ara-5
Ara-6
Ara+1
Ara+2
Ara+4
Ara+5
Ara-1
Ara-2
Ara-3
Ara-4
Ara+3
Ara+6
Cumulative mutations (normalized)
Generations (x 10,000)

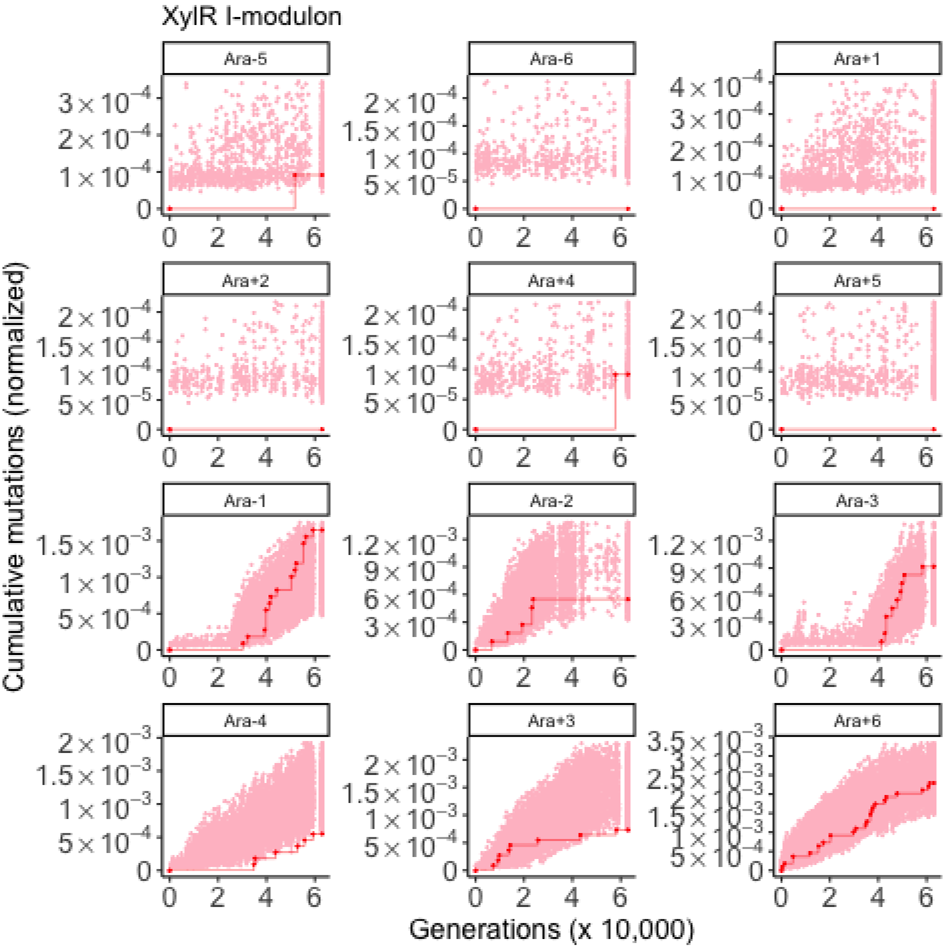

XylR I-modulon
Ara-5
Ara-6
Ara+1
Ara+2
Ara+4
Ara+5
Ara-1
Ara-2
Ara-3
Ara-4
Ara+3
Ara+6
Cumulative mutations (normalized)
Generations (x 10,000)

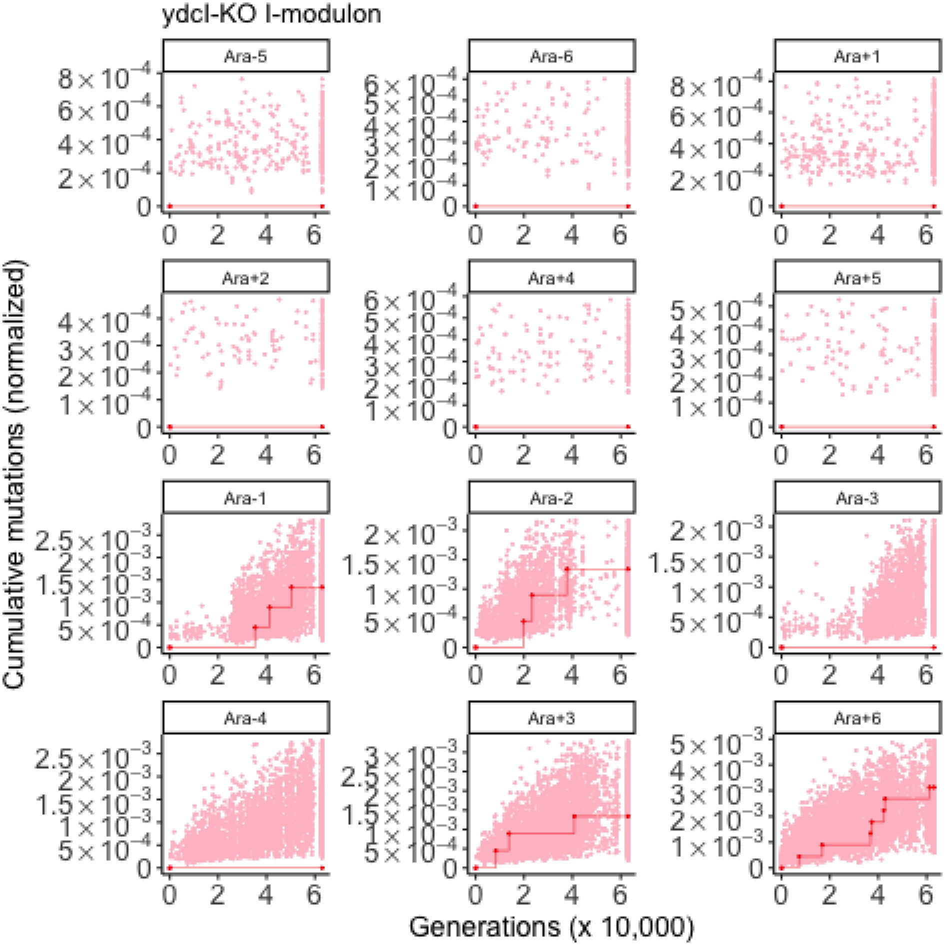

ydcI-KO I-modulon
Ara-5
Ara-6
Ara+1
Ara+2
Ara+4
Ara+5
Ara-1
Ara-2
Ara-3
Ara-4
Ara+3
Ara+6
Cumulative mutations (normalized)
Generations (x 10,000)

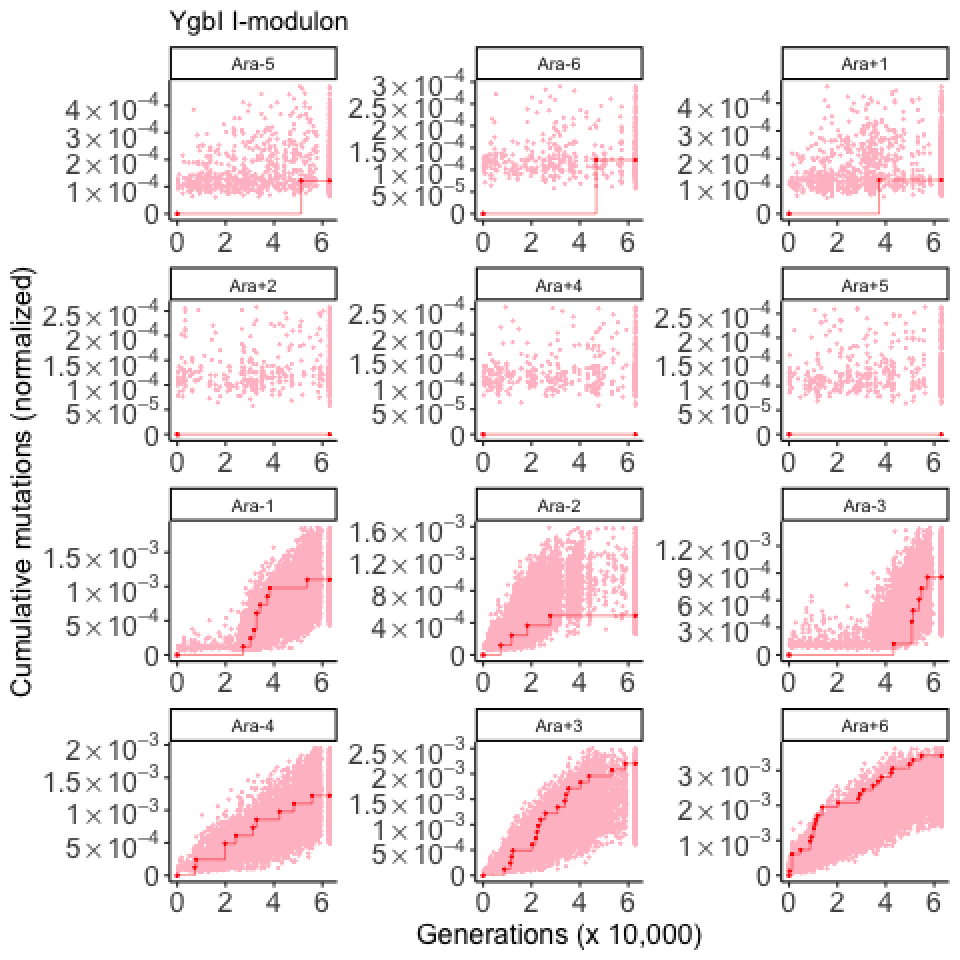

YgbI I-modulon
Ara-5
Ara-6
Ara+1
Ara+2
Ara+4
Ara+5
Ara-1
Ara-2
Ara-3
Ara-4
Ara+3
Ara+6
Cumulative mutations (normalized)
Generations (x 10,000)

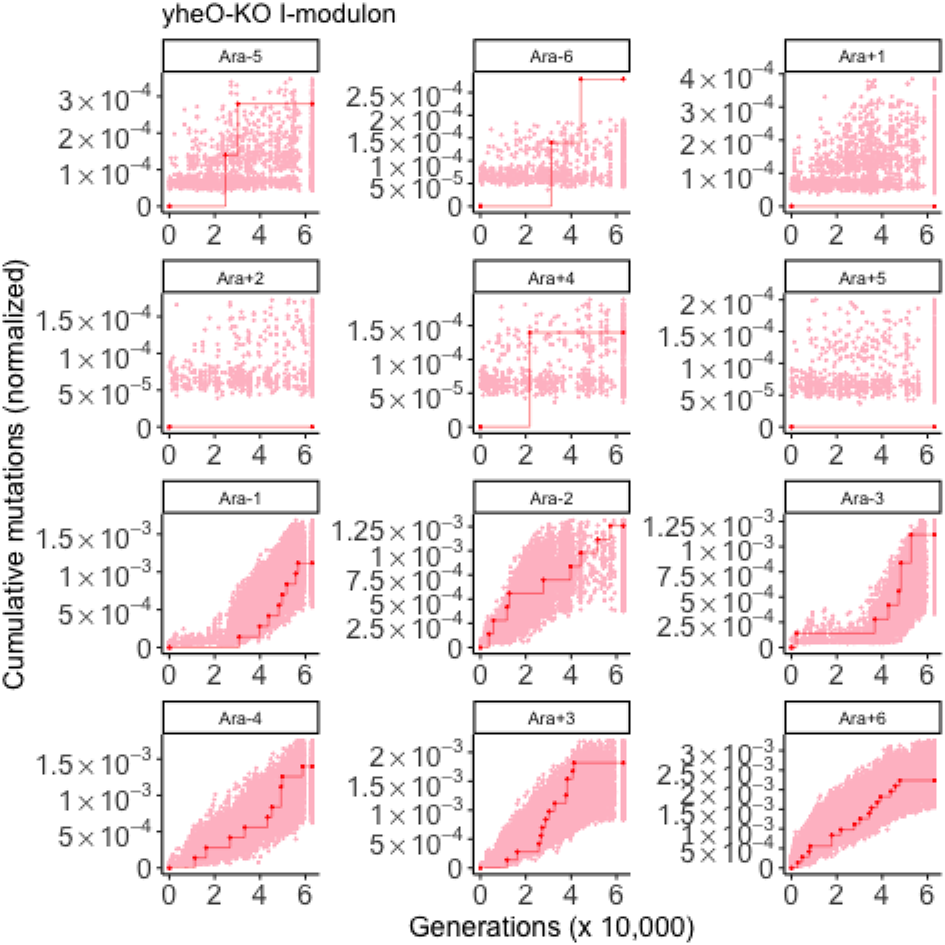

yheO-KO I-modulon
Ara-5
Ara-6
Ara+1
Ara+2
Ara+4
Ara+5
Ara-1
Ara-2
Ara-3
Ara-4
Ara+3
Ara+6
Cumulative mutations (normalized)
Generations (x 10,000)

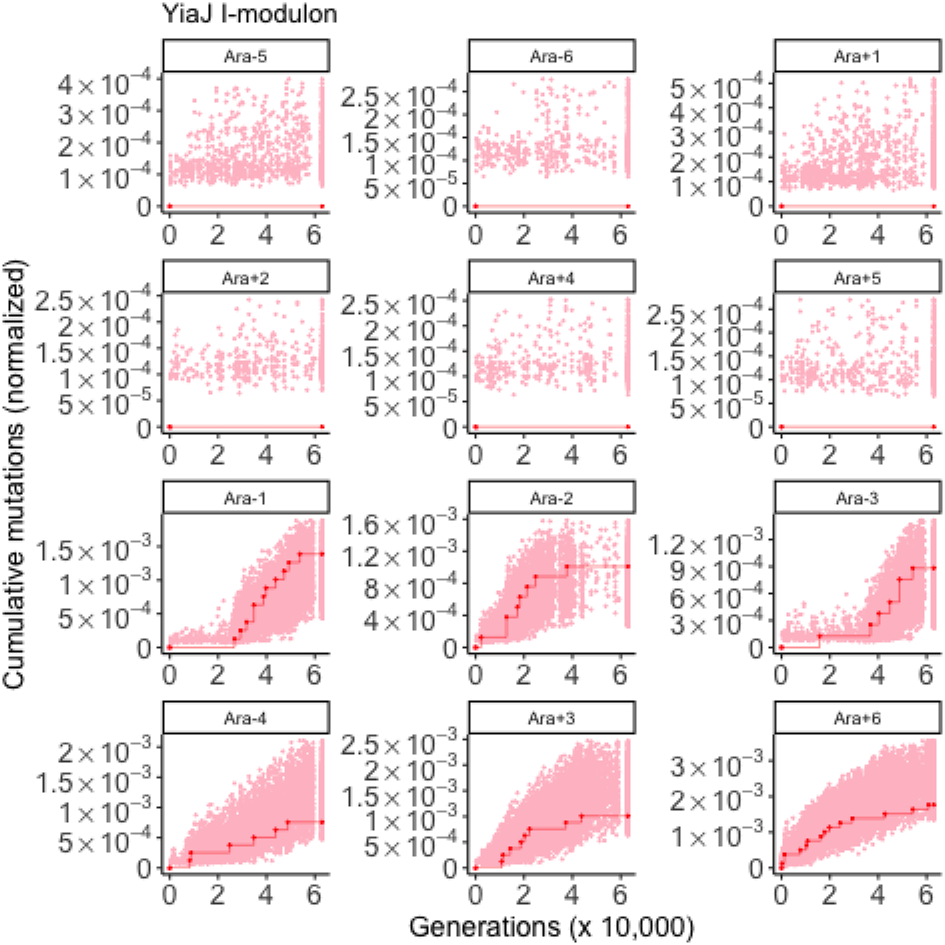

YiaJ I-modulon
Ara-5
Ara-6
Ara+1
Ara+2
Ara+4
Ara+5
Ara-1
Ara-2
Ara-3
Ara-4
Ara+3
Ara+6
Cumulative mutations (normalized)
Generations (x 10,000)

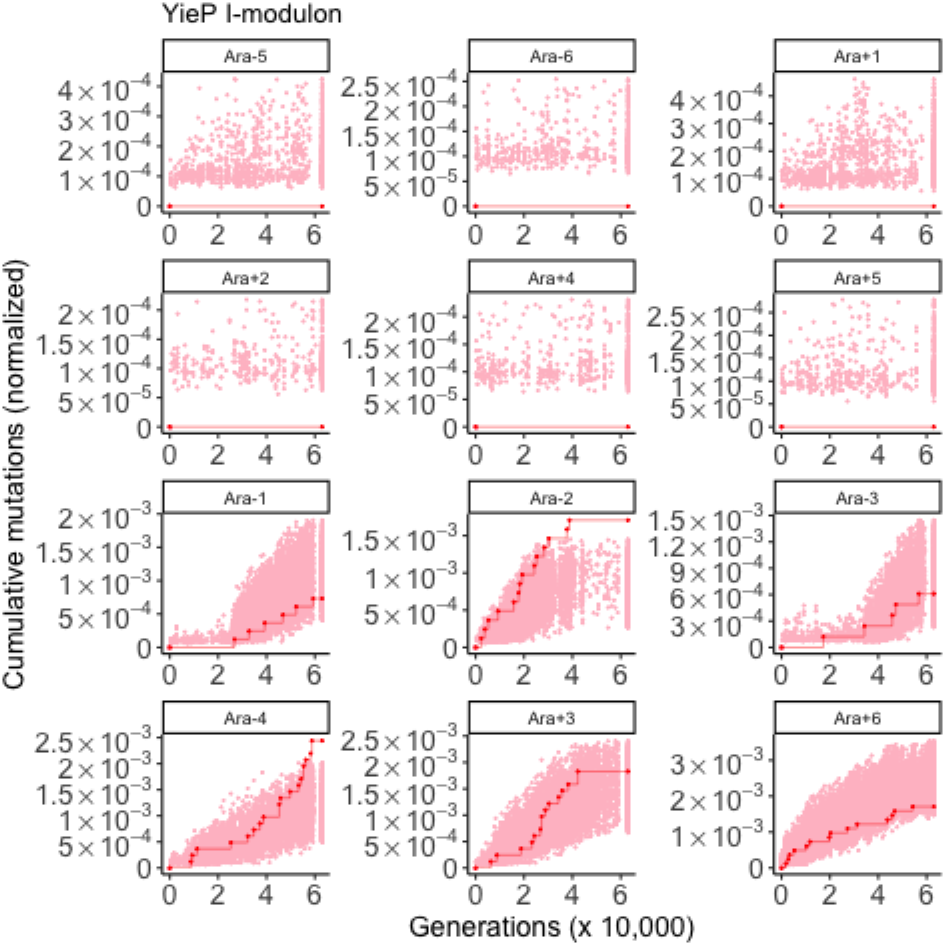
YieP I-modulon
Ara-5
Ara-6
Ara+1
Ara+2
Ara+4
Ara+5
Ara-1
Ara-2
Ara-3
Ara-4
Ara+3
Ara+6
Cumulative mutations (normalized)
Generations (x 10,000)

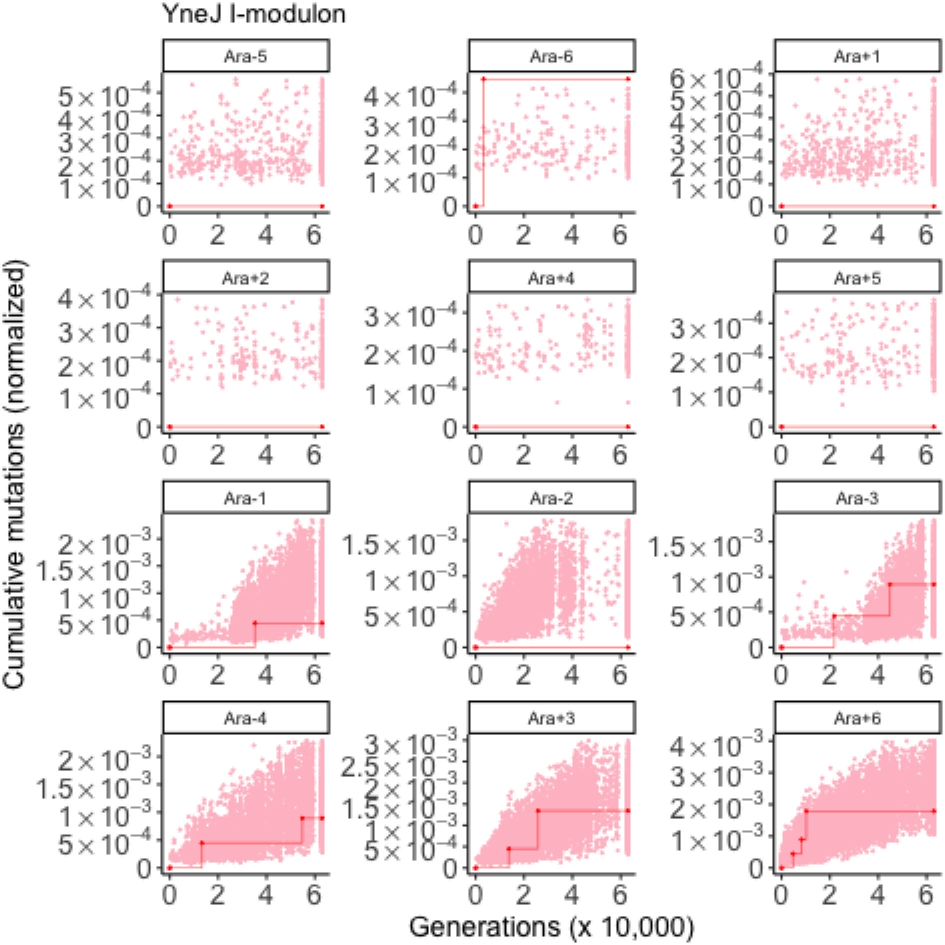

YneJ I-modulon
Ara-5
Ara-6
Ara+1
Ara+2
Ara+4
Ara+5
Ara-1
Ara-2
Ara-3
Ara-4
Ara+3
Ara+6
Cumulative mutations (normalized)
Generations (x 10,000)

Zinc I-modulon

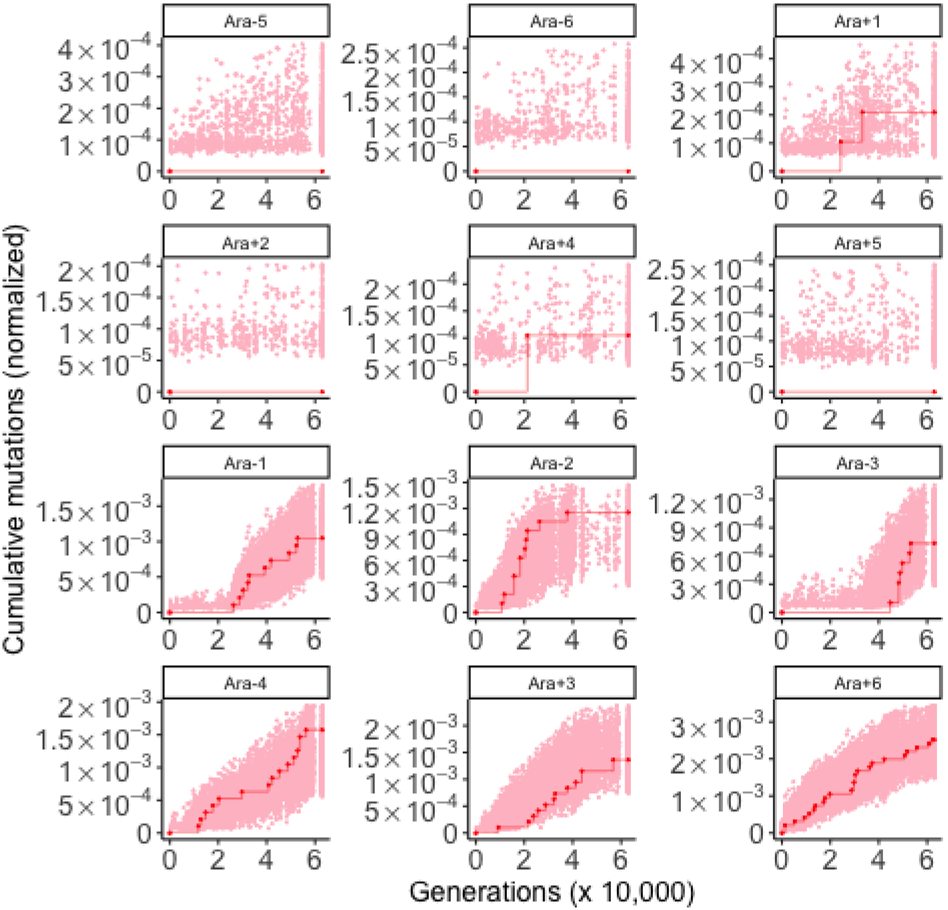

Supplement: S2 File — (PDF) [file pgen.1010324.s003.pdf]
