# Supplementary material for: Discovery of positive and purifying selection in metagenomic time series of hypermutator microbial populations
Source: PLoS Genet. 2022 Aug 18;18(8):e1010324. doi: 10.1371/journal.pgen.1010324 (PMC9426924; doi:10.1371/journal.pgen.1010324)
Supplement: S1 Table — (DOCX) [file pgen.1010324.s005.docx]

**S1 Table**

| **Gene** | **Locus tag** | **Gene length** | **Product** |
| --- | --- | --- | --- |
| *rlpB* | ECB_00610 | 582 | minor lipoprotein |
| *rplJ* | ECB_03861 | 498 | 50S ribosomal protein L10 |
| *ECB_01539* | ECB_01539 | 408 | protein similar to DicA regulator of DicB encoded by prophage CP-933O |
| *sdhD* | ECB_00682 | 348 | succinate dehydrogenase cytochrome b556 small membrane subunit |
| *rplV* | ECB_03166 | 333 | 50S ribosomal protein L22 |
| *rpsJ* | ECB_03172 | 312 | 30S ribosomal protein S10 |
| *rpsN* | ECB_03158 | 306 | 30S ribosomal protein S14 |
| *yrbB* | ECB_03056 | 294 | hypothetical protein |
| *groES* | ECB_04012 | 294 | co-chaperonin GroES |
| *rpsS* | ECB_03167 | 279 | 30S ribosomal protein S19 |
| *rpsT* | ECB_00027 | 264 | 30S ribosomal protein S20 |
| *rpsQ* | ECB_03162 | 255 | 30S ribosomal protein S17 |
| *atpE* | ECB_03621 | 240 | F0F1 ATP synthase subunit C |
| *rpmC* | ECB_03163 | 192 | 50S ribosomal protein L29 |
| *ydaE* | ECB_01329 | 171 | conserved protein |
| *rpmJ* | ECB_03150 | 117 | 50S ribosomal protein L36 |
| *ybgT* | ECB_00694 | 114 | hypothetical protein |
| *rhoL* | ECB_03660 | 102 | rho operon leader peptide |
