# Supplementary material for: Discovery of positive and purifying selection in metagenomic time series of hypermutator microbial populations
Source: PLoS Genet. 2022 Aug 18;18(8):e1010324. doi: 10.1371/journal.pgen.1010324 (PMC9426924; doi:10.1371/journal.pgen.1010324)
Supplement: S2 Table — (DOCX) [file pgen.1010324.s006.docx]

**S2 Table**

| **Gene** | **Locus tag** | **Gene length** | **Product** |
| --- | --- | --- | --- |
| *atpD* | ECB_03616 | 1383 | F0F1 ATP synthase subunit beta |
| *icdA* | ECB_01134 | 1251 | isocitrate dehydrogenase |
| *thyA* | ECB_02675 | 795 | thymidylate synthase |
| *plsC* | ECB_02890 | 738 | 1-acyl-sn-glycerol-3-phosphate acyltransferase |
| *pdxH* | ECB_01608 | 657 | pyridoxamine 5'-phosphate oxidase |
| *rplD* | ECB_03170 | 606 | 50S ribosomal protein L4 |
| *lpcA* | ECB_00217 | 579 | phosphoheptose isomerase |
| *rimM* | ECB_02497 | 549 | 16S rRNA-processing protein |
| *rplE* | ECB_03159 | 540 | 50S ribosomal protein L5 |
| *sixA* | ECB_02264 | 486 | phosphohistidine phosphatase |
| *folK* | ECB_00141 | 480 | 2-amino-4-hydroxy-6-hydroxymethyldihyropteridine pyrophosphokinase |
| *accB* | ECB_03113 | 471 | acetyl-CoA carboxylase |
| *holD* | ECB_04247 | 414 | DNA polymerase III subunit psi |
| *rpsI* | ECB_03090 | 393 | 30S ribosomal protein S9 |
| *iscU* | ECB_02421 | 387 | scaffold protein |
| *rplQ* | ECB_03145 | 384 | 50S ribosomal protein L17 |
| *panD* | ECB_00130 | 381 | aspartate 1-decarboxylase precursor |
| *rpsL* | ECB_03193 | 375 | 30S ribosomal protein S12 |
| *rplN* | ECB_03161 | 372 | 50S ribosomal protein L14 |
| *rnpA* | ECB_03587 | 360 | ribonuclease P |
| *rplT* | ECB_01685 | 357 | 50S ribosomal protein L20 |
| *yadR* | ECB_00155 | 345 | hypothetical protein |
| *fdx* | ECB_02417 | 336 | [2Fe-2S] ferredoxin |
| *secG* | ECB_03040 | 333 | protein-export membrane protein |
| *ybaB* | ECB_00422 | 330 | hypothetical protein |
| *acpP* | ECB_01090 | 237 | acyl carrier protein |
| *rpmI* | ECB_01686 | 198 | 50S ribosomal protein L35 |
| *rpmH* | ECB_03586 | 141 | 50S ribosomal protein L34 |
| *pheL* | ECB_02487 | 48 | pheA gene leader peptide |
| *trpL* | ECB_01239 | 45 | trp operon leader peptide |
