# Supplementary material for: Discovery of positive and purifying selection in metagenomic time series of hypermutator microbial populations
Source: PLoS Genet. 2022 Aug 18;18(8):e1010324. doi: 10.1371/journal.pgen.1010324 (PMC9426924; doi:10.1371/journal.pgen.1010324)
Supplement: S3 Table — (DOCX) [file pgen.1010324.s007.docx]

**S3 Table**

| **Page^*^** | **I-modulon** | **Populations of Interest** | **Hypothesis** |
| --- | --- | --- | --- |
| 3  11  13  16  18  19 | ArcA-2  CpxR  Crp-1  CsqR  CysB  Deletion-1 | Ara−2, Ara−3  Ara−3, Ara−4, Ara+6  Ara−5, Ara+1  Ara+3, Ara+6  Ara−1, Ara+6  Ara−3 | purifying selection  positive and purifying selection  positive selection  purifying selection  purifying selection  positive selection |
| 22 | Duplication-1 | Ara−2, Ara−3, Ara−4, Ara+3 | strong purifying selection |
| 23 | e14-deletion | Ara−3 | strong positive selection |
| 24 | efeU-repair | Ara+3, Ara+6 | strong positive selection, purifying selection |
| 28  30  31 | FadR  fimbriae  FlhDC | Ara−1, Ara−2, Ara+6  Ara−2  Ara−2, Ara+6 | purifying selection  strong positive selection  positive selection |
| 37 | fur-KO | Ara−3 | strong positive selection |
| 38  40 | GadEWX  GadWX-KO | Ara−1, Ara−3, Ara+6  Ara+6 | positive selection  positive selection |
| 46 | insertion | Ara−1, Ara+6 | strong positive selection |
| 48  49  50  53  63  69  72  77 | Leu/Ile  lipopolysaccharide  Lrp  MetJ  PurR-1  RcsAB  sgrT  translation | Ara−1, Ara−4, Ara+3, Ara+6  Ara−1  Ara+6  Ara−4, Ara+3, Ara+6  Ara−1  Ara−3, Ara+3  Ara−1, Ara−4,  Ara+1, Ara+2, Ara+5, Ara−3, Ara+6 | strong purifying selection  strong positive selection  purifying selection  purifying selection  purifying selection  purifying selection  purifying selection  positive and purifying selection |
| 79  81 | uncharacterized-1  uncharacterized-3 | Ara+2, Ara+5, Ara+3  Ara−1 | positive selection  positive selection |
| 82  90 | uncharacterized-4  YieP | Ara−6, Ara+1, Ara+2, Ara+4, Ara+5, Ara−2, Ara−3, Ara−4, Ara+3, Ara+6  Ara−2, Ara−4 | strong positive and purifying selection  positive selection |

^*^Page numbers refers to Supplementary Files 1 (STIMS across all LTEE populations) and Supplementary File 2 (STIMS on individual LTEE populations).
