# Supplementary material for: Discovery of positive and purifying selection in metagenomic time series of hypermutator microbial populations
Source: PLoS Genet. 2022 Aug 18;18(8):e1010324. doi: 10.1371/journal.pgen.1010324 (PMC9426924; doi:10.1371/journal.pgen.1010324)
Supplement: S4 Table — (DOCX) [file pgen.1010324.s008.docx]

**S4 Table**

| **I-modulon** | **FDR-corrected *p*-value** | **Inference** |
| --- | --- | --- |
| CysB | *p* < 10^−5^ | purifying selection |
| Leu/Ile | *p* < 10^−5^ | purifying selection |
| Duplication-1 | *p* < 10^−4^ | purifying selection |
| fimbrae | *p* < 10^−4^ | purifying selection |
| RcsAB | *p* = 0.000172 | purifying selection |
| Crp-1 | *p* = 0.000317 | positive selection |
| flu-yeeRS | *p* = 0.000636 | positive selection |
| MetJ | *p* = 0.000877 | purifying selection |
| FadR | *p* = 0.000989 | purifying selection |
| Pyruvate | *p* = 0.00241 | purifying selection |
| translation | *p* = 0.00268 | purifying selection |
| CsqR | *p* = 0.00288 | purifying selection |
| Lrp | *p* = 0.00347 | purifying selection |
| ArcA-2 | *p* = 0.00358 | purifying selection |
| GadEWX | *p* = 0.00369 | positive selection |
| Insertion | *p* = 0.00775 | positive selection |
