# Supplementary figures and images for: Discovery of positive and purifying selection in metagenomic time series of hypermutator microbial populations
Source: PLoS Genet. 2022 Aug 18;18(8):e1010324. doi: 10.1371/journal.pgen.1010324 (PMC9426924; doi:10.1371/journal.pgen.1010324)

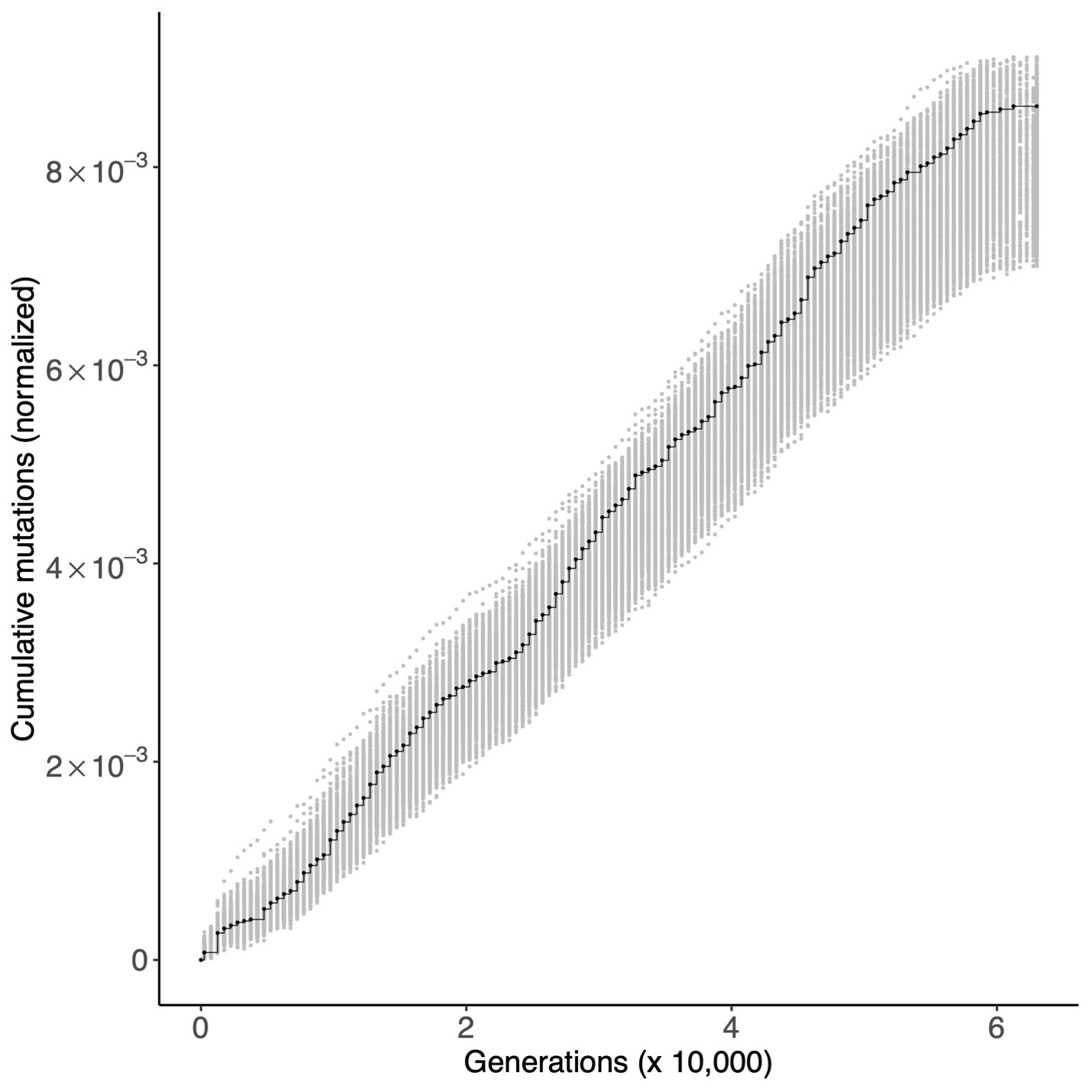

Supplement: S1 Fig — Each panel shows the cumulative number of mutations in the gold-standard neutral genes (black) in all 12 LTEE populations. For comparison, random sets of 57 genes were sampled 1,000 times, and the cumulative number of mutations in those random gene sets, normalized by gene length, were calculated. The middle 95% of this null distribution is shown in gray, in order to show a two-tailed statistical comparison of the cumulative number of mutations in the gold-standard neutral gene set to the null distribution at α = 0.05. (JPG) [file pgen.1010324.s009.jpg]

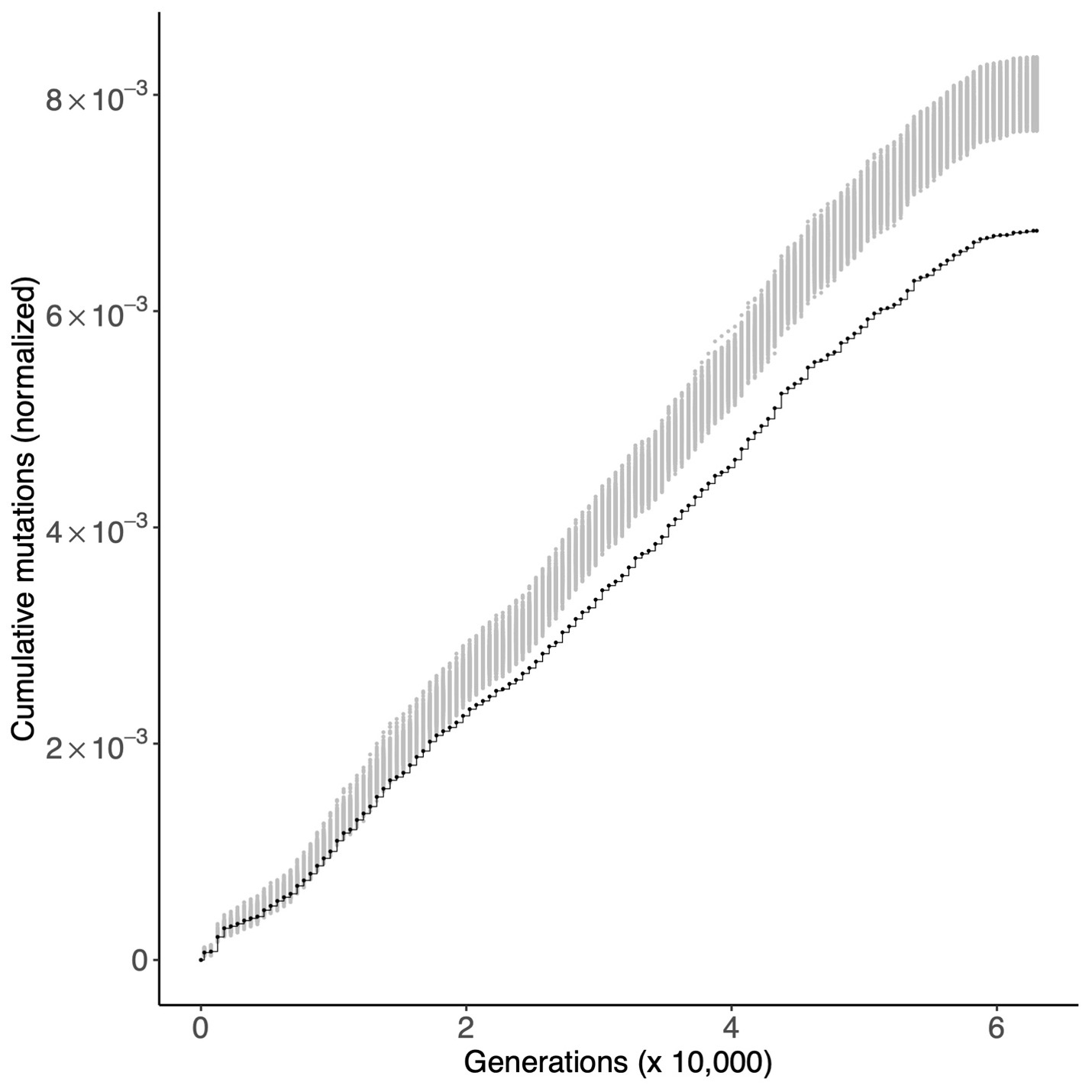

Supplement: S2 Fig — Each panel shows the cumulative number of mutations in the gold standard set of genes under purifying selection (black) in all 12 LTEE populations. For comparison, random sets of 490 genes were sampled 1,000 times, and the cumulative number of mutations in those random gene sets, normalized by gene length, were calculated. The middle 95% of this null distribution is shown in gray. (JPG) [file pgen.1010324.s010.jpg]

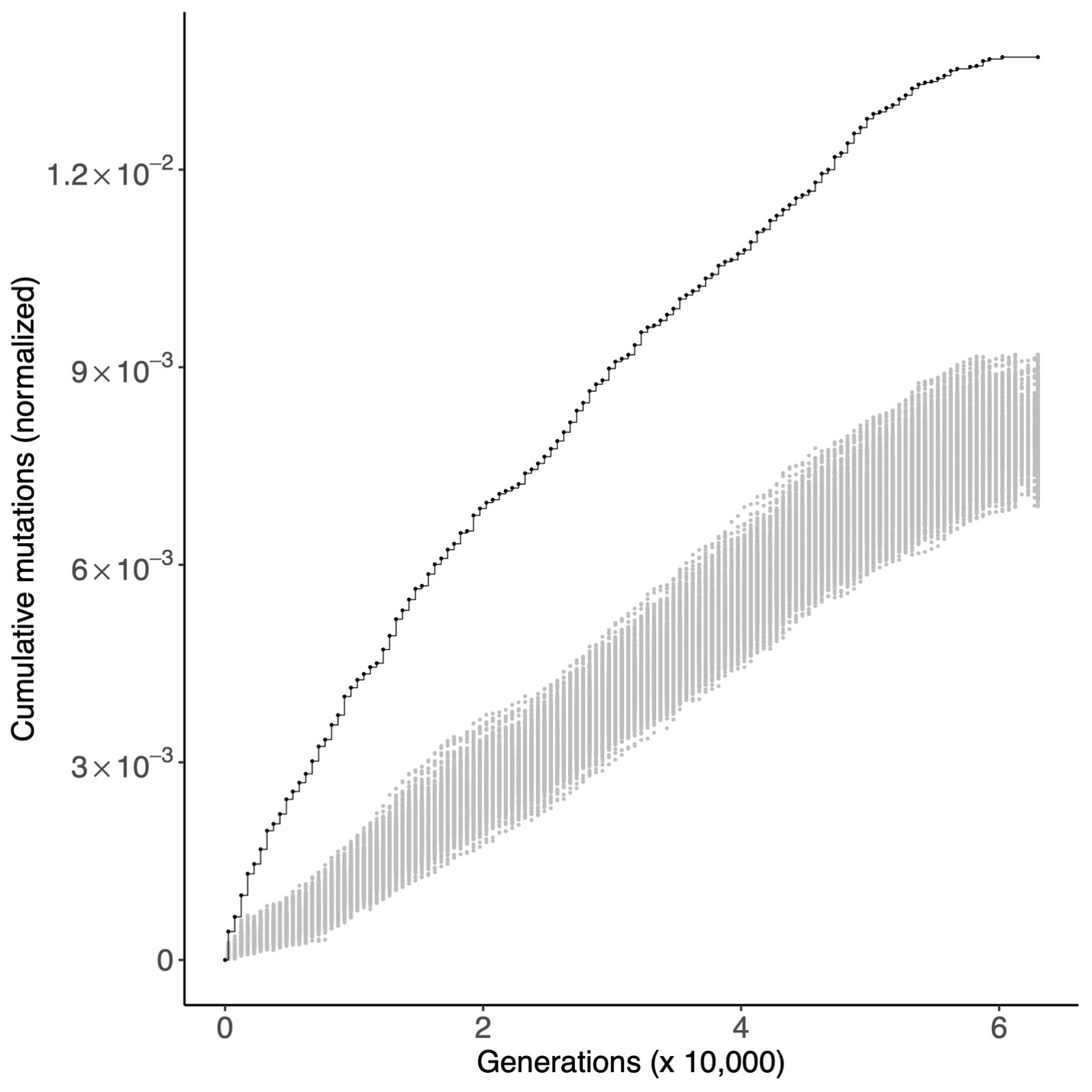

Supplement: S3 Fig — Each panel shows the cumulative number of mutations in the gold standard set of genes under positive selection (black) in all 12 LTEE populations. These genes are the 50 genes with the strongest signal of parallel evolution in clones isolated from the six nonmutator populations, as scored by Tenaillon and colleagues [15]. For comparison, random sets of 50 genes were sampled 1,000 times, and the cumulative number of mutations in those random gene sets, normalized by gene length, were calculated. The middle 95% of this null distribution is shown in gray. (JPG) [file pgen.1010324.s011.jpg]

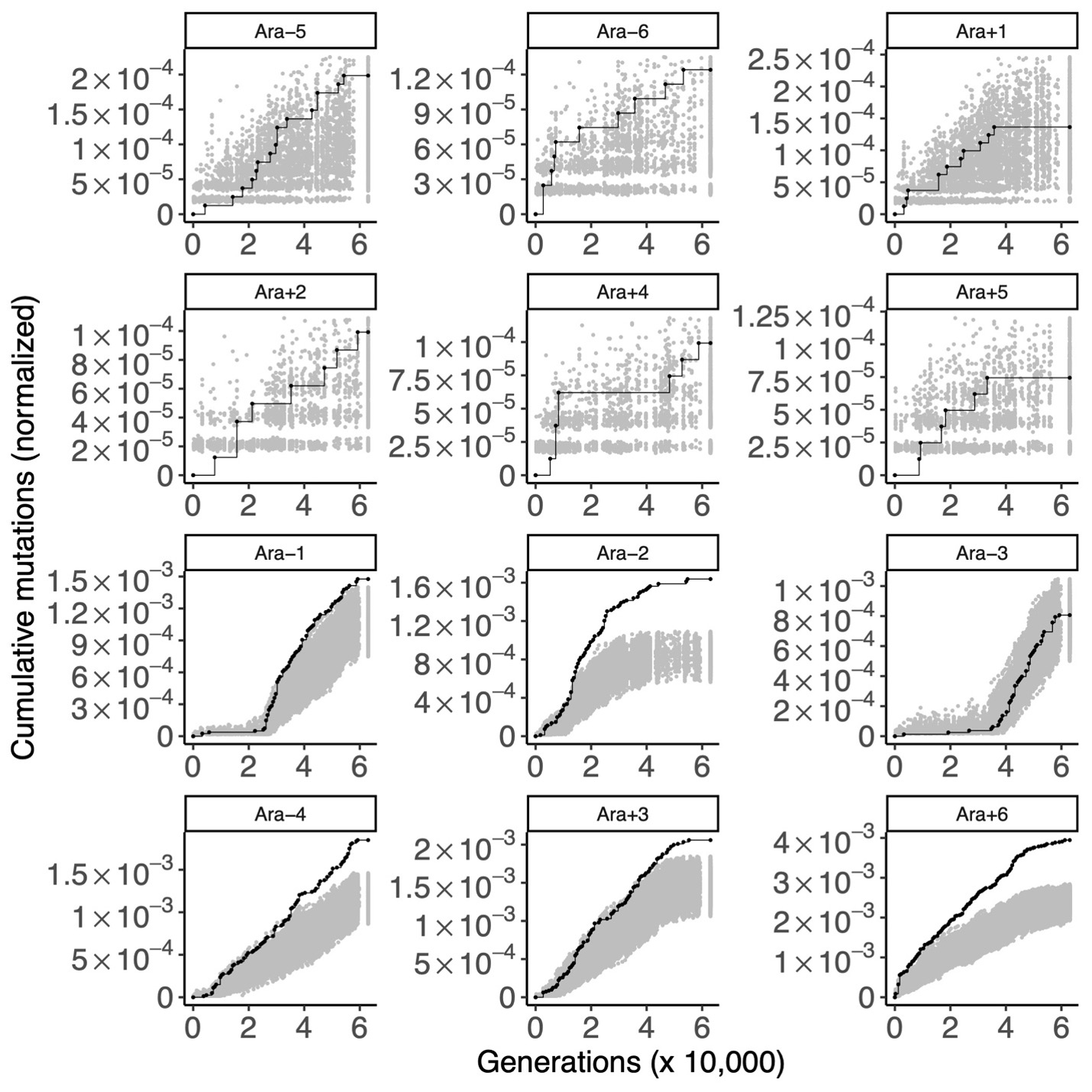

Supplement: S4 Fig — Each panel shows the cumulative number of mutations in the 50 genes with the strongest signal of parallel evolution in clones isolated from the six hypermutator populations, as scored by Tenaillon and colleagues [15], in the 12 LTEE populations. These curves are drawn in black. For comparison, random sets of 50 genes were sampled 1,000 times, and the cumulative number of mutations in those random gene sets, normalized by gene length, were calculated. The middle 95% of this null distribution is shown in gray. The top six panels are the nonmutator populations and the bottom six panels are the hypermutator populations. (JPG) [file pgen.1010324.s012.jpg]

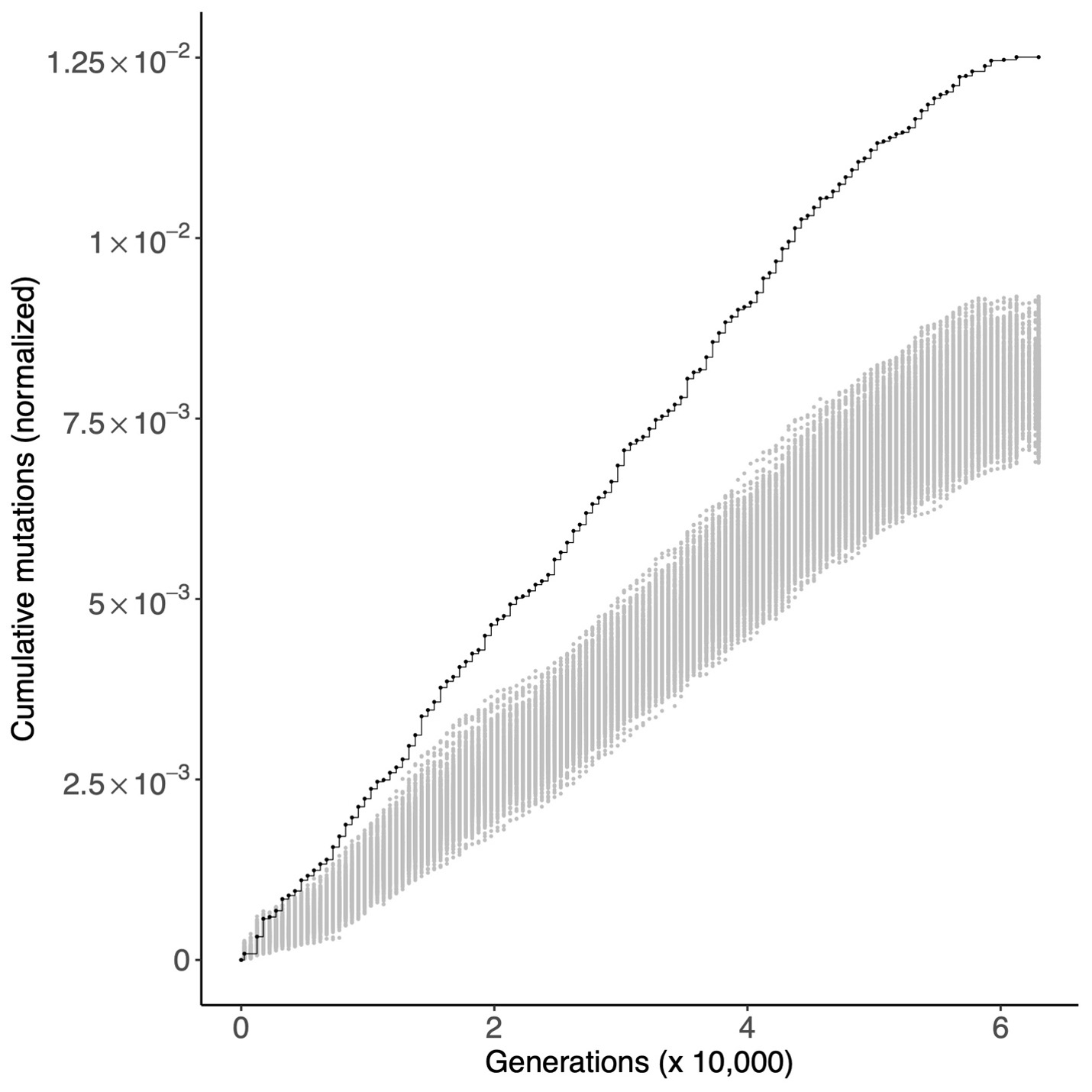

Supplement: S5 Fig — Each panel shows the cumulative number of mutations in the 50 genes with the strongest signal of parallel evolution in clones isolated from the six hypermutator populations, as scored by Tenaillon and colleagues [15], in all 12 LTEE populations. These curves are drawn in black. For comparison, random sets of 50 genes were sampled 1,000 times, and the cumulative number of mutations in those random gene sets, normalized by gene length, were calculated. The middle 95% of this null distribution is shown in gray. (JPG) [file pgen.1010324.s013.jpg]

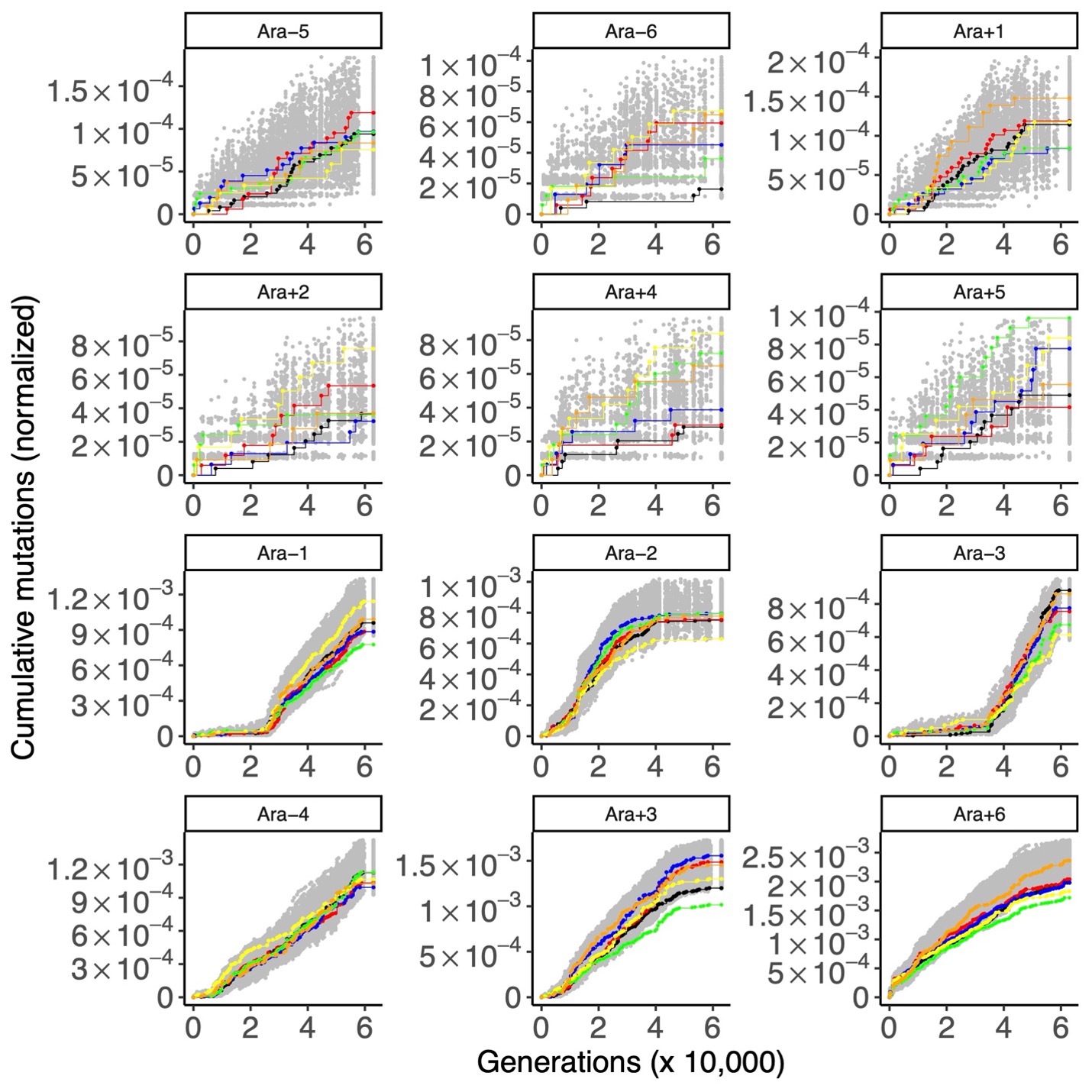

Supplement: S6 Fig — Each panel shows the cumulative number of mutations in the A-sector (black), S-sector (red), O-sector (blue), U-sector (green), R-sector (yellow), and C-sector (orange) in the 12 LTEE populations. For comparison, random sets of 92 genes (i.e. the smallest proteome sector cardinality) were sampled 1,000 times, and the cumulative number of mutations in those random gene sets, normalized by gene length, were calculated. The middle 95% of this null distribution is shown in gray. The top six panels are the nonmutator populations and the bottom six panels are the hypermutator populations. (JPG) [file pgen.1010324.s014.jpg]

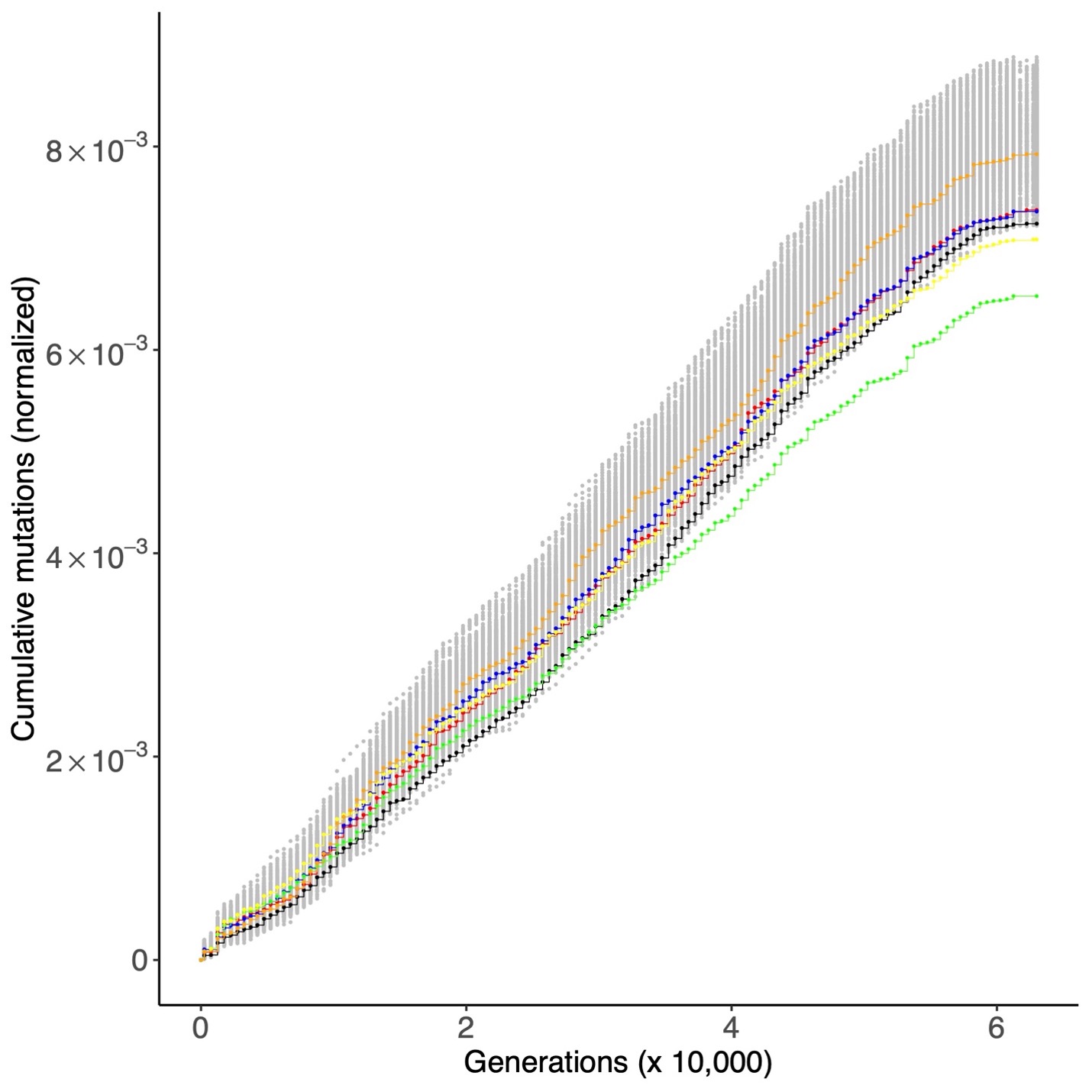

Supplement: S7 Fig — Each panel shows the cumulative number of mutations in the A-sector (black), S-sector (red), O-sector (blue), U-sector (green), R-sector (yellow), and C-sector (orange) in all 12 LTEE populations. For comparison, random sets of 92 genes (i.e. the smallest proteome sector cardinality) were sampled 1,000 times, and the cumulative number of mutations in those random gene sets, normalized by gene length, were calculated. The middle 95% of this null distribution is shown in gray. (JPG) [file pgen.1010324.s015.jpg]

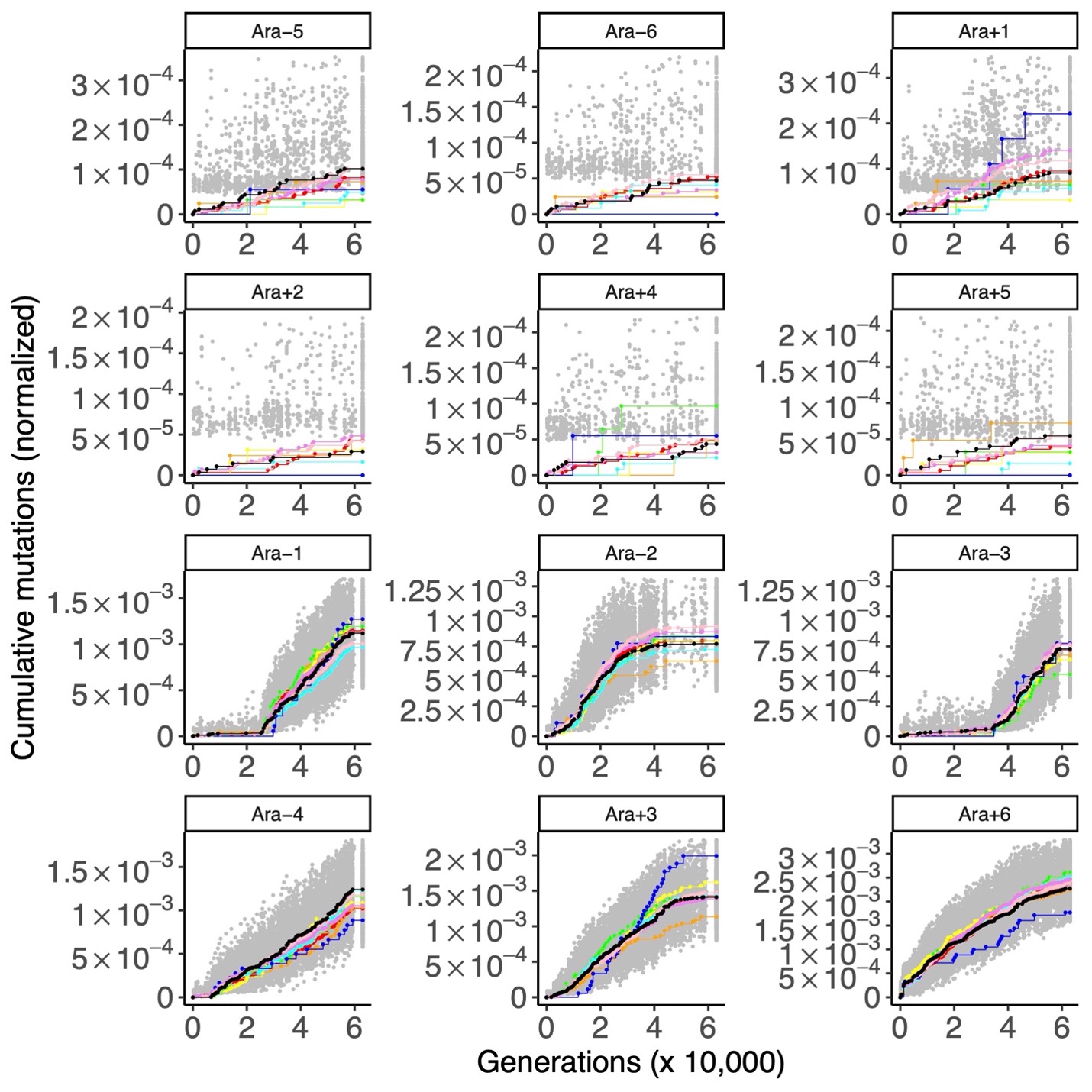

Supplement: S8 Fig — Each panel shows the cumulative number of mutations in eigengene 1 (red), eigengene 2 (orange), eigengene 3 (yellow), eigengene 4 (green), eigengene 5 (cyan), eigengene 6 (blue), eigengene 7 (violet), eigengene 8 (pink), and eigengene 9 (black) in the 12 LTEE populations. For comparison, random sets of 15 genes (i.e. the smallest eigengene cardinality) were sampled 1,000 times, and the cumulative number of mutations in those random gene sets, normalized by gene length, were calculated. The middle 95% of this null distribution is shown in gray. The top six panels are the nonmutator populations and the bottom six panels are the hypermutator populations. (JPG) [file pgen.1010324.s016.jpg]

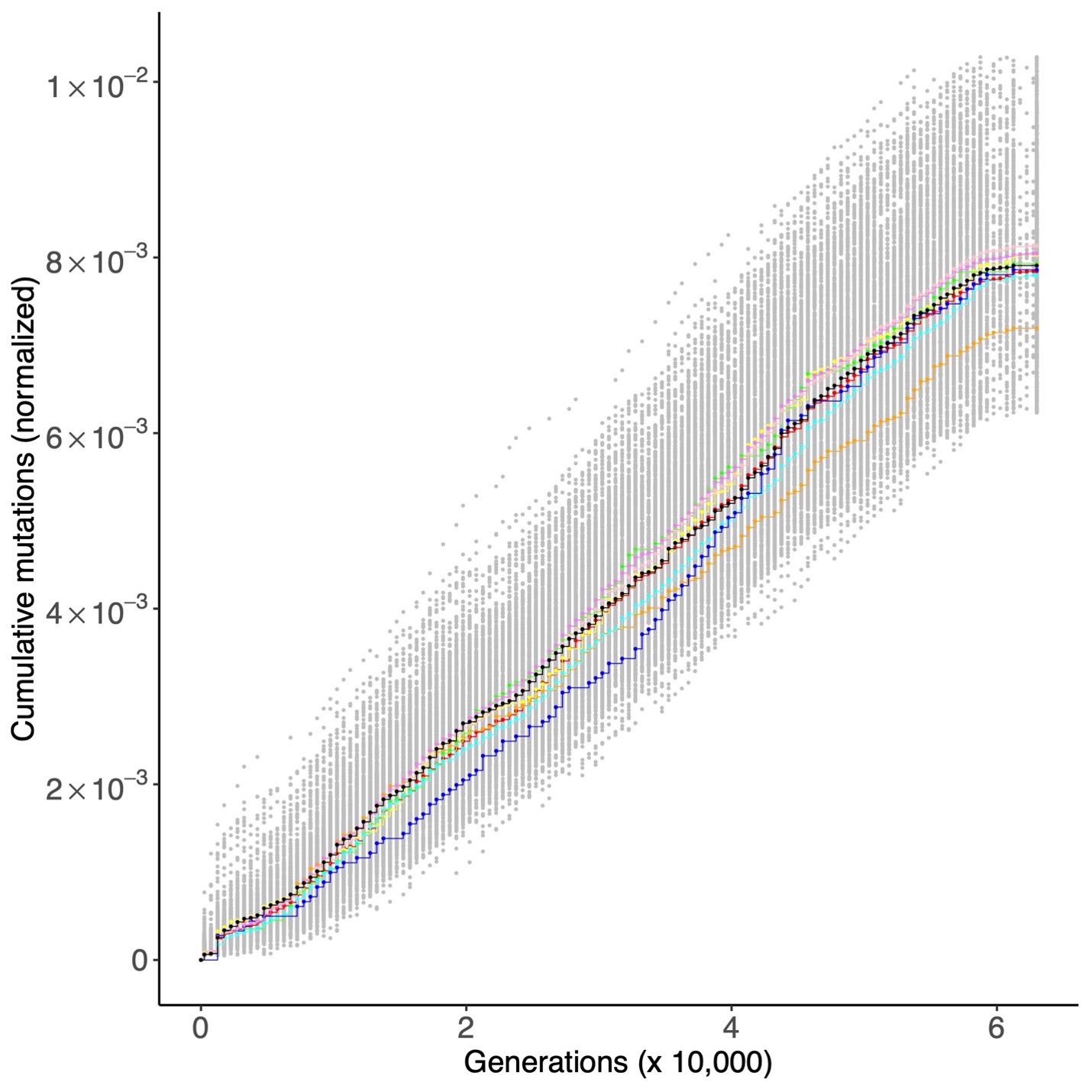

Supplement: S9 Fig — Each panel shows the cumulative number of mutations in eigengene 1 (red), eigengene 2 (orange), eigengene 3 (yellow), eigengene 4 (green), eigengene 5 (cyan), eigengene 6 (blue), eigengene 7 (violet), eigengene 8 (pink), and eigengene 9 (black) in the 12 LTEE populations. For comparison, random sets of 15 genes (i.e. the smallest eigengene cardinality) were sampled 1,000 times, and the cumulative number of mutations in those random gene sets, normalized by gene length, were calculated. (JPG) [file pgen.1010324.s017.jpg]

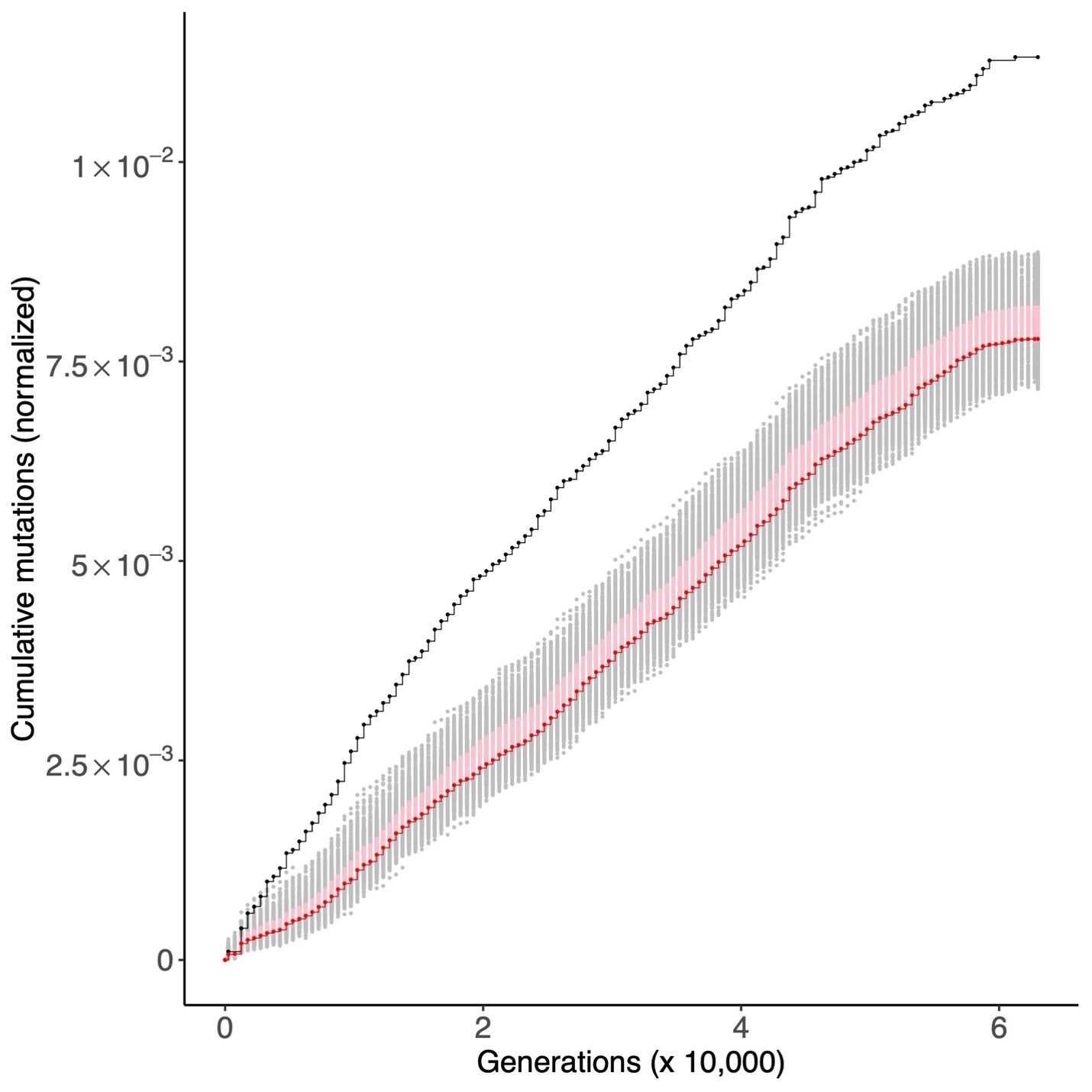

Supplement: S10 Fig — Each panel shows the cumulative number of mutations in 56 I-modulon regulators (black) and in 1,061 genes regulated by I-modulon regulators (red) in the 12 LTEE populations. For comparison to the I-modulon regulators, random sets of 56 genes were sampled 1,000 times, and the cumulative number of mutations in those random gene sets, normalized by gene length, were calculated. The middle 95% of this null distribution is shown in gray. A similar procedure was used with random sets of 1,061 genes to make a comparable distribution in pink to compare to the genes regulated by I-modulon regulators. (JPG) [file pgen.1010324.s018.jpg]

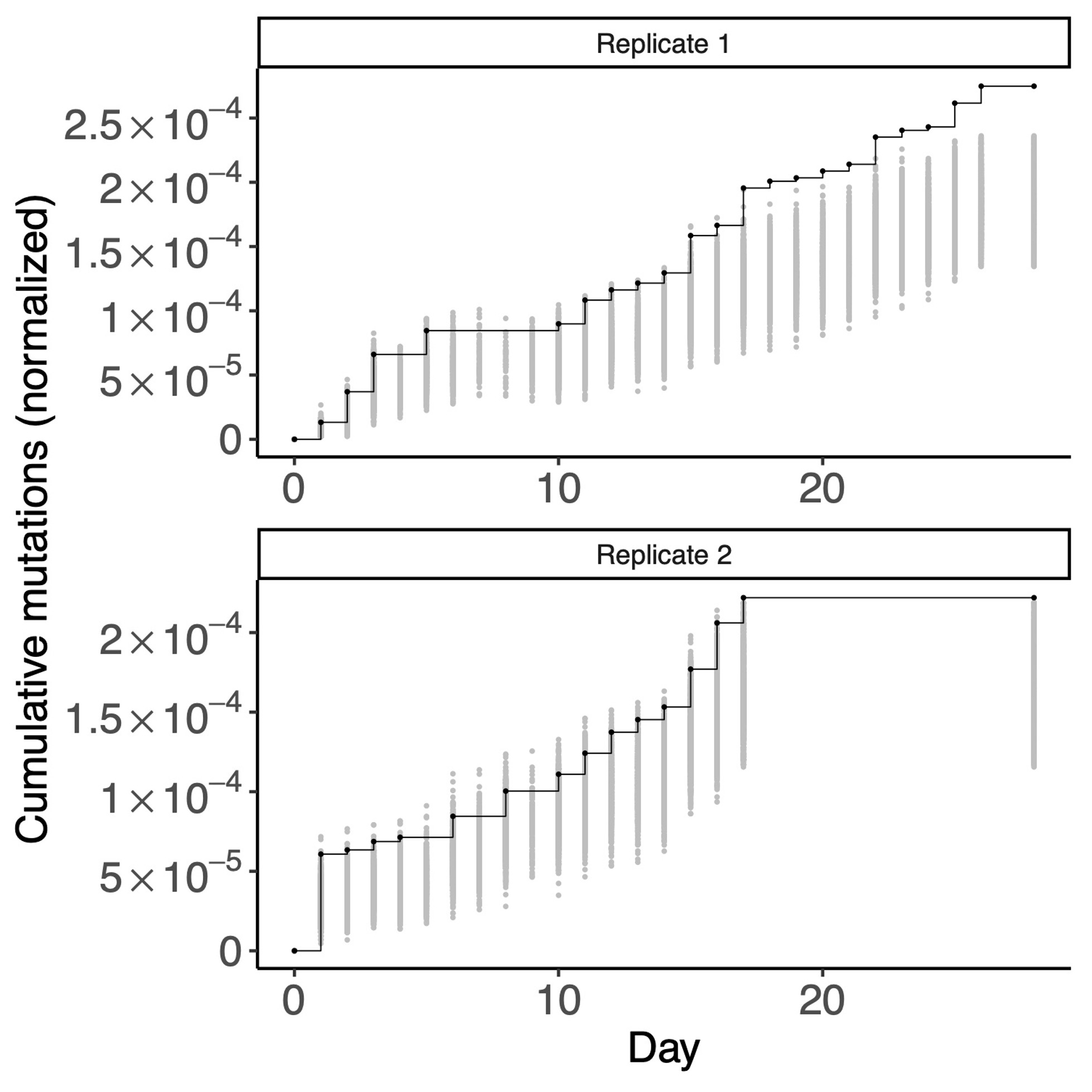

Supplement: S11 Fig — Each panel shows the cumulative number of mutations in 424 annotated regulatory genes (black), in two replicate populations of hypermutator P. aeruginosa. For comparison, random sets of 424 genes were sampled 1,000 times, and the cumulative number of mutations in those random gene sets, normalized by gene length, were calculated. The middle 95% of this null distribution is shown in gray. (JPG) [file pgen.1010324.s019.jpg]
